# Shared polygenic risk and causal inferences in amyotrophic lateral sclerosis

**DOI:** 10.1002/ana.25431

**Published:** 2019-03-13

**Authors:** Sara Bandres‐Ciga, Alastair J. Noyce, Gibran Hemani, Aude Nicolas, Andrea Calvo, Gabriele Mora, The ITALSGEN Consortium, Alessandro Arosio, Marco Barberis, Ilaria Bartolomei, Stefania Battistini, Michele Benigni, Giuseppe Borghero, Maura Brunetti, Andrea Calvo, Stefania Cammarosano, Antonino Cannas, Antonio Canosa, Margherita Capasso, Claudia Caponnetto, Carla Caredda, Paola Carrera, Federico Casale, Sebastiano Cavallaro, Adriano Chiò, Tiziana Colletti, Francesca L. Conforti, Amelia Conte, Lucia Corrado, Emanuela Costantino, Sandra D'Alfonso, Antonio Fasano, Cinzia Femiano, Carlo Ferrarese, Nicola Fini, Gianluca Floris, Giuseppe Fuda, Fabio Giannini, Maurizio Grassano, Antonio Ilardi, Vincenzo La Bella, Serena Lattante, Giancarlo Logroscino, Francesco O. Logullo, Daniela Loi, Christian Lunetta, Gianluigi Mancardi, Paola Mandich, Jessica Mandrioli, Umberto Manera, Giuseppe Marangi, Kalliopi Marinou, Giuseppe Marrali, Maria Giovanna Marrosu, Letizia Mazzini, Maurizio Melis, Sonia Messina, Cristina Moglia, Maria Rosaria Monsurro, Gabriele Mora, Lorena Mosca, Patrizia Occhineri, Paola Origone, Carla Pani, Silvana Penco, Antonio Petrucci, Giovanni Piccirillo, Angelo Pirisi, Fabrizio Pisano, Maura Pugliatti, Gabriella Restagno, Claudia Ricci, Maria Rita Murru, Nilo Riva, Mario Sabatelli, Fabrizio Salvi, Marialuisa Santarelli, Riccardo Sideri, Isabella Simone, Rossella Spataro, Raffaella Tanel, Gioacchino Tedeschi, Stefania Tranquilli, Lucio Tremolizzo, Francesca Trojsi, Paolo Volanti, Marcella Zollino, The International ALS Genomics Consortium, Yevgeniya Abramzon, Sampath Arepalli, Robert H. Baloh, Robert Bowser, Christopher B. Brady, Alexis Brice, James Broach, Roy H. Campbell, William Camu, Ruth Chia, Adriano Chiò, John Cooper‐Knock, Daniele Cusi, Jinhui Ding, Carsten Drepper, Vivian E. Drory, Travis L. Dunckley, John D. Eicher, Faraz Faghri, Eva Feldman, Mary Kay Floeter, Pietro Fratta, Joshua T. Geiger, Glenn Gerhard, J. Raphael Gibbs, Summer B. Gibson, Jonathan D. Glass, John Hardy, Matthew B. Harms, Terry D. Heiman‐Patterson, Dena G. Hernandez, Lilja Jansson, Freya Kamel, Janine Kirby, Neil W. Kowall, Hannu Laaksovirta, Francesco Landi, Isabelle Le Ber, Serge Lumbroso, Daniel J.L. MacGowan, Nicholas J. Maragakis, Kevin Mouzat, Natalie A. Murphy, Liisa Myllykangas, Mike A. Nalls, Aude Nicolas, Richard W. Orrell, Lyle W. Ostrow, Roger Pamphlett, Stuart Pickering‐Brown, Erik Pioro, Hannah A. Pliner, Stefan M. Pulst, John M. Ravits, Alan E. Renton, Alberto Rivera, Wim Robbrecht, Ekaterina Rogaeva, Sara Rollinson, Jeffrey D. Rothstein, Erika Salvi, Sonja W. Scholz, Michael Sendtner, Pamela J. Shaw, Katie C. Sidle, Zachary Simmons, Andrew B. Singleton, David C. Stone, Raimo Sulkava, Pentti J. Tienari, Bryan J. Traynor, John Q. Trojanowski, Juan C. Troncoso, Philip Van Damme, Vivianna M. Van Deerlin, Ludo Van Den Bosch, Lorne Zinman, Pentti J. Tienari, David J. Stone, Mike A. Nalls, Andrew B. Singleton, Adriano Chiò, Bryan J. Traynor

**Affiliations:** ^1^ Molecular Genetics Section, Laboratory of Neurogenetics National Institute on Aging, National Institutes of Health Bethesda MD; ^2^ Instituto de Investigación Biosanitaria de Granada (ibs.GRANADA) Granada Spain; ^3^ Preventive Neurology Unit, Wolfson Institute of Preventive Medicine Queen Mary University of London London United Kingdom; ^4^ Department of Clinical and Movement Neurosciences University College London, Institute of Neurology London United Kingdom; ^5^ MRC Integrative Epidemiology Unit University of Bristol Bristol United Kingdom; ^6^ Neuromuscular Diseases Research Section, Laboratory of Neurogenetics National Institute on Aging, National Institutes of Health Bethesda MD; ^7^ ‘Rita Levi Montalcini’ Department of Neuroscience University of Turin Turin Italy; ^8^ ALS Center Istituti Clinici Scientifici Maugeri, IRCCS Milan Italy; ^9^ Department of Neurology, Helsinki University Hospital and Molecular Neurology Programme, Biomedicum University of Helsinki Helsinki Finland; ^10^ Genetics and Pharmacogenomics, Merck Research Laboratories Merck & Co., Inc. West Point PA; ^11^ Data Tecnica International Glen Echo MD; ^12^ Institute of Cognitive Sciences and Technologies C.N.R Rome Italy; ^13^ Azienda Ospedaliero Universitaria Città della Salute e della Scienza Turin Italy; ^14^ Department of Neurology Johns Hopkins University Baltimore MD

## Abstract

**Objective:**

To identify shared polygenic risk and causal associations in amyotrophic lateral sclerosis (ALS).

**Methods:**

Linkage disequilibrium score regression and Mendelian randomization were applied in a large‐scale, data‐driven manner to explore genetic correlations and causal relationships between >700 phenotypic traits and ALS. Exposures consisted of publicly available genome‐wide association studies (GWASes) summary statistics from *MR Base* and *LD‐hub*. The outcome data came from the recently published ALS GWAS involving 20,806 cases and 59,804 controls. Multivariate analyses, genetic risk profiling, and Bayesian colocalization analyses were also performed.

**Results:**

We have shown, by linkage disequilibrium score regression, that ALS shares polygenic risk genetic factors with a number of traits and conditions, including positive correlations with smoking status and moderate levels of physical activity, and negative correlations with higher cognitive performance, higher educational attainment, and light levels of physical activity. Using Mendelian randomization, we found evidence that hyperlipidemia is a causal risk factor for ALS and localized putative functional signals within loci of interest.

**Interpretation:**

Here, we have developed a public resource (https://lng-nia.shinyapps.io/mrshiny) which we hope will become a valuable tool for the ALS community, and that will be expanded and updated as new data become available. Shared polygenic risk exists between ALS and educational attainment, physical activity, smoking, and tenseness/restlessness. We also found evidence that elevated low‐desnity lipoprotein cholesterol is a causal risk factor for ALS. Future randomized controlled trials should be considered as a proof of causality. Ann Neurol 2019;85:470–481

Amyotrophic lateral sclerosis (ALS; OMIM #105400) is a progressive, fatal neurodegenerative disease. Symptom onset of ALS peaks in the mid‐sixties, and most patients succumb to the disease within 2 to 5 years of becoming symptomatic.^1^ Prevalence of ALS is projected to nearly double by 2040, primarily attributed to aging of the global population.^2^

Despite considerable advances made in understanding the genetic architecture underlying ALS,^3^, ^4^ the contribution of lifestyle factors and of disease‐related conditions predisposing individuals to the disorder have been more difficult to elucidate. Epidemiological studies have attempted to identify risk factors and comorbidities associated with ALS, although the inability of such observational research to fully mitigate confounding effects or exclude reverse causality has made it challenging to find replicable causes of the disease.^5^

Genome‐wide association studies (GWAS) have revolutionized human genetics and have led to the discovery of thousands of risk variants involved in disease etiology.^6^ From the perspective of ALS research, summary statistics from hundreds of these studies have been published online in an effort to facilitate the application of current generation genomic techniques, such as linkage disequilibrium (LD) score regression testing and Mendelian randomization. Both methodologies are powerful tools to assess causality and investigate the extent to which genetic etiologies are shared across different diseases.

LD score regression and Mendelian randomization test distinct aspects of the genetic architecture underlying a disease. More specifically, LD score regression investigates whether polygenic risk contributing to a phenotype of interest might also contribute to the risk of ALS. This approach relies on the identification of shared genome‐wide heritability to pinpoint overlapping polygenic genetic variation between traits (pleiotropic relationship).^7^ On the other hand, Mendelian randomization uses genetic data to assess whether an exposure exerts a causal effect on a particular outcome.^8^ In contrast to LD score regression, Mendelian randomization usually focuses on genome‐wide significant single‐nucleotide polymorphisms (SNPs) for the exposure of interest (causal relationship).^8^ Because this analytical technique relies solely on genetic elements that remain constant over the life span of an individual, and that are randomized during gametogenesis, it effectively excludes reverse causality and reduces confounding to allow more reliable identification of a causal association between exposure and outcome.

Recent ALS‐related Mendelian randomization studies have focused on a hypothesis driven by only a single trait.^9^, ^10^, ^11^ Here, we implemented LD score regression and Mendelian randomization in a large‐scale audit relevant to ALS. In brief, our goal was to survey curated libraries of GWAS results using LD score regression and Mendelian randomization. The former is more liberal in identifying shared variation that suggests a significant degree of shared genetic risk, whereas the latter is more conservative and attempts to pinpoint causal associations by established loci. We also created an online resource (https://lng-nia.shinyapps.io/mrshiny) that can be used by the ALS community to inform pleiotropy or causality when undertaking observational studies or pursuing disease‐modifying interventions.

## Materials and Methods

### *Outcome Data*

Summary statistics from our recently published GWAS of ALS involving 20,806 cases and 59,804 controls of European ancestry were used as the outcome for both LD score regression and Mendelian randomization analyses. This study included 10,031,630 genotyped and imputed variants. Sample recruitment and genotyping quality‐control procedures are described elsewhere.^4^

### *LD Score Regression*

LD patterns across the genome enable the calculation of genetic correlations between traits. This is because the observed association for an SNP is a product of both its own contribution toward a phenotype and the association of the SNPs that are in LD with it. SNPs in regions of high LD tag a greater proportion of the genome and will show stronger associations than SNPs in regions of low LD. Using the known LD structure of a reference SNP panel, the heritability of a single phenotype or the genetic correlation of two phenotypes can be computed using LD score regression.^7^, ^12^

To study shared genetic risk by LD score regression, we used *LD Hub*, a centralized database of summary‐level GWAS results across multiple diseases/traits gathered from publicly available resources.^13^ LD score regression was implemented by regression of the chi‐squared statistics for the genetic associations with the trait against the LD scores for genetic variants across the whole genome. Unlike Mendelian randomization, LD score regression does not assess casualty, but rather only assesses multidirectional correlations, and can distinguish between population stratification and polygenicity in GWAS studies. Default settings were used in our analyses.

### *Mendelian Randomization*

Mendelian randomization is a proxy‐based approach for exploring whether an exposure is causally associated with an outcome. This is done by: identifying the SNPs associated with a particular exposure (eg, SNPs identified in a GWAS as being associated with colon cancer); extracting data for those SNPs from the outcome (in this case, a large‐scale GWAS of ALS^4^); harmonizing the exposure and outcome summary data; and applying Mendelian randomization methods to test for a causal relationship between the exposure and the outcome rooted in genetic associations.

Similar to *LD‐Hub*, the *MR Base* database is a curated database containing summary results from 1,094 GWASes involving 889 traits.^14^ These traits encompass a wide range of physiological characteristics and disease phenotypes. Each trait was tested separately as an exposure to determine if it alters risk of developing ALS. The analyses were performed using the R package *TwoSampleMR* (version 3.2.2; R Foundation for Statistical Computing, Vienna, Austria). The instrumental variables used for each exposure/phenotype consisted of the per‐allele log‐odds ratio (ie, beta estimate) and standard errors for all independent loci (ie, SNPs) reaching genome‐wide significance in the tested GWAS. Of the 1,094 GWASes with data available in *MR Base* (accessed August 15, 2018), 635 GWASes (consisting of 345 published GWASes and 290 unpublished GWASes performed on the UK Biobank; http://www.ukbiobank.ac.uk/) were included in our analysis based on the following criteria: (1) GWAS with at least two associated SNPs with *p* values <5.0 × 10^–8^, considering this *p* value to be the generally accepted genome‐wide significant threshold; (2) SNPs present in both the exposure and outcome (ALS) data sets or when not present their LD proxies (R^2^ value > = 0.8); and (3) independent SNPs (R^2^ <0.001 with any other associated SNP within 10 Mb), considered as the most stringent clumping threshold used when performing Mendelian randomization analyses.

**Figure 1:**
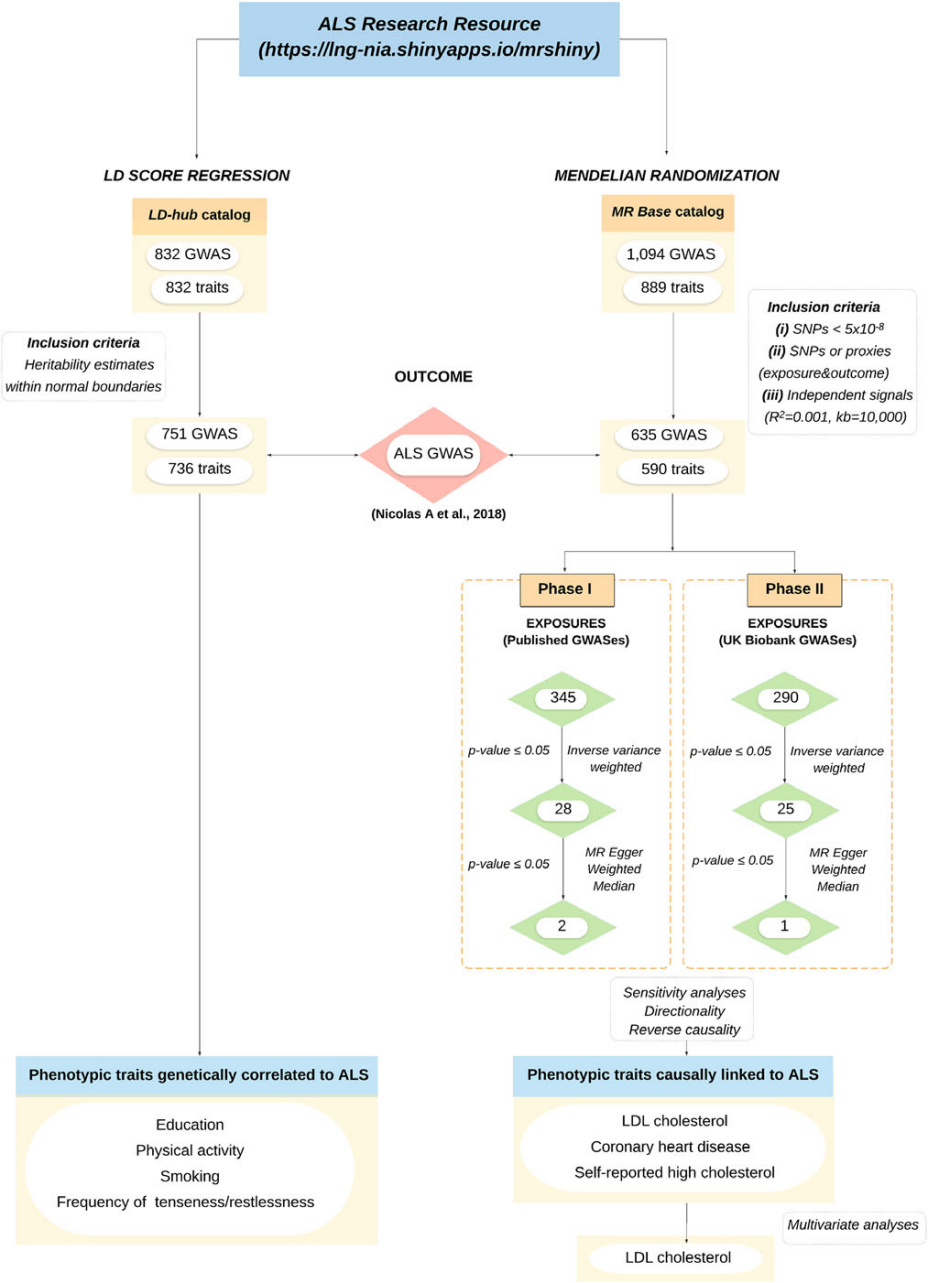
**Flow chart of analysis.** The ALS Research Resource is an interactive tool where the user can explore genetic correlations and causal associations across more than 700 traits. GWAS exposures used for LD score regressions are available in LD hub at http://ldsc.broadinstitute.org/ldhub/. GWAS exposures used for the Mendelian randomization analyses are available in MR Base at http://www.mrbase.org/. (A) The inclusion criteria used for LD score regression analyses comprise traits with heritability estimates within normal boundaries. (B) The inclusion criteria used for Mendelian randomization includes (1) GWAS with at least two associated SNPs with *p* values <5.0 × 10^–8^; (2) SNPs present in both the exposure and outcome (ALS) data sets or when not present their linkage‐disequilibrium (LD) proxies (R^2^ value > = 0.8); and (3) independent SNPs (R^2^ < 0.001 with any other associated SNP within 10 Mb), considered as the most stringent clumping threshold used when performing MR analyses. (C) LD score regression analyses included 751 publicly available GWASes considered as exposures of interest versus the most recent ALS GWAS as an outcome, and (D) MR analyses were performed considering two phases. Phase I includes 345 available GWASes in the public domain as exposures of interest while phase II includes unpublished UK Biobank GWAS data. (E) Significantly associated GWASes with ALS at inverse variance weighted (*p* < 0.05). (F) Significantly associated GWASes with ALS at weighted median and MR Egger (*p* < 0.05). (G) Causally linked GWASes with ALS after performing reverse causality, sensitivity, and directionality analyses. (H) Multivariate analyses used to explore how each related exposure of interest independently contributes to ALS. ALS = amyotrophic lateral sclerosis; GWAS = genome‐wide association study; kb = kilobases; R^2^ = clumping threshold; LDL = low‐density lipoprotein.

Harmonization was undertaken to rule out strand mismatches and ensure alignment of SNP effect sizes. Within each exposure GWAS, Wald ratios were calculated for each extracted SNP by dividing the per‐allele log‐odds ratio of that variant in the ALS data by the log‐odds ratio of the same variant in the exposure data. We then applied a two‐step approach designed to decrease the risk of false‐positive associations (see Fig 1 for the workflow).

First, the inverse‐variance weighted method was implemented to examine the relationship between the exposure and ALS. In this method, the Wald ratio for each SNP is weighted according to the inverse variance, and a line, constrained to pass through the origin, is fitted to the data. The slope of the line represents the pooled‐effect estimate of the Wald ratios.^15^ Traits were brought forward to the next stage of analysis only if the *p* value of the pooled‐effect estimate was ≤0.05. Next, two Mendelian randomization sensitivity tests (ie, MR Egger and weighted median) were applied to those traits/GWASes passing the first phase of analysis. These sensitivity analyses evaluated core assumptions of Mendelian randomization, and traits were considered to be consistent with a causal effect when *p* values were ≤0.05. Heterogeneity of effects were tested using the Cochran's Q test, quantified using the I^2^ statistic, and displayed in forest plots. Steiger analyses were performed to verify that the proposed instruments were directly associated with the outcome^16^ or effect estimate directionality.

We evaluated the possibility that the overall estimate was driven by a single SNP using leave‐one‐out analyses for each of the GWASes associated with ALS. We further explored the possibility of reverse causality by using SNPs tagging the five independent loci described in the ALS GWAS as exposure instrument variables and the identified GWASes as the outcome. Lasso‐based multivariate analysis was used to explore how each related exposure of interest (ie, low‐density lipoprotein [LDL] cholesterol, self‐reported cholesterol, and coronary heart disease) independently contribute to ALS.

### *Genetic Risk Score*

To further test the relationship between LDL cholesterol and ALS, a cumulative genetic risk score for LDL cholesterol was calculated in a smaller subset of the samples for which individual genotype data were available, including 8,229 ALS cases and 36,329 controls.^4^ Instrumental variables of interest were incorporated and weighted by beta values in the ALS GWAS. Next, a logistic regression was performed on this subset of cases and controls, regressing disease against quintile membership based on genetic risk score.^17^ Odds ratios were reported comparing the lowest risk quintile (reference group) to the remaining quintiles. Genetic risk scores were also calculated for different subtypes of ALS patients (carriers of the pathogenic *C9orf72* repeat expansion, familial cases, sporadic cases, male cases, and female cases). Risk profiling was adjusted for sex, age, and 20 principal components to account for population stratification.

### *Colocalization Analysis*

Bayesian colocalization analysis was used as a statistical method to identify putative candidate genetic variants involved in LDL cholesterol blood levels that contribute most to the risk of developing ALS.^18^ For these analyses, we considered the 78 SNPs that were significantly associated with increased LDL cholesterol and were used as relevant instrumental variables for Mendelian randomization analyses. We extracted summary statistics for those variants (as well as variants 1 megabase [Mb] upstream and downstream) from the LDL cholesterol GWAS and from the ALS GWAS. Bayesian colocalization was then run for each independent region as implemented in the R package coloc (https://cran.r-project.org/package=coloc). This analysis assessed the probability of each SNP being responsible for the change in ALS risk through variation in LDL cholesterol. We derived posterior probabilities (PPH_0‐4_) for each region and considered PPH_4_ > 0.95 as strong evidence for colocalization under the assumption of a single causative variant per locus.

## Results

### *Large‐Scale LD Score Regression Analysis in ALS*

LD score regression was applied to examine the genetic correlation between our recently published GWAS meta‐analysis of ALS^4^ and 736 phenotypes available in *LD‐hub*, a centralized database of GWAS results across multiple diseases and traits (http://ldsc.broadinstitute.org/ldhub/; Fig 1).

### *Traits Genetically Correlated to ALS by LD Score Regression*

Our analyses identified 18 traits that were genetically correlated to ALS after adjusting for multiple testing by false discovery rate (Table 1). Among these, nine traits were related to educational attainment and intelligence, indicating that higher levels of education were associated with a decreased risk of ALS (smallest adjusted *p* value = 1.78 × 10^–4^; regression coefficient = –0.338; 95% confidence interval [CI] = –0.46, –0.20).

**Table 1:** Linkage Disequilibrium Score Regression Results for Traits Genetically Correlated With ALS

	Trait	Source	rg [se]	*p*	FDR *p*	h^2^ [se]
Education	Fluid intelligence score	UKBB	–0.338 [0.067]	4.74E‐07	1.78E‐04	0.238 [0.011]
	Qualifications: Other professional qualifications eg: nursing_ teaching	UKBB	–0.257 [0.071]	3.00E‐04	1.73E‐02	0.047 [0.003]
	Qualifications: A levels/AS levels or equivalent	UKBB	–0.255 [0.059]	1.61E‐05	1.66E‐03	0.097 [0.004]
	Qualifications: college or university degree	UKBB	–0.249 [0.053]	2.77E‐06	5.19E‐04	0.168 [0.005]
	Qualifications: O levels/GCSEs or equivalent	UKBB	–0.238 [0.069]	5.00E‐04	2.68E‐02	0.049 [0.003]
	Age completed full‐time education	UKBB	–0.229 [0.068]	7.00E‐04	3.09E‐02	0.084 [0.005]
	Years of schooling 2016	27225129	–0.226 [0.059]	1.00E‐04	6.83E‐03	0.127 [0.004]
	No. of incorrect matches in round	UKBB	0.229 [0.06]	1.00E‐04	6.83E‐03	0.055 [0.003]
	Qualifications: none of the above	UKBB	0.255 [0.059]	1.49E‐05	1.66E‐03	0.098 [0.004]
Activity	Types of transport used (excluding work): walk	UKBB	–0.403 [0.085]	2.11E‐06	5.19E‐04	0.033 [0.002]
	Types of transport used ( excluding work): public transport	UKBB	–0.402 [0.092]	1.13E‐05	1.66E‐03	0.022 [0.002]
	Types of physical activity in last 4 weeks: light DIY	UKBB	–0.287 [0.07]	4.24E‐05	3.54E‐03	0.039 [0.002]
	Types of physical activity in last 4 weeks: walking for pleasure	UKBB	–0.286 [0.079]	3.00E‐04	1.73E‐02	0.037 [0.002]
	Duration of moderate activity	UKBB	0.283 [0.084]	7.00E‐04	3.09E‐02	0.032 [0.002]
	Job involves mainly walking or standing	UKBB	0.216 [0.065]	9.00E‐04	3.56E‐02	0.08 [0.004]
Smoking	Exposure to tobacco smoke at home	UKBB	0.42 [0.122]	6.00E‐04	3.00E‐02	0.012 [0.002]
	Light smokers (at least 100 smokes in lifetime)	UKBB	0.427 [0.1]	1.77E‐05	1.66E‐03	0.077 [0.008]
Other	Frequency of tenseness/restlessness in last 2 weeks	UKBB	0.227 [0.068]	9.00E‐04	3.56E‐02	0.044 [0.003]

See supplementary materials for a description of the phenotypes included in the UK Biobank data set and Figure 1 for the number of traits screened as part of the LD score regression analysis. Source: number denotes PubMed identification numbers.

UKBB = UK Biobank; rg = regression; se = standard error; FDR = false discovery rate adjusted *p* value; h^2^ = observed narrow‐sense heritability.

Traits related to light physical activity, including walking for pleasure, walking as a mean of transport, and light DIY physical activities, were associated with decreased risk of developing ALS (smallest adjusted *p* value = 5.19 × 10^–4^; regression coefficient = –0.403; 95% CI = –0.35, –0.14), whereas heavier activity levels such as duration of moderate activity and performing a job that involves mainly walking or standing were positively associated to ALS (smallest adjusted *p* value = 3.09 × 10^–2^; regression coefficient = 0.28; 95% CI = –0.36, –0.09).

Smoking behavior, including exposure to tobacco and being a light smoker, showed genetic correlation with ALS (smallest adjusted *p* value = 1.66 × 10^–3^; regression coefficient = 0.42; 95% CI = 0.23, 0.62). Detailed results for the remaining 718 nonsignificant traits are shown in Table S1 and can be interactively searched at https://lng-nia.shinyapps.io/mrshiny.

### *Large‐Scale Mendelian Randomization in ALS*

Next, we performed Mendelian randomization to further investigate causal links between multiple phenotypic traits (exposures) and ALS (outcome, Fig 1). The exposures of interest consisted of 345 GWASes involving a wide range of physiological characteristics and disease phenotypes for which data were available in *MR Base* (http://www.mrbase.org/). The recently published GWAS of ALS involving 20,806 cases and 59,804 controls was used as the outcome.^4^

There are no previous reports in the literature where multiple phenotypes were tested using Mendelian randomization in an unbiased, hypothesis‐free manner. This raised concerns about false‐positive associations and multiple‐testing correction. To control for this and confirm the validity of our findings, we replicated in an independent collection of phenotypes (290 unpublished GWASes performed on the UK Biobank, http://www.ukbiobank.ac.uk/). Here, we only report associations that were significant across both the published and unpublished sets of GWASes. Detailed results for the 635 GWAS under study are shown in Tables S2 to S4 and can be visually explored at https://lng-nia.shinyapps.io/mrshiny.

### *Traits Causally Linked to ALS by Mendelian Randomization*

We identified the phenotypic traits LDL cholesterol and coronary heart disease in the published GWASes, and self‐reported high cholesterol in the UK Biobank, as being causally linked to ALS risk (see Table 2 and Table S5 for SNPs used to construct the instruments of interest. Multivariate analysis showed that the signals arising from the coronary heart disease and self‐reported high cholesterol were driven by SNPs related to LDL cholesterol, revealing that both traits represent closely related phenotypes (Table 3).

**Table 2:** Mendelian Randomization Results for Exposures Causally Linked to ALS

			Inverse Variance Weighted		MR Egger		Weighted Median	
Exposure	Source	No. of SNPs	OR [CI 95%]	*p*	OR [CI 95%]	*p*	OR [CI 95%]	*p*
LDL cholesterol id:300	24097068	78	1.116 [1.03–1.20]	0·003	1.115 [1.00–1.23]	0·054	1.108 [1.00–1.226]	0·046
Coronary heart disease id:7	26343387	37	1.063 [1.0–1.13]	0·047	1.175 [1.01–1.35]	0·032	1.116 [1.020–1.220]	0·015
Self‐reported: high cholesterol id:UKB‐a:108	UKBB	49	2.389 [1.48–3.84]	0·0003	2.669 [1.08–6.55]	0·038	2.110 [1.021–4.357]	0·044

id = specific code attributed to each trait by *MR Base*; No. of SNPs = number of SNPs; OR = odds ratio; CI = confidence interval; LDL = low‐densitity lipoprotein.

**Table 3:** Multivariable Analysis to Estimate the Simultaneous Effects of Two Exposures

a) Analysis comparing LDL cholesterol vs self‐reported cholesterol				
Exposure	No. of SNPs	beta	se	*p*
LDL cholesterol || id:300	72	0·175	0·084	0·019
Self‐reported high cholesterol || id:UKB‐a:108	34	–0·847	0·722	0·120

b) Analysis comparing LDL cholesterol vs coronary heart disease				
Exposure	No. of SNPs	beta	se	*p*
LDL cholesterol || id:300	74	0·087	0·040	0·014
Coronary heart disease || id:7	26	0·027	0·038	0·239

id = specific code attributed to each trait by *MR Base*; se = standard error; No. of SNPs = number of SNPs; LDL = low‐density lipoprotein.

Leave‐one‐out analysis indicated that no single SNP accounted for these associations in isolation (Table S6). Additional analyses examining directionality, pleiotropy, and reverse causality did not indicate any violation of core Mendelian randomization assumptions for these traits (Tables S7–S8). We used genetic risk profiling to estimate the extent to which risk of developing ALS is attributable to LDL cholesterol. We found that individuals with the highest burden of genetic risk were 1.075 times more likely to develop ALS (95% CI, 1.001–1.150; *p* value = 0.003). The increase in ALS risk associated with LDL cholesterol levels was similar across different subtypes of ALS (*C9orf72* carriers, familial ALS, sporadic ALS, male, and female‐only cases; Table S9).

### *Identification of Functional Causal Variants*

**Figure 2:**
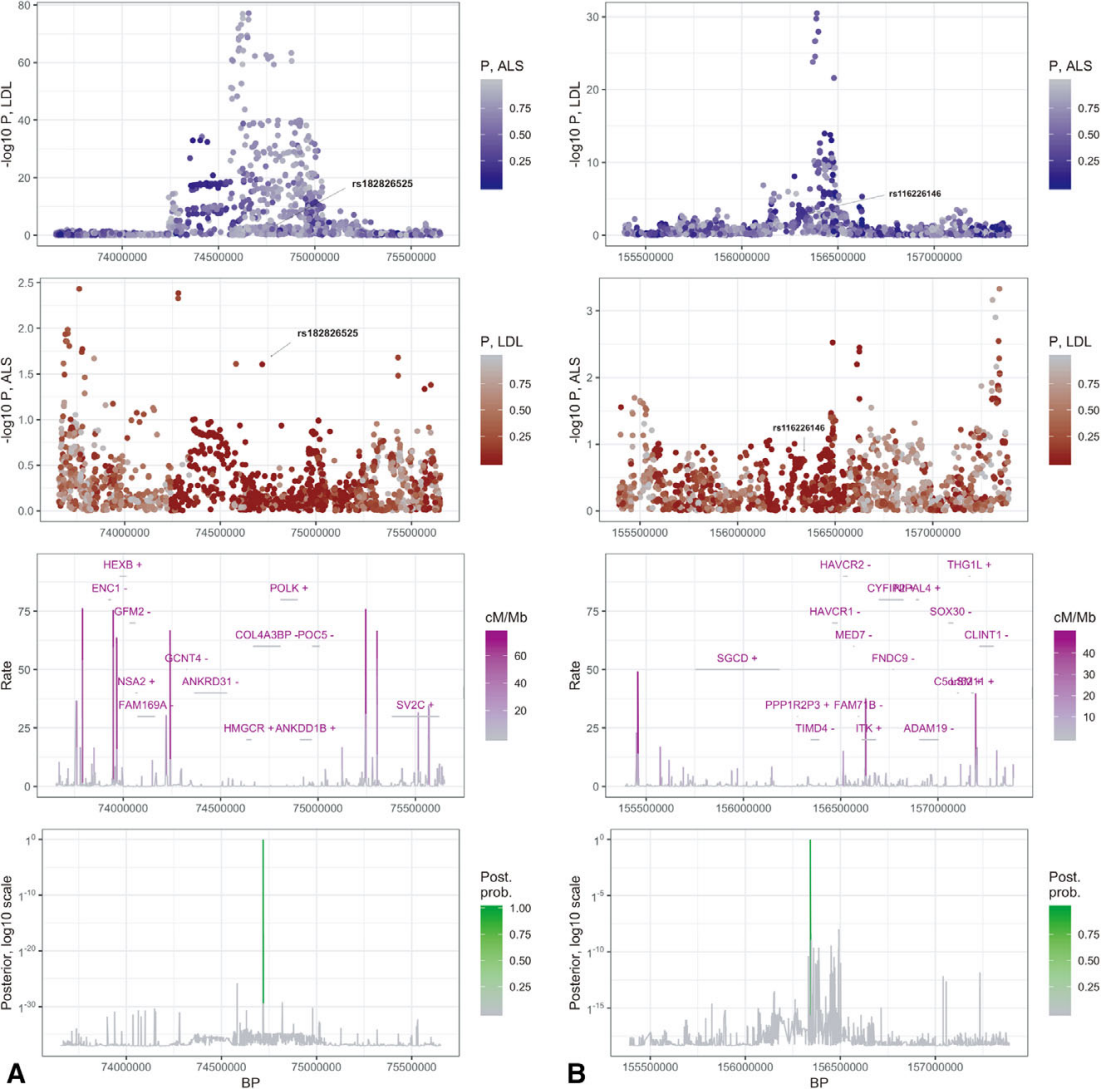
**Bayesian colocalization plots.** A plot and B plot represent two independent LDL‐cholesterol–associated regions with posterior probability greater than 95% of sharing a causal variant involved in ALS. Panels in column A show the region spanning chr5:73656720‐75651786 where rs182826525 is likely the shared causal variant with a posterior probability of nearly 100%. Panels in column B show the region spanning chr5:155390511‐157388284 where rs116226146 is likely the shared causal variant with a posterior probability of 96%. The first row displays the *p* values from the LDL GWAS for each region. Color is coded by *p* values in the ALS GWAS. The second row displays the *p* values from the ALS GWAS for the same regions. Color is coded by *p* values in the LDL GWAS. The third row shows local gene positions (with strands denoted by ±), as well as recombination rates measured in cM/Mb.^38^ The bottom row shows the posterior probabilities of a shared causal variant between LDL cholesterol and ALS. ALS = amyotrophic lateral sclerosis; GWAS = genome‐wide association study; kb = kilobases; LDL = low‐density lipoprotein.

Bayesian colocalization analysis was performed to putatively identify the functional candidate variants that may drive risk of developing ALS through shared pathway effects of LDL cholesterol levels. We focused our efforts on the 78 independent regions associated with LDL cholesterol in the exposure GWAS. This analysis identified two independent regions with >95% probability of containing a shared causal SNP (Table S10). Fine mapping of these regions identified two SNPs (rs182826525 within *COL4A3BP* and rs116226146 intergenic between *PPP1R2P3* and *TIMD4*) as being causally linked to ALS through an increase of LDL cholesterol levels (Fig 2).

## Discussion

We applied cutting‐edge analytical techniques to genomic data across a wide range of phenotypic traits to identify factors associated with risk of developing ALS. This hypothesis‐free, data‐driven approach provided prima facie evidence supporting the existence of multiple such factors. Using genetic data to comprehensively map the risk factor landscape of ALS represents a novel approach in neurological disease. We used these results from nearly 25 million individuals (24,538,000 from published GWAS and ∼337,159 from UKBB studies) to establish a public resource that can be accessed by other researchers to explore risk factors and shared disease mechanisms in ALS.

LD score regression analyses found that common genetic variation associated with higher cognitive performance is negatively correlated to ALS. At a molecular level, these findings indicate that the genetic factors driving mental ability and ALS overlap to some extent. This may have been an expected outcome given the well‐known relationship between ALS and frontotemporal dementia, and our findings are consistent with previous epidemiological reports assessing the causal relationship between education and ALS.^19^ Nevertheless, the large number of samples analyzed in our study and its grounding in genetics put this risk factor on a firmer footing within the ALS field. Similar education effects have been observed in Alzheimer's disease,^20^ but understanding how education protects against neurodegeneration or which genetic variants are responsible for this shared risk will require additional study. One intriguing possibility is that the genetic variants responsible for ALS in middle age or in the elderly are also associated with decreased cognitive performance at a younger age. This is plausibly consistent with the observation that connectivity and gray matter volumes are altered in asymptomatic carriers of the *C9orf72* repeat expansion.^21^, ^22^

Epidemiological case‐control studies have extensively reported a relationship between exercise and risk of developing ALS,^23^, ^24^, ^25^ though there are conflicting results as to the level of physical activity required to increase risk.^19^ Our genetic‐based data demonstrate that this neuromuscular interconnection may be more complex than previously appreciated: Light physical activity, including walking for pleasure or light DYI activities, was negatively associated with developing ALS, whereas more strenuous activity, such as duration of moderate activity, was paradoxically correlated with ALS. Extrapolating from these observations to neuromuscular physiology, relatively low levels of exercise may exert a neuroprotective effect by preventing muscle atrophy that, in turn, supports motor neuron integrity though the neuroadaptive generation of neurotrophic factors.^26^ In contrast, excessive physical activity may be detrimental to motor neurons because of excessive free radical production and/or glutamate excitotoxicity that overwhelms neuroprotective mechanisms.^27^, ^28^ Regardless, our data do not provide any insight into the effect of exercise on survival once the patient has presented with symptoms.

There is compelling epidemiological evidence showing that cigarette smoking is a key environmental risk factor for ALS.^29^ Our LD data not only confirm that being a smoker is positively correlated to developing ALS, but also show that this effect is mediated, at least in part, through shared genetic mechanisms. This is an example of the ability of this type of genomic analysis to identify pleiotropic effects, which is where a defect in a single gene can give rise to multiple, apparently unrelated phenotypes. Here, we are extending the concept of pleiotropy beyond the single‐gene paradigm to encompass inherently complex traits driven by multiple genetic variants spread across the genome and that are typically outside of coding regions. LD score regression is not designed to identify the specific shared genetic variants responsible for both phenotypic traits, but instead focuses on establishing whether such pleiotropy exists between traits.

Using Mendelian randomization, we found strong evidence that an alteration of lipid metabolism is causally linked to ALS. We undertook sensitivity analyses to reduce the possibility of bias in our results and replicated our findings across three different exposure GWASes, including a large, independent cohort obtained from the UK Biobank. Furthermore, we demonstrated that the increased risk of ALS attributed to coronary heart disease is driven by LDL cholesterol. Though hyperlipidemia only modestly increases the risk of ALS, this effect likely operates over the lifetime of the individual, and the cumulative effect on disease risk may be substantial.

Previous epidemiological studies have explored the role of blood lipids in the pathogenesis of ALS. These observational studies have yielded controversial results, with many reporting that hyperlipidemia increases disease risk and others suggesting the opposite.^30^, ^31^, ^32^, ^33^, ^34^, ^35^, ^36^ In addition to being underpowered, much of this previous research was based on blood‐lipid profiles obtained after diagnosis of ALS when ancillary factors may be influencing these levels.^30^, ^31^, ^32^, ^33^, ^34^, ^35^, ^36^

The singular advantage of Mendelian randomization is that it is agnostic to these confounders and can be considered nature's randomized controlled trial. Based on genetic data that remain constant between the presymptomatic and symptomatic phases of the disease, it accurately pinpoints predisposing factors for the disease of interest. While the manuscript for this article was under review, a link between blood lipids and the risk of ALS has been recently reported in European and East Asian populations using polygenic risk scores and Mendelian randomization.^9^, ^10^ These studies were performed involving a smaller cohort of ALS cases and were based on the a priori hypothesis that blood lipids were involved in the pathogenesis of ALS. Our work extends these recent reports by definitely applying Mendelian randomization across a large number of phenotypic traits in an unbiased fashion, replicating our findings in an independent cohort (UK Biobank), and delineating the specific aspects of lipid metabolism relevant to the pathogenesis of ALS.

Circulating blood cholesterol are multifunctional molecules, involved primarily in energy generation, as precursors or cofactors for signaling molecules, and in neuronal development and function.^37^ Dysregulation of cholesterol homeostasis in the brain has been linked to many neurodegenerative diseases such as Huntington's disease, Parkinson's disease, Niemann‐Pick disease type C, and, most notably, Alzheimer's disease.^38^ The generation and clearance of β‐amyloid protein is regulated by cholesterol, and drugs that inhibit cholesterol synthesis lower this protein within neurons,^39^ as is the more recent finding that the two secretory forms (APPɑ and APP β) of amyloid precursor protein (APP) have opposing associations with β‐amyloid generation, cholesterol biosynthesis, and LDL receptor levels.^40^ The identification of the cholesterol transport protein, apolipoprotein E, as a major genetic risk factor for Alzheimer's disease is also consistent with a role for cholesterol in the pathogenesis of neurodegenerative disease.^41^, ^42^ Despite this, the molecular mechanisms by which altered lipid metabolism leads to neuron degeneration are unclear.

An important question arising from our analysis centers on why LDL may causally affect ALS, while at the same time LDL levels are not genetically correlated with ALS under the LD Score regression model. This apparent divergence is because the variants linked to these two traits are not pleiotropic, and again highlights the fact that Mendelian randomization and LD regression analysis investigate different aspects of the genetic architecture underlying diseases. Mendelian randomization allows us to compare two groups of people that differ by the genetic variants of interest and therefore by any modifiable factor to which those genetic variants relate. In this case, genetic variants that are associated with LDL metabolism affect LDL levels, and a ratio measure is calculated to determine how much this estimated change in LDL level would predispose individuals to ALS. If substantial pleiotropy were present, we would find that the same genetic variants that affect LDL metabolism also increase the risk of ALS by themselves (ie, genetic correlation). Such pleiotropy was not observed in our data. Instead, we found that the only mechanism by which ALS risk could be increased is through an increase of LDL cholesterol levels (ie, linear association).

Our data led us to propose that lowering blood‐cholesterol levels is a viable strategy for reducing risk associated with ALS. A similar approach may be effective in Alzheimer's disease where exposure to statins is associated with substantially reduced risk of dementia in observational studies.^43^, ^44^ Though the American Heart Association guidelines for treating blood cholesterol to reduce cardiovascular risk are widely implemented in the community, they primarily focus on patients aged >50 years.^45^ An alternative strategy may be to identify a younger subpopulation at increased risk of developing ALS and institute treatment with lipid‐lowering agents. This approach would initially focus on individuals with a family history of ALS or frontotemporal dementia, and on presymptomatic cases carrying the *C9orf72* repeat expansion; together, these subtypes account for nearly 1 in 5 cases of ALS.^46^ Long‐term monitoring would be required to detect side effects from the medication and determine effect on age of disease onset.

We conclude by saying that the reported findings should be interpreted in the context of existing evidence from other research studies using different designs, and definite conclusions should not be elaborated uniquely based on Mendelian randomization results. Future randomized controlled trials should be considered as a proof of causality.

### *Limitations to This Study*

Our analyses were limited to only those GWAS studies present in two public databases, namely *LD‐hub* and *MR Base*. Furthermore, the available data are focused on European populations. We envisage that future studies may expand our findings by utilizing larger sample sizes, greater density across the genome, and importantly non‐European populations, highlighting the utility of an ALS resource that is constantly updated as new data become available.

One of the main caveats of working with summary level data (rather than individual‐level data) is that there is no possibility to filter and exclude sample overlap. For Mendelian randomization analyses, we cannot exclude the possibility that samples from the same individuals were used in both the GWASes that we identified as significant exposures and in the ALS GWAS that we used as the outcome measure. Such sample overlap may bias estimates in MR and increase type 1 error rates. We reviewed the origin of the European cohorts present in our ALS outcome and in the significantly associated exposures, and the results of this comparison are outlined in Table S11. Our data suggest that sample overlap had only a minimal effect on our results. We also performed sensitivity analyses by calculating the F‐statistic parameter as described elsewhere.^47^ Our results showed that two of the three GWASes of interest for which the F‐statistic could be calculated were considered strong instruments and are unlikely to be susceptible to bias because of overlapping samples (F‐statistic for LDL cholesterol = 59.02; F‐statistic for coronary heart disease = 742.2). Furthermore, sample overlap alone cannot account for our findings, given that any sample overlap would be equally likely to occur across the diverse GWASes that we studied, and yet we consistently identified altered lipid metabolism as a risk factor for ALS across multiple GWAS studies and across multiple populations. There is no realistic scenario in which sample overlap could have been consistently confined to just GWASes involving lipid metabolism. However, given that Mendelian randomization effect estimates are often small, mandating additional follow‐up on connected pathways.

A concern that might arise is to what extent hereditary cases of ALS carrying rare genetic variants might have influenced our analyses. One should expect that carrying large effect, rarer variants would not generally preclude the carrying of more common, small effect genetic risk factors which comprise the majority of GWAS results that were used for Mendelian randomization and LD Score regressions.

Finally, we are aware that certain bias could exists because of undetectable issues in underlying GWAS results utilized in this survey, but the fact that we have replicated our results in independent GWASes alleviates this concern.

## Author Contributions

S.B.C., A.J.N., M.A.N., and B.J.T. contributed to the conception and design of the study. A.C., G.M., P.J.T., Ad.C., and B.J.T., S.B.C., A.J.N., G.B., and M.N. contributed to the acquisition and analysis of data. S.B.C., A.J.N., M.A.N., A.N., A.C., G.M., P.J.T., D.J.S., A.B.S., and Ad.C. contributed to drafting a significant portion of the manuscript or figures. We thank the ITALSGEN Consortium, and the International ALS Genetics Consortium for their contributions (see Supplementary Information for additional details).

## Potential Conflicts of Interest

B.J.T., P.J.T., and A.B.S. hold patents on the clinical testing and therapeutic intervention for the hexanucleotide repeat expansion of *C9orf72*. All other authors declare that they have no conflicts of interest.

## Supporting information


**Appendix S1** Supporting information
