# Supplementary material for: Shared polygenic risk and causal inferences in amyotrophic lateral sclerosis
Source: Ann Neurol. 2019 Mar 13;85(4):470–81. doi: 10.1002/ana.25431 (PMC6450729; doi:10.1002/ana.25431)
Supplement: Supplementary file 1 — Appendix S1 Supporting information [file ANA-85-470-s001.pdf]

Supplementary material

Shared polygenic risk and causal inferences in amyotrophic lateral sclerosis

Sara Bandres-Ciga, PhD[1]*, Alastair J. Noyce, MD, PhD[2,3]*, Gibran Hemani, PhD[4], Aude Nicolas, PhD[5], Andrea Calvo, MD[6], Gabriele Mora, MD[7]; The ITALSGEN Consortium; The International ALS Genomics Consortium; Pentti J. Tienari, MD[8], David J. Stone, PhD[9], Mike A. Nalls, PhD[1,10], Andrew B. Singleton, PhD[1], Adriano Chiò, MD[6,11,12,*], and Bryan J. Traynor, MD, PhD[5,13,*]

* Equal contribution

Table S1. Linkage disequilibrium score regression analyses performed on 736 GWASes…………………………………………………… 1

Table S2. Mendelian randomization analysis performed on 635 GWASes across 570 phenotypic traits;
(clumping threshold of R2 = 0,001, kb = 10,000)…… 19

Table S3. Horizontal pleiotropy analysis performed on 635 GWASes across 570 phenotypic traits………………………………… 58

Table S4. Heterogeneity analysis performed on 635 GWASes across 570 phenotypic traits…………………………………………… 73

Table S5. SNPs used to construct the three instruments causally linked to ALS………………………………………………………… 114

Table S6. Leave-one-out analysis of the three exposures causally linked to ALS………………………………………………………. 118

Table S7. Horizontal pleiotropy, heterogeneity and directionality analyses of the three exposures
causally linked to ALS………123

Table S8. Reverse causality analysis……. 124

Table S9. Genetic risk profiling of LDL cholesterol in ALS subtypes………………………………………………………………………………. 125

Table S10. Bayesian colocalization analysis of the 78 independent regions associated to LDL cholesterol and ALS……….. 126

Table S11. Summary of the cohorts included in the exposures of interest and the outcome……………………………………… 129

Description of the significant phenotypes genetically correlated with ALS and included in the UK Biobank dataset………. 131

Consortia members……… 133

Table S1. Linkage disequilibrium score regression analyses performed on 736 GWASes

Trait	Source	rg	se	z	p-value	FDR p-value	h2 obs	h2 obs se	h2 int	h2 int se	gcov int	gcov int se
18:2 linoleic acid (LA)	27005778	0.168	0.208	0.808	0.419	0.909	0.125	0.047	1.0038	0.0097	-5.00E-04	0.0054
22:6 docosahexaenoic acid	27005778	-0.161	0.161	-1.001	0.317	0.813	0.132	0.037	0.9955	0.0072	0.0058	0.0045
2hr glucose adjusted for BMI	20081857	0.133	0.173	0.770	0.442	0.918	0.103	0.035	0.9933	0.0072	-0.0012	0.0045
Acetate	27005778	0.057	0.188	0.302	0.763	0.985	0.056	0.019	1.0015	0.0069	-0.002	0.0045
Acetoacetate	27005778	0.099	0.192	0.516	0.606	0.980	0.075	0.028	0.9795	0.0071	0.0017	0.0047
Adiponectin	22479202	-0.021	0.133	-0.161	0.872	0.986	0.120	0.025	1.0221	0.0121	-0.0078	0.0054
Adopted as a child	0	0.319	0.175	1.824	0.068	0.505	0.006	0.001	1.001	0.0066	-0.0025	0.005
Age at first live birth	0	-0.223	0.071	-3.142	0.002	0.058	0.166	0.008	1.0469	0.009	-0.0015	0.0058
Age at last live birth	0	-0.166	0.087	-1.914	0.056	0.458	0.088	0.006	1.0168	0.0082	-0.0012	0.0054
Age at Menarche	25231870	0.096	0.062	1.560	0.119	0.602	0.202	0.010	0.9539	0.0116	-0.009	0.0053
Age at Menopause	26414677	0.003	0.080	0.038	0.970	0.995	0.136	0.016	0.9898	0.0137	-0.0044	0.0049
Age completed full time education	0	-0.229	0.068	-3.375	0.001	0.031	0.084	0.005	1.0631	0.0094	-0.0093	0.0052
Age of first birth	27798627	-0.237	0.075	-3.168	0.002	0.054	0.063	0.004	0.9524	0.0079	-0.0026	0.0049
Age of smoking initiation	20418890	0.412	0.200	2.064	0.039	0.433	0.063	0.018	0.9989	0.0062	-0.0154	0.0046
Age started oral contraceptive pill	0	-0.224	0.094	-2.390	0.017	0.268	0.049	0.004	1.0152	0.0074	0.0028	0.0052
Age when periods started (menarche)	0	0.085	0.057	1.496	0.135	0.611	0.209	0.011	1.004	0.0123	-0.0024	0.0064
Alanine	27005778	-0.013	0.152	-0.086	0.931	0.986	0.085	0.027	1.0118	0.0073	0.0024	0.0046
Albumin	27005778	0.026	0.215	0.121	0.904	0.986	0.058	0.024	0.9905	0.0067	0.0034	0.0045
Alcohol drinker status: Never	0	-0.215	0.104	-2.062	0.039	0.433	0.014	0.002	1.0161	0.0066	0.0033	0.0048
Alcohol drinker status: Previous	0	0.036	0.119	0.305	0.761	0.985	0.013	0.002	1.0067	0.0067	0.0056	0.005
Alcohol intake frequency.	0	-0.017	0.056	-0.299	0.765	0.985	0.084	0.004	1.0448	0.0104	0.0066	0.0056
Alcohol intake versus 10 years previously	0	0.160	0.084	1.910	0.056	0.458	0.035	0.002	1.0299	0.0081	0.0056	0.005
Alcohol usually taken with meals	0	-0.171	0.068	-2.520	0.012	0.225	0.110	0.005	1.0594	0.0091	-0.0041	0.0053
Alzheimers disease	24162737	0.153	0.150	1.018	0.309	0.811	0.045	0.021	1.0659	0.029	0.0272	0.0052
Amyotrophic lateral sclerosis	27455348	0.741	0.075	9.867	0.000	0.000	0.049	0.013	0.9944	0.0063	0.7923	0.0063
Anorexia Nervosa	24514567	0.042	0.079	0.530	0.596	0.980	0.559	0.031	0.8976	0.0071	0.031	0.0049
Apolipoprotein A-I	27005778	0.055	0.172	0.321	0.748	0.985	0.073	0.031	1.0053	0.0117	0.0018	0.0043
Apolipoprotein B	27005778	-0.014	0.166	-0.084	0.933	0.986	0.084	0.039	1.0084	0.0145	-4.00E-04	0.0044
Arm fat mass (left)	0	0.068	0.052	1.314	0.189	0.698	0.232	0.008	1.0675	0.0173	-0.0066	0.0075
Arm fat mass (right)	0	0.070	0.052	1.344	0.179	0.689	0.231	0.008	1.0706	0.0174	-0.0071	0.0076
Arm fat percentage (left)	0	0.063	0.051	1.223	0.221	0.740	0.219	0.007	1.0739	0.0177	-0.0058	0.0074
Arm fat percentage (right)	0	0.066	0.052	1.276	0.202	0.709	0.216	0.007	1.0786	0.0174	-0.0058	0.0072
Arm fat-free mass (left)	0	0.048	0.047	1.030	0.303	0.802	0.265	0.011	1.1098	0.0258	-0.0069	0.0073
Arm fat-free mass (right)	0	0.033	0.046	0.710	0.478	0.923	0.266	0.011	1.1035	0.027	-0.0037	0.0072
Arm predicted mass (left)	0	0.045	0.047	0.968	0.333	0.831	0.264	0.011	1.1119	0.0262	-0.0063	0.0073
Arm predicted mass (right)	0	0.034	0.047	0.739	0.460	0.918	0.265	0.011	1.1055	0.0271	-0.0045	0.0072
Asthma	17611496	0.110	0.152	0.726	0.468	0.918	0.117	0.026	1.011	0.0083	0.0249	0.0053
Attention deficit hyperactivity disorder	20732625	0.034	0.199	0.171	0.864	0.986	0.254	0.091	1.0051	0.0061	-0.0011	0.0045
Attention deficit hyperactivity disorder (GC)	27663945	0.316	0.207	1.526	0.127	0.602	0.073	0.030	0.9928	0.0074	7.00E-04	0.0049

Attention deficit hyperactivity disorder (No GC)	27663945	0.316	0.207	1.527	0.127	0.602	0.074	0.030	1.0074	0.0074	7.00E-04	0.0049
Autism spectrum disorder	0	-0.191	0.109	-1.756	0.079	0.505	0.460	0.052	0.9645	0.0066	-4.00E-04	0.0047
Average number of double bonds in a fatty acid chain	27005778	-0.161	0.132	-1.218	0.223	0.740	0.172	0.053	1.0015	0.0076	0.0045	0.0044
Average number of methylene groups per a double bond	27005778	0.149	0.130	1.144	0.253	0.772	0.202	0.079	0.9956	0.0081	-0.0064	0.0044
Average weekly beer plus cider intake	0	0.160	0.089	1.800	0.072	0.505	0.052	0.004	1.063	0.0094	0.005	0.0054
Average weekly champagne plus white wine intake	0	-0.225	0.080	-2.830	0.005	0.122	0.031	0.003	1.0401	0.0077	-5.00E-04	0.0044
Average weekly fortified wine intake	0	-0.223	0.142	-1.577	0.115	0.594	0.013	0.002	1.0173	0.0066	3.00E-04	0.0047
Average weekly intake of other alcoholic drinks	0	0.390	0.286	1.365	0.172	0.674	0.008	0.006	1.0035	0.0065	-0.0055	0.0049
Average weekly red wine intake	0	-0.133	0.074	-1.811	0.070	0.505	0.057	0.003	1.0124	0.0074	-0.0053	0.0046
Average weekly spirits intake	0	-0.017	0.090	-0.190	0.850	0.985	0.032	0.003	1.0166	0.0077	-8.00E-04	0.0047
Back pain for 3+ months	0	-0.121	0.135	-0.896	0.370	0.867	0.040	0.006	0.9919	0.0066	0.0107	0.0048
Basal metabolic rate	0	0.024	0.046	0.523	0.601	0.980	0.284	0.012	1.1164	0.0274	-0.0054	0.0073
Bilateral oophorectomy (both ovaries removed)	0	-0.015	0.118	-0.125	0.900	0.986	0.021	0.003	1.0116	0.0072	9.00E-04	0.005
Bipolar disorder	21926972	0.011	0.095	0.119	0.906	0.986	0.435	0.037	1.0219	0.0072	0.0346	0.005
Birth weight	0	0.116	0.066	1.743	0.081	0.505	0.108	0.006	1.0516	0.0137	-0.0042	0.0057
Birth weight	27680694	0.051	0.068	0.756	0.450	0.918	0.103	0.007	1.0406	0.0097	-0.0021	0.0049
Birth weight of first child	0	0.080	0.073	1.098	0.272	0.782	0.109	0.006	1.0235	0.0099	-0.0055	0.0053
od clot_ DVT_ bronchitis_ emphysema_ asthma_ rhinitis_ eczema_ allergy diagnosed by doctor: Asth	0	0.006	0.065	0.089	0.929	0.986	0.057	0.006	1.0267	0.0148	-0.0023	0.0047
VT_ bronchitis_ emphysema_ asthma_ rhinitis_ eczema_ allergy diagnosed by doctor: Blood clot in	0	0.109	0.136	0.804	0.421	0.910	0.011	0.002	1.0118	0.0093	-0.0073	0.0052
_ DVT_ bronchitis_ emphysema_ asthma_ rhinitis_ eczema_ allergy diagnosed by doctor: Blood clot	0	0.182	0.147	1.237	0.216	0.740	0.006	0.002	1.0081	0.0074	-0.0011	0.0048
_ bronchitis_ emphysema_ asthma_ rhinitis_ eczema_ allergy diagnosed by doctor: Emphysema/ch	0	0.194	0.119	1.625	0.104	0.555	0.013	0.002	0.9851	0.0073	0.0017	0.0049
bronchitis_ emphysema_ asthma_ rhinitis_ eczema_ allergy diagnosed by doctor: Hayfever_ allergic	0	-0.063	0.063	-1.011	0.312	0.813	0.073	0.006	1.042	0.0153	-0.0058	0.0053
t_ DVT_ bronchitis_ emphysema_ asthma_ rhinitis_ eczema_ allergy diagnosed by doctor: None of t	0	0.003	0.062	0.048	0.961	0.995	0.077	0.006	1.0373	0.0167	0.0071	0.0053
Body fat	26833246	0.103	0.088	1.181	0.238	0.758	0.107	0.008	0.9045	0.0062	-0.0041	0.0046
Body fat percentage	0	0.073	0.051	1.434	0.152	0.631	0.217	0.007	1.0835	0.0173	-0.0049	0.0075
Body mass index	20935630	0.130	0.066	1.967	0.049	0.444	0.189	0.010	1.0115	0.0104	-0.012	0.0051
Body mass index (BMI)	0	0.063	0.053	1.179	0.238	0.758	0.247	0.009	1.0735	0.0185	-0.0023	0.0079
Breastfed as a baby	0	0.084	0.092	0.911	0.363	0.856	0.026	0.002	1.0217	0.0069	-0.0171	0.005
Bring up phlegm/sputum/mucus on most days	0	0.210	0.135	1.554	0.120	0.602	0.026	0.006	1.0082	0.0072	-0.0028	0.0047
Cancer code_ self-reported: basal cell carcinoma	0	0.063	0.123	0.510	0.610	0.980	0.011	0.002	0.9971	0.0079	-0.0019	0.0051
Cancer code_ self-reported: breast cancer	0	0.144	0.131	1.101	0.271	0.782	0.010	0.002	1.0182	0.0079	-0.0027	0.0048
Cancer code_ self-reported: lung cancer	0	0.237	0.675	0.351	0.726	0.985	0.001	0.001	0.9924	0.0066	2.00E-04	0.0048
Cancer code_ self-reported: malignant melanoma	0	-0.255	0.172	-1.483	0.138	0.611	0.005	0.002	0.9939	0.0062	0.0058	0.0048
Cancer code_ self-reported: prostate cancer	0	0.158	0.159	0.993	0.321	0.817	0.006	0.002	1.0145	0.0075	-0.004	0.0048
Cancer code_ self-reported: small intestine/small bowel cancer	0	-0.473	0.488	-0.970	0.332	0.831	0.001	0.001	0.9985	0.007	0.0104	0.0051
Cancer code_ self-reported: squamous cell carcinoma	0	0.068	0.251	0.271	0.787	0.985	0.002	0.001	0.9996	0.0065	-0.0044	0.0045
Cancer diagnosed by doctor	0	-0.041	0.147	-0.280	0.780	0.985	0.007	0.002	1.0099	0.0064	0.0065	0.0046
Celiac disease	20190752	-0.077	0.147	-0.525	0.600	0.980	0.313	0.048	1.0643	0.0107	0.0563	0.0066
Chest pain or discomfort	0	0.016	0.074	0.218	0.828	0.985	0.033	0.002	1.0166	0.0079	0.0027	0.0049
Chest pain or discomfort walking normally	0	0.126	0.126	1.002	0.317	0.813	0.075	0.009	1.0089	0.0071	0.0083	0.005
Child birth length	25281659	0.014	0.105	0.137	0.891	0.986	0.171	0.023	0.9922	0.007	0.0043	0.0045
Child birth weight	23202124	0.077	0.126	0.613	0.540	0.975	0.114	0.018	1.0033	0.0058	1.00E-04	0.0044
Childhood IQ	23358156	-0.122	0.135	-0.902	0.367	0.864	0.275	0.045	1.0018	0.0092	-0.0037	0.006

Childhood obesity	22484627	-0.051	0.099	-0.516	0.606	0.980	0.414	0.046	0.9262	0.0076	0.0104	0.0049
Cholesterol esters in large HDL	27005778	0.073	0.136	0.533	0.594	0.980	0.122	0.031	1.0013	0.0141	0.0021	0.0043
Cholesterol esters in large LDL	27005778	-0.043	0.212	-0.200	0.841	0.985	0.075	0.049	1.0127	0.0216	0.0015	0.0051
Cholesterol esters in large VLDL	27005778	0.151	0.124	1.211	0.226	0.741	0.163	0.034	0.9781	0.0068	-0.0041	0.0041
Cholesterol esters in medium HDL	27005778	-0.046	0.198	-0.235	0.814	0.985	0.054	0.029	1.0005	0.0089	0.0038	0.0046
Cholesterol esters in medium LDL	27005778	-0.063	0.206	-0.303	0.762	0.985	0.080	0.048	1.0104	0.0206	0.002	0.005
Cholesterol esters in medium VLDL	27005778	0.084	0.125	0.674	0.501	0.944	0.151	0.037	0.9864	0.0078	-0.0027	0.0043
Chronic Kidney Disease	26831199	-0.190	0.169	-1.123	0.262	0.772	0.018	0.006	1.017	0.0088	0.0215	0.0053
Chronotype	27494321	0.009	0.074	0.118	0.906	0.986	0.102	0.006	1.0139	0.0076	-0.0027	0.0049
Cigarettes smoked per day	20418890	-0.001	0.151	-0.006	0.995	0.995	0.060	0.015	1.0046	0.006	-7.00E-04	0.0045
Citrate	27005778	-0.209	0.180	-1.163	0.245	0.768	0.071	0.021	1.0122	0.0075	0.0041	0.0047
College completion	23722424	-0.268	0.099	-2.712	0.007	0.157	0.079	0.006	1.021	0.0088	0.0079	0.0059
Comparative body size at age 10	0	0.042	0.056	0.742	0.458	0.918	0.146	0.009	1.0161	0.0166	-0.0135	0.0061
Comparative height size at age 10	0	-0.002	0.047	-0.040	0.968	0.995	0.241	0.012	1.1147	0.0342	-0.0066	0.0067
Concentration of chylomicrons and largest VLDL particles	27005778	0.111	0.143	0.777	0.437	0.918	0.115	0.027	0.9887	0.0064	8.00E-04	0.0042
Concentration of IDL particles	27005778	0.024	0.191	0.123	0.902	0.986	0.075	0.042	1.0185	0.0179	0.0015	0.0047
Concentration of large HDL particles	27005778	0.038	0.135	0.285	0.775	0.985	0.130	0.030	1.0003	0.015	0.0033	0.0041
Concentration of large LDL particles	27005778	-0.022	0.204	-0.107	0.915	0.986	0.075	0.049	1.0163	0.0213	0.0013	0.0049
Concentration of large VLDL particles	27005778	0.072	0.128	0.564	0.573	0.980	0.131	0.034	0.9742	0.0068	-0.001	0.0041
Concentration of medium HDL particles	27005778	0.014	0.181	0.078	0.938	0.989	0.063	0.026	0.9956	0.0075	0.0036	0.0046
Concentration of medium LDL particles	27005778	-0.036	0.203	-0.176	0.860	0.986	0.079	0.047	1.012	0.0199	0.001	0.0049
Concentration of medium VLDL particles	27005778	0.140	0.123	1.137	0.255	0.772	0.150	0.035	0.9806	0.0068	-0.0015	0.0039
Concentration of small LDL particles	27005778	0.005	0.177	0.029	0.977	0.995	0.093	0.042	1.0073	0.0158	-3.34E-05	0.0047
Concentration of small VLDL particles	27005778	0.049	0.128	0.381	0.703	0.985	0.150	0.037	0.9905	0.0078	-0.0026	0.0043
Concentration of very large HDL particles	27005778	0.144	0.187	0.770	0.441	0.918	0.064	0.027	1.0023	0.0148	-5.00E-04	0.0044
Concentration of very large VLDL particles	27005778	0.099	0.147	0.675	0.499	0.944	0.124	0.030	0.9838	0.0067	-0.0025	0.0045
Concentration of very small VLDL particles	27005778	0.046	0.143	0.320	0.749	0.985	0.116	0.036	1.0054	0.0122	6.66E-05	0.0045
Coronary artery disease	26343387	-0.021	0.068	-0.309	0.757	0.985	0.079	0.006	1.0442	0.0093	0.0361	0.0047
Cough on most days	0	-0.062	0.130	-0.475	0.635	0.980	0.048	0.006	0.9985	0.0069	0.0051	0.0056
Creatinine	27005778	-0.218	0.135	-1.616	0.106	0.558	0.114	0.024	1.0123	0.0071	0.0095	0.0046
Creatinine (enzymatic) in urine	0	-0.029	0.067	-0.440	0.660	0.985	0.066	0.003	1.0191	0.0087	0.0044	0.0058
Crohns disease	26192919	0.084	0.085	0.981	0.326	0.825	0.505	0.057	1.0219	0.0114	0.0479	0.0049
Current employment status: Doing unpaid or voluntary work	0	-0.296	0.145	-2.050	0.040	0.433	0.009	0.001	1.0085	0.0063	0.0049	0.005
Current employment status: In paid employment or self-employed	0	-0.217	0.140	-1.547	0.122	0.602	0.008	0.002	1.0188	0.0067	0.0062	0.0049
Current employment status: Looking after home and/or family	0	-0.337	0.154	-2.190	0.029	0.398	0.006	0.001	1.0002	0.0067	0.0019	0.0046
Current employment status: Retired	0	-0.041	0.147	-0.276	0.782	0.985	0.008	0.002	1.0164	0.007	-0.006	0.0051
Current employment status: Unable to work because of sickness or disability	0	0.182	0.096	1.896	0.058	0.459	0.025	0.002	1.0176	0.0073	0.0074	0.0052
Current tobacco smoking	0	0.146	0.073	1.995	0.046	0.444	0.055	0.003	1.0136	0.0092	0.0014	0.005
Daytime dozing / sleeping (narcolepsy)	0	0.091	0.068	1.335	0.182	0.689	0.049	0.003	1.0101	0.0087	-0.0042	0.005
Depressive symptoms	27089181	0.107	0.088	1.212	0.226	0.741	0.047	0.004	1.0002	0.0071	0.01	0.005
Description of average fatty acid chain length; not actual carbon number	27005778	-0.130	0.188	-0.690	0.490	0.937	0.100	0.034	0.9831	0.0065	-0.0012	0.0044
Diabetes diagnosed by doctor	0	-0.065	0.071	-0.923	0.356	0.849	0.044	0.003	1.0397	0.0104	0.0107	0.0053
Diagnoses - main ICD10: B37 Candidiasis	0	0.795	1.584	0.502	0.616	0.980	0.001	0.001	1.0018	0.0064	-0.0046	0.0042

Diagnoses - main ICD10: C44 Other malignant neoplasms of skin	0	0.031	0.128	0.241	0.809	0.985	0.010	0.002	1.0106	0.0086	0.0043	0.0053
Diagnoses - main ICD10: C50 Malignant neoplasm of breast	0	0.094	0.133	0.712	0.476	0.922	0.009	0.002	1.003	0.0076	-6.00E-04	0.0045
Diagnoses - main ICD10: C61 Malignant neoplasm of prostate	0	0.098	0.203	0.483	0.629	0.980	0.004	0.002	1.0192	0.0067	-1.00E-04	0.005
Diagnoses - main ICD10: D12 Benign neoplasm of colon_ rectum_ anus and anal canal	0	0.222	0.126	1.756	0.079	0.505	0.009	0.001	1.0035	0.0063	-0.0039	0.005
Diagnoses - main ICD10: D25 Leiomyoma of uterus	0	0.048	0.155	0.307	0.759	0.985	0.006	0.001	0.993	0.006	0.0015	0.0048
Diagnoses - main ICD10: E04 Other non-toxic goitre	0	0.136	0.177	0.767	0.443	0.918	0.005	0.001	0.9924	0.0063	4.00E-04	0.0047
Diagnoses - main ICD10: F31 Bipolar affective disorder	0	-0.348	0.183	-1.901	0.057	0.458	0.005	0.002	0.9874	0.007	0.0091	0.0042
Diagnoses - main ICD10: F43 Reaction to severe stress_ and adjustment disorders	0	-0.276	0.617	-0.447	0.655	0.985	0.001	0.001	1.0028	0.0055	0.0054	0.0049
Diagnoses - main ICD10: G47 Sleep disorders	0	-0.005	0.169	-0.031	0.975	0.995	0.006	0.002	0.9946	0.007	-0.0065	0.0048
Diagnoses - main ICD10: G56 Mononeuropathies of upper limb	0	-0.114	0.108	-1.065	0.287	0.793	0.014	0.002	1.0085	0.0073	0.0081	0.0048
Diagnoses - main ICD10: H25 Senile cataract	0	0.284	0.218	1.301	0.193	0.702	0.003	0.001	1.0131	0.0069	-0.0065	0.005
Diagnoses - main ICD10: H26 Other cataract	0	0.030	0.112	0.267	0.790	0.985	0.009	0.002	0.9934	0.0073	4.00E-04	0.0047
Diagnoses - main ICD10: I10 Essential (primary) hypertension	0	-0.139	0.244	-0.569	0.569	0.980	0.003	0.001	0.9976	0.006	0.0012	0.0048
Diagnoses - main ICD10: I20 Angina pectoris	0	-0.072	0.144	-0.500	0.617	0.980	0.008	0.002	1.0093	0.0069	0.0104	0.0048
Diagnoses - main ICD10: I21 Acute myocardial infarction	0	-0.381	0.129	-2.957	0.003	0.092	0.010	0.002	1.0059	0.0072	0.0079	0.0047
Diagnoses - main ICD10: I25 Chronic ischaemic heart disease	0	-0.127	0.099	-1.283	0.199	0.706	0.020	0.002	1.0203	0.0072	0.0129	0.0049
Diagnoses - main ICD10: I30 Acute pericarditis	0	0.076	0.249	0.304	0.761	0.985	0.002	0.001	0.9947	0.0068	-0.0031	0.005
Diagnoses - main ICD10: I48 Atrial fibrillation and flutter	0	-0.127	0.110	-1.149	0.251	0.772	0.013	0.002	1.0108	0.0079	0.0036	0.0047
Diagnoses - main ICD10: I80 Phlebitis and thrombophlebitis	0	-0.024	0.156	-0.153	0.879	0.986	0.008	0.002	0.9942	0.0084	-6.00E-04	0.0047
Diagnoses - main ICD10: I83 Varicose veins of lower extremities	0	0.165	0.096	1.729	0.084	0.511	0.019	0.002	1.0343	0.0111	-0.0028	0.0047
Diagnoses - main ICD10: I84 Haemorrhoids	0	0.004	0.121	0.034	0.973	0.995	0.009	0.002	1.0021	0.0074	0.0039	0.005
Diagnoses - main ICD10: J22 Unspecified acute lower respiratory infection	0	0.141	0.241	0.582	0.561	0.980	0.003	0.002	0.9997	0.0073	-0.0069	0.0052
Diagnoses - main ICD10: J33 Nasal polyp	0	0.003	0.172	0.017	0.986	0.995	0.003	0.002	1.0075	0.0072	-0.001	0.0043
Diagnoses - main ICD10: J34 Other disorders of nose and nasal sinuses	0	0.166	0.242	0.686	0.493	0.940	0.002	0.001	1.0079	0.0066	-0.0039	0.0046
Diagnoses - main ICD10: J44 Other chronic obstructive pulmonary disease	0	-0.313	0.179	-1.748	0.081	0.505	0.005	0.001	0.996	0.0067	0.0075	0.0047
Diagnoses - main ICD10: K20 Oesophagitis	0	0.184	0.261	0.703	0.482	0.928	0.002	0.001	1.0066	0.0064	9.00E-04	0.0044
Diagnoses - main ICD10: K21 Gastro-oesophageal reflux disease	0	0.136	0.152	0.893	0.372	0.867	0.006	0.001	1.0022	0.0057	0.0034	0.0045
Diagnoses - main ICD10: K22 Other diseases of oesophagus	0	0.067	0.208	0.323	0.747	0.985	0.004	0.002	1.0045	0.0065	0.0025	0.0051
Diagnoses - main ICD10: K29 Gastritis and duodenitis	0	0.284	0.146	1.951	0.051	0.444	0.007	0.002	1.0043	0.0065	-0.0013	0.0049
Diagnoses - main ICD10: K30 Dyspepsia	0	0.051	0.223	0.228	0.820	0.985	0.003	0.001	1.0041	0.0064	-0.0019	0.0052
Diagnoses - main ICD10: K35 Acute appendicitis	0	0.388	0.265	1.465	0.143	0.612	0.002	0.001	1.0043	0.0068	0.0034	0.0047
Diagnoses - main ICD10: K40 Inguinal hernia	0	-0.175	0.103	-1.690	0.091	0.525	0.018	0.002	0.9913	0.0069	0.0054	0.005
Diagnoses - main ICD10: K43 Ventral hernia	0	0.140	0.188	0.742	0.458	0.918	0.005	0.001	0.9931	0.0065	-0.0023	0.0056
Diagnoses - main ICD10: K44 Diaphragmatic hernia	0	-0.150	0.168	-0.893	0.372	0.867	0.005	0.001	1.0053	0.0064	-0.0015	0.0048
Diagnoses - main ICD10: K50 Crohns disease [regional enteritis]	0	-0.228	0.171	-1.333	0.183	0.689	0.004	0.001	1.0027	0.0067	0.0056	0.0045
Diagnoses - main ICD10: K51 Ulcerative colitis	0	-0.030	0.166	-0.181	0.857	0.985	0.005	0.002	1.0131	0.0066	0.0016	0.0049
Diagnoses - main ICD10: K52 Other non-infective gastro-enteritis and colitis	0	-0.121	0.162	-0.746	0.456	0.918	0.005	0.001	1.0038	0.0061	0.0088	0.0043
Diagnoses - main ICD10: K57 Diverticular disease of intestine	0	0.073	0.105	0.694	0.488	0.937	0.013	0.002	1.0036	0.0065	4.00E-04	0.005
Diagnoses - main ICD10: K60 Fissure and fistula of anal and rectal regions	0	0.029	0.157	0.187	0.851	0.985	0.006	0.001	0.9814	0.0067	-0.0033	0.0047
Diagnoses - main ICD10: K62 Other diseases of anus and rectum	0	-0.175	0.170	-1.031	0.303	0.802	0.005	0.002	1.0017	0.0067	0.0105	0.0047
Diagnoses - main ICD10: K76 Other diseases of liver	0	-0.136	0.190	-0.715	0.475	0.921	0.004	0.001	0.9931	0.0062	0.0017	0.0044
Diagnoses - main ICD10: K80 Cholelithiasis	0	-0.123	0.134	-0.919	0.358	0.849	0.010	0.002	1.0235	0.0084	0.0086	0.0045
Diagnoses - main ICD10: L03 Cellulitis	0	0.080	0.185	0.432	0.666	0.985	0.004	0.002	1.0029	0.0069	2.00E-04	0.0048

Diagnoses - main ICD10: M10 Gout	0	0.588	0.336	1.748	0.081	0.505	0.002	0.002	0.9997	0.0068	-0.0046	0.0048
Diagnoses - main ICD10: M16 Coxarthrosis [arthrosis of hip]	0	-0.009	0.148	-0.061	0.951	0.991	0.011	0.002	1.0103	0.0081	-0.001	0.0054
Diagnoses - main ICD10: M17 Gonarthrosis [arthrosis of knee]	0	0.215	0.110	1.957	0.050	0.444	0.014	0.002	1.0063	0.007	-0.0083	0.0048
Diagnoses - main ICD10: M20 Acquired deformities of fingers and toes	0	-0.071	0.131	-0.541	0.588	0.980	0.009	0.002	1.0007	0.0071	-4.00E-04	0.0051
Diagnoses - main ICD10: M21 Other acquired deformities of limbs	0	0.412	1.792	0.230	0.818	0.985	0.000	0.001	1.0082	0.0066	-6.00E-04	0.0046
Diagnoses - main ICD10: M23 Internal derangement of knee	0	0.034	0.126	0.270	0.787	0.985	0.008	0.002	1.0053	0.0072	0.0046	0.0042
Diagnoses - main ICD10: M24 Other specific joint derangements	0	-0.047	0.211	-0.223	0.824	0.985	0.004	0.001	0.9878	0.0061	-1.47E-06	0.0045
Diagnoses - main ICD10: M25 Other joint disorders_ not elsewhere classified	0	-0.213	0.392	-0.543	0.587	0.980	0.001	0.001	1.0157	0.0068	0.0054	0.0047
Diagnoses - main ICD10: M54 Dorsalgia	0	0.344	0.126	2.725	0.006	0.155	0.010	0.002	1.0044	0.0072	2.00E-04	0.0046
Diagnoses - main ICD10: M67 Other disorders of synovium and tendon	0	-0.180	0.279	-0.644	0.520	0.956	0.002	0.001	0.9993	0.0062	0.0044	0.0049
Diagnoses - main ICD10: M70 Soft tissue disorders related to use_ overuse and pressure	0	0.253	0.189	1.338	0.181	0.689	0.004	0.001	0.983	0.0066	-0.0092	0.0045
Diagnoses - main ICD10: M72 Fibroblastic disorders	0	0.287	0.146	1.970	0.049	0.444	0.011	0.003	1.0169	0.0205	-0.007	0.0053
Diagnoses - main ICD10: N19 Unspecified renal failure	0	0.014	0.226	0.061	0.951	0.991	0.003	0.001	0.9995	0.0067	-0.0031	0.0048
Diagnoses - main ICD10: N20 Calculus of kidney and ureter	0	0.250	0.141	1.768	0.077	0.505	0.008	0.001	1.0081	0.0066	-0.0059	0.0047
Diagnoses - main ICD10: N32 Other disorders of bladder	0	-0.095	0.186	-0.509	0.611	0.980	0.004	0.001	0.9894	0.0058	0.0059	0.0045
Diagnoses - main ICD10: N40 Hyperplasia of prostate	0	-0.114	0.165	-0.691	0.490	0.937	0.006	0.002	1.0098	0.0068	8.00E-04	0.0045
Diagnoses - main ICD10: N81 Female genital prolapse	0	0.146	0.147	0.996	0.319	0.815	0.007	0.002	1.0243	0.0065	-0.0036	0.0046
Diagnoses - main ICD10: N92 Excessive_ frequent and irregular menstruation	0	0.028	0.153	0.185	0.854	0.985	0.007	0.002	1.0008	0.0073	0.0016	0.005
Diagnoses - main ICD10: O75 Other complications of labour and delivery_ not elsewhere classified	0	0.158	0.218	0.724	0.469	0.918	0.003	0.001	0.9873	0.0058	-0.0049	0.0049
Diagnoses - main ICD10: R04 Haemorrhage from respiratory passages	0	0.072	0.218	0.328	0.743	0.985	0.003	0.001	0.9973	0.0063	0.0011	0.0049
Diagnoses - main ICD10: R07 Pain in throat and chest	0	0.180	0.101	1.792	0.073	0.505	0.016	0.002	1.0057	0.0076	0.0018	0.0048
Diagnoses - main ICD10: R10 Abdominal and pelvic pain	0	0.243	0.107	2.268	0.023	0.348	0.012	0.002	0.9962	0.0073	-0.0011	0.0049
Diagnoses - main ICD10: R11 Nausea and vomiting	0	-0.032	0.384	-0.084	0.933	0.986	0.001	0.001	0.9946	0.0061	3.00E-04	0.0052
Diagnoses - main ICD10: R14 Flatulence and related conditions	0	-0.108	0.225	-0.482	0.630	0.980	0.003	0.001	0.9921	0.0067	3.08E-06	0.0048
Diagnoses - main ICD10: R31 Unspecified haematuria	0	0.326	0.212	1.539	0.124	0.602	0.003	0.002	1.0077	0.0061	-0.0014	0.0047
Diagnoses - main ICD10: R35 Polyuria	0	0.206	0.245	0.840	0.401	0.896	0.003	0.002	0.9979	0.0066	-0.001	0.0044
Diagnoses - main ICD10: R55 Syncope and collapse	0	-0.049	0.173	-0.284	0.776	0.985	0.005	0.001	1.0083	0.0071	0.0026	0.0048
Diagnoses - main ICD10: R69 Unknown and unspecified causes of morbidity	0	-0.118	0.159	-0.743	0.458	0.918	0.005	0.001	0.9981	0.0068	0.0078	0.0048
Diagnoses - main ICD10: S09 Other and unspecified injuries of head	0	0.067	0.219	0.307	0.759	0.985	0.004	0.002	0.9938	0.0063	-0.0028	0.005
Diagnoses - main ICD10: S52 Fracture of forearm	0	-0.044	0.194	-0.225	0.822	0.985	0.006	0.002	0.9987	0.007	-0.0032	0.005
Diagnoses - main ICD10: S66 Injury of muscle and tendon at wrist and hand level	0	0.146	0.265	0.552	0.581	0.980	0.002	0.001	0.9996	0.0066	-0.0022	0.0048
Diagnoses - main ICD10: S76 Injury of muscle and tendon at hip and thigh level	0	-0.137	0.284	-0.483	0.629	0.980	0.002	0.001	0.998	0.0065	0.0046	0.004
gnoses - main ICD10: T84 Complications of internal orthopaedic prosthetic devices_ implants and gra	0	0.293	0.178	1.647	0.100	0.553	0.005	0.002	0.9902	0.0069	-0.0095	0.0051
es - main ICD10: Z09 Follow-up examination after treatment for conditions other than malignant neo	0	-0.067	0.196	-0.343	0.732	0.985	0.003	0.001	0.9991	0.0061	-0.0017	0.0044
Diagnoses - main ICD10: Z47 Other orthopaedic follow-up care	0	0.066	0.222	0.298	0.766	0.985	0.004	0.001	0.9974	0.0062	0.002	0.0048
Diagnoses - main ICD10: Z80 Family history of malignant neoplasm	0	-0.169	0.353	-0.479	0.632	0.980	0.001	0.001	0.9908	0.0059	-0.0029	0.0046
Diastolic blood pressure_ automated reading	0	-0.053	0.064	-0.833	0.405	0.900	0.136	0.006	1.0665	0.0156	0.0065	0.007
Difference in height between adolescence and adulthood; age 14	23449627	0.243	0.159	1.527	0.127	0.602	0.453	0.110	0.9809	0.0067	-0.0071	0.005
Difference in height between childhood and adulthood; age 8	23449627	0.221	0.142	1.556	0.120	0.602	0.335	0.051	0.9721	0.0077	-0.0094	0.0052
Difficulty not smoking for 1 day	0	0.015	0.145	0.106	0.916	0.986	0.111	0.022	0.9894	0.0065	-0.0017	0.0049
Distance between home and job workplace	0	0.044	0.179	0.248	0.804	0.985	0.012	0.003	1.0076	0.0068	0.0042	0.0051
Doctor diagnosed asthma	0	0.130	0.100	1.306	0.192	0.702	0.069	0.010	0.9993	0.0078	-0.0031	0.005
Doctor diagnosed hayfever or allergic rhinitis	0	0.065	0.098	0.663	0.508	0.950	0.079	0.009	1.009	0.008	-0.0031	0.0052

Drive faster than motorway speed limit	0	-0.072	0.070	-1.033	0.302	0.802	0.057	0.003	1.0295	0.0089	-0.0076	0.0056
Duration of fitness test	0	-0.232	0.222	-1.045	0.296	0.801	0.021	0.009	1.0041	0.0064	-2.00E-04	0.0046
Duration of heavy DIY	0	0.081	0.109	0.743	0.458	0.918	0.033	0.004	1.0075	0.0067	0.0036	0.0048
Duration of light DIY	0	0.090	0.098	0.924	0.355	0.849	0.035	0.003	1.0038	0.0073	-0.0028	0.005
Duration of moderate activity	0	0.283	0.084	3.374	0.001	0.031	0.032	0.002	1.0094	0.0075	-0.0074	0.0043
Duration of other exercises	0	0.289	0.134	2.160	0.031	0.421	0.019	0.003	1.0086	0.007	-0.0095	0.005
Duration of strenuous sports	0	-0.226	0.208	-1.086	0.278	0.783	0.031	0.014	0.9941	0.0068	9.00E-04	0.0051
Duration of vigorous activity	0	0.281	0.092	3.065	0.002	0.069	0.036	0.003	0.9902	0.0074	-0.0066	0.0051
Duration of walks	0	0.137	0.081	1.698	0.090	0.525	0.047	0.003	1.0071	0.0082	-0.0013	0.0053
Duration walking for pleasure	0	-0.140	0.099	-1.410	0.159	0.645	0.025	0.002	1.011	0.0069	0.0039	0.0053
Eczema	26482879	-0.156	0.139	-1.123	0.261	0.772	0.068	0.015	1.0238	0.0072	0.0231	0.005
Ever depressed for a whole week	0	-0.031	0.093	-0.329	0.742	0.985	0.064	0.005	0.9924	0.0069	0.0049	0.0048
Ever had bowel cancer screening	0	-0.128	0.121	-1.064	0.287	0.793	0.013	0.002	1.0205	0.0069	0.0068	0.0053
Ever had hysterectomy (womb removed)	0	0.014	0.137	0.099	0.921	0.986	0.022	0.003	1.0052	0.0073	0.0052	0.0052
Ever had prostate specific antigen (PSA) test	0	-0.011	0.106	-0.100	0.920	0.986	0.034	0.003	1.0036	0.0073	-0.0023	0.0049
Ever had stillbirth_ spontaneous miscarriage or termination	0	0.212	0.121	1.746	0.081	0.505	0.020	0.003	0.9955	0.0078	-0.0107	0.0044
Ever highly irritable/argumentative for 2 days	0	0.108	0.096	1.124	0.261	0.772	0.052	0.006	0.9986	0.0077	0.007	0.0047
Ever manic/hyper for 2 days	0	0.141	0.160	0.884	0.377	0.868	0.018	0.005	0.9952	0.0065	-3.00E-04	0.0046
Ever smoked	0	0.114	0.061	1.887	0.059	0.463	0.075	0.003	1.0216	0.0101	-0.0024	0.0053
Ever stopped smoking for 6+ months	0	0.317	0.179	1.774	0.076	0.505	0.020	0.005	1.0007	0.0066	-0.0025	0.0049
Ever taken oral contraceptive pill	0	-0.171	0.116	-1.471	0.141	0.612	0.019	0.003	1.0116	0.007	0.0011	0.0045
Ever unenthusiastic/disinterested for a whole week	0	0.010	0.099	0.098	0.922	0.986	0.058	0.006	1.0001	0.0073	0.0061	0.0047
Ever used hormone-replacement therapy (HRT)	0	0.062	0.094	0.653	0.514	0.953	0.041	0.003	0.9998	0.0072	0.0024	0.0049
Ever vs never smoked	20418890	0.168	0.104	1.616	0.106	0.558	0.075	0.007	0.9965	0.0059	-0.0054	0.0046
Excessive daytime sleepiness	27992416	0.085	0.097	0.878	0.380	0.868	0.054	0.005	1.0034	0.0068	-0.0031	0.0048
Exposure to tobacco smoke at home	0	0.420	0.122	3.428	0.001	0.030	0.012	0.002	0.9987	0.0063	-0.0025	0.0048
Exposure to tobacco smoke outside home	0	0.161	0.086	1.868	0.062	0.478	0.032	0.002	1.0198	0.0083	-0.0022	0.005
Extreme bmi	23563607	0.121	0.101	1.199	0.231	0.741	0.691	0.055	1.03	0.0113	-0.0116	0.0069
Extreme height	23563607	-0.003	0.073	-0.042	0.967	0.995	1.228	0.108	1.0372	0.0175	-0.0102	0.0079
Extreme waist-to-hip ratio	23563607	-0.045	0.118	-0.385	0.700	0.985	0.360	0.057	0.9773	0.0085	0.0021	0.006
Eye problems/disorders: Cataract	0	0.008	0.129	0.062	0.951	0.991	0.023	0.004	1.0133	0.0068	-0.0022	0.0046
Eye problems/disorders: Diabetes related eye disease	0	-0.210	0.141	-1.488	0.137	0.611	0.020	0.004	1.0062	0.0061	0.0031	0.0047
Eye problems/disorders: Glaucoma	0	0.082	0.126	0.655	0.512	0.953	0.039	0.006	1.0196	0.0079	-0.0076	0.0056
Eye problems/disorders: Injury or trauma resulting in loss of vision	0	0.147	0.783	0.188	0.851	0.985	0.001	0.004	1.0104	0.0061	0.002	0.0051
Falls in the last year	0	0.163	0.079	2.064	0.039	0.433	0.033	0.002	1.004	0.0077	-0.0021	0.0051
Family relationship satisfaction	0	-0.104	0.088	-1.176	0.239	0.759	0.058	0.005	1.0039	0.0072	0.0083	0.0045
Fasting glucose main effect	22581228	0.083	0.100	0.836	0.403	0.899	0.098	0.019	0.9985	0.0094	-0.0013	0.0045
Fasting insulin main effect	22581228	-0.125	0.131	-0.950	0.342	0.840	0.070	0.010	1.0157	0.0069	-0.0018	0.0047
Fasting proinsulin	20081858	-0.240	0.184	-1.308	0.191	0.702	0.173	0.089	0.9844	0.0097	0.0111	0.0043
Father still alive	0	-0.089	0.159	-0.557	0.577	0.980	0.006	0.002	1.0165	0.0066	4.00E-04	0.0048
Fathers age at death	27015805	-0.465	0.146	-3.194	0.001	0.053	0.040	0.007	1.0173	0.0068	0.0047	0.0048
Fathers age at death	0	-0.212	0.103	-2.059	0.040	0.433	0.034	0.003	1.0229	0.0078	0.0027	0.0055
Fed-up feelings	0	0.065	0.065	1.005	0.315	0.813	0.072	0.003	1.0241	0.0101	0.0039	0.0053

Femoral Neck bone mineral density	26367794	0.035	0.089	0.392	0.695	0.985	0.126	0.015	0.9751	0.008	0.0034	0.0046
Femoral neck bone mineral density	22504420	0.009	0.075	0.116	0.908	0.986	0.304	0.025	0.9799	0.0086	-0.0022	0.0047
Ferritin	25352340	-0.047	0.161	-0.295	0.768	0.985	0.091	0.027	1.0279	0.0093	2.00E-04	0.0063
Financial situation satisfaction	0	0.219	0.103	2.118	0.034	0.433	0.057	0.006	1.0058	0.008	-0.0058	0.0056
Fluid intelligence score	0	-0.338	0.067	-5.036	0.000	0.000	0.238	0.011	1.0429	0.0103	0.0024	0.0054
Forced expiratory volume in 1 second (FEV1)	21946350	0.102	0.125	0.817	0.414	0.903	0.173	0.022	0.8807	0.0119	-0.0072	0.0079
Forced expiratory volume in 1 second (FEV1)	28166213	0.018	0.070	0.258	0.796	0.985	0.265	0.017	0.9704	0.007	-8.00E-04	0.0048
Forced expiratory volume in 1 second (FEV1)	26635082	0.039	0.101	0.387	0.699	0.985	0.141	0.016	0.9916	0.0067	-0.0029	0.0049
Forced expiratory volume in 1 second (FEV1)/Forced Vital capacity(FVC)	28166213	0.113	0.072	1.570	0.117	0.599	0.261	0.020	0.9709	0.0081	-0.0046	0.005
Forced expiratory volume in 1 second (FEV1)/Forced Vital capacity(FVC)	21946350	-0.087	0.091	-0.959	0.337	0.831	0.123	0.014	0.9289	0.0078	0.0075	0.0048
Forced expiratory volume in 1 second (FEV1)/Forced Vital capacity(FVC)	26635082	-0.031	0.118	-0.265	0.791	0.985	0.113	0.015	0.9816	0.007	0.0035	0.0048
Forced expiratory volume in 1-second (FEV1)	0	-0.005	0.052	-0.104	0.917	0.986	0.148	0.006	1.0653	0.0132	-0.0046	0.0064
Forced expiratory volume in 1-second (FEV1)_ Best measure	0	0.015	0.053	0.276	0.783	0.985	0.163	0.007	1.0573	0.0132	-0.0067	0.0065
Forced expiratory volume in 1-second (FEV1)_ predicted	0	-0.071	0.072	-0.991	0.322	0.817	0.172	0.012	1.0387	0.0118	8.00E-04	0.006
Forced expiratory volume in 1-second (FEV1)_ predicted percentage	0	-0.054	0.064	-0.855	0.393	0.888	0.202	0.010	1.0338	0.0097	0.0018	0.0056
Forced vital capacity (FVC)	0	0.008	0.049	0.171	0.864	0.986	0.170	0.007	1.0953	0.0167	-0.0076	0.0066
Forced vital capacity (FVC)_ Best measure	0	0.012	0.050	0.234	0.815	0.985	0.189	0.008	1.0816	0.0163	-0.0085	0.0066
Forced Vital capacity(FVC)	26635082	0.050	0.107	0.469	0.639	0.980	0.147	0.015	0.9855	0.0067	-0.0043	0.0049
Forced Vital capacity(FVC)	28166213	-0.018	0.070	-0.261	0.794	0.985	0.260	0.016	0.9769	0.0071	2.00E-04	0.0047
Forearm Bone mineral density	26367794	-0.092	0.211	-0.437	0.662	0.985	0.092	0.042	1.0109	0.0065	0.0036	0.0045
Former alcohol drinker	0	0.215	0.144	1.497	0.134	0.611	0.123	0.021	0.9966	0.0063	8.00E-04	0.0049
Former vs Current smoker	20418890	0.038	0.152	0.250	0.803	0.985	0.063	0.011	1.0008	0.0066	9.00E-04	0.0048
Fracture resulting from simple fall	0	0.193	0.180	1.073	0.283	0.789	0.050	0.014	0.9934	0.0062	-8.00E-04	0.0043
Fractured bone site(s): Ankle	0	0.166	0.147	1.133	0.257	0.772	0.006	0.002	0.9953	0.0066	-0.0031	0.0047
Fractured bone site(s): Arm	0	-0.069	0.198	-0.347	0.729	0.985	0.005	0.002	0.9965	0.0069	0.0017	0.0051
Fractured bone site(s): Other bones	0	-0.195	0.135	-1.437	0.151	0.631	0.009	0.002	1.0004	0.0069	0.0083	0.0047
Fractured bone site(s): Wrist	0	-0.035	0.168	-0.208	0.835	0.985	0.007	0.002	1.0081	0.007	0.0012	0.0048
Fractured/broken bones in last 5 years	0	-0.111	0.099	-1.121	0.262	0.772	0.019	0.002	1.008	0.0074	0.0091	0.0046
Free cholesterol	27005778	-0.191	0.259	-0.736	0.462	0.918	0.067	0.042	1.0236	0.0106	0.0069	0.0053
Free cholesterol in IDL	27005778	-0.045	0.192	-0.234	0.815	0.985	0.071	0.041	1.0201	0.0197	0.0045	0.0048
Free cholesterol in large HDL	27005778	0.080	0.143	0.561	0.575	0.980	0.105	0.028	1.0089	0.014	9.00E-04	0.004
Free cholesterol in large LDL	27005778	0.006	0.210	0.026	0.979	0.995	0.064	0.047	1.0171	0.0233	0.0019	0.0049
Free cholesterol in large VLDL	27005778	-0.008	0.127	-0.066	0.948	0.991	0.122	0.030	0.9893	0.0067	0.0031	0.0041
Free cholesterol in medium HDL	27005778	0.017	0.175	0.099	0.922	0.986	0.065	0.023	0.9984	0.0083	0.0034	0.0044
Free cholesterol in medium VLDL	27005778	0.041	0.133	0.306	0.760	0.985	0.111	0.032	0.996	0.0069	-4.00E-04	0.0039
Free cholesterol in small VLDL	27005778	0.021	0.148	0.143	0.886	0.986	0.111	0.033	1.0002	0.0087	2.00E-04	0.0044
Free cholesterol in very large HDL	27005778	0.197	0.204	0.965	0.334	0.831	0.049	0.025	1.0142	0.0136	-0.0018	0.0043
Free cholesterol to esterified cholesterol ratio	27005778	-0.205	0.276	-0.743	0.457	0.918	0.059	0.049	1.0132	0.0125	0.0043	0.005
Frequency of depressed mood in last 2 weeks	0	0.149	0.075	1.984	0.047	0.444	0.042	0.003	1.0117	0.0084	-9.00E-04	0.0051
Frequency of heavy DIY in last 4 weeks	0	-0.013	0.140	-0.089	0.929	0.986	0.020	0.004	1.01	0.0062	0.0022	0.0045
Frequency of light DIY in last 4 weeks	0	-0.046	0.099	-0.466	0.641	0.981	0.022	0.003	1.0043	0.0064	-0.0031	0.0043
Frequency of other exercises in last 4 weeks	0	0.273	0.142	1.927	0.054	0.456	0.021	0.003	0.9986	0.0065	-0.0032	0.0049
Frequency of stair climbing in last 4 weeks	0	-0.107	0.073	-1.465	0.143	0.612	0.035	0.002	1.017	0.0077	-0.0084	0.0054

Frequency of tenseness / restlessness in last 2 weeks	0	0.227	0.068	3.315	0.001	0.036	0.044	0.003	1.0141	0.0093	-1.00E-04	0.0048
Frequency of tiredness / lethargy in last 2 weeks	0	0.093	0.057	1.626	0.104	0.555	0.060	0.003	1.0241	0.0095	0.0051	0.0046
Frequency of travelling from home to job workplace	0	0.139	0.111	1.252	0.211	0.736	0.022	0.003	1.0173	0.0068	-0.0025	0.0047
Frequency of unenthusiasm / disinterest in last 2 weeks	0	0.158	0.071	2.228	0.026	0.367	0.039	0.003	1.0177	0.0089	0.0035	0.005
Frequency of walking for pleasure in last 4 weeks	0	0.046	0.085	0.544	0.586	0.980	0.034	0.003	1.0075	0.0077	-0.0012	0.0053
Friendships satisfaction	0	-0.161	0.093	-1.723	0.085	0.511	0.062	0.005	0.9984	0.0073	0.0088	0.0049
Getting up in morning	0	-0.084	0.062	-1.362	0.173	0.674	0.068	0.003	1.0401	0.0104	-5.00E-04	0.0057
Glucose	27005778	0.082	0.160	0.513	0.608	0.980	0.087	0.021	0.9953	0.0064	-1.00E-04	0.0048
Glutamine	27005778	0.307	0.182	1.693	0.091	0.525	0.063	0.020	1.0177	0.0076	-0.0041	0.0046
Glycoprotein acetyls; mainly a1-acid glycoprotein	27005778	0.196	0.159	1.229	0.219	0.740	0.099	0.029	0.9861	0.0062	-0.0031	0.0046
Guilty feelings	0	0.065	0.077	0.843	0.399	0.895	0.051	0.003	1.0078	0.0085	0.0045	0.0053
Had major operations	0	0.030	0.114	0.264	0.792	0.985	0.025	0.004	1.006	0.0074	-0.0022	0.0047
Had menopause	0	0.007	0.155	0.045	0.965	0.995	0.020	0.004	1.0049	0.0069	0.0025	0.0053
Had other major operations	0	0.158	0.112	1.409	0.159	0.645	0.031	0.003	1.0095	0.0069	-0.0066	0.0052
Hair/balding pattern: Pattern 2	0	0.135	0.090	1.501	0.133	0.611	0.052	0.007	1.0347	0.0134	-0.0057	0.0049
Hair/balding pattern: Pattern 3	0	-0.044	0.094	-0.469	0.639	0.980	0.056	0.014	1.0206	0.0192	6.00E-04	0.0052
Hair/balding pattern: Pattern 4	0	-0.007	0.066	-0.109	0.913	0.986	0.168	0.020	1.0976	0.0242	0.0036	0.0062
Hand grip strength (left)	0	-0.001	0.053	-0.011	0.992	0.995	0.104	0.004	1.0551	0.0124	-0.0014	0.0059
Hand grip strength (right)	0	-0.018	0.054	-0.330	0.742	0.985	0.104	0.004	1.061	0.0134	0.0037	0.0058
Handedness (chirality/laterality): Left-handed	0	0.049	0.125	0.392	0.695	0.985	0.010	0.002	1.0055	0.0071	-0.0019	0.0049
Handedness (chirality/laterality): Use both right and left hands equally	0	-0.215	0.176	-1.217	0.224	0.740	0.004	0.001	1.0062	0.0066	0.0068	0.0048
Happiness	0	-0.101	0.090	-1.114	0.265	0.778	0.062	0.005	1.0047	0.0075	0.0102	0.0046
HbA1C	20858683	-0.013	0.124	-0.106	0.916	0.986	0.065	0.012	0.9993	0.0071	0.0068	0.0048
HDL cholesterol	20686565	-0.062	0.078	-0.795	0.427	0.911	0.128	0.023	1.0579	0.0492	5.00E-04	0.0053
Headaches for 3+ months	0	-0.145	0.120	-1.200	0.230	0.741	0.056	0.007	1.0068	0.0065	6.00E-04	0.0047
Health satisfaction	0	0.120	0.086	1.388	0.165	0.659	0.083	0.005	1.0067	0.0076	0.0014	0.0051
Hearing aid user	0	-0.137	0.131	-1.039	0.299	0.802	0.017	0.003	1.0052	0.0067	8.00E-04	0.005
Hearing difficulty/problems with background noise	0	-0.147	0.070	-2.092	0.037	0.433	0.052	0.003	1.02	0.0082	0.0129	0.0053
Hearing difficulty/problems: Yes	0	-0.138	0.071	-1.948	0.051	0.444	0.042	0.002	1.0144	0.009	0.0118	0.0047
Heart rate	23583979	0.068	0.093	0.729	0.466	0.918	0.084	0.009	1.0123	0.0085	-0.0038	0.0053
Heel bone mineral density (BMD) T-score_ automated	0	0.123	0.063	1.933	0.053	0.454	0.294	0.034	1.0857	0.0347	-0.0057	0.0073
Heel bone mineral density (BMD) T-score_ automated (left)	0	0.125	0.072	1.746	0.081	0.505	0.300	0.036	1.0327	0.0214	-0.0106	0.0062
Heel bone mineral density (BMD) T-score_ automated (right)	0	0.151	0.073	2.069	0.039	0.433	0.302	0.037	1.0317	0.0224	-0.0124	0.0065
Height_2010	20881960	-0.018	0.052	-0.352	0.725	0.985	0.285	0.016	1.0187	0.0186	-0.0134	0.0056
Height; Females at age 10 and males at age 12	23449627	-0.101	0.091	-1.108	0.268	0.780	0.431	0.048	0.9523	0.008	0.0062	0.0049
Hip circumference	25673412	0.062	0.067	0.924	0.355	0.849	0.127	0.006	0.8608	0.0079	-0.0084	0.0048
Hip circumference	0	0.005	0.050	0.107	0.915	0.986	0.218	0.009	1.0804	0.0188	-0.0045	0.0072
HOMA-B	20081858	-0.071	0.143	-0.497	0.619	0.980	0.090	0.013	0.9891	0.0063	-0.0034	0.0047
HOMA-IR	20081858	-0.023	0.158	-0.143	0.887	0.986	0.068	0.012	1.0032	0.0064	-0.0028	0.0051
Home area population density - urban or rural: England/Wales - Village - less sparse	0	0.032	0.256	0.125	0.900	0.986	0.002	0.001	1.0245	0.0064	-0.0045	0.005
Home area population density - urban or rural: Scotland - Large Urban Area	0	-0.456	0.160	-2.850	0.004	0.118	0.011	0.003	2.1366	0.0147	-0.0123	0.0066
ICV	25607358	0.035	0.160	0.219	0.827	0.985	0.175	0.045	1.0022	0.0067	0.0029	0.0049
Illness_ injury_ bereavement_ stress in last 2 years: Death of a close relative	0	0.112	0.140	0.804	0.422	0.910	0.006	0.002	1.0039	0.0062	0.0051	0.0048

Illness_ injury_ bereavement_ stress in last 2 years: Financial difficulties	0	0.151	0.075	2.014	0.044	0.435	0.033	0.002	1.0275	0.008	-0.0024	0.005
Illness_ injury_ bereavement_ stress in last 2 years: Marital separation/divorce	0	0.038	0.168	0.224	0.823	0.985	0.004	0.002	1.0098	0.0068	0.0026	0.0042
Illness_ injury_ bereavement_ stress in last 2 years: None of the above	0	-0.018	0.088	-0.199	0.842	0.985	0.023	0.002	1.0117	0.0059	-0.0041	0.005
llness_ injury_ bereavement_ stress in last 2 years: Serious illness_ injury or assault of a close relativ	0	-0.089	0.133	-0.672	0.501	0.944	0.011	0.002	1.0103	0.007	-0.0055	0.0049
Illness_ injury_ bereavement_ stress in last 2 years: Serious illness_ injury or assault to yourself	0	-0.137	0.105	-1.295	0.195	0.704	0.014	0.002	1.0115	0.0069	0.0104	0.0047
Illnesses of father: Chronic bronchitis/emphysema	0	0.286	0.107	2.682	0.007	0.161	0.015	0.002	1.0239	0.0069	-0.0035	0.0049
Illnesses of father: Diabetes	0	0.103	0.109	0.943	0.346	0.844	0.018	0.002	1.027	0.0076	-0.002	0.0049
Illnesses of father: Heart disease	0	-0.105	0.082	-1.287	0.198	0.706	0.031	0.003	1.0008	0.0087	0.0075	0.0048
Illnesses of father: High blood pressure	0	0.055	0.107	0.512	0.609	0.980	0.019	0.002	1.0139	0.007	0.0015	0.0052
Illnesses of father: Lung cancer	0	0.190	0.111	1.718	0.086	0.511	0.015	0.002	0.9928	0.0072	-0.0025	0.0047
Illnesses of father: None of the above (group 1)	0	-0.047	0.116	-0.400	0.689	0.985	0.015	0.002	1.0234	0.0068	-0.0047	0.005
Illnesses of father: None of the above (group 2)	0	-0.211	0.144	-1.466	0.143	0.612	0.009	0.002	1.0015	0.0072	4.00E-04	0.005
Illnesses of father: Prostate cancer	0	0.203	0.157	1.293	0.196	0.704	0.008	0.002	1.0122	0.0078	-0.0033	0.0048
Illnesses of mother: Alzheimers disease/dementia	0	0.161	0.213	0.753	0.452	0.918	0.004	0.005	1.0342	0.034	-0.0022	0.005
Illnesses of mother: Breast cancer	0	-0.081	0.153	-0.533	0.594	0.980	0.009	0.002	1.0075	0.008	-0.003	0.0049
Illnesses of mother: Chronic bronchitis/emphysema	0	0.208	0.106	1.966	0.049	0.444	0.013	0.002	1.0038	0.0067	-0.0039	0.0051
Illnesses of mother: Diabetes	0	0.007	0.090	0.074	0.941	0.990	0.021	0.002	1.0161	0.0091	-0.0016	0.0047
Illnesses of mother: Heart disease	0	-0.145	0.106	-1.370	0.171	0.671	0.016	0.002	1.011	0.0068	0.009	0.0048
Illnesses of mother: High blood pressure	0	-0.080	0.086	-0.934	0.350	0.849	0.029	0.002	1.0184	0.0086	0.0058	0.0053
Illnesses of mother: None of the above (group 1)	0	0.123	0.108	1.140	0.254	0.772	0.017	0.002	1.0131	0.0073	-0.0031	0.005
Illnesses of mother: None of the above (group 2)	0	-0.210	0.128	-1.639	0.101	0.553	0.009	0.002	1.0009	0.0065	0.0096	0.0046
Illnesses of siblings: Diabetes	0	0.025	0.100	0.253	0.800	0.985	0.021	0.002	1.0098	0.0068	0.0036	0.0051
Illnesses of siblings: Heart disease	0	-0.024	0.106	-0.225	0.822	0.985	0.017	0.002	1.012	0.007	0.0049	0.005
Illnesses of siblings: High blood pressure	0	-0.032	0.081	-0.390	0.696	0.985	0.037	0.003	1.0067	0.0081	0.002	0.0056
Illnesses of siblings: None of the above (group 1)	0	0.043	0.079	0.540	0.589	0.980	0.036	0.003	1.0136	0.0077	-0.0052	0.0052
Illnesses of siblings: None of the above (group 2)	0	-0.263	0.152	-1.724	0.085	0.511	0.009	0.002	1.0034	0.0068	2.00E-04	0.0044
Illnesses of siblings: Severe depression	0	0.013	0.117	0.114	0.910	0.986	0.014	0.002	1.0055	0.0064	0.004	0.0043
Illnesses of siblings: Stroke	0	-0.068	0.188	-0.363	0.717	0.985	0.004	0.002	1.008	0.0064	0.0063	0.0047
Impedance of arm (left)	0	-0.053	0.053	-1.009	0.313	0.813	0.233	0.008	1.0979	0.0213	-0.0025	0.0075
Impedance of arm (right)	0	-0.051	0.053	-0.963	0.336	0.831	0.236	0.008	1.082	0.0219	-0.002	0.0075
Impedance of leg (left)	0	0.006	0.053	0.122	0.903	0.986	0.241	0.009	1.082	0.0219	0.0034	0.0073
Impedance of leg (right)	0	-0.008	0.050	-0.165	0.869	0.986	0.240	0.009	1.0922	0.0218	0.0054	0.0071
Impedance of whole body	0	-0.023	0.053	-0.438	0.662	0.985	0.258	0.009	1.0901	0.0218	6.00E-04	0.0076
Infant head circumference	22504419	0.023	0.158	0.148	0.883	0.986	0.229	0.045	0.9906	0.0066	-0.0025	0.0052
Inflammatory Bowel Disease (Euro)	26192919	0.123	0.085	1.447	0.148	0.628	0.335	0.033	1.0529	0.0099	0.0652	0.005
Insomnia	28604731	0.120	0.100	1.201	0.230	0.741	0.048	0.005	1.003	0.007	-0.0028	0.0048
Insomnia	27992416	-0.075	0.094	-0.797	0.425	0.911	0.133	0.012	1.0025	0.008	0.0013	0.0053
Intelligence	28530673	-0.224	0.072	-3.111	0.002	0.062	0.189	0.010	1.0087	0.0079	-0.0038	0.005
Irritability	0	-0.032	0.078	-0.406	0.685	0.985	0.068	0.004	0.9931	0.0113	0.0054	0.006
Isoleucine	27005778	-0.103	0.163	-0.634	0.526	0.962	0.071	0.024	0.9948	0.0066	0.0023	0.0043
Job involves heavy manual or physical work	0	0.127	0.062	2.056	0.040	0.433	0.087	0.005	1.0361	0.0088	0.0042	0.0047
Job involves mainly walking or standing	0	0.216	0.065	3.324	0.001	0.036	0.080	0.004	1.0309	0.009	0.0028	0.0047
Job involves shift work	0	0.079	0.085	0.930	0.352	0.849	0.031	0.003	1.002	0.0065	-0.0014	0.0045

Knee pain for 3+ months	0	-0.149	0.148	-1.007	0.314	0.813	0.026	0.007	1.0007	0.0065	0.0059	0.0047
LDL cholesterol	20686565	0.015	0.090	0.163	0.870	0.986	0.105	0.028	1.0505	0.0481	0.0034	0.0054
Leg fat mass (left)	0	0.079	0.053	1.507	0.132	0.611	0.232	0.008	1.0713	0.0173	-0.0037	0.0077
Leg fat mass (right)	0	0.071	0.053	1.336	0.182	0.689	0.231	0.008	1.0708	0.0174	-0.0028	0.0078
Leg fat percentage (left)	0	0.104	0.052	2.022	0.043	0.433	0.208	0.006	1.0847	0.0162	-0.003	0.0075
Leg fat percentage (right)	0	0.090	0.052	1.749	0.080	0.505	0.208	0.006	1.0819	0.0166	-0.0014	0.0075
Leg fat-free mass (left)	0	0.012	0.047	0.255	0.799	0.985	0.267	0.011	1.1064	0.025	-0.0055	0.0072
Leg fat-free mass (right)	0	0.011	0.045	0.244	0.807	0.985	0.264	0.011	1.1135	0.0256	-0.0063	0.007
Leg pain on walking	0	0.109	0.099	1.105	0.269	0.781	0.057	0.005	1.0103	0.0067	-6.00E-04	0.0052
Leg predicted mass (left)	0	0.009	0.047	0.185	0.854	0.985	0.266	0.011	1.1062	0.0249	-0.005	0.0073
Leg predicted mass (right)	0	0.008	0.045	0.186	0.853	0.985	0.264	0.011	1.1132	0.0256	-0.0059	0.007
Length of menstrual cycle	0	-0.099	0.126	-0.784	0.433	0.918	0.103	0.021	0.9989	0.0069	0.0026	0.005
Length of working week for main job	0	0.111	0.136	0.818	0.414	0.903	0.019	0.003	1.0086	0.0066	-0.0038	0.0053
Leptin_adjBMI	26833098	0.030	0.131	0.230	0.819	0.985	0.095	0.018	1.0034	0.0069	0.0023	0.0049
Leptin_not_adjBMI	26833098	0.072	0.124	0.582	0.560	0.980	0.099	0.016	0.9941	0.0065	0.004	0.0048
Leucine	27005778	-0.193	0.210	-0.921	0.357	0.849	0.046	0.021	1.0091	0.0067	0.0051	0.0047
Light smokers_ at least 100 smokes in lifetime	0	0.427	0.100	4.292	0.000	0.002	0.077	0.008	1.005	0.0075	-0.0038	0.005
Loneliness_ isolation	0	0.059	0.067	0.891	0.373	0.867	0.038	0.002	1.0227	0.0077	0.0046	0.0043
Long-standing illness_ disability or infirmity	0	-0.017	0.059	-0.286	0.775	0.985	0.050	0.002	1.027	0.0087	0.0049	0.0047
Loud music exposure frequency	0	0.273	0.134	2.031	0.042	0.433	0.029	0.005	1.0131	0.0069	-0.0033	0.0047
Lumbar Spine bone mineral density	26367794	-0.157	0.088	-1.775	0.076	0.505	0.134	0.015	0.9752	0.008	0.008	0.0046
Lumbar spine bone mineral density	22504420	0.003	0.078	0.033	0.974	0.995	0.271	0.025	1.0125	0.0086	5.00E-04	0.0045
Lung adenocarcinoma	27488534	0.169	0.161	1.049	0.294	0.801	0.033	0.011	1.0182	0.0063	0.0195	0.0039
Lung cancer	27488534	0.222	0.109	2.039	0.042	0.433	0.316	0.066	1.0144	0.0073	0.0358	0.0044
Lung cancer (all)	24880342	0.185	0.119	1.548	0.122	0.602	0.128	0.029	1.0079	0.008	0.0414	0.0046
Lung cancer (squamous cell)	24880342	0.097	0.177	0.546	0.585	0.980	0.051	0.019	1.0063	0.0067	0.029	0.0045
Major depressive disorder	22472876	0.122	0.116	1.057	0.291	0.796	0.172	0.026	1.005	0.006	0.0129	0.0047
Maternal smoking around birth	0	0.167	0.069	2.409	0.016	0.268	0.050	0.003	1.0362	0.0088	0.0048	0.0053
Maximum heart rate during fitness test	0	-0.050	0.150	-0.332	0.740	0.985	0.068	0.013	1.0163	0.0087	0.0033	0.0056
Maximum workload during fitness test	0	0.050	0.140	0.357	0.721	0.985	0.043	0.010	1.0075	0.007	-0.003	0.0046
Mean Accumbens	25607358	-0.055	0.225	-0.246	0.806	0.985	0.091	0.036	0.9791	0.0064	0.0052	0.005
Mean Caudate	25607358	-0.032	0.116	-0.272	0.786	0.985	0.250	0.039	0.9684	0.006	0.0024	0.0044
Mean diameter for HDL particles	27005778	0.103	0.143	0.725	0.469	0.918	0.118	0.030	1.0086	0.0187	0.0011	0.0041
Mean diameter for LDL particles	27005778	0.024	0.214	0.111	0.911	0.986	0.047	0.026	1.0004	0.0122	0.002	0.0044
Mean diameter for VLDL particles	27005778	0.069	0.134	0.514	0.608	0.980	0.134	0.035	0.9904	0.0085	-0.0035	0.0042
Mean Hippocampus	25607358	-0.004	0.176	-0.020	0.984	0.995	0.141	0.039	0.9873	0.0065	6.00E-04	0.005
Mean Pallidum	25607358	-0.346	0.161	-2.148	0.032	0.425	0.160	0.041	0.978	0.0069	0.0089	0.005
Mean platelet volume	22139419	0.023	0.103	0.225	0.822	0.985	0.319	0.051	0.9816	0.0108	4.00E-04	0.0055
Mean Putamen	25607358	0.002	0.108	0.015	0.988	0.995	0.299	0.046	0.95	0.0068	0.0055	0.0046
Mean Thalamus	25607358	-0.072	0.163	-0.440	0.660	0.985	0.139	0.036	0.9794	0.0063	0.004	0.0049
Mean time to correctly identify matches	0	-0.033	0.059	-0.559	0.576	0.980	0.073	0.003	1.0304	0.0094	0.0065	0.0052
Medication for cholesterol_ blood pressure or diabetes: Blood pressure medication	0	0.010	0.078	0.124	0.902	0.986	0.105	0.006	1.0316	0.0102	0.0078	0.0058
Medication for cholesterol_ blood pressure or diabetes: Cholesterol lowering medication	0	-0.042	0.083	-0.503	0.615	0.980	0.068	0.006	1.0328	0.0142	0.0097	0.0051

Medication for cholesterol_ blood pressure or diabetes: Insulin	0	-0.261	0.176	-1.487	0.137	0.611	0.010	0.003	1.0109	0.007	0.009	0.0046
Medication for cholesterol_ blood pressure or diabetes: None of the above	0	-0.023	0.079	-0.289	0.773	0.985	0.089	0.005	1.0325	0.0102	-0.0069	0.0056
ation for cholesterol_ blood pressure_ diabetes_ or take exogenous hormones: Blood pressure medi	0	-0.001	0.074	-0.010	0.992	0.995	0.105	0.007	1.033	0.012	-6.00E-04	0.006
on for cholesterol_ blood pressure_ diabetes_ or take exogenous hormones: Cholesterol lowering m	0	0.050	0.085	0.587	0.557	0.980	0.051	0.006	1.0247	0.0137	0.0031	0.0051
ion for cholesterol_ blood pressure_ diabetes_ or take exogenous hormones: Hormone replacement	0	0.264	0.138	1.905	0.057	0.458	0.016	0.003	1.001	0.0061	-0.0025	0.0052
edication for cholesterol_ blood pressure_ diabetes_ or take exogenous hormones: None of the abo	0	-0.010	0.078	-0.121	0.904	0.986	0.066	0.004	1.0185	0.0086	-0.0027	0.0055
Medication for pain relief_ constipation_ heartburn: Aspirin	0	0.105	0.096	1.084	0.279	0.783	0.021	0.002	1.0136	0.007	-0.0027	0.0057
Medication for pain relief_ constipation_ heartburn: Ibuprofen (e.g. Nurofen)	0	-0.008	0.089	-0.091	0.928	0.986	0.023	0.002	0.9976	0.0063	-0.004	0.0047
Medication for pain relief_ constipation_ heartburn: Laxatives (e.g. Dulcolax_ Senokot)	0	0.216	0.105	2.055	0.040	0.433	0.012	0.002	1.0093	0.0068	-0.0059	0.0046
Medication for pain relief_ constipation_ heartburn: None of the above	0	-0.027	0.069	-0.398	0.690	0.985	0.054	0.003	1.0295	0.0088	-0.0036	0.0057
Medication for pain relief_ constipation_ heartburn: Omeprazole (e.g. Zanprol)	0	-0.045	0.089	-0.502	0.616	0.980	0.018	0.002	1.0197	0.0071	0.0107	0.0053
Medication for pain relief_ constipation_ heartburn: Paracetamol	0	0.008	0.071	0.118	0.906	0.986	0.043	0.002	1.0266	0.0083	0.0058	0.0055
Mineral and other dietary supplements: Calcium	0	0.063	0.109	0.576	0.565	0.980	0.012	0.002	0.9979	0.0063	-0.0056	0.0047
Mineral and other dietary supplements: Fish oil (including cod liver oil)	0	0.003	0.092	0.028	0.977	0.995	0.027	0.002	1.006	0.0071	-0.0031	0.0052
Mineral and other dietary supplements: Glucosamine	0	-0.062	0.085	-0.726	0.468	0.918	0.024	0.002	1.0088	0.0074	0.0021	0.0051
Mineral and other dietary supplements: None of the above	0	-0.017	0.084	-0.205	0.838	0.985	0.030	0.002	1.0099	0.0067	0.0014	0.005
Mineral and other dietary supplements: Selenium	0	0.097	0.161	0.602	0.547	0.976	0.006	0.002	1.0002	0.0071	-0.0039	0.005
Mineral and other dietary supplements: Zinc	0	0.051	0.121	0.419	0.675	0.985	0.011	0.001	0.9896	0.0058	-0.0031	0.0045
Miserableness	0	0.013	0.069	0.191	0.849	0.985	0.064	0.004	1.0177	0.011	0.0068	0.0053
Mono-unsaturated fatty acids	27005778	0.097	0.186	0.521	0.602	0.980	0.104	0.042	0.9884	0.0078	-0.0011	0.0047
Mood swings	0	0.104	0.061	1.720	0.086	0.511	0.071	0.003	1.0174	0.0098	4.00E-04	0.0056
Morning/evening person (chronotype)	0	-0.015	0.060	-0.251	0.802	0.985	0.116	0.004	1.0412	0.0112	0.0045	0.006
Most recent bowel cancer screening	0	0.143	0.231	0.619	0.536	0.974	0.012	0.006	1.0119	0.0059	-0.0077	0.0047
Mothers age at death	27015805	-0.116	0.151	-0.769	0.442	0.918	0.036	0.008	1.0131	0.0072	-0.0015	0.005
Mothers age at death	0	-0.083	0.122	-0.681	0.496	0.943	0.024	0.003	1.0294	0.008	-0.0046	0.0054
Mouth/teeth dental problems: Bleeding gums	0	0.054	0.102	0.526	0.599	0.980	0.022	0.002	1.0118	0.008	-0.0094	0.0059
Mouth/teeth dental problems: Dentures	0	0.137	0.075	1.820	0.069	0.505	0.048	0.003	1.036	0.0094	-0.0048	0.006
Mouth/teeth dental problems: Loose teeth	0	0.154	0.112	1.374	0.169	0.671	0.015	0.002	1.011	0.007	-0.0034	0.0054
Mouth/teeth dental problems: Mouth ulcers	0	-0.153	0.094	-1.625	0.104	0.555	0.029	0.004	1.032	0.011	0.007	0.0051
Mouth/teeth dental problems: None of the above	0	-0.123	0.081	-1.517	0.129	0.606	0.038	0.002	1.0185	0.008	0.007	0.0056
Mouth/teeth dental problems: Painful gums	0	-0.030	0.149	-0.198	0.843	0.985	0.007	0.002	1.0045	0.0064	0.0039	0.0049
Mouth/teeth dental problems: Toothache	0	0.088	0.133	0.661	0.509	0.950	0.008	0.002	1.0043	0.0072	-0.0071	0.0049
Multiple sclerosis	21833088	0.208	0.241	0.861	0.389	0.884	0.058	0.028	1.0584	0.0088	0.0377	0.0059
Nap during day	0	0.060	0.055	1.088	0.277	0.783	0.081	0.004	1.0234	0.0096	-0.0014	0.0054
Neck/shoulder pain for 3+ months	0	0.047	0.180	0.262	0.793	0.985	0.021	0.006	1.0083	0.0074	0.0066	0.0046
Neo-conscientiousness	21173776	-0.056	0.222	-0.252	0.801	0.985	0.073	0.030	1.0004	0.0068	0.0036	0.0053
Neo-openness to experience	21173776	0.103	0.161	0.642	0.521	0.956	0.106	0.027	0.9918	0.0066	-0.0084	0.005
Nervous feelings	0	-0.001	0.077	-0.006	0.995	0.995	0.066	0.004	1.0146	0.0122	0.0126	0.0055
Neuroticism	24828478	0.162	0.144	1.125	0.261	0.772	0.015	0.003	1.0078	0.0061	-0.0037	0.005
Neuroticism	27089181	0.040	0.087	0.455	0.650	0.985	0.089	0.007	0.9902	0.0117	0.0075	0.0055
Neuroticism score	0	0.069	0.067	1.023	0.307	0.808	0.118	0.006	1.0178	0.0134	0.0042	0.0058
No-wear time bias adjusted acceleration standard deviation	0	-0.077	0.089	-0.865	0.387	0.881	0.089	0.008	1.0011	0.0075	0.0043	0.0044
Noisy workplace	0	0.044	0.092	0.485	0.627	0.980	0.062	0.006	1.0286	0.0072	0.0066	0.0049

Non-accidental death in close genetic family	0	-0.124	0.174	-0.716	0.474	0.921	0.018	0.005	0.9953	0.0065	-0.003	0.0049
Non-cancer illness code_ self-reported: allergy or anaphylactic reaction to drug	0	0.204	0.399	0.512	0.608	0.980	0.001	0.001	1.0079	0.0061	-0.0043	0.0046
Non-cancer illness code_ self-reported: angina	0	-0.029	0.090	-0.324	0.746	0.985	0.023	0.002	1.0215	0.0077	0.0122	0.0047
Non-cancer illness code_ self-reported: ankylosing spondylitis	0	-0.219	0.305	-0.718	0.473	0.921	0.002	0.001	1.003	0.0064	0.0036	0.0048
Non-cancer illness code_ self-reported: anxiety/panic attacks	0	0.072	0.153	0.471	0.638	0.980	0.006	0.001	0.9999	0.006	0.0041	0.005
Non-cancer illness code_ self-reported: arthritis (nos)	0	-0.163	0.285	-0.572	0.568	0.980	0.002	0.001	1.0154	0.0065	0.0065	0.0049
Non-cancer illness code_ self-reported: asthma	0	0.008	0.065	0.127	0.899	0.986	0.058	0.006	1.0274	0.0148	-0.0014	0.0048
Non-cancer illness code_ self-reported: back problem	0	-0.054	0.167	-0.326	0.745	0.985	0.005	0.001	1.0058	0.0063	0.0038	0.0049
Non-cancer illness code_ self-reported: bladder problem (not cancer)	0	0.453	0.296	1.534	0.125	0.602	0.002	0.001	1.002	0.0071	-0.0107	0.0048
Non-cancer illness code_ self-reported: bone disorder	0	0.076	0.283	0.268	0.789	0.985	0.002	0.001	1.0036	0.006	0.0012	0.0049
Non-cancer illness code_ self-reported: cholelithiasis/gall stones	0	0.036	0.157	0.226	0.821	0.985	0.008	0.002	1.0211	0.0079	-0.0015	0.0052
Non-cancer illness code_ self-reported: chronic obstructive airways disease/copd	0	-0.125	0.164	-0.763	0.446	0.918	0.006	0.002	0.9926	0.007	0.0015	0.005
Non-cancer illness code_ self-reported: crohns disease	0	-0.032	0.172	-0.188	0.851	0.985	0.005	0.002	1.0056	0.0068	-0.0019	0.0048
Non-cancer illness code_ self-reported: deep venous thrombosis (dvt)	0	0.113	0.133	0.848	0.396	0.892	0.011	0.002	1.0096	0.0093	-0.007	0.0052
Non-cancer illness code_ self-reported: depression	0	0.040	0.097	0.415	0.678	0.985	0.019	0.002	0.9998	0.0072	0.0046	0.0048
Non-cancer illness code_ self-reported: diabetes	0	-0.059	0.074	-0.797	0.426	0.911	0.036	0.003	1.0381	0.0103	0.0093	0.005
Non-cancer illness code_ self-reported: diverticular disease/diverticulitis	0	0.105	0.110	0.959	0.338	0.831	0.013	0.002	0.9943	0.0069	-0.0046	0.0042
Non-cancer illness code_ self-reported: eczema/dermatitis	0	0.024	0.129	0.187	0.851	0.985	0.008	0.002	1.0148	0.0072	-7.00E-04	0.0047
Non-cancer illness code_ self-reported: emphysema/chronic bronchitis	0	0.214	0.133	1.610	0.107	0.560	0.010	0.002	0.9876	0.007	0.0032	0.0049
Non-cancer illness code_ self-reported: enlarged prostate	0	0.366	0.160	2.283	0.023	0.345	0.006	0.002	1.0034	0.0065	-0.0128	0.0051
Non-cancer illness code_ self-reported: gastro-oesophageal reflux (gord) / gastric reflux	0	0.041	0.114	0.357	0.721	0.985	0.011	0.002	1.0142	0.0072	0.0032	0.0047
Non-cancer illness code_ self-reported: glaucoma	0	0.019	0.130	0.143	0.887	0.986	0.011	0.002	1.0147	0.0079	-0.0055	0.0054
Non-cancer illness code_ self-reported: gout	0	-0.055	0.089	-0.624	0.533	0.971	0.025	0.007	0.9928	0.0188	0.002	0.0052
Non-cancer illness code_ self-reported: hayfever/allergic rhinitis	0	-0.139	0.095	-1.464	0.143	0.612	0.023	0.002	1.0184	0.0085	0.0042	0.0052
Non-cancer illness code_ self-reported: heart attack/myocardial infarction	0	-0.110	0.090	-1.226	0.220	0.740	0.019	0.002	1.0104	0.0073	0.0087	0.0043
Non-cancer illness code_ self-reported: hiatus hernia	0	-0.108	0.131	-0.824	0.410	0.903	0.008	0.001	1.0325	0.0064	4.00E-04	0.0046
Non-cancer illness code_ self-reported: high cholesterol	0	0.025	0.077	0.323	0.746	0.985	0.040	0.005	1.0563	0.0274	0.0068	0.0054
Non-cancer illness code_ self-reported: hypertension	0	0.035	0.061	0.569	0.569	0.980	0.115	0.005	1.0565	0.017	0.0066	0.0065
Non-cancer illness code_ self-reported: hyperthyroidism/thyrotoxicosis	0	-0.033	0.136	-0.245	0.807	0.985	0.009	0.003	1.0047	0.0077	0.0027	0.0046
Non-cancer illness code_ self-reported: hypertrophic cardiomyopathy (hcm / hocm)	0	0.719	0.408	1.762	0.078	0.505	0.002	0.001	1.0017	0.0066	-0.0038	0.005
Non-cancer illness code_ self-reported: hypopituitarism	0	-0.293	0.334	-0.877	0.380	0.868	0.002	0.001	0.9906	0.0061	0.012	0.0047
Non-cancer illness code_ self-reported: hypothyroidism/myxoedema	0	-0.134	0.094	-1.430	0.153	0.631	0.051	0.006	1.0312	0.0152	0.0121	0.0063
Non-cancer illness code_ self-reported: iron deficiency anaemia	0	-0.216	0.157	-1.372	0.170	0.671	0.005	0.001	0.9898	0.006	0.0015	0.0044
Non-cancer illness code_ self-reported: joint disorder	0	0.033	0.237	0.139	0.889	0.986	0.002	0.001	0.9912	0.0063	0.001	0.0047
Non-cancer illness code_ self-reported: kidney stone/ureter stone/bladder stone	0	0.123	0.126	0.977	0.329	0.828	0.009	0.002	1.0029	0.0077	-0.0072	0.0046
Non-cancer illness code_ self-reported: malabsorption/coeliac disease	0	-0.044	0.126	-0.346	0.730	0.985	0.017	0.012	0.9956	0.0205	-0.0024	0.0047
Non-cancer illness code_ self-reported: mania/bipolar disorder/manic depression	0	-0.229	0.154	-1.483	0.138	0.611	0.006	0.001	1.0007	0.0061	0.0073	0.0047
Non-cancer illness code_ self-reported: migraine	0	0.001	0.098	0.010	0.992	0.995	0.017	0.002	1.0121	0.0074	-0.0052	0.0046
Non-cancer illness code_ self-reported: muscle or soft tissue injuries	0	0.448	0.270	1.659	0.097	0.544	0.002	0.001	1.0001	0.0071	-0.0092	0.0047
Non-cancer illness code_ self-reported: nasal polyps	0	-0.006	0.148	-0.041	0.968	0.995	0.005	0.002	1.0069	0.0065	0.0014	0.0044
Non-cancer illness code_ self-reported: osteoarthritis	0	0.101	0.096	1.051	0.293	0.801	0.019	0.002	1.0282	0.0076	-0.0069	0.0054
Non-cancer illness code_ self-reported: osteoporosis	0	0.002	0.113	0.019	0.985	0.995	0.015	0.002	1.0002	0.0075	-0.0031	0.0053
Non-cancer illness code_ self-reported: pernicious anaemia	0	-0.435	0.213	-2.043	0.041	0.433	0.004	0.001	0.9916	0.0064	0.0088	0.0051

Non-cancer illness code_ self-reported: pneumothorax	0	-0.057	0.211	-0.273	0.785	0.985	0.003	0.001	0.9885	0.007	9.04E-05	0.0048
Non-cancer illness code_ self-reported: polio / poliomyelitis	0	0.134	0.221	0.606	0.545	0.975	0.003	0.001	0.9923	0.0065	7.00E-04	0.0047
Non-cancer illness code_ self-reported: psoriasis	0	-0.023	0.164	-0.141	0.888	0.986	0.007	0.002	1.0178	0.0082	0.0015	0.0047
Non-cancer illness code_ self-reported: pulmonary embolism +/- dvt	0	0.162	0.151	1.073	0.284	0.789	0.006	0.002	1.0076	0.0075	-0.0014	0.0049
Non-cancer illness code_ self-reported: retinal detachment	0	0.076	0.211	0.360	0.719	0.985	0.003	0.001	1.003	0.0066	0.0017	0.0047
Non-cancer illness code_ self-reported: rheumatoid arthritis	0	-0.025	0.173	-0.147	0.883	0.986	0.005	0.001	1.0044	0.0063	0.0135	0.0047
Non-cancer illness code_ self-reported: sleep apnoea	0	0.120	0.196	0.611	0.541	0.975	0.003	0.001	1.0036	0.0069	-0.0013	0.0047
Non-cancer illness code_ self-reported: type 2 diabetes	0	-0.067	0.161	-0.413	0.679	0.985	0.006	0.001	1.0111	0.0063	0.0035	0.0051
Non-cancer illness code_ self-reported: ulcerative colitis	0	-0.257	0.168	-1.536	0.125	0.602	0.006	0.002	1.0111	0.0079	-0.0028	0.0045
Non-cancer illness code_ self-reported: uterine fibroids	0	-0.041	0.151	-0.274	0.784	0.985	0.007	0.002	1.0076	0.0068	0.0051	0.0051
Non-cancer illness code_ self-reported: vaginal prolapse/uterine prolapse	0	-0.106	0.193	-0.550	0.583	0.980	0.005	0.002	1.0063	0.0067	0.0076	0.0052
Non-cancer illness code_ self-reported: varicose veins	0	0.062	0.167	0.374	0.709	0.985	0.004	0.001	1.0111	0.0064	0.0014	0.0045
Non-cancer illness code_ self-reported: vitiligo	0	0.300	0.323	0.928	0.354	0.849	0.002	0.001	0.9933	0.0071	-0.0014	0.0042
Number of children ever born	27798627	0.083	0.079	1.044	0.297	0.801	0.026	0.002	0.9689	0.0069	0.0013	0.0048
Number of children fathered	0	0.063	0.104	0.605	0.545	0.975	0.032	0.004	1.0111	0.0072	-0.0034	0.0049
Number of cigarettes currently smoked daily (current cigarette smokers)	0	-0.075	0.158	-0.474	0.636	0.980	0.103	0.026	1.0009	0.0069	-0.0067	0.0054
Number of cigarettes previously smoked daily	0	0.057	0.084	0.678	0.498	0.944	0.101	0.014	1.0016	0.009	-0.0073	0.0051
Number of days/week of moderate physical activity 10+ minutes	0	0.092	0.076	1.204	0.229	0.741	0.042	0.003	1.0124	0.008	-0.0017	0.0046
Number of days/week of vigorous physical activity 10+ minutes	0	0.144	0.079	1.823	0.068	0.505	0.038	0.002	1.0155	0.0084	-0.0031	0.0049
Number of days/week walked 10+ minutes	0	-0.012	0.067	-0.181	0.857	0.985	0.042	0.002	1.0097	0.0075	0.0041	0.0049
Number of depression episodes	0	0.030	0.212	0.142	0.887	0.986	0.032	0.012	1.0047	0.0064	-0.002	0.0049
Number of full brothers	0	0.210	0.105	1.997	0.046	0.444	0.019	0.002	1.0282	0.0074	-0.0065	0.0049
Number of full sisters	0	-0.027	0.102	-0.261	0.795	0.985	0.019	0.002	1.0395	0.0066	1.00E-04	0.0049
Number of incorrect matches in round	0	0.229	0.060	3.804	0.000	0.007	0.055	0.003	1.0039	0.009	-0.0032	0.005
Number of live births	0	0.078	0.073	1.062	0.288	0.793	0.062	0.004	1.0073	0.008	0.0044	0.005
Number of older siblings	0	0.113	0.310	0.365	0.715	0.985	0.006	0.005	1.0064	0.006	-8.00E-04	0.0051
Number of operations_ self-reported	0	0.036	0.077	0.470	0.638	0.980	0.038	0.002	1.0354	0.0077	9.39E-05	0.0055
Number of pregnancy terminations	0	0.223	0.126	1.769	0.077	0.505	0.060	0.010	1.0077	0.0073	-0.0016	0.005
Number of self-reported cancers	0	0.090	0.150	0.605	0.546	0.975	0.007	0.002	1.0135	0.0064	0.005	0.0047
Number of self-reported non-cancer illnesses	0	0.029	0.061	0.478	0.633	0.980	0.061	0.003	1.0263	0.0102	-2.00E-04	0.0052
Number of treatments/medications taken	0	0.037	0.065	0.571	0.568	0.980	0.061	0.003	1.0319	0.009	-0.0012	0.0053
Number of trend entries	0	-0.130	0.172	-0.758	0.449	0.918	0.031	0.009	1.0057	0.0066	6.00E-04	0.005
Number of unsuccessful stop-smoking attempts	0	0.100	0.130	0.765	0.445	0.918	0.050	0.007	1.0066	0.0069	-7.00E-04	0.0053
Obesity class 1	23563607	0.086	0.070	1.230	0.219	0.740	0.217	0.012	1.018	0.0109	-0.0101	0.0063
Obesity class 2	23563607	0.028	0.076	0.368	0.713	0.985	0.184	0.012	1.0022	0.0099	-0.0057	0.0061
Obesity class 3	23563607	0.075	0.115	0.652	0.514	0.953	0.122	0.014	0.9797	0.0091	-0.0104	0.0062
Omega-3 fatty acids	27005778	0.049	0.147	0.336	0.737	0.985	0.156	0.042	0.9909	0.0078	0.0035	0.0044
Omega-9 and saturated fatty acids	27005778	0.006	0.210	0.028	0.978	0.995	0.085	0.043	0.9964	0.0078	0.0021	0.0047
Other eye problems	0	0.027	0.122	0.221	0.826	0.985	0.009	0.002	1.0241	0.0071	-4.71E-05	0.0048
Other serious medical condition/disability diagnosed by doctor	0	-0.063	0.077	-0.810	0.418	0.909	0.025	0.002	1.011	0.0072	0.0018	0.0048
Overall health rating	0	0.134	0.054	2.463	0.014	0.253	0.100	0.003	1.0625	0.0106	0.0052	0.0057
Overweight	23563607	0.216	0.075	2.878	0.004	0.111	0.110	0.007	1.0246	0.0095	-0.0172	0.006
Pack years adult smoking as proportion of life span exposed to smoking PREVIEW ONLY	0	0.146	0.081	1.810	0.070	0.505	0.127	0.011	1.0154	0.0092	-0.0078	0.0056

Pack years of smoking PREVIEW ONLY	0	0.144	0.085	1.707	0.088	0.519	0.109	0.010	1.0158	0.0088	-0.0068	0.0057
Pain type(s) experienced in last month: Back pain	0	0.117	0.070	1.665	0.096	0.542	0.039	0.002	1.0247	0.0093	-0.0024	0.0051
Pain type(s) experienced in last month: Facial pain	0	0.106	0.163	0.651	0.515	0.953	0.007	0.001	0.997	0.0061	3.00E-04	0.005
Pain type(s) experienced in last month: Headache	0	-0.099	0.065	-1.524	0.128	0.602	0.045	0.003	1.0047	0.0093	0.0087	0.0047
Pain type(s) experienced in last month: Hip pain	0	0.163	0.085	1.921	0.055	0.456	0.024	0.002	1.0186	0.0077	0.0022	0.0051
Pain type(s) experienced in last month: Knee pain	0	0.122	0.073	1.688	0.092	0.525	0.041	0.002	1.0144	0.0082	-0.0081	0.0053
Pain type(s) experienced in last month: Neck or shoulder pain	0	0.152	0.074	2.065	0.039	0.433	0.033	0.002	1.0251	0.0073	0.0059	0.0047
Pain type(s) experienced in last month: None of the above	0	-0.163	0.060	-2.702	0.007	0.157	0.055	0.003	1.0367	0.0087	-0.0022	0.0046
Pain type(s) experienced in last month: Pain all over the body	0	0.150	0.128	1.171	0.242	0.762	0.010	0.002	1.0037	0.0074	0.0069	0.0051
Pain type(s) experienced in last month: Stomach or abdominal pain	0	0.012	0.093	0.126	0.900	0.986	0.022	0.002	1.0066	0.0071	0.0057	0.0049
Parents age at death	27015805	-0.342	0.172	-1.981	0.048	0.444	0.029	0.007	1.0162	0.0063	0.0052	0.0046
Parkinsons disease	19915575	0.124	0.120	1.035	0.301	0.802	0.408	0.119	1.1237	0.0091	-0.0143	0.0047
Past tobacco smoking	0	-0.125	0.060	-2.089	0.037	0.433	0.091	0.004	1.0208	0.0104	-0.0013	0.0055
Peak expiratory flow (PEF)	0	-0.022	0.062	-0.353	0.724	0.985	0.090	0.005	1.0551	0.0121	-0.0042	0.0057
PGC cross-disorder analysis	23453885	0.040	0.087	0.452	0.652	0.985	0.173	0.013	1.0125	0.0114	0.0325	0.0066
Phenylalanine	27005778	-0.015	0.207	-0.072	0.943	0.990	0.055	0.022	1.0045	0.0069	0.0044	0.0046
Phospholipids in chylomicrons and largest VLDL particles	27005778	0.189	0.145	1.303	0.193	0.702	0.096	0.027	0.9854	0.0067	-9.00E-04	0.0042
Phospholipids in IDL	27005778	-0.019	0.198	-0.097	0.923	0.986	0.061	0.041	1.0211	0.0191	0.0045	0.0046
Phospholipids in large HDL	27005778	0.016	0.139	0.115	0.908	0.986	0.125	0.030	0.9992	0.014	0.0039	0.0042
Phospholipids in large LDL	27005778	0.042	0.208	0.200	0.841	0.985	0.063	0.044	1.0175	0.0211	5.00E-04	0.0048
Phospholipids in large VLDL	27005778	0.107	0.131	0.820	0.412	0.903	0.116	0.030	0.9898	0.0064	-4.00E-04	0.0041
Phospholipids in medium HDL	27005778	0.065	0.169	0.385	0.700	0.985	0.065	0.023	0.9959	0.0077	0.0026	0.0046
Phospholipids in medium LDL	27005778	0.001	0.202	0.007	0.995	0.995	0.073	0.042	1.0085	0.019	-9.00E-04	0.0048
Phospholipids in medium VLDL	27005778	0.038	0.133	0.286	0.775	0.985	0.110	0.032	0.9971	0.0068	3.00E-04	0.004
Phospholipids in small VLDL	27005778	0.051	0.142	0.362	0.717	0.985	0.116	0.034	0.9986	0.0083	-9.00E-04	0.0043
Phospholipids in very large HDL	27005778	0.083	0.161	0.514	0.608	0.980	0.090	0.030	1.0082	0.0166	0.0011	0.0043
Phospholipids in very large VLDL	27005778	0.077	0.137	0.557	0.578	0.980	0.105	0.028	0.9861	0.0068	9.00E-04	0.0043
Phospholipids in very small VLDL	27005778	-0.003	0.169	-0.016	0.987	0.995	0.085	0.038	1.0148	0.0153	0.0022	0.0044
Platelet count	22139419	-0.111	0.082	-1.342	0.180	0.689	0.121	0.011	0.9843	0.008	0.002	0.0048
Potassium in urine	0	-0.184	0.074	-2.500	0.012	0.233	0.043	0.002	1.0174	0.0086	0.0074	0.0053
Primary biliary cirrhosis	26394269	-0.085	0.131	-0.646	0.518	0.956	0.376	0.064	1.0037	0.0098	0.0797	0.0058
Primary sclerosing cholangitis	27992413	-0.007	0.104	-0.067	0.947	0.991	0.390	0.155	0.995	0.0152	0.0339	0.0052
Prospective memory result	0	0.254	0.098	2.582	0.010	0.204	0.058	0.005	1.0109	0.0078	7.92E-06	0.005
Pulse rate	0	0.001	0.074	0.010	0.992	0.995	0.135	0.012	1.0257	0.0143	-0.0016	0.0056
Pulse rate_ automated reading	0	-0.040	0.054	-0.727	0.467	0.918	0.154	0.011	1.0479	0.0285	0.0075	0.0062
Pulse wave Arterial Stiffness index	0	-0.053	0.119	-0.450	0.653	0.985	0.038	0.005	1.0089	0.0072	-0.0013	0.0052
Pulse wave peak to peak time	0	0.030	0.118	0.251	0.802	0.985	0.040	0.005	1.0101	0.0071	0.0013	0.0052
Pulse wave reflection index	0	-0.030	0.103	-0.292	0.770	0.985	0.055	0.005	1.0085	0.0074	-0.0029	0.0047
Qualifications: A levels/AS levels or equivalent	0	-0.255	0.059	-4.313	0.000	0.002	0.097	0.004	1.0534	0.0116	-0.0023	0.0054
Qualifications: College or University degree	0	-0.249	0.053	-4.688	0.000	0.001	0.168	0.005	1.1171	0.0134	-0.0018	0.006
Qualifications: CSEs or equivalent	0	0.217	0.086	2.536	0.011	0.221	0.021	0.002	1.06	0.0072	-8.00E-04	0.005
Qualifications: None of the above	0	0.255	0.059	4.330	0.000	0.002	0.098	0.004	1.0667	0.0116	0.0063	0.0056
Qualifications: NVQ or HND or HNC or equivalent	0	0.128	0.110	1.162	0.245	0.768	0.015	0.002	1.0105	0.0068	3.00E-04	0.005

Qualifications: O levels/GCSEs or equivalent	0	-0.238	0.069	-3.459	0.001	0.027	0.049	0.003	1.0169	0.0093	-0.0074	0.005
Qualifications: Other professional qualifications eg: nursing_ teaching	0	-0.257	0.071	-3.595	0.000	0.017	0.047	0.003	1.026	0.0093	-0.0046	0.0052
Ratio of bisallylic groups to double bonds	27005778	-0.082	0.105	-0.781	0.435	0.918	0.293	0.108	0.978	0.0089	0.0059	0.0045
Ratio of bisallylic groups to total fatty acids	27005778	-0.085	0.114	-0.745	0.456	0.918	0.273	0.097	0.9861	0.0077	0.0051	0.0044
Reason for glasses/contact lenses: For astigmatism	0	-0.146	0.118	-1.240	0.215	0.740	0.009	0.002	1.0264	0.0067	0.0074	0.0047
ason for glasses/contact lenses: For just reading/near work as you are getting older (called presbyop	0	0.143	0.231	0.618	0.537	0.974	0.004	0.002	1.1366	0.008	0.0021	0.0055
ct lenses: For long-sightedness_ i.e. for distance and near_ but particularly for near tasks like readin	0	0.164	0.137	1.198	0.231	0.741	0.010	0.002	1.064	0.0072	0.0048	0.0051
ontact lenses: For short-sightedness_ i.e. only or mainly for distance viewing such as driving_ cinem	0	-0.161	0.082	-1.954	0.051	0.444	0.025	0.002	1.1157	0.0086	0.007	0.0051
Reason for reducing amount of alcohol drunk: Health precaution	0	0.107	0.093	1.155	0.248	0.772	0.046	0.005	0.9969	0.0069	-0.0039	0.0047
Relative age of first facial hair	0	0.050	0.068	0.724	0.469	0.918	0.123	0.008	1.047	0.0151	-0.0069	0.0057
Relative age voice broke	0	-0.014	0.083	-0.165	0.869	0.986	0.072	0.005	1.0109	0.0085	-0.0034	0.0054
Reproduciblity of spirometry measurement using ERS/ATS criteria	0	-0.017	0.132	-0.130	0.897	0.986	0.014	0.002	1.0081	0.0072	0.0012	0.0047
Rheumatoid Arthritis	24390342	0.006	0.077	0.076	0.939	0.989	0.161	0.029	1.0214	0.0161	0.0275	0.0044
Risk taking	0	0.045	0.068	0.666	0.506	0.949	0.056	0.003	1.0048	0.0087	0.0028	0.0049
Schizophrenia	25056061	0.142	0.056	2.543	0.011	0.221	0.461	0.019	1.0501	0.0131	0.0598	0.0063
Seen a psychiatrist for nerves_ anxiety_ tension or depression	0	0.178	0.074	2.398	0.017	0.268	0.031	0.002	1.004	0.0081	-0.0019	0.0046
Seen doctor (GP) for nerves_ anxiety_ tension or depression	0	0.106	0.064	1.664	0.096	0.542	0.061	0.003	1.0145	0.0097	-0.0018	0.0051
Sensitivity / hurt feelings	0	0.064	0.073	0.877	0.380	0.868	0.063	0.003	1.0213	0.0092	0.0039	0.0053
Serum creatinine	26831199	0.115	0.077	1.487	0.137	0.611	0.108	0.009	0.9751	0.0131	-0.0066	0.0057
Serum creatinine (non-diabetes)	26831199	0.074	0.078	0.942	0.346	0.844	0.115	0.011	0.9785	0.0131	-0.0041	0.0056
Serum cystatin c	26831199	0.043	0.097	0.441	0.660	0.985	0.169	0.067	0.9621	0.014	6.00E-04	0.0052
Serum total cholesterol	27005778	-0.070	0.212	-0.330	0.741	0.985	0.064	0.038	1.0224	0.0154	0.003	0.0049
Serum total triglycerides	27005778	0.060	0.126	0.478	0.633	0.980	0.125	0.035	0.9922	0.0074	-0.0019	0.004
Serumurate overweight	25811787	-0.023	0.110	-0.209	0.835	0.985	0.512	0.293	0.9714	0.0256	0.0055	0.0044
Shortness of breath walking on level ground	0	0.104	0.096	1.081	0.280	0.783	0.049	0.005	1.0074	0.0065	9.00E-04	0.0052
Sitting height	0	-0.007	0.043	-0.170	0.865	0.986	0.322	0.018	1.1362	0.0418	-0.0099	0.0072
Sitting height ratio	25865494	0.053	0.114	0.460	0.646	0.985	0.224	0.028	0.9791	0.0075	-0.0062	0.0049
Sleep duration	27494321	-0.016	0.098	-0.165	0.869	0.986	0.056	0.005	1.0191	0.0072	2.00E-04	0.005
Sleep duration	0	-0.005	0.069	-0.065	0.948	0.991	0.071	0.004	1.0231	0.0101	-0.0025	0.0058
Sleeplessness / insomnia	0	0.029	0.066	0.431	0.667	0.985	0.062	0.003	1.0184	0.0085	0.0039	0.0056
Smoking status: Current	0	0.168	0.075	2.237	0.025	0.365	0.052	0.003	1.0142	0.009	-9.00E-04	0.0051
Smoking status: Previous	0	0.161	0.067	2.402	0.016	0.268	0.052	0.003	1.0201	0.0096	0.002	0.0051
Smoking/smokers in household	0	0.330	0.137	2.417	0.016	0.268	0.010	0.002	1.0112	0.0074	-0.0016	0.0049
Snoring	0	0.019	0.065	0.290	0.772	0.985	0.060	0.003	1.0235	0.0092	-0.0037	0.0055
Sodium in urine	0	0.021	0.063	0.341	0.733	0.985	0.071	0.003	1.0291	0.01	0.0022	0.0059
Squamous cell lung cancer	27488534	0.175	0.156	1.124	0.261	0.772	0.039	0.012	1.0098	0.0066	0.0262	0.0044
Standing height	0	-0.011	0.044	-0.254	0.800	0.985	0.451	0.022	1.2501	0.0572	-0.0122	0.0085
Started insulin within one year diagnosis of diabetes	0	-0.205	0.156	-1.315	0.189	0.698	0.166	0.046	1.0066	0.0083	0.0059	0.0052
Subjective well being	27089181	0.100	0.088	1.129	0.259	0.772	0.025	0.002	1.0011	0.0072	-0.0047	0.0051
Suffer from nerves	0	0.114	0.077	1.488	0.137	0.611	0.046	0.003	0.9989	0.0094	-0.0016	0.005
Systemic lupus erythematosus	26502338	-0.055	0.139	-0.396	0.692	0.985	0.389	0.067	1.1057	0.0097	0.0375	0.006
Systolic blood pressure_ automated reading	0	0.023	0.063	0.368	0.713	0.985	0.128	0.006	1.0669	0.0154	0.0033	0.0067
Taking other prescription medications	0	0.071	0.073	0.962	0.336	0.831	0.043	0.002	1.0161	0.0085	-0.0018	0.0054

Target heart rate achieved	0	0.131	0.174	0.752	0.452	0.918	0.034	0.011	1.0068	0.0069	-0.0023	0.0045
Tense / highly strung	0	0.095	0.076	1.238	0.216	0.740	0.057	0.003	0.9959	0.0095	0.005	0.0054
Time from waking to first cigarette	0	0.261	0.159	1.639	0.101	0.553	0.085	0.026	1.0161	0.0075	-0.003	0.0046
Time spent driving	0	0.152	0.086	1.755	0.079	0.505	0.043	0.003	1.0011	0.0071	-1.00E-04	0.0051
Time spent using computer	0	-0.160	0.057	-2.807	0.005	0.125	0.096	0.004	1.0447	0.0093	0.0013	0.0052
Time spent watching television (TV)	0	0.159	0.060	2.662	0.008	0.167	0.099	0.004	1.0567	0.0108	0.0058	0.0058
Tinnitus: Yes_ but not now_ but have in the past	0	0.146	0.166	0.879	0.379	0.868	0.018	0.004	0.9913	0.0063	3.20E-05	0.005
Tinnitus: Yes_ now most or all of the time	0	-0.021	0.112	-0.190	0.850	0.985	0.034	0.005	1.0014	0.0067	-0.0039	0.0042
Tinnitus: Yes_ now some of the time	0	0.077	0.240	0.321	0.748	0.985	0.009	0.004	0.9988	0.0063	0.0022	0.0054
Tobacco smoking: Ex-smoker	0	0.222	0.110	2.023	0.043	0.433	0.077	0.008	1.0088	0.008	-0.005	0.0051
Total Cholesterol	20686565	-0.018	0.079	-0.233	0.816	0.985	0.134	0.024	1.0205	0.0346	0.0042	0.0057
Total cholesterol in HDL	27005778	0.046	0.145	0.315	0.753	0.985	0.092	0.028	1.0072	0.0126	0.0014	0.0042
Total cholesterol in IDL	27005778	0.008	0.209	0.038	0.970	0.995	0.071	0.044	1.019	0.0196	9.00E-04	0.005
Total cholesterol in large HDL	27005778	0.126	0.142	0.889	0.374	0.867	0.103	0.028	1.0072	0.0142	2.00E-04	0.004
Total cholesterol in large LDL	27005778	0.005	0.211	0.026	0.980	0.995	0.065	0.046	1.0151	0.0224	0.0016	0.0049
Total cholesterol in large VLDL	27005778	0.064	0.131	0.489	0.625	0.980	0.115	0.029	0.9923	0.0066	0.0022	0.0039
Total cholesterol in LDL	27005778	-0.043	0.209	-0.204	0.839	0.985	0.068	0.050	1.014	0.0236	0.0013	0.0049
Total cholesterol in medium HDL	27005778	0.066	0.190	0.348	0.728	0.985	0.053	0.025	0.9993	0.0089	0.002	0.0045
Total cholesterol in medium LDL	27005778	-0.019	0.208	-0.089	0.929	0.986	0.069	0.046	1.0131	0.0219	4.00E-04	0.0049
Total cholesterol in medium VLDL	27005778	0.068	0.129	0.530	0.596	0.980	0.125	0.035	0.9924	0.0075	-0.0013	0.0041
Total cholesterol in small LDL	27005778	-0.049	0.204	-0.242	0.809	0.985	0.069	0.043	1.0166	0.019	6.00E-04	0.005
Total cholesterol in small VLDL	27005778	0.048	0.166	0.290	0.772	0.985	0.090	0.032	1.0056	0.0096	9.00E-04	0.0046
Total cholesterol in very large HDL	27005778	0.287	0.255	1.126	0.260	0.772	0.033	0.023	1.0132	0.0115	-0.0028	0.0045
Total lipids in chylomicrons and largest VLDL particles	27005778	0.159	0.146	1.085	0.278	0.783	0.118	0.028	0.9908	0.0067	-0.0019	0.0044
Total lipids in IDL	27005778	0.018	0.197	0.092	0.927	0.986	0.073	0.043	1.0193	0.0187	0.0013	0.0048
Total lipids in large HDL	27005778	0.039	0.135	0.290	0.772	0.985	0.128	0.031	1.0014	0.0148	0.003	0.0042
Total lipids in large LDL	27005778	-0.028	0.208	-0.134	0.893	0.986	0.074	0.050	1.0149	0.0219	0.0013	0.005
Total lipids in large VLDL	27005778	0.145	0.132	1.097	0.273	0.782	0.138	0.030	0.9856	0.0063	-0.0033	0.0043
Total lipids in medium HDL	27005778	0.006	0.187	0.033	0.974	0.995	0.059	0.026	0.9971	0.0079	0.0035	0.0046
Total lipids in medium LDL	27005778	-0.037	0.202	-0.183	0.855	0.985	0.081	0.049	1.0103	0.0206	0.0013	0.0049
Total lipids in medium VLDL	27005778	0.105	0.127	0.824	0.410	0.903	0.140	0.034	0.986	0.0066	-0.0041	0.0041
Total lipids in small HDL	27005778	-0.333	0.239	-1.394	0.163	0.656	0.053	0.026	1.0028	0.0073	0.0058	0.0043
Total lipids in small LDL	27005778	0.021	0.186	0.111	0.912	0.986	0.086	0.044	1.0083	0.0175	-2.00E-04	0.0047
Total lipids in small VLDL	27005778	0.049	0.129	0.379	0.705	0.985	0.144	0.037	0.9921	0.008	-0.0022	0.0043
Total lipids in very large HDL	27005778	0.108	0.194	0.558	0.577	0.980	0.059	0.028	1.0164	0.0147	9.00E-04	0.0044
Total lipids in very large VLDL	27005778	0.079	0.136	0.580	0.562	0.980	0.136	0.030	0.9789	0.007	-0.002	0.0044
Total lipids in very small VLDL	27005778	0.065	0.150	0.433	0.665	0.985	0.107	0.035	1.0098	0.0126	-3.46E-06	0.0045
Townsend deprivation index at recruitment	0	0.118	0.067	1.750	0.080	0.505	0.034	0.002	1.0348	0.0071	-2.00E-04	0.0047
Transferrin	25352340	-0.049	0.128	-0.385	0.701	0.985	0.162	0.076	1.0623	0.0234	0.0031	0.0058
Transport type for commuting to job workplace: Car/motor vehicle	0	0.133	0.107	1.238	0.216	0.740	0.024	0.003	1.0244	0.007	0.0077	0.0046
Transport type for commuting to job workplace: Cycle	0	-0.286	0.097	-2.952	0.003	0.092	0.031	0.003	1.0162	0.0076	-0.0014	0.0048
Transport type for commuting to job workplace: Public transport	0	-0.168	0.108	-1.555	0.120	0.602	0.031	0.003	1.0253	0.0073	-0.0069	0.0047
Transport type for commuting to job workplace: Walk	0	-0.246	0.121	-2.032	0.042	0.433	0.017	0.003	1.0041	0.0063	-9.00E-04	0.0044

Triglycerides	20686565	0.027	0.082	0.326	0.745	0.985	0.168	0.029	0.9681	0.0162	-0.001	0.0059
Triglycerides in chylomicrons and largest VLDL particles	27005778	0.187	0.145	1.287	0.198	0.706	0.091	0.027	0.9907	0.0067	-0.0038	0.0042
Triglycerides in IDL	27005778	0.052	0.147	0.351	0.726	0.985	0.108	0.034	1.0104	0.0149	8.00E-04	0.0045
Triglycerides in large VLDL	27005778	0.077	0.135	0.569	0.569	0.980	0.111	0.029	0.989	0.0063	-4.00E-04	0.0041
Triglycerides in medium VLDL	27005778	0.075	0.142	0.526	0.599	0.980	0.096	0.030	0.9992	0.0064	-0.0014	0.004
Triglycerides in small HDL	27005778	-0.037	0.176	-0.209	0.835	0.985	0.066	0.027	0.9989	0.008	0.0024	0.0043
Triglycerides in small VLDL	27005778	0.072	0.132	0.549	0.583	0.980	0.120	0.035	0.9942	0.0072	-0.002	0.004
Triglycerides in very large HDL	27005778	0.292	0.167	1.746	0.081	0.505	0.083	0.026	1.0153	0.0271	-0.0038	0.0046
Triglycerides in very large VLDL	27005778	0.077	0.131	0.589	0.556	0.980	0.118	0.029	0.9818	0.0071	-0.0021	0.0041
Triglycerides in very small VLDL	27005778	0.053	0.131	0.404	0.686	0.985	0.139	0.036	0.9985	0.0096	-0.0011	0.0044
Trunk fat mass	0	0.067	0.050	1.322	0.186	0.696	0.231	0.008	1.0791	0.018	-0.006	0.0075
Trunk fat percentage	0	0.067	0.051	1.323	0.186	0.696	0.207	0.007	1.0847	0.0174	-0.0052	0.0073
Trunk fat-free mass	0	0.013	0.044	0.291	0.771	0.985	0.287	0.013	1.1371	0.0312	-0.0035	0.0071
Trunk predicted mass	0	0.010	0.044	0.223	0.824	0.985	0.285	0.013	1.1364	0.031	-0.003	0.0071
Type 2 Diabetes	22885922	0.076	0.101	0.754	0.451	0.918	0.088	0.009	1.0079	0.0071	0.0294	0.0049
Type of tobacco previously smoked: Cigars or pipes	0	-0.130	0.212	-0.611	0.541	0.975	0.013	0.005	0.9995	0.0061	-0.0015	0.0042
ypes of physical activity in last 4 weeks: Heavy DIY (eg: weeding_ lawn mowing_ carpentry_ digging	0	-0.165	0.073	-2.264	0.024	0.348	0.034	0.002	1.0063	0.0072	-0.0062	0.0052
Types of physical activity in last 4 weeks: Light DIY (eg: pruning_ watering the lawn)	0	-0.287	0.070	-4.094	0.000	0.004	0.039	0.002	1.0061	0.0081	-0.0015	0.0052
Types of physical activity in last 4 weeks: None of the above	0	0.176	0.084	2.089	0.037	0.433	0.024	0.002	1.0169	0.0073	0.0047	0.005
Types of physical activity in last 4 weeks: Other exercises (eg: swimming_ cycling_ keep fit_ bowling	0	-0.109	0.066	-1.637	0.102	0.553	0.047	0.002	1.0125	0.0084	-0.0048	0.0052
Types of physical activity in last 4 weeks: Strenuous sports	0	-0.021	0.086	-0.246	0.806	0.985	0.022	0.002	1.0106	0.0069	-0.0049	0.0045
Types of physical activity in last 4 weeks: Walking for pleasure (not as a means of transport)	0	-0.286	0.079	-3.595	0.000	0.017	0.037	0.002	1.0404	0.0072	0.0052	0.0055
Types of transport used (excluding work): Car/motor vehicle	0	0.064	0.088	0.725	0.469	0.918	0.024	0.002	1.0096	0.007	-0.0028	0.005
Types of transport used (excluding work): Cycle	0	-0.120	0.084	-1.431	0.153	0.631	0.025	0.002	1.0257	0.0068	2.00E-04	0.005
Types of transport used (excluding work): Public transport	0	-0.402	0.092	-4.390	0.000	0.002	0.022	0.002	1.0271	0.0071	0.0017	0.0051
Types of transport used (excluding work): Walk	0	-0.403	0.085	-4.743	0.000	0.001	0.033	0.002	1.0345	0.0087	0.0043	0.0051
Tyrosine	27005778	-0.088	0.155	-0.570	0.569	0.980	0.077	0.027	0.998	0.0064	0.0052	0.0044
Ulcerative colitis	26192919	0.034	0.099	0.341	0.733	0.985	0.258	0.031	1.0471	0.0094	0.0616	0.0048
Underlying (primary) cause of death: ICD10: E85.4 Organ-limited amyloidosis	0	0.384	0.345	1.112	0.266	0.778	0.064	0.060	0.9994	0.0068	-0.0032	0.0044
Underlying (primary) cause of death: ICD10: J84.1 Other interstitial pulmonary diseases with fibrosis	0	-0.005	0.238	-0.022	0.982	0.995	0.097	0.065	0.9941	0.007	-0.0044	0.0047
Urate	23263486	-0.084	0.060	-1.402	0.161	0.650	0.176	0.058	0.9532	0.0488	0.0034	0.0051
Urinary albumin-to-creatinine ratio	26631737	-0.167	0.145	-1.152	0.249	0.772	0.046	0.009	0.9963	0.0068	-6.00E-04	0.0051
Urinary albumin-to-creatinine ratio (non-diabetes)	26631737	-0.260	0.142	-1.831	0.067	0.505	0.053	0.011	0.995	0.0072	0.0019	0.005
Used an inhaler for chest within last hour	0	-0.010	0.199	-0.050	0.961	0.995	0.004	0.002	1.0024	0.0064	-0.0039	0.0046
Usual walking pace	0	-0.052	0.057	-0.919	0.358	0.849	0.078	0.003	1.0267	0.0096	-0.0049	0.0053
Valine	27005778	-0.241	0.165	-1.463	0.143	0.612	0.060	0.020	1.0162	0.0075	0.0084	0.0041
Vascular/heart problems diagnosed by doctor: Angina	0	-0.043	0.091	-0.475	0.635	0.980	0.022	0.002	1.0247	0.0077	0.0132	0.0048
Vascular/heart problems diagnosed by doctor: Heart attack	0	-0.117	0.091	-1.279	0.201	0.709	0.019	0.002	1.0124	0.0074	0.0072	0.0043
Vascular/heart problems diagnosed by doctor: High blood pressure	0	0.039	0.061	0.636	0.525	0.962	0.116	0.005	1.0614	0.017	0.0053	0.0065
Vascular/heart problems diagnosed by doctor: None of the above	0	-0.034	0.060	-0.570	0.569	0.980	0.113	0.005	1.0598	0.0157	-0.0066	0.0063
Vitamin and mineral supplements: Multivitamins +/- minerals	0	0.101	0.083	1.219	0.223	0.740	0.024	0.002	1.0077	0.0074	-0.008	0.0049
Vitamin and mineral supplements: None of the above	0	-0.092	0.075	-1.217	0.224	0.740	0.030	0.002	0.9947	0.0067	0.0092	0.005
Vitamin and mineral supplements: Vitamin A	0	0.203	0.203	1.002	0.317	0.813	0.004	0.002	1.0041	0.0062	-0.0014	0.0051

Vitamin and mineral supplements: Vitamin B	0	-0.070	0.131	-0.532	0.595	0.980	0.008	0.002	0.9992	0.0066	-0.0017	0.0046
Vitamin and mineral supplements: Vitamin C	0	0.064	0.110	0.580	0.562	0.980	0.013	0.002	0.9985	0.0075	-0.0031	0.0047
Vitamin and mineral supplements: Vitamin D	0	-0.029	0.157	-0.186	0.853	0.985	0.006	0.002	0.9981	0.0071	-5.00E-04	0.0048
Vitamin and mineral supplements: Vitamin E	0	-0.078	0.162	-0.479	0.632	0.980	0.007	0.001	0.9952	0.0062	0.0037	0.0052
Waist circumference	25673412	0.074	0.068	1.090	0.276	0.783	0.121	0.005	0.8452	0.0076	-0.0021	0.0048
Waist circumference	0	0.023	0.051	0.446	0.655	0.985	0.200	0.007	1.0623	0.0169	-0.0028	0.0072
Waist-to-hip ratio	25673412	0.058	0.068	0.848	0.397	0.892	0.113	0.007	0.9212	0.0089	0.0057	0.0055
Wears glasses or contact lenses	0	-0.048	0.101	-0.477	0.633	0.980	0.016	0.002	1.0011	0.0076	-2.00E-04	0.005
Weight	0	0.050	0.048	1.030	0.303	0.802	0.265	0.010	1.0856	0.0216	-0.007	0.0076
Weight change compared with 1 year ago	0	0.051	0.133	0.379	0.705	0.985	0.009	0.001	1.0226	0.0063	0.0019	0.0049
Wheeze or whistling in the chest in last year	0	0.092	0.064	1.442	0.149	0.630	0.060	0.003	1.0317	0.0099	0.0031	0.0053
Whole body fat mass	0	0.072	0.051	1.410	0.158	0.645	0.234	0.008	1.0745	0.0177	-0.0067	0.0076
Whole body fat-free mass	0	0.015	0.045	0.336	0.737	0.985	0.289	0.012	1.1295	0.0295	-0.0045	0.0072
Whole body water mass	0	0.014	0.045	0.304	0.761	0.985	0.289	0.012	1.1289	0.0292	-0.0046	0.0072
Why reduced smoking: Illness or ill health	0	0.061	0.214	0.284	0.777	0.985	0.108	0.043	0.9984	0.0065	-0.0014	0.0051
Why stopped smoking: Doctors advice	0	0.025	0.184	0.134	0.893	0.986	0.018	0.005	0.9973	0.0059	0.0043	0.005
Why stopped smoking: Health precaution	0	0.026	0.099	0.266	0.790	0.985	0.055	0.006	0.9985	0.0066	-0.0026	0.0047
Why stopped smoking: None of the above	0	-0.024	0.118	-0.201	0.841	0.985	0.040	0.006	0.9974	0.0065	0.001	0.005
Work/job satisfaction	0	0.091	0.115	0.795	0.426	0.911	0.049	0.007	0.9945	0.0067	-0.0037	0.0049
Worrier / anxious feelings	0	0.066	0.080	0.827	0.408	0.903	0.079	0.005	1.001	0.012	0.0077	0.0061
Worry too long after embarrassment	0	-0.054	0.073	-0.741	0.459	0.918	0.065	0.003	1.0172	0.0098	-0.0017	0.0057
Years of schooling (proxy cognitive performance)	25201988	-0.197	0.086	-2.301	0.021	0.335	0.107	0.007	1.0266	0.0092	0.0016	0.006
Years of schooling 2013	23722424	-0.212	0.089	-2.396	0.017	0.268	0.084	0.006	1.019	0.009	0.0021	0.0057
Years of schooling 2016	27225129	-0.226	0.059	-3.856	0.000	0.007	0.127	0.004	0.9263	0.01	-0.0049	0.0055

Source: number denotes PMID; UKBB, UK Biobank; rg, genetic correlation (regression); se, standard error; FDR, false discovery rate adjusted p-value; h2 obs, shared heritability observed; h2 int, shared heritability intercept, gcov_int, cross-trait intercept

Table S2. Mendelian randomization analysis performed on 635 GWASes across 570 phenotypic traits; (clumping threshold of R2 = 0.001, kb = 10,000)

Trait	MR test	n0 of SNPs	beta	se	p-value
1 5-anhydroglucitol (1 5-AG) || id:419	MR Egger	3	-1.480	1.077	0.400
1 5-anhydroglucitol (1 5-AG) || id:419	Weighted median	3	-0.459	0.420	0.274
1 5-anhydroglucitol (1 5-AG) || id:419	Inverse variance weighted	3	-0.422	0.379	0.265
1-arachidonoylglycerophosphocholine* || id:558	MR Egger	3	0.096	0.460	0.869
1-arachidonoylglycerophosphocholine* || id:558	Weighted median	3	-0.008	0.231	0.972
1-arachidonoylglycerophosphocholine* || id:558	Inverse variance weighted	3	-0.031	0.237	0.895
1-arachidonoylglycerophosphoethanolamine* || id:682	MR Egger	3	0.770	1.142	0.622
1-arachidonoylglycerophosphoethanolamine* || id:682	Weighted median	3	0.018	0.339	0.958
1-arachidonoylglycerophosphoethanolamine* || id:682	Inverse variance weighted	3	-0.071	0.320	0.825
1-arachidonoylglycerophosphoinositol* || id:634	MR Egger	4	-0.854	1.169	0.541
1-arachidonoylglycerophosphoinositol* || id:634	Weighted median	4	0.048	0.339	0.887
1-arachidonoylglycerophosphoinositol* || id:634	Inverse variance weighted	4	0.243	0.293	0.406
1-eicosatrienoylglycerophosphocholine* || id:602	Inverse variance weighted	2	-0.434	0.390	0.266
1-linoleoylglycerophosphoethanolamine* || id:497	Inverse variance weighted	2	0.060	0.293	0.838
1-palmitoylglycerophosphoethanolamine || id:707	Inverse variance weighted	2	0.014	0.458	0.976
10-undecenoate (11:1n1) || id:482	Inverse variance weighted	2	0.072	0.274	0.793
18,02 linoleic acid (LA) || id:893	MR Egger	13	0.142	0.090	0.140
18,02 linoleic acid (LA) || id:893	Weighted median	13	0.066	0.050	0.179
18,02 linoleic acid (LA) || id:893	Inverse variance weighted	13	0.092	0.037	0.012
2-aminobutyrate || id:471	Inverse variance weighted	2	-0.369	0.643	0.566
2-hydroxyisobutyrate || id:427	MR Egger	3	-0.332	2.755	0.924
2-hydroxyisobutyrate || id:427	Weighted median	3	0.081	0.545	0.882
2-hydroxyisobutyrate || id:427	Inverse variance weighted	3	0.403	0.809	0.618
22,06 docosahexaenoic acid || id:852	MR Egger	5	-0.006	0.396	0.989
22,06 docosahexaenoic acid || id:852	Weighted median	5	0.072	0.082	0.374
22,06 docosahexaenoic acid || id:852	Inverse variance weighted	5	0.117	0.067	0.080
3-dehydrocarnitine* || id:500	MR Egger	3	-2.949	4.390	0.623
3-dehydrocarnitine* || id:500	Weighted median	3	-0.064	0.507	0.900
3-dehydrocarnitine* || id:500	Inverse variance weighted	3	-0.015	0.617	0.981
3-methyl-2-oxovalerate || id:383	MR Egger	3	-1.271	4.659	0.830
3-methyl-2-oxovalerate || id:383	Weighted median	3	0.699	0.756	0.355
3-methyl-2-oxovalerate || id:383	Inverse variance weighted	3	0.619	0.675	0.360
4-acetamidobutanoate || id:339	Inverse variance weighted	2	-0.304	1.070	0.776
4-methyl-2-oxopentanoate || id:429	Inverse variance weighted	2	-1.041	1.730	0.547
Acetoacetate || id:838	Inverse variance weighted	2	0.126	0.347	0.716
Acetylcarnitine || id:463	Inverse variance weighted	2	-0.042	0.465	0.929
Acetylphosphate || id:378	Inverse variance weighted	2	1.296	1.196	0.279
Adiponectin || id:1	MR Egger	14	-0.049	0.112	0.670
Adiponectin || id:1	Weighted median	14	-0.060	0.099	0.548

Adiponectin || id:1	Inverse variance weighted	14	-0.071	0.074	0.342
ADpSGEGDFXAEGGGVR* || id:601	Inverse variance weighted	2	0.313	0.251	0.213
ADSGEGDFXAEGGGVR* || id:539	Inverse variance weighted	2	-0.042	0.142	0.766
Age at first live birth || id:UKB-a:319	MR Egger	17	-0.298	0.868	0.737
Age at first live birth || id:UKB-a:319	Weighted median	17	-0.084	0.212	0.693
Age at first live birth || id:UKB-a:319	Inverse variance weighted	17	-0.116	0.155	0.453
Age at last live birth || id:UKB-a:320	MR Egger	4	-2.801	3.630	0.521
Age at last live birth || id:UKB-a:320	Weighted median	4	-0.538	0.391	0.169
Age at last live birth || id:UKB-a:320	Inverse variance weighted	4	0.048	0.419	0.908
Age at menarche || id:1095	MR Egger	60	0.114	0.180	0.528
Age at menarche || id:1095	Weighted median	60	0.069	0.068	0.309
Age at menarche || id:1095	Inverse variance weighted	60	0.055	0.047	0.247
Age at menopause || id:1004	MR Egger	38	-0.011	0.037	0.765
Age at menopause || id:1004	Weighted median	38	-0.020	0.020	0.326
Age at menopause || id:1004	Inverse variance weighted	38	-0.013	0.015	0.393
Age completed full time education || id:UKB-a:505	MR Egger	20	-0.647	1.122	0.571
Age completed full time education || id:UKB-a:505	Weighted median	20	-0.325	0.300	0.279
Age completed full time education || id:UKB-a:505	Inverse variance weighted	20	-0.344	0.259	0.183
Age started oral contraceptive pill || id:UKB-a:323	Inverse variance weighted	2	1.130	0.447	0.011
Age when periods started (menarche) || id:UKB-a:315	MR Egger	118	0.192	0.227	0.400
Age when periods started (menarche) || id:UKB-a:315	Weighted median	118	0.061	0.114	0.593
Age when periods started (menarche) || id:UKB-a:315	Inverse variance weighted	118	0.154	0.078	0.048
Alanine || id:469	Inverse variance weighted	2	-0.078	0.972	0.936
Alanine || id:840	MR Egger	6	0.274	0.366	0.496
Alanine || id:840	Weighted median	6	0.094	0.097	0.335
Alanine || id:840	Inverse variance weighted	6	0.072	0.078	0.359
Albumin || id:841	Inverse variance weighted	2	0.055	0.163	0.737
Alcohol intake frequency. || id:UKB-a:25	MR Egger	42	0.050	0.124	0.691
Alcohol intake frequency. || id:UKB-a:25	Weighted median	42	-0.037	0.107	0.731
Alcohol intake frequency. || id:UKB-a:25	Inverse variance weighted	42	-0.019	0.071	0.786
Alcohol intake versus 10 years previously || id:UKB-a:32	MR Egger	9	-0.338	1.037	0.754
Alcohol intake versus 10 years previously || id:UKB-a:32	Weighted median	9	0.480	0.510	0.347
Alcohol intake versus 10 years previously || id:UKB-a:32	Inverse variance weighted	9	0.772	0.464	0.096
Alcohol usually taken with meals || id:UKB-a:31	MR Egger	14	-0.863	3.984	0.832
Alcohol usually taken with meals || id:UKB-a:31	Weighted median	14	-0.063	0.530	0.905
Alcohol usually taken with meals || id:UKB-a:31	Inverse variance weighted	14	0.103	0.392	0.792
Alpha-hydroxyisovalerate || id:616	MR Egger	3	-0.370	0.885	0.748
Alpha-hydroxyisovalerate || id:616	Weighted median	3	-0.130	0.278	0.640
Alpha-hydroxyisovalerate || id:616	Inverse variance weighted	3	-0.091	0.265	0.732
Alzheimer's disease || id:297	MR Egger	12	-0.039	0.123	0.755
Alzheimer's disease || id:297	Weighted median	12	-0.045	0.047	0.341
Alzheimer's disease || id:297	Inverse variance weighted	12	-0.039	0.034	0.255

Alzheimer's disease || id:298	MR Egger	19	-0.019	0.116	0.875
Alzheimer's disease || id:298	Weighted median	19	-0.041	0.045	0.364
Alzheimer's disease || id:298	Inverse variance weighted	19	-0.020	0.036	0.574
Amyotrophic lateral sclerosis || id:1085	MR Egger	7	5.521	1.072	0.004
Amyotrophic lateral sclerosis || id:1085	Weighted median	7	3.876	0.423	0.000
Amyotrophic lateral sclerosis || id:1085	Inverse variance weighted	7	4.026	0.383	0.000
Amyotrophic lateral sclerosis || id:1086	MR Egger	4	1.058	0.334	0.087
Amyotrophic lateral sclerosis || id:1086	Weighted median	4	0.982	0.112	0.000
Amyotrophic lateral sclerosis || id:1086	Inverse variance weighted	4	0.951	0.098	0.000
Androsterone sulfate || id:460	MR Egger	3	-0.139	0.117	0.444
Androsterone sulfate || id:460	Weighted median	3	-0.062	0.078	0.431
Androsterone sulfate || id:460	Inverse variance weighted	3	-0.024	0.108	0.825
Apolipoprotein A-I || id:842	MR Egger	10	0.016	0.128	0.902
Apolipoprotein A-I || id:842	Weighted median	10	-0.068	0.059	0.248
Apolipoprotein A-I || id:842	Inverse variance weighted	10	-0.060	0.047	0.200
Apolipoprotein B || id:843	MR Egger	18	0.015	0.075	0.847
Apolipoprotein B || id:843	Weighted median	18	0.061	0.052	0.243
Apolipoprotein B || id:843	Inverse variance weighted	18	0.097	0.044	0.026
Arachidonic acid (20:4n6) || id:1141	MR Egger	86	-0.002	0.002	0.318
Arachidonic acid (20:4n6) || id:1141	Weighted median	86	-0.001	0.002	0.552
Arachidonic acid (20:4n6) || id:1141	Inverse variance weighted	86	0.000	0.001	0.765
Arm fat percentage (left) || id:UKB-a:286	MR Egger	248	-0.214	0.257	0.406
Arm fat percentage (left) || id:UKB-a:286	Weighted median	248	0.087	0.104	0.401
Arm fat percentage (left) || id:UKB-a:286	Inverse variance weighted	248	-0.001	0.079	0.989
Asparagine || id:638	Inverse variance weighted	2	-0.309	0.855	0.718
Asthma || id:44	MR Egger	8	-0.101	0.236	0.683
Asthma || id:44	Weighted median	8	0.047	0.049	0.333
Asthma || id:44	Inverse variance weighted	8	0.050	0.037	0.175
Average number of double bonds in a fatty acid chain || id:851	MR Egger	5	0.071	0.086	0.470
Average number of double bonds in a fatty acid chain || id:851	Weighted median	5	-0.002	0.047	0.972
Average number of double bonds in a fatty acid chain || id:851	Inverse variance weighted	5	-0.022	0.045	0.616
Average number of methylene groups in a fatty acid chain || id:848	MR Egger	3	-0.004	0.153	0.983
Average number of methylene groups in a fatty acid chain || id:848	Weighted median	3	-0.010	0.058	0.863
Average number of methylene groups in a fatty acid chain || id:848	Inverse variance weighted	3	-0.020	0.055	0.719
Average number of methylene groups per double bond || id:847	MR Egger	5	-0.027	0.087	0.774
Average number of methylene groups per double bond || id:847	Weighted median	5	-0.001	0.045	0.984
Average number of methylene groups per double bond || id:847	Inverse variance weighted	5	0.000	0.040	0.997
Average weekly beer plus cider intake || id:UKB-a:28	MR Egger	11	0.298	1.303	0.824
Average weekly beer plus cider intake || id:UKB-a:28	Weighted median	11	0.457	0.443	0.302
Average weekly beer plus cider intake || id:UKB-a:28	Inverse variance weighted	11	0.409	0.452	0.366
Average weekly champagne plus white wine intake || id:UKB-a:27	Inverse variance weighted	2	-0.373	0.654	0.568
Average weekly red wine intake || id:UKB-a:26	MR Egger	12	0.519	0.744	0.501

Average weekly red wine intake || id:UKB-a:26	Weighted median	12	0.076	0.373	0.839
Average weekly red wine intake || id:UKB-a:26	Inverse variance weighted	12	-0.132	0.279	0.636
Betaine || id:362	MR Egger	3	0.530	1.347	0.761
Betaine || id:362	Weighted median	3	-0.075	0.495	0.880
Betaine || id:362	Inverse variance weighted	3	-0.184	0.460	0.690
Bilateral oophorectomy (both ovaries removed) || id:UKB-a:325	MR Egger	4	-2.404	6.767	0.756
Bilateral oophorectomy (both ovaries removed) || id:UKB-a:325	Weighted median	4	-0.892	1.385	0.520
Bilateral oophorectomy (both ovaries removed) || id:UKB-a:325	Inverse variance weighted	4	-0.165	1.215	0.892
Bipolar disorder || id:800	MR Egger	4	0.185	0.285	0.583
Bipolar disorder || id:800	Weighted median	4	0.007	0.063	0.915
Bipolar disorder || id:800	Inverse variance weighted	4	-0.019	0.070	0.789
Bipolar disorder || id:801	MR Egger	4	0.185	0.285	0.583
Bipolar disorder || id:801	Weighted median	4	0.007	0.065	0.916
Bipolar disorder || id:801	Inverse variance weighted	4	-0.019	0.070	0.789
Birth length || id:29	Inverse variance weighted	2	0.250	0.190	0.190
Birth weight || id:1083	MR Egger	43	0.249	0.302	0.415
Birth weight || id:1083	Weighted median	43	-0.067	0.121	0.580
Birth weight || id:1083	Inverse variance weighted	43	-0.011	0.089	0.899
Birth weight || id:1084	MR Egger	46	0.142	0.294	0.630
Birth weight || id:1084	Weighted median	46	-0.061	0.118	0.606
Birth weight || id:1084	Inverse variance weighted	46	-0.008	0.085	0.929
Birth weight || id:27	MR Egger	4	-0.880	0.639	0.303
Birth weight || id:27	Weighted median	4	-0.103	0.136	0.451
Birth weight || id:27	Inverse variance weighted	4	0.008	0.117	0.946
Birth weight || id:UKB-a:198	MR Egger	81	0.062	0.245	0.802
Birth weight || id:UKB-a:198	Weighted median	81	0.055	0.104	0.597
Birth weight || id:UKB-a:198	Inverse variance weighted	81	0.070	0.073	0.340
Birth weight of first child || id:UKB-a:318	MR Egger	42	0.146	0.292	0.620
Birth weight of first child || id:UKB-a:318	Weighted median	42	-0.030	0.101	0.769
Birth weight of first child || id:UKB-a:318	Inverse variance weighted	42	-0.037	0.077	0.634
Body mass index || id:2	MR Egger	79	-0.088	0.176	0.617
Body mass index || id:2	Weighted median	79	-0.099	0.101	0.327
Body mass index || id:2	Inverse variance weighted	79	-0.037	0.072	0.608
Body mass index || id:835	MR Egger	68	0.109	0.233	0.641
Body mass index || id:835	Weighted median	68	0.017	0.114	0.882
Body mass index || id:835	Inverse variance weighted	68	0.017	0.079	0.830
Bradykinin des-arg(9) || id:656	MR Egger	3	-0.224	0.208	0.476
Bradykinin des-arg(9) || id:656	Weighted median	3	0.032	0.072	0.658
Bradykinin des-arg(9) || id:656	Inverse variance weighted	3	0.048	0.066	0.467
Breastfed as a baby || id:UKB-a:33	Inverse variance weighted	2	-2.503	2.677	0.350
Bring up phlegm/sputum/mucus on most days || id:UKB-a:259	MR Egger	3	2.857	22.350	0.919
Bring up phlegm/sputum/mucus on most days || id:UKB-a:259	Weighted median	3	-0.675	1.254	0.590

Bring up phlegm/sputum/mucus on most days || id:UKB-a:259	Inverse variance weighted	3	-0.573	1.113	0.607
Butyrylcarnitine || id:476	MR Egger	6	0.032	0.118	0.799
Butyrylcarnitine || id:476	Weighted median	6	-0.001	0.080	0.988
Butyrylcarnitine || id:476	Inverse variance weighted	6	-0.037	0.078	0.639
C-reactive protein || id:1015	MR Egger	3	0.729	0.379	0.305
C-reactive protein || id:1015	Weighted median	3	-0.026	0.133	0.846
C-reactive protein || id:1015	Inverse variance weighted	3	-0.020	0.137	0.883
Cancer code self-reported: basal cell carcinoma || id:UKB-a:59	MR Egger	14	4.026	8.639	0.650
Cancer code self-reported: basal cell carcinoma || id:UKB-a:59	Weighted median	14	0.570	3.241	0.860
Cancer code self-reported: basal cell carcinoma || id:UKB-a:59	Inverse variance weighted	14	1.205	2.417	0.618
Cancer code self-reported: breast cancer || id:UKB-a:55	MR Egger	17	1.437	3.823	0.712
Cancer code self-reported: breast cancer || id:UKB-a:55	Weighted median	17	0.238	1.744	0.892
Cancer code self-reported: breast cancer || id:UKB-a:55	Inverse variance weighted	17	0.886	1.271	0.486
Cancer code self-reported: lung cancer || id:UKB-a:54	MR Egger	4	-64.615	52.453	0.343
Cancer code self-reported: lung cancer || id:UKB-a:54	Weighted median	4	13.236	28.643	0.644
Cancer code self-reported: lung cancer || id:UKB-a:54	Inverse variance weighted	4	16.289	24.970	0.514
Cancer code self-reported: malignant melanoma || id:UKB-a:58	MR Egger	4	11.550	13.318	0.477
Cancer code self-reported: malignant melanoma || id:UKB-a:58	Weighted median	4	0.178	5.301	0.973
Cancer code self-reported: malignant melanoma || id:UKB-a:58	Inverse variance weighted	4	-0.933	5.092	0.855
Cancer code self-reported: prostate cancer || id:UKB-a:57	MR Egger	13	3.001	8.717	0.737
Cancer code self-reported: prostate cancer || id:UKB-a:57	Weighted median	13	-3.667	3.433	0.285
Cancer code self-reported: prostate cancer || id:UKB-a:57	Inverse variance weighted	13	-1.433	2.604	0.582
Cancer code self-reported: small intestine/small bowel cancer || id:UKB-a:56	MR Egger	4	94.721	218.012	0.706
Cancer code self-reported: small intestine/small bowel cancer || id:UKB-a:56	Weighted median	4	19.774	35.401	0.576
Cancer code self-reported: small intestine/small bowel cancer || id:UKB-a:56	Inverse variance weighted	4	33.081	30.585	0.279
Cancer code self-reported: squamous cell carcinoma || id:UKB-a:60	MR Egger	3	-17.394	102.421	0.893
Cancer code self-reported: squamous cell carcinoma || id:UKB-a:60	Weighted median	3	41.755	27.708	0.132
Cancer code self-reported: squamous cell carcinoma || id:UKB-a:60	Inverse variance weighted	3	27.572	25.605	0.282
Cancer diagnosed by doctor || id:UKB-a:307	MR Egger	5	15.464	37.118	0.705
Cancer diagnosed by doctor || id:UKB-a:307	Weighted median	5	0.552	1.843	0.765
Cancer diagnosed by doctor || id:UKB-a:307	Inverse variance weighted	5	1.411	1.480	0.340
Caprylate (8:0) || id:481	Inverse variance weighted	2	-0.772	1.239	0.533
Cardioembolic stroke || id:1109	Inverse variance weighted	2	0.050	0.045	0.269
Carnitine || id:379	MR Egger	16	0.842	0.964	0.397
Carnitine || id:379	Weighted median	16	0.420	0.619	0.497
Carnitine || id:379	Inverse variance weighted	16	0.396	0.505	0.433
CD123 on 11c+123+DC || id:159	MR Egger	6	0.131	0.251	0.629
CD123 on 11c+123+DC || id:159	Weighted median	6	0.015	0.060	0.796
CD123 on 11c+123+DC || id:159	Inverse variance weighted	6	0.013	0.049	0.786
CD32 on 11c+123+DC || id:162	Inverse variance weighted	2	0.014	0.069	0.836
CD34 on Stem || id:151	Inverse variance weighted	2	0.040	0.131	0.758
Celiac disease || id:1058	MR Egger	13	-0.010	0.024	0.697

Celiac disease || id:1058	Weighted median	13	0.000	0.018	0.990
Celiac disease || id:1058	Inverse variance weighted	13	-0.001	0.016	0.925
Celiac disease || id:1059	MR Egger	11	0.021	0.139	0.884
Celiac disease || id:1059	Weighted median	11	0.010	0.035	0.784
Celiac disease || id:1059	Inverse variance weighted	11	-0.007	0.037	0.850
Celiac disease || id:1060	MR Egger	7	0.619	0.217	0.036
Celiac disease || id:1060	Weighted median	7	0.049	0.036	0.179
Celiac disease || id:1060	Inverse variance weighted	7	0.024	0.038	0.517
Celiac disease || id:276	MR Egger	12	0.019	0.133	0.887
Celiac disease || id:276	Weighted median	12	-0.002	0.032	0.946
Celiac disease || id:276	Inverse variance weighted	12	-0.011	0.034	0.749
Childhood obesity || id:1096	MR Egger	5	-0.738	0.301	0.092
Childhood obesity || id:1096	Weighted median	5	-0.035	0.051	0.492
Childhood obesity || id:1096	Inverse variance weighted	5	-0.030	0.059	0.612
Cholesterol || id:307	Inverse variance weighted	2	0.048	0.791	0.952
Cholesterol esters in large HDL || id:875	MR Egger	14	0.092	0.106	0.402
Cholesterol esters in large HDL || id:875	Weighted median	14	-0.027	0.048	0.579
Cholesterol esters in large HDL || id:875	Inverse variance weighted	14	-0.064	0.044	0.144
Cholesterol esters in large VLDL || id:881	MR Egger	21	-0.009	0.046	0.842
Cholesterol esters in large VLDL || id:881	Weighted median	21	0.047	0.041	0.256
Cholesterol esters in large VLDL || id:881	Inverse variance weighted	21	0.067	0.032	0.037
Cholesterol esters in large VLDL || id:887	MR Egger	10	0.089	0.117	0.469
Cholesterol esters in large VLDL || id:887	Weighted median	10	0.108	0.068	0.112
Cholesterol esters in large VLDL || id:887	Inverse variance weighted	10	0.107	0.050	0.034
Cholesterol esters in medium HDL || id:899	MR Egger	7	0.194	0.184	0.339
Cholesterol esters in medium HDL || id:899	Weighted median	7	-0.114	0.084	0.174
Cholesterol esters in medium HDL || id:899	Inverse variance weighted	7	-0.162	0.078	0.039
Chronic kidney disease || id:1102	MR Egger	4	0.316	0.193	0.242
Chronic kidney disease || id:1102	Weighted median	4	0.054	0.068	0.425
Chronic kidney disease || id:1102	Inverse variance weighted	4	0.054	0.055	0.327
Chronotype || id:1087	MR Egger	9	0.443	0.618	0.496
Chronotype || id:1087	Weighted median	9	0.243	0.253	0.337
Chronotype || id:1087	Inverse variance weighted	9	0.283	0.198	0.153
cis-trans-18:2 || id:1150	MR Egger	5	1.353	1.020	0.277
cis-trans-18:2 || id:1150	Weighted median	5	1.087	0.860	0.206
cis-trans-18:2 || id:1150	Inverse variance weighted	5	1.301	0.668	0.051
Citrate || id:341	MR Egger	6	0.414	1.829	0.832
Citrate || id:341	Weighted median	6	0.169	0.637	0.791
Citrate || id:341	Inverse variance weighted	6	0.119	0.538	0.824
Citrate || id:849	MR Egger	5	0.515	0.779	0.556
Citrate || id:849	Weighted median	5	0.017	0.104	0.874
Citrate || id:849	Inverse variance weighted	5	-0.019	0.096	0.844

Citrulline || id:356	MR Egger	4	9.322	12.053	0.520
Citrulline || id:356	Weighted median	4	-0.379	0.814	0.641
Citrulline || id:356	Inverse variance weighted	4	-0.367	0.708	0.604
College completion || id:836	MR Egger	3	-0.562	1.431	0.762
College completion || id:836	Weighted median	3	-0.290	0.158	0.067
College completion || id:836	Inverse variance weighted	3	-0.324	0.131	0.013
Comparative body size at age 10 || id:UKB-a:34	MR Egger	155	-0.181	0.209	0.388
Comparative body size at age 10 || id:UKB-a:34	Weighted median	155	-0.129	0.142	0.365
Comparative body size at age 10 || id:UKB-a:34	Inverse variance weighted	155	-0.116	0.095	0.220
Concentration of IDL particles || id:870	MR Egger	18	-0.022	0.057	0.706
Concentration of IDL particles || id:870	Weighted median	18	0.051	0.045	0.250
Concentration of IDL particles || id:870	Inverse variance weighted	18	0.064	0.034	0.059
Concentration of large HDL particles || id:878	MR Egger	12	0.096	0.117	0.434
Concentration of large HDL particles || id:878	Weighted median	12	-0.035	0.048	0.463
Concentration of large HDL particles || id:878	Inverse variance weighted	12	-0.044	0.042	0.290
Concentration of large LDL particles || id:884	MR Egger	21	-0.007	0.047	0.883
Concentration of large LDL particles || id:884	Weighted median	21	0.047	0.042	0.266
Concentration of large LDL particles || id:884	Inverse variance weighted	21	0.064	0.032	0.044
Concentration of large VLDL particles || id:890	MR Egger	8	0.113	0.141	0.454
Concentration of large VLDL particles || id:890	Weighted median	8	0.105	0.070	0.133
Concentration of large VLDL particles || id:890	Inverse variance weighted	8	0.089	0.054	0.099
Copper || id:1073	Inverse variance weighted	2	-0.006	0.047	0.895
Coronary heart disease || id:6	MR Egger	3	0.120	0.441	0.831
Coronary heart disease || id:6	Weighted median	3	0.088	0.061	0.151
Coronary heart disease || id:6	Inverse variance weighted	3	0.075	0.052	0.151
Coronary heart disease || id:7	MR Egger	37	0.162	0.073	0.032
Coronary heart disease || id:7	Weighted median	37	0.110	0.046	0.015
Coronary heart disease || id:7	Inverse variance weighted	37	0.062	0.031	0.047
Coronary heart disease || id:8	MR Egger	15	0.173	0.095	0.092
Coronary heart disease || id:8	Weighted median	15	0.067	0.044	0.124
Coronary heart disease || id:8	Inverse variance weighted	15	0.041	0.032	0.200
Coronary heart disease || id:9	MR Egger	10	0.206	0.137	0.172
Coronary heart disease || id:9	Weighted median	10	0.107	0.061	0.080
Coronary heart disease || id:9	Inverse variance weighted	10	0.055	0.052	0.288
Creatinine (enzymatic) in urine || id:UKB-a:333	MR Egger	21	-0.836	0.945	0.388
Creatinine (enzymatic) in urine || id:UKB-a:333	Weighted median	21	0.285	0.310	0.358
Creatinine (enzymatic) in urine || id:UKB-a:333	Inverse variance weighted	21	0.281	0.247	0.254
Creatinine || id:850	MR Egger	6	-0.133	0.512	0.807
Creatinine || id:850	Weighted median	6	-0.004	0.117	0.976
Creatinine || id:850	Inverse variance weighted	6	-0.016	0.089	0.861
Crohn's disease || id:10	MR Egger	103	-0.018	0.028	0.516
Crohn's disease || id:10	Weighted median	103	-0.017	0.018	0.367

Crohn's disease || id:10	Inverse variance weighted	103	-0.017	0.012	0.172
Crohn's disease || id:11	MR Egger	13	0.014	0.029	0.642
Crohn's disease || id:11	Weighted median	13	-0.026	0.016	0.100
Crohn's disease || id:11	Inverse variance weighted	13	-0.010	0.013	0.474
Crohn's disease || id:12	MR Egger	117	0.023	0.038	0.554
Crohn's disease || id:12	Weighted median	117	-0.003	0.018	0.853
Crohn's disease || id:12	Inverse variance weighted	117	-0.008	0.014	0.572
Crohn's disease || id:30	MR Egger	47	0.016	0.028	0.572
Crohn's disease || id:30	Weighted median	47	-0.012	0.018	0.517
Crohn's disease || id:30	Inverse variance weighted	47	0.001	0.012	0.914
Current tobacco smoking || id:UKB-a:16	MR Egger	16	-2.789	1.816	0.147
Current tobacco smoking || id:UKB-a:16	Weighted median	16	-0.599	0.604	0.321
Current tobacco smoking || id:UKB-a:16	Inverse variance weighted	16	0.515	0.507	0.310
Daytime dozing / sleeping (narcolepsy) || id:UKB-a:15	MR Egger	19	2.106	2.652	0.438
Daytime dozing / sleeping (narcolepsy) || id:UKB-a:15	Weighted median	19	0.365	0.586	0.533
Daytime dozing / sleeping (narcolepsy) || id:UKB-a:15	Inverse variance weighted	19	0.304	0.517	0.556
Decanoylcarnitine || id:618	MR Egger	3	0.926	0.996	0.523
Decanoylcarnitine || id:618	Weighted median	3	0.458	0.230	0.046
Decanoylcarnitine || id:618	Inverse variance weighted	3	0.479	0.266	0.072
Dehydroisoandrosterone sulfate (DHEA-S) || id:478	Inverse variance weighted	2	-0.168	0.515	0.744
Diabetes diagnosed by doctor || id:UKB-a:306	MR Egger	49	-0.687	1.099	0.535
Diabetes diagnosed by doctor || id:UKB-a:306	Weighted median	49	-0.337	0.739	0.649
Diabetes diagnosed by doctor || id:UKB-a:306	Inverse variance weighted	49	-1.211	0.464	0.009
Diagnoses - main ICD10: B37 Candidiasis || id:UKB-a:517	MR Egger	7	69.469	57.662	0.282
Diagnoses - main ICD10: B37 Candidiasis || id:UKB-a:517	Weighted median	7	52.432	25.986	0.044
Diagnoses - main ICD10: B37 Candidiasis || id:UKB-a:517	Inverse variance weighted	7	51.934	20.688	0.012
Diagnoses - main ICD10: C44 Other malignant neoplasms of skin || id:UKB-a:518	MR Egger	20	0.487	5.150	0.926
Diagnoses - main ICD10: C44 Other malignant neoplasms of skin || id:UKB-a:518	Weighted median	20	-0.910	2.237	0.684
Diagnoses - main ICD10: C44 Other malignant neoplasms of skin || id:UKB-a:518	Inverse variance weighted	20	0.370	1.560	0.812
Diagnoses - main ICD10: C50 Malignant neoplasm of breast || id:UKB-a:519	MR Egger	7	-3.512	6.318	0.602
Diagnoses - main ICD10: C50 Malignant neoplasm of breast || id:UKB-a:519	Weighted median	7	0.572	2.507	0.820
Diagnoses - main ICD10: C50 Malignant neoplasm of breast || id:UKB-a:519	Inverse variance weighted	7	1.422	1.980	0.472
Diagnoses - main ICD10: C61 Malignant neoplasm of prostate || id:UKB-a:520	MR Egger	10	2.790	18.030	0.881
Diagnoses - main ICD10: C61 Malignant neoplasm of prostate || id:UKB-a:520	Weighted median	10	-3.880	4.118	0.346
Diagnoses - main ICD10: C61 Malignant neoplasm of prostate || id:UKB-a:520	Inverse variance weighted	10	-3.015	3.198	0.346
Diagnoses - main ICD10: D12 Benign neoplasm of colon rectum anus and anal canal || id:UKB-a:521	MR Egger	3	-6.284	26.943	0.854
Diagnoses - main ICD10: D12 Benign neoplasm of colon rectum anus and anal canal || id:UKB-a:521	Weighted median	3	2.446	4.330	0.572
Diagnoses - main ICD10: D12 Benign neoplasm of colon rectum anus and anal canal || id:UKB-a:521	Inverse variance weighted	3	0.474	3.827	0.901
Diagnoses - main ICD10: D25 Leiomyoma of uterus || id:UKB-a:522	MR Egger	4	-37.213	16.166	0.148
Diagnoses - main ICD10: D25 Leiomyoma of uterus || id:UKB-a:522	Weighted median	4	3.286	5.455	0.547
Diagnoses - main ICD10: D25 Leiomyoma of uterus || id:UKB-a:522	Inverse variance weighted	4	-2.447	6.363	0.700
Diagnoses - main ICD10: E03 Other hypothyroidism || id:UKB-a:523	MR Egger	7	-27.390	119.472	0.828

Diagnoses - main ICD10: E03 Other hypothyroidism || id:UKB-a:523	Weighted median	7	2.119	29.692	0.943
Diagnoses - main ICD10: E03 Other hypothyroidism || id:UKB-a:523	Inverse variance weighted	7	17.639	23.526	0.453
Diagnoses - main ICD10: E04 Other non-toxic goitre || id:UKB-a:524	MR Egger	5	-4.427	48.477	0.933
Diagnoses - main ICD10: E04 Other non-toxic goitre || id:UKB-a:524	Weighted median	5	0.870	10.318	0.933
Diagnoses - main ICD10: E04 Other non-toxic goitre || id:UKB-a:524	Inverse variance weighted	5	1.041	8.460	0.902
Diagnoses - main ICD10: G56 Mononeuropathies of upper limb || id:UKB-a:528	MR Egger	5	8.542	12.508	0.544
Diagnoses - main ICD10: G56 Mononeuropathies of upper limb || id:UKB-a:528	Weighted median	5	-1.227	4.071	0.763
Diagnoses - main ICD10: G56 Mononeuropathies of upper limb || id:UKB-a:528	Inverse variance weighted	5	2.080	3.715	0.576
Diagnoses - main ICD10: H25 Senile cataract || id:UKB-a:529	Inverse variance weighted	2	-9.048	7.272	0.213
Diagnoses - main ICD10: H26 Other cataract || id:UKB-a:530	MR Egger	3	-37.503	29.145	0.421
Diagnoses - main ICD10: H26 Other cataract || id:UKB-a:530	Weighted median	3	5.208	5.168	0.314
Diagnoses - main ICD10: H26 Other cataract || id:UKB-a:530	Inverse variance weighted	3	1.413	4.529	0.755
Diagnoses - main ICD10: I10 Essential (primary) hypertension || id:UKB-a:531	Inverse variance weighted	2	15.196	19.369	0.433
Diagnoses - main ICD10: I20 Angina pectoris || id:UKB-a:532	Inverse variance weighted	2	12.132	6.544	0.064
Diagnoses - main ICD10: I21 Acute myocardial infarction || id:UKB-a:533	MR Egger	3	11.670	14.136	0.561
Diagnoses - main ICD10: I21 Acute myocardial infarction || id:UKB-a:533	Weighted median	3	7.531	5.570	0.176
Diagnoses - main ICD10: I21 Acute myocardial infarction || id:UKB-a:533	Inverse variance weighted	3	8.117	4.997	0.104
Diagnoses - main ICD10: I25 Chronic ischaemic heart disease || id:UKB-a:534	MR Egger	15	5.761	2.446	0.035
Diagnoses - main ICD10: I25 Chronic ischaemic heart disease || id:UKB-a:534	Weighted median	15	2.939	1.534	0.055
Diagnoses - main ICD10: I25 Chronic ischaemic heart disease || id:UKB-a:534	Inverse variance weighted	15	2.657	1.187	0.025
Diagnoses - main ICD10: I30 Acute pericarditis || id:UKB-a:535	MR Egger	7	23.946	58.626	0.700
Diagnoses - main ICD10: I30 Acute pericarditis || id:UKB-a:535	Weighted median	7	23.269	28.904	0.421
Diagnoses - main ICD10: I30 Acute pericarditis || id:UKB-a:535	Inverse variance weighted	7	28.998	23.478	0.217
Diagnoses - main ICD10: I48 Atrial fibrillation and flutter || id:UKB-a:536	MR Egger	13	0.013	3.604	0.997
Diagnoses - main ICD10: I48 Atrial fibrillation and flutter || id:UKB-a:536	Weighted median	13	1.335	2.322	0.565
Diagnoses - main ICD10: I48 Atrial fibrillation and flutter || id:UKB-a:536	Inverse variance weighted	13	1.387	1.651	0.401
Diagnoses - main ICD10: I80 Phlebitis and thrombophlebitis || id:UKB-a:537	MR Egger	6	-9.303	4.254	0.094
Diagnoses - main ICD10: I80 Phlebitis and thrombophlebitis || id:UKB-a:537	Weighted median	6	-6.857	3.396	0.043
Diagnoses - main ICD10: I80 Phlebitis and thrombophlebitis || id:UKB-a:537	Inverse variance weighted	6	-3.540	3.151	0.261
Diagnoses - main ICD10: I83 Varicose veins of lower extremities || id:UKB-a:538	MR Egger	15	-3.481	5.059	0.503
Diagnoses - main ICD10: I83 Varicose veins of lower extremities || id:UKB-a:538	Weighted median	15	-0.945	1.928	0.624
Diagnoses - main ICD10: I83 Varicose veins of lower extremities || id:UKB-a:538	Inverse variance weighted	15	-0.885	1.493	0.553
Diagnoses - main ICD10: I84 Haemorrhoids || id:UKB-a:539	Inverse variance weighted	2	4.679	4.440	0.292
Diagnoses - main ICD10: J33 Nasal polyp || id:UKB-a:541	MR Egger	7	-9.827	18.578	0.619
Diagnoses - main ICD10: J33 Nasal polyp || id:UKB-a:541	Weighted median	7	-2.850	6.212	0.646
Diagnoses - main ICD10: J33 Nasal polyp || id:UKB-a:541	Inverse variance weighted	7	-3.232	5.011	0.519
Diagnoses - main ICD10: J44 Other chronic obstructive pulmonary disease || id:UKB-a:543	Inverse variance weighted	2	-12.697	24.726	0.608
Diagnoses - main ICD10: K40 Inguinal hernia || id:UKB-a:549	MR Egger	10	1.589	5.556	0.782
Diagnoses - main ICD10: K40 Inguinal hernia || id:UKB-a:549	Weighted median	10	0.599	2.034	0.769
Diagnoses - main ICD10: K40 Inguinal hernia || id:UKB-a:549	Inverse variance weighted	10	-1.415	1.515	0.350
Diagnoses - main ICD10: K50 Crohn's disease [regional enteritis] || id:UKB-a:552	MR Egger	3	35.052	27.117	0.419
Diagnoses - main ICD10: K50 Crohn's disease [regional enteritis] || id:UKB-a:552	Weighted median	3	14.284	13.787	0.300

Diagnoses - main ICD10: K50 Crohn's disease [regional enteritis] || id:UKB-a:552	Inverse variance weighted	3	22.723	12.279	0.064
Diagnoses - main ICD10: K51 Ulcerative colitis || id:UKB-a:553	Inverse variance weighted	2	1.466	10.061	0.884
Diagnoses - main ICD10: K57 Diverticular disease of intestine || id:UKB-a:555	Inverse variance weighted	2	1.992	4.844	0.681
Diagnoses - main ICD10: K76 Other diseases of liver || id:UKB-a:558	MR Egger	4	54.006	28.332	0.197
Diagnoses - main ICD10: K76 Other diseases of liver || id:UKB-a:558	Weighted median	4	28.738	21.079	0.173
Diagnoses - main ICD10: K76 Other diseases of liver || id:UKB-a:558	Inverse variance weighted	4	20.924	17.037	0.219
Diagnoses - main ICD10: K80 Cholelithiasis || id:UKB-a:559	MR Egger	11	0.276	1.734	0.877
Diagnoses - main ICD10: K80 Cholelithiasis || id:UKB-a:559	Weighted median	11	-0.365	1.348	0.787
Diagnoses - main ICD10: K80 Cholelithiasis || id:UKB-a:559	Inverse variance weighted	11	-0.833	1.146	0.467
Diagnoses - main ICD10: L03 Cellulitis || id:UKB-a:560	MR Egger	3	8.664	14.060	0.648
Diagnoses - main ICD10: L03 Cellulitis || id:UKB-a:560	Weighted median	3	-1.421	6.886	0.837
Diagnoses - main ICD10: L03 Cellulitis || id:UKB-a:560	Inverse variance weighted	3	0.454	5.939	0.939
Diagnoses - main ICD10: M10 Gout || id:UKB-a:561	MR Egger	5	-97.168	192.483	0.648
Diagnoses - main ICD10: M10 Gout || id:UKB-a:561	Weighted median	5	-31.458	33.493	0.348
Diagnoses - main ICD10: M10 Gout || id:UKB-a:561	Inverse variance weighted	5	-1.925	36.716	0.958
Diagnoses - main ICD10: M17 Gonarthrosis [arthrosis of knee] || id:UKB-a:563	Inverse variance weighted	2	-6.931	5.484	0.206
Diagnoses - main ICD10: M21 Other acquired deformities of limbs || id:UKB-a:565	MR Egger	6	-4.412	20.959	0.844
Diagnoses - main ICD10: M21 Other acquired deformities of limbs || id:UKB-a:565	Weighted median	6	1.377	14.508	0.924
Diagnoses - main ICD10: M21 Other acquired deformities of limbs || id:UKB-a:565	Inverse variance weighted	6	-4.609	11.598	0.691
Diagnoses - main ICD10: M72 Fibroblastic disorders || id:UKB-a:572	MR Egger	19	-4.174	3.419	0.239
Diagnoses - main ICD10: M72 Fibroblastic disorders || id:UKB-a:572	Weighted median	19	-4.459	2.692	0.098
Diagnoses - main ICD10: M72 Fibroblastic disorders || id:UKB-a:572	Inverse variance weighted	19	-3.142	1.850	0.089
Diagnoses - main ICD10: N19 Unspecified renal failure || id:UKB-a:573	MR Egger	7	27.974	82.434	0.748
Diagnoses - main ICD10: N19 Unspecified renal failure || id:UKB-a:573	Weighted median	7	46.293	29.362	0.115
Diagnoses - main ICD10: N19 Unspecified renal failure || id:UKB-a:573	Inverse variance weighted	7	33.163	23.846	0.164
Diagnoses - main ICD10: N20 Calculus of kidney and ureter || id:UKB-a:574	MR Egger	3	0.008	133.939	1.000
Diagnoses - main ICD10: N20 Calculus of kidney and ureter || id:UKB-a:574	Weighted median	3	-11.283	7.804	0.148
Diagnoses - main ICD10: N20 Calculus of kidney and ureter || id:UKB-a:574	Inverse variance weighted	3	-8.723	6.444	0.176
Diagnoses - main ICD10: N40 Hyperplasia of prostate || id:UKB-a:576	MR Egger	7	4.863	19.065	0.809
Diagnoses - main ICD10: N40 Hyperplasia of prostate || id:UKB-a:576	Weighted median	7	6.032	4.672	0.197
Diagnoses - main ICD10: N40 Hyperplasia of prostate || id:UKB-a:576	Inverse variance weighted	7	2.881	4.418	0.514
Diagnoses - main ICD10: N81 Female genital prolapse || id:UKB-a:577	MR Egger	3	14.670	19.409	0.588
Diagnoses - main ICD10: N81 Female genital prolapse || id:UKB-a:577	Weighted median	3	0.333	5.290	0.950
Diagnoses - main ICD10: N81 Female genital prolapse || id:UKB-a:577	Inverse variance weighted	3	1.002	4.505	0.824
Diagnoses - main ICD10: O75 Other complications of labour and delivery not elsewhere classified || id:UKB-a:579	MR Egger	8	98.556	227.274	0.680
Diagnoses - main ICD10: O75 Other complications of labour and delivery not elsewhere classified || id:UKB-a:579	Weighted median	8	11.430	24.430	0.640
Diagnoses - main ICD10: O75 Other complications of labour and delivery not elsewhere classified || id:UKB-a:579	Inverse variance weighted	8	12.997	20.047	0.517
Diagnoses - main ICD10: R31 Unspecified haematuria || id:UKB-a:585	MR Egger	3	-4.988	22.213	0.859
Diagnoses - main ICD10: R31 Unspecified haematuria || id:UKB-a:585	Weighted median	3	3.847	4.397	0.382
Diagnoses - main ICD10: R31 Unspecified haematuria || id:UKB-a:585	Inverse variance weighted	3	4.126	4.844	0.394
Diastolic blood pressure automated reading || id:UKB-a:359	MR Egger	176	-0.051	0.224	0.821
Diastolic blood pressure automated reading || id:UKB-a:359	Weighted median	176	-0.097	0.091	0.284

Diastolic blood pressure automated reading || id:UKB-a:359	Inverse variance weighted	176	-0.042	0.062	0.502
Difficulty not smoking for 1 day || id:UKB-a:344	MR Egger	4	0.040	0.414	0.933
Difficulty not smoking for 1 day || id:UKB-a:344	Weighted median	4	-0.156	0.131	0.236
Difficulty not smoking for 1 day || id:UKB-a:344	Inverse variance weighted	4	-0.135	0.114	0.234
Dihomo-gamma-linolenic acid (20:3n6) || id:1140	MR Egger	150	-0.007	0.007	0.309
Dihomo-gamma-linolenic acid (20:3n6) || id:1140	Weighted median	150	0.001	0.005	0.913
Dihomo-gamma-linolenic acid (20:3n6) || id:1140	Inverse variance weighted	150	0.006	0.003	0.033
Dihomo-linolenate (20:3n3 or n6) || id:712	Inverse variance weighted	2	-0.397	0.473	0.401
Docosapentaenoic acid (22:5n3) || id:1144	MR Egger	3	-0.174	0.345	0.703
Docosapentaenoic acid (22:5n3) || id:1144	Weighted median	3	-0.026	0.170	0.879
Docosapentaenoic acid (22:5n3) || id:1144	Inverse variance weighted	3	-0.003	0.165	0.985
Doctor diagnosed asthma || id:UKB-a:255	MR Egger	20	0.481	1.015	0.641
Doctor diagnosed asthma || id:UKB-a:255	Weighted median	20	0.206	0.384	0.592
Doctor diagnosed asthma || id:UKB-a:255	Inverse variance weighted	20	0.164	0.274	0.550
Doctor diagnosed hayfever or allergic rhinitis || id:UKB-a:254	MR Egger	18	0.941	0.990	0.356
Doctor diagnosed hayfever or allergic rhinitis || id:UKB-a:254	Weighted median	18	0.029	0.327	0.929
Doctor diagnosed hayfever or allergic rhinitis || id:UKB-a:254	Inverse variance weighted	18	0.094	0.312	0.762
Drive faster than motorway speed limit || id:UKB-a:8	MR Egger	12	0.703	2.114	0.746
Drive faster than motorway speed limit || id:UKB-a:8	Weighted median	12	-0.653	0.428	0.127
Drive faster than motorway speed limit || id:UKB-a:8	Inverse variance weighted	12	-0.755	0.368	0.040
Duration of vigorous activity || id:UKB-a:512	Inverse variance weighted	2	-0.594	0.552	0.282
Duration of walks || id:UKB-a:507	MR Egger	4	5.119	7.222	0.552
Duration of walks || id:UKB-a:507	Weighted median	4	1.626	0.617	0.008
Duration of walks || id:UKB-a:507	Inverse variance weighted	4	0.826	0.773	0.285
Eicosapentaenoic acid (20:5n3) || id:1147	MR Egger	4	0.084	0.143	0.617
Eicosapentaenoic acid (20:5n3) || id:1147	Weighted median	4	0.111	0.074	0.135
Eicosapentaenoic acid (20:5n3) || id:1147	Inverse variance weighted	4	0.099	0.068	0.144
Epiandrosterone sulfate || id:627	Inverse variance weighted	2	-0.086	0.350	0.807
Erythronate* || id:578	MR Egger	3	-1.477	5.724	0.839
Erythronate* || id:578	Weighted median	3	0.172	0.821	0.834
Erythronate* || id:578	Inverse variance weighted	3	0.030	0.729	0.967
Ever depressed for a whole week || id:UKB-a:373	MR Egger	4	1.273	3.241	0.732
Ever depressed for a whole week || id:UKB-a:373	Weighted median	4	0.685	0.776	0.377
Ever depressed for a whole week || id:UKB-a:373	Inverse variance weighted	4	0.125	0.712	0.860
Ever smoked || id:UKB-a:236	MR Egger	37	-0.736	1.409	0.605
Ever smoked || id:UKB-a:236	Weighted median	37	0.028	0.434	0.948
Ever smoked || id:UKB-a:236	Inverse variance weighted	37	0.348	0.299	0.245
Ever used hormone-replacement therapy (HRT) || id:UKB-a:324	MR Egger	5	-0.393	1.217	0.768
Ever used hormone-replacement therapy (HRT) || id:UKB-a:324	Weighted median	5	0.396	0.699	0.571
Ever used hormone-replacement therapy (HRT) || id:UKB-a:324	Inverse variance weighted	5	0.399	0.572	0.486
Extreme body mass index || id:85	MR Egger	7	-0.045	0.190	0.822
Extreme body mass index || id:85	Weighted median	7	-0.086	0.035	0.013

Extreme body mass index || id:85	Inverse variance weighted	7	-0.064	0.037	0.088
Extreme height || id:86	MR Egger	45	-0.026	0.052	0.626
Extreme height || id:86	Weighted median	45	0.005	0.017	0.763
Extreme height || id:86	Inverse variance weighted	45	0.000	0.011	0.994
Extreme waist-to-hip ratio || id:87	Inverse variance weighted	2	0.005	0.051	0.918
Falls in the last year || id:UKB-a:262	MR Egger	3	22.693	10.924	0.286
Falls in the last year || id:UKB-a:262	Weighted median	3	2.131	1.538	0.166
Falls in the last year || id:UKB-a:262	Inverse variance weighted	3	1.531	1.711	0.371
Fed-up feelings || id:UKB-a:49	MR Egger	31	1.365	2.653	0.611
Fed-up feelings || id:UKB-a:49	Weighted median	31	0.110	0.538	0.839
Fed-up feelings || id:UKB-a:49	Inverse variance weighted	31	0.251	0.424	0.554
Ferritin || id:1050	MR Egger	4	-0.210	0.191	0.387
Ferritin || id:1050	Weighted median	4	0.021	0.120	0.860
Ferritin || id:1050	Inverse variance weighted	4	0.031	0.098	0.753
Fluid intelligence score || id:UKB-a:196	MR Egger	42	-0.056	0.234	0.813
Fluid intelligence score || id:UKB-a:196	Weighted median	42	-0.074	0.058	0.205
Fluid intelligence score || id:UKB-a:196	Inverse variance weighted	42	-0.057	0.052	0.273
Forced expiratory volume in 1-second (FEV1) Best measure || id:UKB-a:231	MR Egger	141	0.628	0.276	0.024
Forced expiratory volume in 1-second (FEV1) Best measure || id:UKB-a:231	Weighted median	141	0.098	0.110	0.373
Forced expiratory volume in 1-second (FEV1) Best measure || id:UKB-a:231	Inverse variance weighted	141	0.094	0.083	0.258
Forced expiratory volume in 1-second (FEV1) predicted || id:UKB-a:234	MR Egger	80	0.289	0.320	0.370
Forced expiratory volume in 1-second (FEV1) predicted || id:UKB-a:234	Weighted median	80	0.039	0.124	0.754
Forced expiratory volume in 1-second (FEV1) predicted || id:UKB-a:234	Inverse variance weighted	80	0.065	0.091	0.477
Forced expiratory volume in 1-second (FEV1) predicted percentage || id:UKB-a:235	MR Egger	52	-0.169	0.410	0.683
Forced expiratory volume in 1-second (FEV1) predicted percentage || id:UKB-a:235	Weighted median	52	-0.042	0.106	0.694
Forced expiratory volume in 1-second (FEV1) predicted percentage || id:UKB-a:235	Inverse variance weighted	52	-0.026	0.089	0.772
Forced expiratory volume in 1-second (FEV1) || id:UKB-a:337	MR Egger	155	0.705	0.285	0.014
Forced expiratory volume in 1-second (FEV1) || id:UKB-a:337	Weighted median	155	0.025	0.115	0.830
Forced expiratory volume in 1-second (FEV1) || id:UKB-a:337	Inverse variance weighted	155	-0.009	0.087	0.921
Forced vital capacity (FVC) Best measure || id:UKB-a:232	MR Egger	190	0.377	0.206	0.069
Forced vital capacity (FVC) Best measure || id:UKB-a:232	Weighted median	190	0.021	0.099	0.830
Forced vital capacity (FVC) Best measure || id:UKB-a:232	Inverse variance weighted	190	0.149	0.073	0.040
Fractured/broken bones in last 5 years || id:UKB-a:308	MR Egger	6	-3.191	2.594	0.286
Fractured/broken bones in last 5 years || id:UKB-a:308	Weighted median	6	1.349	1.212	0.266
Fractured/broken bones in last 5 years || id:UKB-a:308	Inverse variance weighted	6	1.059	0.988	0.284
Free cholesterol || id:858	MR Egger	12	-0.061	0.081	0.471
Free cholesterol || id:858	Weighted median	12	0.060	0.050	0.227
Free cholesterol || id:858	Inverse variance weighted	12	0.050	0.038	0.181
Free cholesterol in IDL || id:868	MR Egger	18	0.003	0.052	0.960
Free cholesterol in IDL || id:868	Weighted median	18	0.049	0.043	0.249
Free cholesterol in IDL || id:868	Inverse variance weighted	18	0.048	0.032	0.133
Free cholesterol in large HDL || id:876	MR Egger	16	0.072	0.098	0.476

Free cholesterol in large HDL || id:876	Weighted median	16	-0.031	0.049	0.524
Free cholesterol in large HDL || id:876	Inverse variance weighted	16	-0.081	0.043	0.060
Free cholesterol in large LDL || id:882	MR Egger	18	-0.003	0.046	0.950
Free cholesterol in large LDL || id:882	Weighted median	18	0.045	0.041	0.275
Free cholesterol in large LDL || id:882	Inverse variance weighted	18	0.064	0.033	0.053
Free cholesterol in large VLDL || id:888	MR Egger	10	0.010	0.149	0.947
Free cholesterol in large VLDL || id:888	Weighted median	10	0.108	0.065	0.097
Free cholesterol in large VLDL || id:888	Inverse variance weighted	10	0.088	0.050	0.080
Free cholesterol in medium HDL || id:900	MR Egger	5	-0.006	0.187	0.975
Free cholesterol in medium HDL || id:900	Weighted median	5	-0.098	0.085	0.248
Free cholesterol in medium HDL || id:900	Inverse variance weighted	5	-0.111	0.073	0.128
Free cholesterol to esterified cholesterol ratio || id:853	MR Egger	13	0.008	0.062	0.895
Free cholesterol to esterified cholesterol ratio || id:853	Weighted median	13	0.061	0.049	0.211
Free cholesterol to esterified cholesterol ratio || id:853	Inverse variance weighted	13	0.050	0.036	0.166
Frequency of depressed mood in last 2 weeks || id:UKB-a:242	MR Egger	7	-1.126	4.011	0.790
Frequency of depressed mood in last 2 weeks || id:UKB-a:242	Weighted median	7	1.315	0.814	0.107
Frequency of depressed mood in last 2 weeks || id:UKB-a:242	Inverse variance weighted	7	0.094	0.753	0.901
Frequency of stair climbing in last 4 weeks || id:UKB-a:514	MR Egger	5	10.899	6.287	0.181
Frequency of stair climbing in last 4 weeks || id:UKB-a:514	Weighted median	5	-1.503	0.524	0.004
Frequency of stair climbing in last 4 weeks || id:UKB-a:514	Inverse variance weighted	5	-0.770	0.596	0.196
Frequency of tenseness / restlessness in last 2 weeks || id:UKB-a:244	MR Egger	7	-0.626	1.557	0.704
Frequency of tenseness / restlessness in last 2 weeks || id:UKB-a:244	Weighted median	7	-0.046	0.841	0.957
Frequency of tenseness / restlessness in last 2 weeks || id:UKB-a:244	Inverse variance weighted	7	0.521	0.623	0.403
Frequency of tiredness / lethargy in last 2 weeks || id:UKB-a:245	MR Egger	20	-0.145	0.905	0.875
Frequency of tiredness / lethargy in last 2 weeks || id:UKB-a:245	Weighted median	20	-0.010	0.359	0.977
Frequency of tiredness / lethargy in last 2 weeks || id:UKB-a:245	Inverse variance weighted	20	-0.158	0.266	0.554
Frequency of unenthusiasm / disinterest in last 2 weeks || id:UKB-a:243	MR Egger	4	3.739	4.246	0.471
Frequency of unenthusiasm / disinterest in last 2 weeks || id:UKB-a:243	Weighted median	4	0.916	0.897	0.307
Frequency of unenthusiasm / disinterest in last 2 weeks || id:UKB-a:243	Inverse variance weighted	4	0.888	0.726	0.221
Frequency of walking for pleasure in last 4 weeks || id:UKB-a:515	Inverse variance weighted	2	-0.262	0.381	0.492
Gamma-glutamylglutamine || id:359	MR Egger	3	-0.493	1.877	0.836
Gamma-glutamylglutamine || id:359	Weighted median	3	-0.649	0.592	0.273
Gamma-glutamylglutamine || id:359	Inverse variance weighted	3	-0.642	0.546	0.240
Gamma-glutamylphenylalanine || id:572	Inverse variance weighted	2	0.579	0.990	0.558
Gamma-glutamyltyrosine || id:360	MR Egger	5	-2.106	4.719	0.686
Gamma-glutamyltyrosine || id:360	Weighted median	5	0.792	0.784	0.312
Gamma-glutamyltyrosine || id:360	Inverse variance weighted	5	1.059	0.626	0.091
Gamma-linolenic acid (18:3n6) || id:1139	MR Egger	369	-0.045	0.044	0.302
Gamma-linolenic acid (18:3n6) || id:1139	Weighted median	369	-0.007	0.028	0.812
Gamma-linolenic acid (18:3n6) || id:1139	Inverse variance weighted	369	0.029	0.017	0.089
Getting up in morning || id:UKB-a:10	MR Egger	37	0.546	0.632	0.393
Getting up in morning || id:UKB-a:10	Weighted median	37	-0.214	0.283	0.450

Getting up in morning || id:UKB-a:10	Inverse variance weighted	37	-0.236	0.194	0.223
Glucose || id:859	MR Egger	4	-0.102	0.310	0.774
Glucose || id:859	Weighted median	4	0.188	0.100	0.062
Glucose || id:859	Inverse variance weighted	4	0.080	0.092	0.384
Glutamine || id:860	MR Egger	5	-0.134	0.172	0.492
Glutamine || id:860	Weighted median	5	-0.065	0.074	0.383
Glutamine || id:860	Inverse variance weighted	5	-0.006	0.087	0.943
Glutaroyl carnitine || id:699	MR Egger	8	0.293	0.850	0.743
Glutaroyl carnitine || id:699	Weighted median	8	0.148	0.263	0.575
Glutaroyl carnitine || id:699	Inverse variance weighted	8	0.136	0.208	0.514
Glycoprotein acetyls || id:863	MR Egger	7	0.111	0.224	0.642
Glycoprotein acetyls || id:863	Weighted median	7	0.120	0.088	0.170
Glycoprotein acetyls || id:863	Inverse variance weighted	7	0.120	0.069	0.082
Glycoproteins || id:862	MR Egger	10	-0.002	0.042	0.971
Glycoproteins || id:862	Weighted median	10	-0.005	0.030	0.882
Glycoproteins || id:862	Inverse variance weighted	10	0.002	0.027	0.942
Gout || id:1054	Inverse variance weighted	2	-0.009	0.076	0.902
Guilty feelings || id:UKB-a:240	MR Egger	16	-2.985	4.620	0.529
Guilty feelings || id:UKB-a:240	Weighted median	16	-0.145	0.777	0.852
Guilty feelings || id:UKB-a:240	Inverse variance weighted	16	-0.545	0.730	0.455
Had menopause || id:UKB-a:316	MR Egger	9	-1.264	0.991	0.243
Had menopause || id:UKB-a:316	Weighted median	9	-0.394	0.531	0.458
Had menopause || id:UKB-a:316	Inverse variance weighted	9	-0.034	0.408	0.934
Haemoglobin concentration || id:270	MR Egger	15	0.236	0.295	0.438
Haemoglobin concentration || id:270	Weighted median	15	-0.039	0.109	0.718
Haemoglobin concentration || id:270	Inverse variance weighted	15	-0.140	0.110	0.205
Hand grip strength (left) || id:UKB-a:374	MR Egger	83	-0.093	0.542	0.864
Hand grip strength (left) || id:UKB-a:374	Weighted median	83	0.209	0.189	0.270
Hand grip strength (left) || id:UKB-a:374	Inverse variance weighted	83	0.283	0.132	0.032
HDL cholesterol || id:299	MR Egger	86	-0.185	0.083	0.029
HDL cholesterol || id:299	Weighted median	86	-0.039	0.061	0.523
HDL cholesterol || id:299	Inverse variance weighted	86	0.005	0.046	0.908
Headaches for 3+ months || id:UKB-a:354	Inverse variance weighted	2	0.516	1.833	0.778
Hearing difficulty/problems with background noise || id:UKB-a:261	MR Egger	15	-0.296	3.048	0.924
Hearing difficulty/problems with background noise || id:UKB-a:261	Weighted median	15	-0.045	0.721	0.950
Hearing difficulty/problems with background noise || id:UKB-a:261	Inverse variance weighted	15	-0.302	0.531	0.569
Hearing difficulty/problems: Yes || id:UKB-a:257	MR Egger	23	-2.349	1.278	0.080
Hearing difficulty/problems: Yes || id:UKB-a:257	Weighted median	23	-0.712	0.549	0.194
Hearing difficulty/problems: Yes || id:UKB-a:257	Inverse variance weighted	23	-0.510	0.390	0.191
Heart rate || id:1056	MR Egger	15	-0.006	0.035	0.871
Heart rate || id:1056	Weighted median	15	-0.002	0.012	0.891
Heart rate || id:1056	Inverse variance weighted	15	-0.006	0.009	0.469

Heel bone mineral density (BMD) T-score automated (left) || id:UKB-a:361	MR Egger	136	0.040	0.071	0.572
Heel bone mineral density (BMD) T-score automated (left) || id:UKB-a:361	Weighted median	136	-0.005	0.057	0.927
Heel bone mineral density (BMD) T-score automated (left) || id:UKB-a:361	Inverse variance weighted	136	0.060	0.033	0.070
Heel bone mineral density (BMD) T-score automated (right) || id:UKB-a:362	MR Egger	137	0.037	0.069	0.589
Heel bone mineral density (BMD) T-score automated (right) || id:UKB-a:362	Weighted median	137	-0.011	0.055	0.837
Heel bone mineral density (BMD) T-score automated (right) || id:UKB-a:362	Inverse variance weighted	137	0.052	0.033	0.115
Height || id:1034	MR Egger	4	3.197	2.131	0.272
Height || id:1034	Weighted median	4	0.004	0.108	0.969
Height || id:1034	Inverse variance weighted	4	-0.023	0.106	0.831
Heptanoate (7:0) || id:349	Inverse variance weighted	2	0.254	0.953	0.790
Hexadecanedioate || id:711	MR Egger	3	-0.194	0.584	0.796
Hexadecanedioate || id:711	Weighted median	3	0.056	0.177	0.752
Hexadecanedioate || id:711	Inverse variance weighted	3	0.105	0.165	0.525
Hexanoylcarnitine || id:467	MR Egger	4	-0.058	0.701	0.942
Hexanoylcarnitine || id:467	Weighted median	4	0.181	0.193	0.347
Hexanoylcarnitine || id:467	Inverse variance weighted	4	0.307	0.320	0.338
High grade serous ovarian cancer || id:1121	MR Egger	13	-0.001	0.064	0.991
High grade serous ovarian cancer || id:1121	Weighted median	13	-0.006	0.039	0.883
High grade serous ovarian cancer || id:1121	Inverse variance weighted	13	-0.008	0.029	0.787
Hip circumference || id:100	Inverse variance weighted	2	-0.247	0.201	0.219
Hip circumference || id:101	MR Egger	5	-0.996	1.426	0.535
Hip circumference || id:101	Weighted median	5	-0.100	0.168	0.553
Hip circumference || id:101	Inverse variance weighted	5	-0.201	0.139	0.149
Hip circumference || id:48	MR Egger	48	0.027	0.305	0.930
Hip circumference || id:48	Weighted median	48	0.033	0.122	0.786
Hip circumference || id:48	Inverse variance weighted	48	0.022	0.082	0.789
Hip circumference || id:49	MR Egger	52	-0.283	0.219	0.202
Hip circumference || id:49	Weighted median	52	-0.086	0.111	0.442
Hip circumference || id:49	Inverse variance weighted	52	-0.041	0.073	0.577
Hip circumference || id:50	MR Egger	24	-0.208	0.368	0.578
Hip circumference || id:50	Weighted median	24	-0.135	0.134	0.313
Hip circumference || id:50	Inverse variance weighted	24	-0.044	0.097	0.651
Hip circumference || id:51	MR Egger	17	-0.275	0.460	0.558
Hip circumference || id:51	Weighted median	17	-0.048	0.161	0.767
Hip circumference || id:51	Inverse variance weighted	17	-0.046	0.128	0.720
Hip circumference || id:52	MR Egger	19	-1.114	0.584	0.073
Hip circumference || id:52	Weighted median	19	0.030	0.142	0.833
Hip circumference || id:52	Inverse variance weighted	19	0.046	0.105	0.665
Hip circumference || id:53	MR Egger	15	-1.226	0.645	0.080
Hip circumference || id:53	Weighted median	15	0.021	0.147	0.885
Hip circumference || id:53	Inverse variance weighted	15	0.038	0.108	0.722
Hip circumference || id:54	MR Egger	74	-0.362	0.243	0.140

Hip circumference || id:54	Weighted median	74	-0.048	0.087	0.584
Hip circumference || id:54	Inverse variance weighted	74	0.001	0.063	0.986
Hip circumference || id:55	MR Egger	73	-0.172	0.223	0.445
Hip circumference || id:55	Weighted median	73	-0.072	0.085	0.396
Hip circumference || id:55	Inverse variance weighted	73	-0.042	0.060	0.477
Hip circumference || id:56	MR Egger	40	-0.139	0.276	0.617
Hip circumference || id:56	Weighted median	40	-0.071	0.095	0.453
Hip circumference || id:56	Inverse variance weighted	40	-0.078	0.067	0.246
Hip circumference || id:57	MR Egger	38	-0.209	0.287	0.471
Hip circumference || id:57	Weighted median	38	-0.158	0.102	0.119
Hip circumference || id:57	Inverse variance weighted	38	-0.104	0.068	0.127
Hip circumference || id:58	MR Egger	30	-0.178	0.332	0.597
Hip circumference || id:58	Weighted median	30	-0.049	0.102	0.632
Hip circumference || id:58	Inverse variance weighted	30	-0.057	0.070	0.413
Hip circumference || id:59	MR Egger	30	-0.282	0.321	0.388
Hip circumference || id:59	Weighted median	30	-0.071	0.097	0.464
Hip circumference || id:59	Inverse variance weighted	30	-0.076	0.070	0.273
Hippocampus volume || id:1045	Inverse variance weighted	2	0.000	0.000	0.379
Histidine || id:866	MR Egger	5	0.606	0.798	0.503
Histidine || id:866	Weighted median	5	0.057	0.116	0.622
Histidine || id:866	Inverse variance weighted	5	0.001	0.132	0.995
Home area population density - urban or rural: Scotland - Large Urban Area || id:UKB-a:228	MR Egger	250	1.318	0.670	0.050
Home area population density - urban or rural: Scotland - Large Urban Area || id:UKB-a:228	Weighted median	250	-0.076	0.456	0.868
Home area population density - urban or rural: Scotland - Large Urban Area || id:UKB-a:228	Inverse variance weighted	250	-0.089	0.315	0.777
HWESASXX* || id:526	Inverse variance weighted	2	-0.208	0.248	0.401
Hydroxyisovaleroyl carnitine || id:698	Inverse variance weighted	2	-0.419	0.400	0.295
Illnesses of father: Chronic bronchitis/emphysema || id:UKB-a:206	Inverse variance weighted	2	-2.456	1.845	0.183
Illnesses of father: Diabetes || id:UKB-a:208	MR Egger	14	-0.009	1.559	0.995
Illnesses of father: Diabetes || id:UKB-a:208	Weighted median	14	-0.498	0.888	0.575
Illnesses of father: Diabetes || id:UKB-a:208	Inverse variance weighted	14	-1.239	0.650	0.057
Illnesses of father: Heart disease || id:UKB-a:201	MR Egger	18	1.884	1.005	0.079
Illnesses of father: Heart disease || id:UKB-a:201	Weighted median	18	0.994	0.505	0.049
Illnesses of father: Heart disease || id:UKB-a:201	Inverse variance weighted	18	0.398	0.464	0.391
Illnesses of father: High blood pressure || id:UKB-a:207	MR Egger	6	4.511	8.387	0.619
Illnesses of father: High blood pressure || id:UKB-a:207	Weighted median	6	0.893	0.999	0.371
Illnesses of father: High blood pressure || id:UKB-a:207	Inverse variance weighted	6	0.842	0.827	0.309
Illnesses of father: Lung cancer || id:UKB-a:205	MR Egger	3	-18.925	13.534	0.395
Illnesses of father: Lung cancer || id:UKB-a:205	Weighted median	3	-1.286	2.026	0.525
Illnesses of father: Lung cancer || id:UKB-a:205	Inverse variance weighted	3	-0.830	1.672	0.620
Illnesses of father: None of the above (group 1) || id:UKB-a:202	MR Egger	6	-1.024	2.731	0.727
Illnesses of father: None of the above (group 1) || id:UKB-a:202	Weighted median	6	-1.712	0.939	0.068
Illnesses of father: None of the above (group 1) || id:UKB-a:202	Inverse variance weighted	6	-1.335	0.752	0.076

Illnesses of father: None of the above (group 2) || id:UKB-a:203	Inverse variance weighted	2	-0.618	2.234	0.782
Illnesses of father: Prostate cancer || id:UKB-a:204	MR Egger	7	1.797	3.181	0.596
Illnesses of father: Prostate cancer || id:UKB-a:204	Weighted median	7	1.262	1.385	0.362
Illnesses of father: Prostate cancer || id:UKB-a:204	Inverse variance weighted	7	0.468	1.085	0.666
Illnesses of mother: Alzheimer's disease/dementia || id:UKB-a:210	MR Egger	4	1.662	0.667	0.130
Illnesses of mother: Alzheimer's disease/dementia || id:UKB-a:210	Weighted median	4	1.194	0.475	0.012
Illnesses of mother: Alzheimer's disease/dementia || id:UKB-a:210	Inverse variance weighted	4	1.145	0.471	0.015
Illnesses of mother: Breast cancer || id:UKB-a:213	MR Egger	11	-0.523	2.505	0.839
Illnesses of mother: Breast cancer || id:UKB-a:213	Weighted median	11	0.107	1.030	0.917
Illnesses of mother: Breast cancer || id:UKB-a:213	Inverse variance weighted	11	0.078	0.874	0.929
Illnesses of mother: Chronic bronchitis/emphysema || id:UKB-a:214	Inverse variance weighted	2	0.156	4.327	0.971
Illnesses of mother: Diabetes || id:UKB-a:216	MR Egger	13	0.269	1.615	0.871
Illnesses of mother: Diabetes || id:UKB-a:216	Weighted median	13	-0.653	0.904	0.470
Illnesses of mother: Diabetes || id:UKB-a:216	Inverse variance weighted	13	-0.931	0.678	0.169
Illnesses of mother: Heart disease || id:UKB-a:209	MR Egger	4	1.850	1.673	0.384
Illnesses of mother: Heart disease || id:UKB-a:209	Weighted median	4	1.372	1.046	0.190
Illnesses of mother: Heart disease || id:UKB-a:209	Inverse variance weighted	4	0.851	0.906	0.348
Illnesses of mother: High blood pressure || id:UKB-a:215	MR Egger	9	4.353	3.133	0.207
Illnesses of mother: High blood pressure || id:UKB-a:215	Weighted median	9	0.708	0.777	0.362
Illnesses of mother: High blood pressure || id:UKB-a:215	Inverse variance weighted	9	0.897	0.613	0.143
Illnesses of mother: None of the above (group 1) || id:UKB-a:211	MR Egger	4	-1.732	4.274	0.725
Illnesses of mother: None of the above (group 1) || id:UKB-a:211	Weighted median	4	-1.193	1.004	0.235
Illnesses of mother: None of the above (group 1) || id:UKB-a:211	Inverse variance weighted	4	-0.674	1.328	0.612
Illnesses of siblings: Diabetes || id:UKB-a:223	MR Egger	5	2.848	3.461	0.471
Illnesses of siblings: Diabetes || id:UKB-a:223	Weighted median	5	-1.029	1.191	0.387
Illnesses of siblings: Diabetes || id:UKB-a:223	Inverse variance weighted	5	-0.560	1.038	0.589
Illnesses of siblings: Heart disease || id:UKB-a:217	MR Egger	3	0.573	3.371	0.893
Illnesses of siblings: Heart disease || id:UKB-a:217	Weighted median	3	2.050	1.533	0.181
Illnesses of siblings: Heart disease || id:UKB-a:217	Inverse variance weighted	3	2.144	1.373	0.118
Illnesses of siblings: High blood pressure || id:UKB-a:222	MR Egger	12	-0.588	4.449	0.898
Illnesses of siblings: High blood pressure || id:UKB-a:222	Weighted median	12	0.453	0.780	0.562
Illnesses of siblings: High blood pressure || id:UKB-a:222	Inverse variance weighted	12	0.176	0.813	0.828
Illnesses of siblings: None of the above (group 1) || id:UKB-a:218	MR Egger	8	-2.317	5.761	0.701
Illnesses of siblings: None of the above (group 1) || id:UKB-a:218	Weighted median	8	1.395	0.933	0.135
Illnesses of siblings: None of the above (group 1) || id:UKB-a:218	Inverse variance weighted	8	1.676	0.882	0.057
Immunoglobulin G index levels || id:1017	Inverse variance weighted	2	0.002	0.010	0.839
Indoleacetate || id:446	Inverse variance weighted	2	-0.060	0.655	0.927
Inflammatory bowel disease || id:292	MR Egger	112	-0.012	0.030	0.693
Inflammatory bowel disease || id:292	Weighted median	112	-0.019	0.022	0.380
Inflammatory bowel disease || id:292	Inverse variance weighted	112	-0.020	0.013	0.123
Inflammatory bowel disease || id:293	MR Egger	8	0.019	0.037	0.630
Inflammatory bowel disease || id:293	Weighted median	8	0.002	0.019	0.915

Inflammatory bowel disease || id:293	Inverse variance weighted	8	-0.004	0.015	0.795
Inflammatory bowel disease || id:294	MR Egger	129	-0.024	0.035	0.507
Inflammatory bowel disease || id:294	Weighted median	129	-0.017	0.021	0.416
Inflammatory bowel disease || id:294	Inverse variance weighted	129	-0.009	0.015	0.551
Inflammatory bowel disease || id:31	MR Egger	58	0.014	0.038	0.710
Inflammatory bowel disease || id:31	Weighted median	58	-0.020	0.021	0.333
Inflammatory bowel disease || id:31	Inverse variance weighted	58	-0.013	0.014	0.378
Invasive mucinous ovarian cancer || id:1123	Inverse variance weighted	2	-0.010	0.043	0.825
Iron || id:1049	MR Egger	3	0.059	0.188	0.806
Iron || id:1049	Weighted median	3	0.002	0.058	0.969
Iron || id:1049	Inverse variance weighted	3	-0.009	0.071	0.896
Irritability || id:UKB-a:47	MR Egger	26	-0.842	3.542	0.814
Irritability || id:UKB-a:47	Weighted median	26	-0.602	0.607	0.321
Irritability || id:UKB-a:47	Inverse variance weighted	26	-0.627	0.560	0.263
Isobutyrylcarnitine || id:573	MR Egger	3	0.217	0.422	0.698
Isobutyrylcarnitine || id:573	Weighted median	3	-0.293	0.214	0.170
Isobutyrylcarnitine || id:573	Inverse variance weighted	3	-0.318	0.205	0.121
Isovalerylcarnitine || id:652	MR Egger	3	2.904	3.731	0.579
Isovalerylcarnitine || id:652	Weighted median	3	-0.040	0.301	0.894
Isovalerylcarnitine || id:652	Inverse variance weighted	3	-0.119	0.264	0.652
Job involves heavy manual or physical work || id:UKB-a:503	MR Egger	16	-2.362	1.332	0.098
Job involves heavy manual or physical work || id:UKB-a:503	Weighted median	16	0.299	0.298	0.316
Job involves heavy manual or physical work || id:UKB-a:503	Inverse variance weighted	16	0.442	0.269	0.100
Job involves mainly walking or standing || id:UKB-a:502	MR Egger	7	0.791	2.990	0.802
Job involves mainly walking or standing || id:UKB-a:502	Weighted median	7	0.224	0.294	0.446
Job involves mainly walking or standing || id:UKB-a:502	Inverse variance weighted	7	0.336	0.261	0.197
Kynurenine || id:375	MR Egger	4	0.742	1.475	0.665
Kynurenine || id:375	Weighted median	4	0.510	0.412	0.216
Kynurenine || id:375	Inverse variance weighted	4	0.056	0.756	0.941
Lactate || id:894	Inverse variance weighted	2	-0.083	0.257	0.747
Laurate (12:0) || id:350	Inverse variance weighted	2	-0.085	1.029	0.934
LDL cholesterol || id:300	MR Egger	78	0.109	0.056	0.054
LDL cholesterol || id:300	Weighted median	78	0.103	0.052	0.046
LDL cholesterol || id:300	Inverse variance weighted	78	0.111	0.038	0.003
Leg fat percentage (left) || id:UKB-a:278	MR Egger	247	-0.853	0.335	0.012
Leg fat percentage (left) || id:UKB-a:278	Weighted median	247	0.032	0.127	0.801
Leg fat percentage (left) || id:UKB-a:278	Inverse variance weighted	247	0.005	0.092	0.960
Leg fat percentage (right) || id:UKB-a:274	MR Egger	245	-0.741	0.340	0.030
Leg fat percentage (right) || id:UKB-a:274	Weighted median	245	0.015	0.127	0.907
Leg fat percentage (right) || id:UKB-a:274	Inverse variance weighted	245	0.006	0.092	0.950
Length of menstrual cycle || id:UKB-a:351	MR Egger	6	-0.219	0.272	0.466
Length of menstrual cycle || id:UKB-a:351	Weighted median	6	-0.192	0.121	0.112

Length of menstrual cycle || id:UKB-a:351	Inverse variance weighted	6	-0.118	0.109	0.280
Leucine || id:306	MR Egger	12	1.084	4.636	0.820
Leucine || id:306	Weighted median	12	0.531	1.004	0.597
Leucine || id:306	Inverse variance weighted	12	0.379	0.736	0.607
Leucine || id:897	MR Egger	3	0.056	0.649	0.946
Leucine || id:897	Weighted median	3	0.054	0.106	0.607
Leucine || id:897	Inverse variance weighted	3	0.031	0.097	0.749
Levulinate (4-oxovalerate) || id:433	Inverse variance weighted	2	0.057	0.869	0.948
Light smokers at least 100 smokes in lifetime || id:UKB-a:313	MR Egger	4	1.478	1.227	0.352
Light smokers at least 100 smokes in lifetime || id:UKB-a:313	Weighted median	4	0.205	0.450	0.649
Light smokers at least 100 smokes in lifetime || id:UKB-a:313	Inverse variance weighted	4	-0.031	0.409	0.940
Linoleic acid (18:2n6) || id:1142	MR Egger	100	0.002	0.001	0.078
Linoleic acid (18:2n6) || id:1142	Weighted median	100	0.002	0.001	0.061
Linoleic acid (18:2n6) || id:1142	Inverse variance weighted	100	0.001	0.001	0.107
Loneliness isolation || id:UKB-a:239	MR Egger	5	-1.015	5.984	0.876
Loneliness isolation || id:UKB-a:239	Weighted median	5	1.260	1.422	0.376
Loneliness isolation || id:UKB-a:239	Inverse variance weighted	5	1.428	1.083	0.187
Long-standing illness disability or infirmity || id:UKB-a:252	MR Egger	14	4.511	1.837	0.030
Long-standing illness disability or infirmity || id:UKB-a:252	Weighted median	14	0.267	0.729	0.714
Long-standing illness disability or infirmity || id:UKB-a:252	Inverse variance weighted	14	-0.195	0.620	0.753
MDC:%32+ || id:266	MR Egger	206	0.024	0.039	0.537
MDC:%32+ || id:266	Weighted median	206	-0.010	0.015	0.524
MDC:%32+ || id:266	Inverse variance weighted	206	-0.009	0.010	0.369
Mean cell haemoglobin || id:271	MR Egger	30	0.041	0.054	0.453
Mean cell haemoglobin || id:271	Weighted median	30	0.013	0.040	0.756
Mean cell haemoglobin || id:271	Inverse variance weighted	30	-0.016	0.025	0.540
Mean cell haemoglobin concentration || id:272	MR Egger	13	-0.825	1.408	0.570
Mean cell haemoglobin concentration || id:272	Weighted median	13	0.144	0.354	0.684
Mean cell haemoglobin concentration || id:272	Inverse variance weighted	13	0.123	0.268	0.646
Mean cell volume || id:273	MR Egger	38	0.005	0.024	0.850
Mean cell volume || id:273	Weighted median	38	0.004	0.015	0.815
Mean cell volume || id:273	Inverse variance weighted	38	-0.001	0.010	0.925
Mean diameter for HDL particles || id:865	MR Egger	13	0.023	0.082	0.783
Mean diameter for HDL particles || id:865	Weighted median	13	0.002	0.041	0.961
Mean diameter for HDL particles || id:865	Inverse variance weighted	13	-0.020	0.032	0.528
Mean diameter for LDL particles || id:896	MR Egger	8	-0.132	0.136	0.368
Mean diameter for LDL particles || id:896	Weighted median	8	-0.047	0.066	0.475
Mean diameter for LDL particles || id:896	Inverse variance weighted	8	-0.040	0.053	0.452
Mean platelet volume || id:1006	MR Egger	23	-0.255	0.657	0.702
Mean platelet volume || id:1006	Weighted median	23	-0.209	0.392	0.594
Mean platelet volume || id:1006	Inverse variance weighted	23	-0.092	0.284	0.746
Mean time to correctly identify matches || id:UKB-a:199	MR Egger	29	-0.288	1.459	0.845

Mean time to correctly identify matches || id:UKB-a:199	Weighted median	29	0.341	0.247	0.167
Mean time to correctly identify matches || id:UKB-a:199	Inverse variance weighted	29	0.072	0.236	0.761
Medication for cholesterol blood pressure or diabetes: Blood pressure medication || id:UKB-a:490	MR Egger	48	-0.980	0.876	0.269
Medication for cholesterol blood pressure or diabetes: Blood pressure medication || id:UKB-a:490	Weighted median	48	0.062	0.264	0.815
Medication for cholesterol blood pressure or diabetes: Blood pressure medication || id:UKB-a:490	Inverse variance weighted	48	0.051	0.222	0.818
Medication for cholesterol blood pressure or diabetes: Cholesterol lowering medication || id:UKB-a:488	MR Egger	34	0.577	0.399	0.158
Medication for cholesterol blood pressure or diabetes: Cholesterol lowering medication || id:UKB-a:488	Weighted median	34	0.541	0.266	0.042
Medication for cholesterol blood pressure or diabetes: Cholesterol lowering medication || id:UKB-a:488	Inverse variance weighted	34	0.635	0.200	0.002
Medication for cholesterol blood pressure or diabetes: Insulin || id:UKB-a:491	MR Egger	6	11.628	6.027	0.126
Medication for cholesterol blood pressure or diabetes: Insulin || id:UKB-a:491	Weighted median	6	-2.207	2.116	0.297
Medication for cholesterol blood pressure or diabetes: Insulin || id:UKB-a:491	Inverse variance weighted	6	-0.921	2.156	0.669
Medication for cholesterol blood pressure or diabetes: None of the above || id:UKB-a:489	MR Egger	42	-0.844	0.636	0.192
Medication for cholesterol blood pressure or diabetes: None of the above || id:UKB-a:489	Weighted median	42	-0.277	0.273	0.309
Medication for cholesterol blood pressure or diabetes: None of the above || id:UKB-a:489	Inverse variance weighted	42	-0.051	0.198	0.798
Mineral and other dietary supplements: Calcium || id:UKB-a:495	Inverse variance weighted	2	4.646	2.892	0.108
Mineral and other dietary supplements: Fish oil (including cod liver oil) || id:UKB-a:492	Inverse variance weighted	2	-1.475	1.409	0.295
Mineral and other dietary supplements: Glucosamine || id:UKB-a:494	Inverse variance weighted	2	3.879	1.742	0.026
Mineral and other dietary supplements: None of the above || id:UKB-a:493	Inverse variance weighted	2	0.378	1.481	0.798
Morning/evening person (chronotype) || id:UKB-a:11	MR Egger	82	-0.241	0.377	0.524
Morning/evening person (chronotype) || id:UKB-a:11	Weighted median	82	-0.007	0.145	0.959
Morning/evening person (chronotype) || id:UKB-a:11	Inverse variance weighted	82	0.085	0.112	0.448
Multiple sclerosis || id:1024	MR Egger	23	0.000	0.031	0.996
Multiple sclerosis || id:1024	Weighted median	23	-0.002	0.022	0.921
Multiple sclerosis || id:1024	Inverse variance weighted	23	-0.007	0.019	0.724
Multiple sclerosis || id:1025	MR Egger	46	0.001	0.030	0.986
Multiple sclerosis || id:1025	Weighted median	46	-0.012	0.021	0.558
Multiple sclerosis || id:1025	Inverse variance weighted	46	-0.024	0.016	0.140
Myo-inositol || id:417	Inverse variance weighted	2	-1.368	0.700	0.051
N-acetylglycine || id:449	Inverse variance weighted	2	-0.014	0.161	0.933
N2-dimethylguanosine || id:679	Inverse variance weighted	2	0.070	1.087	0.948
Nap during day || id:UKB-a:12	MR Egger	48	0.620	0.971	0.527
Nap during day || id:UKB-a:12	Weighted median	48	0.227	0.316	0.472
Nap during day || id:UKB-a:12	Inverse variance weighted	48	-0.041	0.243	0.865
Nervous feelings || id:UKB-a:50	MR Egger	26	-5.735	3.016	0.069
Nervous feelings || id:UKB-a:50	Weighted median	26	0.423	0.620	0.495
Nervous feelings || id:UKB-a:50	Inverse variance weighted	26	0.079	0.504	0.876
Neuroblastoma || id:816	MR Egger	3	-0.438	0.246	0.326
Neuroblastoma || id:816	Weighted median	3	-0.046	0.035	0.195
Neuroblastoma || id:816	Inverse variance weighted	3	-0.042	0.032	0.189
Neuroticism || id:1007	MR Egger	9	-0.719	2.090	0.741
Neuroticism || id:1007	Weighted median	9	-0.467	0.299	0.118
Neuroticism || id:1007	Inverse variance weighted	9	-0.447	0.240	0.062

Neuroticism score || id:UKB-a:230	MR Egger	64	-0.326	0.184	0.082
Neuroticism score || id:UKB-a:230	Weighted median	64	0.004	0.046	0.929
Neuroticism score || id:UKB-a:230	Inverse variance weighted	64	0.007	0.035	0.841
NKeff:%314-158a+ || id:241	MR Egger	227	-0.033	0.045	0.465
NKeff:%314-158a+ || id:241	Weighted median	227	-0.011	0.014	0.443
NKeff:%314-158a+ || id:241	Inverse variance weighted	227	-0.004	0.010	0.682
Non-cancer illness code self-reported: angina || id:UKB-a:62	MR Egger	13	5.131	3.118	0.128
Non-cancer illness code self-reported: angina || id:UKB-a:62	Weighted median	13	3.342	1.623	0.039
Non-cancer illness code self-reported: angina || id:UKB-a:62	Inverse variance weighted	13	3.212	1.252	0.010
Non-cancer illness code self-reported: ankylosing spondylitis || id:UKB-a:88	Inverse variance weighted	2	-1.188	2.202	0.590
Non-cancer illness code self-reported: asthma || id:UKB-a:66	MR Egger	72	0.506	0.782	0.520
Non-cancer illness code self-reported: asthma || id:UKB-a:66	Weighted median	72	0.299	0.398	0.452
Non-cancer illness code self-reported: asthma || id:UKB-a:66	Inverse variance weighted	72	0.261	0.298	0.381
Non-cancer illness code self-reported: cholelithiasis/gall stones || id:UKB-a:71	MR Egger	12	-0.758	2.523	0.770
Non-cancer illness code self-reported: cholelithiasis/gall stones || id:UKB-a:71	Weighted median	12	-0.499	1.496	0.739
Non-cancer illness code self-reported: cholelithiasis/gall stones || id:UKB-a:71	Inverse variance weighted	12	0.203	1.653	0.902
Non-cancer illness code self-reported: crohns disease || id:UKB-a:103	MR Egger	6	22.233	10.320	0.098
Non-cancer illness code self-reported: crohns disease || id:UKB-a:103	Weighted median	6	4.601	7.687	0.549
Non-cancer illness code self-reported: crohns disease || id:UKB-a:103	Inverse variance weighted	6	13.884	5.931	0.019
Non-cancer illness code self-reported: deep venous thrombosis (dvt) || id:UKB-a:65	MR Egger	9	-3.195	1.989	0.152
Non-cancer illness code self-reported: deep venous thrombosis (dvt) || id:UKB-a:65	Weighted median	9	-2.891	1.403	0.039
Non-cancer illness code self-reported: deep venous thrombosis (dvt) || id:UKB-a:65	Inverse variance weighted	9	-0.858	1.415	0.544
Non-cancer illness code self-reported: diabetes || id:UKB-a:74	MR Egger	36	-1.449	1.534	0.351
Non-cancer illness code self-reported: diabetes || id:UKB-a:74	Weighted median	36	-0.383	0.989	0.699
Non-cancer illness code self-reported: diabetes || id:UKB-a:74	Inverse variance weighted	36	-0.988	0.601	0.100
Non-cancer illness code self-reported: diverticular disease/diverticulitis || id:UKB-a:102	Inverse variance weighted	2	10.280	6.568	0.118
Non-cancer illness code self-reported: enlarged prostate || id:UKB-a:95	MR Egger	7	8.240	17.888	0.664
Non-cancer illness code self-reported: enlarged prostate || id:UKB-a:95	Weighted median	7	2.012	3.839	0.600
Non-cancer illness code self-reported: enlarged prostate || id:UKB-a:95	Inverse variance weighted	7	1.432	3.001	0.633
Non-cancer illness code self-reported: glaucoma || id:UKB-a:79	MR Egger	8	1.440	8.444	0.870
Non-cancer illness code self-reported: glaucoma || id:UKB-a:79	Weighted median	8	-0.415	3.300	0.900
Non-cancer illness code self-reported: glaucoma || id:UKB-a:79	Inverse variance weighted	8	-0.285	2.498	0.909
Non-cancer illness code self-reported: gout || id:UKB-a:107	MR Egger	22	1.473	1.992	0.468
Non-cancer illness code self-reported: gout || id:UKB-a:107	Weighted median	22	1.910	1.473	0.195
Non-cancer illness code self-reported: gout || id:UKB-a:107	Inverse variance weighted	22	-0.234	1.183	0.843
Non-cancer illness code self-reported: hayfever/allergic rhinitis || id:UKB-a:94	MR Egger	20	-0.215	3.408	0.950
Non-cancer illness code self-reported: hayfever/allergic rhinitis || id:UKB-a:94	Weighted median	20	0.078	1.138	0.945
Non-cancer illness code self-reported: hayfever/allergic rhinitis || id:UKB-a:94	Inverse variance weighted	20	-0.162	0.863	0.851
Non-cancer illness code self-reported: heart attack/myocardial infarction || id:UKB-a:63	MR Egger	10	5.550	4.446	0.247
Non-cancer illness code self-reported: heart attack/myocardial infarction || id:UKB-a:63	Weighted median	10	4.303	1.987	0.030
Non-cancer illness code self-reported: heart attack/myocardial infarction || id:UKB-a:63	Inverse variance weighted	10	4.124	1.607	0.010
Non-cancer illness code self-reported: high cholesterol || id:UKB-a:108	MR Egger	49	0.982	0.460	0.038

Non-cancer illness code self-reported: high cholesterol || id:UKB-a:108	Weighted median	49	0.747	0.370	0.044
Non-cancer illness code self-reported: high cholesterol || id:UKB-a:108	Inverse variance weighted	49	0.871	0.243	0.000
Non-cancer illness code self-reported: hypertension || id:UKB-a:61	MR Egger	154	-0.462	0.436	0.290
Non-cancer illness code self-reported: hypertension || id:UKB-a:61	Weighted median	154	0.147	0.226	0.515
Non-cancer illness code self-reported: hypertension || id:UKB-a:61	Inverse variance weighted	154	0.131	0.143	0.357
Non-cancer illness code self-reported: hyperthyroidism/thyrotoxicosis || id:UKB-a:76	MR Egger	6	-1.184	7.576	0.883
Non-cancer illness code self-reported: hyperthyroidism/thyrotoxicosis || id:UKB-a:76	Weighted median	6	-1.555	3.023	0.607
Non-cancer illness code self-reported: hyperthyroidism/thyrotoxicosis || id:UKB-a:76	Inverse variance weighted	6	-1.274	3.216	0.692
Non-cancer illness code self-reported: hypertrophic cardiomyopathy (hcm / hocm) || id:UKB-a:114	MR Egger	23	67.214	43.195	0.135
Non-cancer illness code self-reported: hypertrophic cardiomyopathy (hcm / hocm) || id:UKB-a:114	Weighted median	23	8.066	21.097	0.702
Non-cancer illness code self-reported: hypertrophic cardiomyopathy (hcm / hocm) || id:UKB-a:114	Inverse variance weighted	23	-3.282	15.587	0.833
Non-cancer illness code self-reported: hypopituitarism || id:UKB-a:98	MR Egger	9	98.510	135.252	0.490
Non-cancer illness code self-reported: hypopituitarism || id:UKB-a:98	Weighted median	9	37.446	30.939	0.226
Non-cancer illness code self-reported: hypopituitarism || id:UKB-a:98	Inverse variance weighted	9	35.883	30.475	0.239
Non-cancer illness code self-reported: hypothyroidism/myxoedema || id:UKB-a:77	MR Egger	80	0.173	0.921	0.852
Non-cancer illness code self-reported: hypothyroidism/myxoedema || id:UKB-a:77	Weighted median	80	0.738	0.565	0.192
Non-cancer illness code self-reported: hypothyroidism/myxoedema || id:UKB-a:77	Inverse variance weighted	80	0.621	0.402	0.123
Non-cancer illness code self-reported: kidney stone/ureter stone/bladder stone || id:UKB-a:72	MR Egger	3	-38.310	275.971	0.912
Non-cancer illness code self-reported: kidney stone/ureter stone/bladder stone || id:UKB-a:72	Weighted median	3	-1.367	7.609	0.857
Non-cancer illness code self-reported: kidney stone/ureter stone/bladder stone || id:UKB-a:72	Inverse variance weighted	3	-4.543	6.242	0.467
Non-cancer illness code self-reported: malabsorption/coeliac disease || id:UKB-a:101	MR Egger	13	-3.330	3.524	0.365
Non-cancer illness code self-reported: malabsorption/coeliac disease || id:UKB-a:101	Weighted median	13	-1.388	2.220	0.532
Non-cancer illness code self-reported: malabsorption/coeliac disease || id:UKB-a:101	Inverse variance weighted	13	-1.125	2.263	0.619
Non-cancer illness code self-reported: migraine || id:UKB-a:78	MR Egger	13	-6.238	6.673	0.370
Non-cancer illness code self-reported: migraine || id:UKB-a:78	Weighted median	13	-1.780	1.815	0.327
Non-cancer illness code self-reported: migraine || id:UKB-a:78	Inverse variance weighted	13	-0.674	1.309	0.607
Non-cancer illness code self-reported: nasal polyps || id:UKB-a:97	MR Egger	5	-48.383	48.313	0.390
Non-cancer illness code self-reported: nasal polyps || id:UKB-a:97	Weighted median	5	6.250	7.910	0.429
Non-cancer illness code self-reported: nasal polyps || id:UKB-a:97	Inverse variance weighted	5	3.079	6.240	0.622
Non-cancer illness code self-reported: osteoarthritis || id:UKB-a:106	Inverse variance weighted	2	0.837	4.279	0.845
Non-cancer illness code self-reported: osteoporosis || id:UKB-a:87	MR Egger	8	-25.032	32.608	0.472
Non-cancer illness code self-reported: osteoporosis || id:UKB-a:87	Weighted median	8	-1.430	3.567	0.689
Non-cancer illness code self-reported: osteoporosis || id:UKB-a:87	Inverse variance weighted	8	-0.029	3.069	0.993
Non-cancer illness code self-reported: pernicious anaemia || id:UKB-a:90	MR Egger	3	33.226	31.630	0.484
Non-cancer illness code self-reported: pernicious anaemia || id:UKB-a:90	Weighted median	3	13.233	13.735	0.335
Non-cancer illness code self-reported: pernicious anaemia || id:UKB-a:90	Inverse variance weighted	3	4.517	11.083	0.684
Non-cancer illness code self-reported: pneumothorax || id:UKB-a:111	MR Egger	5	-49.391	112.746	0.691
Non-cancer illness code self-reported: pneumothorax || id:UKB-a:111	Weighted median	5	15.401	28.914	0.594
Non-cancer illness code self-reported: pneumothorax || id:UKB-a:111	Inverse variance weighted	5	1.969	23.832	0.934
Non-cancer illness code self-reported: polio / poliomyelitis || id:UKB-a:112	MR Egger	5	-203.162	101.834	0.140
Non-cancer illness code self-reported: polio / poliomyelitis || id:UKB-a:112	Weighted median	5	2.985	31.676	0.925
Non-cancer illness code self-reported: polio / poliomyelitis || id:UKB-a:112	Inverse variance weighted	5	18.586	34.998	0.595

Non-cancer illness code self-reported: psoriasis || id:UKB-a:100	MR Egger	15	-0.361	1.254	0.778
Non-cancer illness code self-reported: psoriasis || id:UKB-a:100	Weighted median	15	-0.163	1.184	0.890
Non-cancer illness code self-reported: psoriasis || id:UKB-a:100	Inverse variance weighted	15	-0.475	1.021	0.642
Non-cancer illness code self-reported: pulmonary embolism +/- dvt || id:UKB-a:64	MR Egger	6	-7.757	7.218	0.343
Non-cancer illness code self-reported: pulmonary embolism +/- dvt || id:UKB-a:64	Weighted median	6	5.124	3.813	0.179
Non-cancer illness code self-reported: pulmonary embolism +/- dvt || id:UKB-a:64	Inverse variance weighted	6	1.002	3.671	0.785
Non-cancer illness code self-reported: rheumatoid arthritis || id:UKB-a:105	MR Egger	5	2.686	6.580	0.710
Non-cancer illness code self-reported: rheumatoid arthritis || id:UKB-a:105	Weighted median	5	-1.643	2.540	0.518
Non-cancer illness code self-reported: rheumatoid arthritis || id:UKB-a:105	Inverse variance weighted	5	-1.489	3.609	0.680
Non-cancer illness code self-reported: type 2 diabetes || id:UKB-a:75	MR Egger	3	7.880	16.790	0.721
Non-cancer illness code self-reported: type 2 diabetes || id:UKB-a:75	Weighted median	3	-2.081	5.722	0.716
Non-cancer illness code self-reported: type 2 diabetes || id:UKB-a:75	Inverse variance weighted	3	-1.832	5.477	0.738
Non-cancer illness code self-reported: ulcerative colitis || id:UKB-a:104	MR Egger	7	6.006	17.735	0.749
Non-cancer illness code self-reported: ulcerative colitis || id:UKB-a:104	Weighted median	7	4.655	6.638	0.483
Non-cancer illness code self-reported: ulcerative colitis || id:UKB-a:104	Inverse variance weighted	7	3.441	5.354	0.520
Non-cancer illness code self-reported: uterine fibroids || id:UKB-a:91	MR Egger	5	-29.873	10.490	0.065
Non-cancer illness code self-reported: uterine fibroids || id:UKB-a:91	Weighted median	5	2.268	5.018	0.651
Non-cancer illness code self-reported: uterine fibroids || id:UKB-a:91	Inverse variance weighted	5	0.326	5.358	0.951
Non-cancer illness code self-reported: varicose veins || id:UKB-a:110	Inverse variance weighted	2	-17.707	17.452	0.310
Non-cancer illness code self-reported: vitiligo || id:UKB-a:115	MR Egger	10	11.558	18.359	0.547
Non-cancer illness code self-reported: vitiligo || id:UKB-a:115	Weighted median	10	8.546	13.254	0.519
Non-cancer illness code self-reported: vitiligo || id:UKB-a:115	Inverse variance weighted	10	4.273	11.366	0.707
Number of children fathered || id:UKB-a:304	Inverse variance weighted	2	-0.381	0.535	0.476
Number of cigarettes currently smoked daily (current cigarette smokers) || id:UKB-a:342	MR Egger	3	-0.067	0.703	0.939
Number of cigarettes currently smoked daily (current cigarette smokers) || id:UKB-a:342	Weighted median	3	-0.066	0.147	0.655
Number of cigarettes currently smoked daily (current cigarette smokers) || id:UKB-a:342	Inverse variance weighted	3	-0.051	0.137	0.708
Number of cigarettes previously smoked daily || id:UKB-a:328	MR Egger	3	-0.137	0.409	0.794
Number of cigarettes previously smoked daily || id:UKB-a:328	Weighted median	3	-0.105	0.144	0.466
Number of cigarettes previously smoked daily || id:UKB-a:328	Inverse variance weighted	3	-0.099	0.142	0.487
Number of days/week of moderate physical activity 10+ minutes || id:UKB-a:508	MR Egger	7	-0.130	1.180	0.916
Number of days/week of moderate physical activity 10+ minutes || id:UKB-a:508	Weighted median	7	0.478	0.212	0.024
Number of days/week of moderate physical activity 10+ minutes || id:UKB-a:508	Inverse variance weighted	7	0.429	0.163	0.009
Number of days/week of vigorous physical activity 10+ minutes || id:UKB-a:511	MR Egger	4	2.970	24.377	0.914
Number of days/week of vigorous physical activity 10+ minutes || id:UKB-a:511	Weighted median	4	0.442	0.317	0.163
Number of days/week of vigorous physical activity 10+ minutes || id:UKB-a:511	Inverse variance weighted	4	-0.023	0.595	0.969
Number of days/week walked 10+ minutes || id:UKB-a:506	MR Egger	11	-1.905	0.989	0.086
Number of days/week walked 10+ minutes || id:UKB-a:506	Weighted median	11	0.025	0.222	0.909
Number of days/week walked 10+ minutes || id:UKB-a:506	Inverse variance weighted	11	-0.045	0.185	0.809
Number of incorrect matches in round || id:UKB-a:356	MR Egger	17	1.425	1.167	0.241
Number of incorrect matches in round || id:UKB-a:356	Weighted median	17	0.089	0.317	0.780
Number of incorrect matches in round || id:UKB-a:356	Inverse variance weighted	17	0.245	0.234	0.294
Number of live births || id:UKB-a:317	Inverse variance weighted	2	0.373	0.397	0.347

Number of operations self-reported || id:UKB-a:23	MR Egger	8	0.035	1.985	0.986
Number of operations self-reported || id:UKB-a:23	Weighted median	8	-0.232	0.550	0.673
Number of operations self-reported || id:UKB-a:23	Inverse variance weighted	8	-0.511	0.482	0.290
Number of self-reported cancers || id:UKB-a:21	MR Egger	8	3.112	4.051	0.471
Number of self-reported cancers || id:UKB-a:21	Weighted median	8	1.931	1.406	0.169
Number of self-reported cancers || id:UKB-a:21	Inverse variance weighted	8	0.326	1.135	0.774
Number of self-reported non-cancer illnesses || id:UKB-a:22	MR Egger	20	0.225	1.163	0.849
Number of self-reported non-cancer illnesses || id:UKB-a:22	Weighted median	20	0.018	0.348	0.958
Number of self-reported non-cancer illnesses || id:UKB-a:22	Inverse variance weighted	20	-0.441	0.247	0.075
Number of treatments/medications taken || id:UKB-a:24	MR Egger	22	-0.635	0.946	0.510
Number of treatments/medications taken || id:UKB-a:24	Weighted median	22	-0.142	0.358	0.691
Number of treatments/medications taken || id:UKB-a:24	Inverse variance weighted	22	-0.019	0.263	0.941
Obesity class 1 || id:90	MR Egger	17	-0.207	0.136	0.149
Obesity class 1 || id:90	Weighted median	17	-0.080	0.049	0.103
Obesity class 1 || id:90	Inverse variance weighted	17	0.002	0.050	0.962
Octadecanedioate || id:735	Inverse variance weighted	2	0.120	0.342	0.725
Octanoylcarnitine || id:615	Inverse variance weighted	2	0.404	0.382	0.291
Omega-3 fatty acids || id:855	MR Egger	5	0.029	0.234	0.909
Omega-3 fatty acids || id:855	Weighted median	5	0.080	0.079	0.307
Omega-3 fatty acids || id:855	Inverse variance weighted	5	0.099	0.062	0.108
Omega-6 fatty acids || id:856	MR Egger	11	0.031	0.109	0.780
Omega-6 fatty acids || id:856	Weighted median	11	0.084	0.056	0.135
Omega-6 fatty acids || id:856	Inverse variance weighted	11	0.061	0.044	0.165
Omega-9 and saturated fatty acids || id:857	MR Egger	7	0.557	0.225	0.057
Omega-9 and saturated fatty acids || id:857	Weighted median	7	0.046	0.072	0.521
Omega-9 and saturated fatty acids || id:857	Inverse variance weighted	7	0.051	0.064	0.430
Other serious medical condition/disability diagnosed by doctor || id:UKB-a:309	MR Egger	3	6.114	4.333	0.392
Other serious medical condition/disability diagnosed by doctor || id:UKB-a:309	Weighted median	3	0.784	1.573	0.618
Other serious medical condition/disability diagnosed by doctor || id:UKB-a:309	Inverse variance weighted	3	0.859	1.503	0.568
Ovarian cancer || id:1120	MR Egger	10	0.027	0.102	0.794
Ovarian cancer || id:1120	Weighted median	10	-0.007	0.051	0.896
Ovarian cancer || id:1120	Inverse variance weighted	10	-0.025	0.040	0.528
Overall health rating || id:UKB-a:251	MR Egger	54	-1.248	1.178	0.294
Overall health rating || id:UKB-a:251	Weighted median	54	-0.258	0.267	0.334
Overall health rating || id:UKB-a:251	Inverse variance weighted	54	-0.343	0.205	0.095
Pack years adult smoking as proportion of life span exposed to smoking PREVIEW ONLY || id:UKB-a:238	MR Egger	11	-0.246	0.265	0.378
Pack years adult smoking as proportion of life span exposed to smoking PREVIEW ONLY || id:UKB-a:238	Weighted median	11	-0.188	0.147	0.199
Pack years adult smoking as proportion of life span exposed to smoking PREVIEW ONLY || id:UKB-a:238	Inverse variance weighted	11	-0.157	0.115	0.174
Pack years of smoking PREVIEW ONLY || id:UKB-a:237	MR Egger	10	-0.030	0.295	0.920
Pack years of smoking PREVIEW ONLY || id:UKB-a:237	Weighted median	10	-0.166	0.150	0.266
Pack years of smoking PREVIEW ONLY || id:UKB-a:237	Inverse variance weighted	10	-0.190	0.124	0.124
Packed cell volume || id:274	MR Egger	13	0.035	0.185	0.855

Packed cell volume || id:274	Weighted median	13	-0.022	0.044	0.610
Packed cell volume || id:274	Inverse variance weighted	13	-0.066	0.040	0.098
Pain type(s) experienced in last month: Back pain || id:UKB-a:473	MR Egger	8	4.931	2.786	0.127
Pain type(s) experienced in last month: Back pain || id:UKB-a:473	Weighted median	8	0.517	0.920	0.574
Pain type(s) experienced in last month: Back pain || id:UKB-a:473	Inverse variance weighted	8	0.346	0.741	0.641
Pain type(s) experienced in last month: Headache || id:UKB-a:469	MR Egger	31	-0.726	1.188	0.546
Pain type(s) experienced in last month: Headache || id:UKB-a:469	Weighted median	31	-0.469	0.542	0.387
Pain type(s) experienced in last month: Headache || id:UKB-a:469	Inverse variance weighted	31	-0.458	0.365	0.210
Pain type(s) experienced in last month: Hip pain || id:UKB-a:475	MR Egger	3	1.234	4.123	0.815
Pain type(s) experienced in last month: Hip pain || id:UKB-a:475	Weighted median	3	0.169	2.186	0.938
Pain type(s) experienced in last month: Hip pain || id:UKB-a:475	Inverse variance weighted	3	0.134	1.881	0.943
Pain type(s) experienced in last month: Knee pain || id:UKB-a:476	MR Egger	7	5.912	5.073	0.296
Pain type(s) experienced in last month: Knee pain || id:UKB-a:476	Weighted median	7	-0.551	1.122	0.623
Pain type(s) experienced in last month: Knee pain || id:UKB-a:476	Inverse variance weighted	7	-0.472	0.843	0.576
Pain type(s) experienced in last month: Neck or shoulder pain || id:UKB-a:472	Inverse variance weighted	2	1.284	1.687	0.447
Pain type(s) experienced in last month: None of the above || id:UKB-a:470	MR Egger	15	-6.002	3.255	0.088
Pain type(s) experienced in last month: None of the above || id:UKB-a:470	Weighted median	15	-0.778	0.730	0.287
Pain type(s) experienced in last month: None of the above || id:UKB-a:470	Inverse variance weighted	15	-0.969	0.499	0.052
palmitoleic acid (16:1n7) || id:1156	MR Egger	5	-0.813	1.683	0.662
palmitoleic acid (16:1n7) || id:1156	Weighted median	5	-0.196	0.405	0.628
palmitoleic acid (16:1n7) || id:1156	Inverse variance weighted	5	-0.197	0.344	0.567
Parkinson's disease || id:812	MR Egger	4	-0.053	0.256	0.856
Parkinson's disease || id:812	Weighted median	4	0.006	0.040	0.875
Parkinson's disease || id:812	Inverse variance weighted	4	0.032	0.036	0.376
Past tobacco smoking || id:UKB-a:17	MR Egger	41	0.061	0.673	0.929
Past tobacco smoking || id:UKB-a:17	Weighted median	41	0.063	0.167	0.705
Past tobacco smoking || id:UKB-a:17	Inverse variance weighted	41	-0.071	0.134	0.596
Peak expiratory flow (PEF) || id:UKB-a:338	MR Egger	78	0.070	0.481	0.885
Peak expiratory flow (PEF) || id:UKB-a:338	Weighted median	78	-0.070	0.168	0.676
Peak expiratory flow (PEF) || id:UKB-a:338	Inverse variance weighted	78	0.017	0.129	0.895
Percent emphysema || id:1005	Inverse variance weighted	2	0.020	0.177	0.911
PGC cross-disorder traits || id:803	MR Egger	4	1.545	0.558	0.110
PGC cross-disorder traits || id:803	Weighted median	4	0.017	0.119	0.888
PGC cross-disorder traits || id:803	Inverse variance weighted	4	0.115	0.149	0.441
Phospholipids in IDL || id:871	MR Egger	20	-0.006	0.052	0.904
Phospholipids in IDL || id:871	Weighted median	20	0.051	0.045	0.256
Phospholipids in IDL || id:871	Inverse variance weighted	20	0.058	0.032	0.069
Phospholipids in large HDL || id:879	MR Egger	12	0.073	0.125	0.570
Phospholipids in large HDL || id:879	Weighted median	12	-0.047	0.049	0.339
Phospholipids in large HDL || id:879	Inverse variance weighted	12	-0.049	0.043	0.253
Phospholipids in large LDL || id:885	MR Egger	20	-0.019	0.049	0.710
Phospholipids in large LDL || id:885	Weighted median	20	0.049	0.042	0.240

Phospholipids in large LDL || id:885	Inverse variance weighted	20	0.070	0.034	0.040
Phospholipids in large VLDL || id:891	MR Egger	8	0.212	0.159	0.232
Phospholipids in large VLDL || id:891	Weighted median	8	0.108	0.068	0.113
Phospholipids in large VLDL || id:891	Inverse variance weighted	8	0.100	0.054	0.064
Platelet count || id:1008	MR Egger	32	0.001	0.002	0.744
Platelet count || id:1008	Weighted median	32	0.001	0.001	0.376
Platelet count || id:1008	Inverse variance weighted	32	0.001	0.001	0.493
Potassium in urine || id:UKB-a:334	MR Egger	9	0.652	1.918	0.744
Potassium in urine || id:UKB-a:334	Weighted median	9	-0.028	0.376	0.940
Potassium in urine || id:UKB-a:334	Inverse variance weighted	9	-0.175	0.300	0.559
Primary sclerosing cholangitis || id:1112	MR Egger	17	0.011	0.027	0.694
Primary sclerosing cholangitis || id:1112	Weighted median	17	-0.001	0.017	0.940
Primary sclerosing cholangitis || id:1112	Inverse variance weighted	17	-0.008	0.015	0.609
Proline || id:355	MR Egger	3	-0.237	0.543	0.738
Proline || id:355	Weighted median	3	-0.064	0.400	0.872
Proline || id:355	Inverse variance weighted	3	-0.016	0.380	0.966
Propionylcarnitine || id:479	MR Egger	4	1.048	1.005	0.406
Propionylcarnitine || id:479	Weighted median	4	-0.052	0.488	0.915
Propionylcarnitine || id:479	Inverse variance weighted	4	-0.138	0.401	0.732
Prospective memory result || id:UKB-a:197	Inverse variance weighted	2	2.048	0.738	0.006
Pulse rate || id:UKB-a:363	MR Egger	50	-0.059	0.155	0.703
Pulse rate || id:UKB-a:363	Weighted median	50	-0.024	0.086	0.779
Pulse rate || id:UKB-a:363	Inverse variance weighted	50	-0.053	0.058	0.365
Pulse wave peak to peak time || id:UKB-a:365	Inverse variance weighted	2	-0.144	0.388	0.711
Pulse wave reflection index || id:UKB-a:364	Inverse variance weighted	2	-0.144	0.375	0.702
Putamen volume || id:1047	MR Egger	4	-0.001	0.001	0.523
Putamen volume || id:1047	Weighted median	4	0.000	0.000	0.735
Putamen volume || id:1047	Inverse variance weighted	4	0.000	0.000	0.432
Pyroglutamine* || id:501	MR Egger	4	0.906	0.732	0.342
Pyroglutamine* || id:501	Weighted median	4	0.273	0.253	0.280
Pyroglutamine* || id:501	Inverse variance weighted	4	0.232	0.217	0.284
Ratio of bisallylic groups to double bonds || id:844	MR Egger	5	0.041	0.061	0.550
Ratio of bisallylic groups to double bonds || id:844	Weighted median	5	-0.003	0.036	0.941
Ratio of bisallylic groups to double bonds || id:844	Inverse variance weighted	5	-0.019	0.035	0.579
Ratio of bisallylic groups to total fatty acids || id:845	MR Egger	4	0.041	0.068	0.610
Ratio of bisallylic groups to total fatty acids || id:845	Weighted median	4	-0.002	0.040	0.962
Ratio of bisallylic groups to total fatty acids || id:845	Inverse variance weighted	4	-0.015	0.038	0.689
Reason for reducing amount of alcohol drunk: Health precaution || id:UKB-a:314	Inverse variance weighted	2	0.240	0.594	0.687
Red blood cell count || id:275	MR Egger	26	-0.366	0.558	0.519
Red blood cell count || id:275	Weighted median	26	-0.059	0.245	0.810
Red blood cell count || id:275	Inverse variance weighted	26	-0.438	0.200	0.029
Rheumatoid arthritis || id:831	MR Egger	14	0.050	0.036	0.198

Rheumatoid arthritis || id:831	Weighted median	14	0.027	0.020	0.182
Rheumatoid arthritis || id:831	Inverse variance weighted	14	0.026	0.016	0.104
Rheumatoid arthritis || id:832	MR Egger	34	0.027	0.043	0.531
Rheumatoid arthritis || id:832	Weighted median	34	-0.013	0.029	0.659
Rheumatoid arthritis || id:832	Inverse variance weighted	34	0.014	0.022	0.518
Rheumatoid arthritis || id:833	MR Egger	45	0.043	0.044	0.339
Rheumatoid arthritis || id:833	Weighted median	45	0.007	0.027	0.806
Rheumatoid arthritis || id:833	Inverse variance weighted	45	0.010	0.021	0.651
Rheumatoid arthritis || id:834	MR Egger	8	-0.009	0.028	0.751
Rheumatoid arthritis || id:834	Weighted median	8	-0.009	0.017	0.583
Rheumatoid arthritis || id:834	Inverse variance weighted	8	0.005	0.018	0.780
Risk taking || id:UKB-a:241	MR Egger	10	-5.874	3.209	0.105
Risk taking || id:UKB-a:241	Weighted median	10	-1.239	0.920	0.178
Risk taking || id:UKB-a:241	Inverse variance weighted	10	-0.734	0.686	0.284
Schizophrenia || id:22	MR Egger	69	-0.094	0.112	0.405
Schizophrenia || id:22	Weighted median	69	-0.035	0.037	0.340
Schizophrenia || id:22	Inverse variance weighted	69	-0.011	0.028	0.711
Seen a psychiatrist for nerves anxiety tension or depression || id:UKB-a:247	MR Egger	3	-5.616	8.386	0.624
Seen a psychiatrist for nerves anxiety tension or depression || id:UKB-a:247	Weighted median	3	-2.350	2.351	0.318
Seen a psychiatrist for nerves anxiety tension or depression || id:UKB-a:247	Inverse variance weighted	3	-1.021	1.819	0.575
Seen doctor (GP) for nerves anxiety tension or depression || id:UKB-a:246	MR Egger	19	0.164	2.953	0.956
Seen doctor (GP) for nerves anxiety tension or depression || id:UKB-a:246	Weighted median	19	0.024	0.667	0.971
Seen doctor (GP) for nerves anxiety tension or depression || id:UKB-a:246	Inverse variance weighted	19	0.001	0.558	0.999
Sensitivity / hurt feelings || id:UKB-a:48	MR Egger	23	-1.750	1.587	0.283
Sensitivity / hurt feelings || id:UKB-a:48	Weighted median	23	0.142	0.559	0.800
Sensitivity / hurt feelings || id:UKB-a:48	Inverse variance weighted	23	-0.018	0.385	0.963
Serine || id:464	Inverse variance weighted	2	-0.037	0.503	0.941
Serum creatinine (eGFRcrea) || id:1104	MR Egger	42	-1.002	1.089	0.363
Serum creatinine (eGFRcrea) || id:1104	Weighted median	42	-0.062	0.426	0.884
Serum creatinine (eGFRcrea) || id:1104	Inverse variance weighted	42	0.452	0.307	0.142
Serum creatinine (eGFRcrea) || id:1105	MR Egger	42	-0.411	1.102	0.711
Serum creatinine (eGFRcrea) || id:1105	Weighted median	42	-0.230	0.435	0.596
Serum creatinine (eGFRcrea) || id:1105	Inverse variance weighted	42	0.249	0.314	0.428
Serum cystatin C (eGFRcys) || id:1106	MR Egger	5	-0.318	0.676	0.670
Serum cystatin C (eGFRcys) || id:1106	Weighted median	5	-0.071	0.233	0.759
Serum cystatin C (eGFRcys) || id:1106	Inverse variance weighted	5	0.011	0.427	0.979
Sitting height ratio || id:1070	MR Egger	4	-0.703	1.198	0.617
Sitting height ratio || id:1070	Weighted median	4	0.003	0.146	0.986
Sitting height ratio || id:1070	Inverse variance weighted	4	0.051	0.122	0.675
Sleep duration || id:1088	MR Egger	3	-0.213	2.391	0.943
Sleep duration || id:1088	Weighted median	3	0.083	0.376	0.825
Sleep duration || id:1088	Inverse variance weighted	3	-0.184	0.329	0.575

Sleep duration || id:UKB-a:9	MR Egger	41	-1.430	0.887	0.115
Sleep duration || id:UKB-a:9	Weighted median	41	0.257	0.315	0.413
Sleep duration || id:UKB-a:9	Inverse variance weighted	41	0.347	0.229	0.130
Sleeplessness / insomnia || id:UKB-a:13	MR Egger	28	0.942	0.780	0.238
Sleeplessness / insomnia || id:UKB-a:13	Weighted median	28	0.233	0.336	0.488
Sleeplessness / insomnia || id:UKB-a:13	Inverse variance weighted	28	0.221	0.252	0.380
Smoking status: Current || id:UKB-a:225	MR Egger	15	-3.808	3.631	0.313
Smoking status: Current || id:UKB-a:225	Weighted median	15	-0.726	1.054	0.490
Smoking status: Current || id:UKB-a:225	Inverse variance weighted	15	0.587	0.831	0.480
Smoking status: Previous || id:UKB-a:224	MR Egger	18	2.077	5.045	0.686
Smoking status: Previous || id:UKB-a:224	Weighted median	18	0.008	0.685	0.991
Smoking status: Previous || id:UKB-a:224	Inverse variance weighted	18	-0.083	0.616	0.893
Snoring || id:UKB-a:14	MR Egger	22	-1.621	2.103	0.450
Snoring || id:UKB-a:14	Weighted median	22	-0.125	0.561	0.823
Snoring || id:UKB-a:14	Inverse variance weighted	22	0.306	0.410	0.455
Sodium in urine || id:UKB-a:335	MR Egger	32	0.076	0.692	0.913
Sodium in urine || id:UKB-a:335	Weighted median	32	0.354	0.229	0.122
Sodium in urine || id:UKB-a:335	Inverse variance weighted	32	0.161	0.161	0.316
Started insulin within one year diagnosis of diabetes || id:UKB-a:331	MR Egger	8	0.016	0.461	0.974
Started insulin within one year diagnosis of diabetes || id:UKB-a:331	Weighted median	8	-0.150	0.168	0.370
Started insulin within one year diagnosis of diabetes || id:UKB-a:331	Inverse variance weighted	8	-0.017	0.143	0.908
Stearate (18:0) || id:331	Inverse variance weighted	2	1.081	0.737	0.142
Stearic acid (18:0) plasma levels || id:1158	MR Egger	3	0.806	1.306	0.648
Stearic acid (18:0) plasma levels || id:1158	Weighted median	3	-0.002	0.060	0.967
Stearic acid (18:0) plasma levels || id:1158	Inverse variance weighted	3	-0.006	0.052	0.905
Suffer from 'nerves' || id:UKB-a:200	MR Egger	10	2.401	5.719	0.686
Suffer from 'nerves' || id:UKB-a:200	Weighted median	10	0.097	0.931	0.917
Suffer from 'nerves' || id:UKB-a:200	Inverse variance weighted	10	0.807	0.734	0.271
Systemic lupus erythematosus || id:815	MR Egger	3	-0.090	0.301	0.816
Systemic lupus erythematosus || id:815	Weighted median	3	-0.020	0.033	0.546
Systemic lupus erythematosus || id:815	Inverse variance weighted	3	-0.049	0.038	0.200
Systolic blood pressure automated reading || id:UKB-a:360	MR Egger	162	-0.023	0.231	0.920
Systolic blood pressure automated reading || id:UKB-a:360	Weighted median	162	0.092	0.097	0.341
Systolic blood pressure automated reading || id:UKB-a:360	Inverse variance weighted	162	0.080	0.070	0.251
Taking other prescription medications || id:UKB-a:310	MR Egger	10	2.841	2.697	0.323
Taking other prescription medications || id:UKB-a:310	Weighted median	10	0.683	0.809	0.399
Taking other prescription medications || id:UKB-a:310	Inverse variance weighted	10	0.353	0.719	0.624
Tense / 'highly strung' || id:UKB-a:52	MR Egger	16	-5.785	5.307	0.294
Tense / 'highly strung' || id:UKB-a:52	Weighted median	16	0.940	0.840	0.263
Tense / 'highly strung' || id:UKB-a:52	Inverse variance weighted	16	0.829	0.627	0.186
Tetradecanedioate || id:709	MR Egger	3	-0.435	0.466	0.522
Tetradecanedioate || id:709	Weighted median	3	0.047	0.160	0.770

Tetradecanedioate || id:709	Inverse variance weighted	3	0.146	0.146	0.318
Time from waking to first cigarette || id:UKB-a:343	MR Egger	3	0.457	1.247	0.776
Time from waking to first cigarette || id:UKB-a:343	Weighted median	3	0.065	0.117	0.578
Time from waking to first cigarette || id:UKB-a:343	Inverse variance weighted	3	0.037	0.108	0.734
Time spent driving || id:UKB-a:7	Inverse variance weighted	2	-0.700	0.715	0.327
Time spent using computer || id:UKB-a:6	MR Egger	36	-0.953	1.300	0.468
Time spent using computer || id:UKB-a:6	Weighted median	36	-0.252	0.255	0.324
Time spent using computer || id:UKB-a:6	Inverse variance weighted	36	-0.278	0.226	0.219
Time spent watching television (TV) || id:UKB-a:5	MR Egger	57	-0.985	0.854	0.253
Time spent watching television (TV) || id:UKB-a:5	Weighted median	57	0.328	0.224	0.144
Time spent watching television (TV) || id:UKB-a:5	Inverse variance weighted	57	0.339	0.173	0.050
Tobacco smoking: Ex-smoker || id:UKB-a:260	Inverse variance weighted	2	-0.862	1.016	0.396
Total cholesterol || id:301	MR Egger	85	0.069	0.064	0.285
Total cholesterol || id:301	Weighted median	85	0.071	0.054	0.192
Total cholesterol || id:301	Inverse variance weighted	85	0.093	0.039	0.019
Total cholesterol in HDL || id:864	MR Egger	13	-0.026	0.107	0.810
Total cholesterol in HDL || id:864	Weighted median	13	-0.059	0.056	0.300
Total cholesterol in HDL || id:864	Inverse variance weighted	13	-0.075	0.043	0.080
Total cholesterol in IDL || id:867	MR Egger	19	0.000	0.050	0.994
Total cholesterol in IDL || id:867	Weighted median	19	0.048	0.042	0.250
Total cholesterol in IDL || id:867	Inverse variance weighted	19	0.057	0.031	0.063
Total cholesterol in large HDL || id:874	MR Egger	15	0.080	0.097	0.423
Total cholesterol in large HDL || id:874	Weighted median	15	-0.035	0.052	0.505
Total cholesterol in large HDL || id:874	Inverse variance weighted	15	-0.087	0.043	0.045
Total cholesterol in large LDL || id:880	MR Egger	21	-0.015	0.046	0.743
Total cholesterol in large LDL || id:880	Weighted median	21	0.045	0.041	0.271
Total cholesterol in large LDL || id:880	Inverse variance weighted	21	0.068	0.033	0.040
Total cholesterol in large VLDL || id:886	MR Egger	9	0.106	0.150	0.502
Total cholesterol in large VLDL || id:886	Weighted median	9	0.118	0.070	0.089
Total cholesterol in large VLDL || id:886	Inverse variance weighted	9	0.105	0.052	0.044
Total cholesterol in LDL || id:895	MR Egger	21	-0.018	0.046	0.691
Total cholesterol in LDL || id:895	Weighted median	21	0.047	0.040	0.244
Total cholesterol in LDL || id:895	Inverse variance weighted	21	0.063	0.034	0.063
Total cholesterol in medium HDL || id:898	MR Egger	6	0.027	0.164	0.878
Total cholesterol in medium HDL || id:898	Weighted median	6	-0.095	0.081	0.240
Total cholesterol in medium HDL || id:898	Inverse variance weighted	6	-0.129	0.070	0.064
Total lipids in IDL || id:869	MR Egger	20	-0.009	0.052	0.868
Total lipids in IDL || id:869	Weighted median	20	0.050	0.046	0.272
Total lipids in IDL || id:869	Inverse variance weighted	20	0.064	0.031	0.037
Total lipids in large HDL || id:877	MR Egger	12	0.095	0.119	0.442
Total lipids in large HDL || id:877	Weighted median	12	-0.038	0.049	0.436
Total lipids in large HDL || id:877	Inverse variance weighted	12	-0.045	0.042	0.285

Total lipids in large LDL || id:883	MR Egger	22	-0.007	0.046	0.884
Total lipids in large LDL || id:883	Weighted median	22	0.047	0.042	0.264
Total lipids in large LDL || id:883	Inverse variance weighted	22	0.066	0.031	0.033
Total lipids in large VLDL || id:889	MR Egger	9	0.174	0.163	0.319
Total lipids in large VLDL || id:889	Weighted median	9	0.100	0.071	0.158
Total lipids in large VLDL || id:889	Inverse variance weighted	9	0.074	0.054	0.170
Total lipids in medium HDL || id:901	MR Egger	5	0.215	0.343	0.575
Total lipids in medium HDL || id:901	Weighted median	5	-0.094	0.097	0.332
Total lipids in medium HDL || id:901	Inverse variance weighted	5	-0.106	0.079	0.181
trans-cis-18:2 || id:1148	MR Egger	3	1.248	2.273	0.680
trans-cis-18:2 || id:1148	Weighted median	3	0.504	0.597	0.399
trans-cis-18:2 || id:1148	Inverse variance weighted	3	0.477	0.522	0.361
Transferrin || id:1052	MR Egger	8	-0.003	0.038	0.946
Transferrin || id:1052	Weighted median	8	-0.039	0.031	0.216
Transferrin || id:1052	Inverse variance weighted	8	-0.032	0.027	0.240
Transferrin Saturation || id:1051	MR Egger	3	-0.093	0.070	0.411
Transferrin Saturation || id:1051	Weighted median	3	0.008	0.042	0.857
Transferrin Saturation || id:1051	Inverse variance weighted	3	0.016	0.055	0.774
Treatment/medication code: allopurinol || id:UKB-a:142	MR Egger	15	1.532	2.410	0.536
Treatment/medication code: allopurinol || id:UKB-a:142	Weighted median	15	2.659	1.857	0.152
Treatment/medication code: allopurinol || id:UKB-a:142	Inverse variance weighted	15	0.356	1.433	0.804
Treatment/medication code: amlodipine || id:UKB-a:150	MR Egger	19	-3.828	4.282	0.384
Treatment/medication code: amlodipine || id:UKB-a:150	Weighted median	19	-0.101	1.379	0.942
Treatment/medication code: amlodipine || id:UKB-a:150	Inverse variance weighted	19	0.520	1.039	0.617
Treatment/medication code: arcoxia 90mg tablet || id:UKB-a:185	MR Egger	14	131.276	55.644	0.036
Treatment/medication code: arcoxia 90mg tablet || id:UKB-a:185	Weighted median	14	-10.063	25.280	0.691
Treatment/medication code: arcoxia 90mg tablet || id:UKB-a:185	Inverse variance weighted	14	10.171	22.717	0.654
Treatment/medication code: aspirin || id:UKB-a:132	MR Egger	7	2.389	2.856	0.441
Treatment/medication code: aspirin || id:UKB-a:132	Weighted median	7	2.728	1.223	0.026
Treatment/medication code: aspirin || id:UKB-a:132	Inverse variance weighted	7	2.849	0.963	0.003
Treatment/medication code: atenolol || id:UKB-a:131	MR Egger	6	9.047	10.276	0.428
Treatment/medication code: atenolol || id:UKB-a:131	Weighted median	6	1.530	2.660	0.565
Treatment/medication code: atenolol || id:UKB-a:131	Inverse variance weighted	6	0.598	2.071	0.773
Treatment/medication code: atorvastatin || id:UKB-a:176	MR Egger	18	2.851	2.264	0.226
Treatment/medication code: atorvastatin || id:UKB-a:176	Weighted median	18	3.273	1.344	0.015
Treatment/medication code: atorvastatin || id:UKB-a:176	Inverse variance weighted	18	3.327	0.966	0.001
Treatment/medication code: atrovent 20micrograms inhaler || id:UKB-a:123	MR Egger	3	11.532	67.366	0.892
Treatment/medication code: atrovent 20micrograms inhaler || id:UKB-a:123	Weighted median	3	6.691	20.693	0.746
Treatment/medication code: atrovent 20micrograms inhaler || id:UKB-a:123	Inverse variance weighted	3	6.499	18.274	0.722
Treatment/medication code: becotide 50 inhaler || id:UKB-a:124	MR Egger	3	17.805	31.056	0.669
Treatment/medication code: becotide 50 inhaler || id:UKB-a:124	Weighted median	3	-3.427	8.733	0.695
Treatment/medication code: becotide 50 inhaler || id:UKB-a:124	Inverse variance weighted	3	-3.759	9.218	0.683

Treatment/medication code: bendroflumethiazide || id:UKB-a:193	MR Egger	27	-3.720	3.036	0.232
Treatment/medication code: bendroflumethiazide || id:UKB-a:193	Weighted median	27	-1.308	0.977	0.181
Treatment/medication code: bendroflumethiazide || id:UKB-a:193	Inverse variance weighted	27	-0.480	0.722	0.506
Treatment/medication code: bisoprolol || id:UKB-a:148	Inverse variance weighted	2	8.256	5.464	0.131
Treatment/medication code: carbimazole || id:UKB-a:140	MR Egger	5	-22.929	45.312	0.648
Treatment/medication code: carbimazole || id:UKB-a:140	Weighted median	5	-46.994	18.780	0.012
Treatment/medication code: carbimazole || id:UKB-a:140	Inverse variance weighted	5	-39.164	15.334	0.011
Treatment/medication code: clopidogrel || id:UKB-a:180	Inverse variance weighted	2	14.909	8.148	0.067
Treatment/medication code: doxazosin || id:UKB-a:149	Inverse variance weighted	2	-0.760	6.564	0.908
Treatment/medication code: eumovate cream || id:UKB-a:152	MR Egger	3	-43.276	33.704	0.421
Treatment/medication code: eumovate cream || id:UKB-a:152	Weighted median	3	-37.608	23.488	0.109
Treatment/medication code: eumovate cream || id:UKB-a:152	Inverse variance weighted	3	-37.239	20.036	0.063
Treatment/medication code: ezetimibe || id:UKB-a:191	MR Egger	6	1.443	14.755	0.927
Treatment/medication code: ezetimibe || id:UKB-a:191	Weighted median	6	15.036	5.222	0.004
Treatment/medication code: ezetimibe || id:UKB-a:191	Inverse variance weighted	6	14.766	4.240	0.000
Treatment/medication code: flecainide || id:UKB-a:163	MR Egger	3	-26.947	24.506	0.470
Treatment/medication code: flecainide || id:UKB-a:163	Weighted median	3	6.374	13.351	0.633
Treatment/medication code: flecainide || id:UKB-a:163	Inverse variance weighted	3	13.651	14.931	0.361
Treatment/medication code: gliclazide || id:UKB-a:139	MR Egger	5	-1.316	7.049	0.864
Treatment/medication code: gliclazide || id:UKB-a:139	Weighted median	5	-2.446	3.570	0.493
Treatment/medication code: gliclazide || id:UKB-a:139	Inverse variance weighted	5	-3.459	3.149	0.272
Treatment/medication code: hydroxocobalamin product || id:UKB-a:134	MR Egger	7	22.582	134.086	0.873
Treatment/medication code: hydroxocobalamin product || id:UKB-a:134	Weighted median	7	-7.033	32.689	0.830
Treatment/medication code: hydroxocobalamin product || id:UKB-a:134	Inverse variance weighted	7	-11.260	26.407	0.670
Treatment/medication code: ibuprofen || id:UKB-a:136	Inverse variance weighted	2	-1.250	2.018	0.536
Treatment/medication code: indivina 1mg/2.5mg tablet || id:UKB-a:181	MR Egger	11	-53.365	44.301	0.259
Treatment/medication code: indivina 1mg/2.5mg tablet || id:UKB-a:181	Weighted median	11	45.194	24.921	0.070
Treatment/medication code: indivina 1mg/2.5mg tablet || id:UKB-a:181	Inverse variance weighted	11	50.635	19.511	0.009
Treatment/medication code: insulin product || id:UKB-a:153	MR Egger	7	11.813	6.661	0.136
Treatment/medication code: insulin product || id:UKB-a:153	Weighted median	7	-2.670	2.823	0.344
Treatment/medication code: insulin product || id:UKB-a:153	Inverse variance weighted	7	-1.057	2.567	0.681
Treatment/medication code: isosorbide dinitrate || id:UKB-a:119	MR Egger	11	-14.899	21.922	0.514
Treatment/medication code: isosorbide dinitrate || id:UKB-a:119	Weighted median	11	0.526	12.179	0.966
Treatment/medication code: isosorbide dinitrate || id:UKB-a:119	Inverse variance weighted	11	8.313	12.751	0.514
Treatment/medication code: kapake tablet || id:UKB-a:125	MR Egger	13	-16.597	41.172	0.695
Treatment/medication code: kapake tablet || id:UKB-a:125	Weighted median	13	9.427	25.383	0.710
Treatment/medication code: kapake tablet || id:UKB-a:125	Inverse variance weighted	13	14.125	18.577	0.447
Treatment/medication code: letrozole || id:UKB-a:174	MR Egger	5	-16.844	38.665	0.693
Treatment/medication code: letrozole || id:UKB-a:174	Weighted median	5	-13.056	26.579	0.623
Treatment/medication code: letrozole || id:UKB-a:174	Inverse variance weighted	5	-17.096	21.107	0.418
Treatment/medication code: levothyroxine sodium || id:UKB-a:190	MR Egger	54	0.185	1.386	0.894
Treatment/medication code: levothyroxine sodium || id:UKB-a:190	Weighted median	54	0.943	0.744	0.205

Treatment/medication code: levothyroxine sodium || id:UKB-a:190	Inverse variance weighted	54	0.624	0.553	0.260
Treatment/medication code: liothyronine || id:UKB-a:157	MR Egger	13	2.005	41.703	0.963
Treatment/medication code: liothyronine || id:UKB-a:157	Weighted median	13	-4.715	26.535	0.859
Treatment/medication code: liothyronine || id:UKB-a:157	Inverse variance weighted	13	1.838	19.695	0.926
Treatment/medication code: lisinopril || id:UKB-a:116	Inverse variance weighted	2	6.638	6.428	0.302
Treatment/medication code: lisinopril+hydrochlorothiazide 10mg/12.5mg tablet || id:UKB-a:127	MR Egger	7	112.018	79.705	0.219
Treatment/medication code: lisinopril+hydrochlorothiazide 10mg/12.5mg tablet || id:UKB-a:127	Weighted median	7	15.919	35.945	0.658
Treatment/medication code: lisinopril+hydrochlorothiazide 10mg/12.5mg tablet || id:UKB-a:127	Inverse variance weighted	7	7.823	27.298	0.774
Treatment/medication code: metformin || id:UKB-a:159	MR Egger	25	-0.581	2.394	0.810
Treatment/medication code: metformin || id:UKB-a:159	Weighted median	25	-0.880	1.412	0.533
Treatment/medication code: metformin || id:UKB-a:159	Inverse variance weighted	25	-1.440	1.004	0.151
Treatment/medication code: morphine || id:UKB-a:137	MR Egger	4	-31.161	48.512	0.586
Treatment/medication code: morphine || id:UKB-a:137	Weighted median	4	-25.563	21.045	0.225
Treatment/medication code: morphine || id:UKB-a:137	Inverse variance weighted	4	-16.189	19.383	0.404
Treatment/medication code: nicorandil || id:UKB-a:165	Inverse variance weighted	2	18.643	13.858	0.179
Treatment/medication code: paracetamol || id:UKB-a:194	MR Egger	9	-10.087	5.383	0.103
Treatment/medication code: paracetamol || id:UKB-a:194	Weighted median	9	0.222	1.139	0.846
Treatment/medication code: paracetamol || id:UKB-a:194	Inverse variance weighted	9	0.316	0.996	0.751
Treatment/medication code: prednisolone || id:UKB-a:141	Inverse variance weighted	2	-15.254	8.932	0.088
Treatment/medication code: quinine || id:UKB-a:138	Inverse variance weighted	2	-4.253	7.225	0.556
Treatment/medication code: ramipril || id:UKB-a:117	MR Egger	5	-0.185	8.883	0.985
Treatment/medication code: ramipril || id:UKB-a:117	Weighted median	5	0.969	2.747	0.724
Treatment/medication code: ramipril || id:UKB-a:117	Inverse variance weighted	5	0.101	2.230	0.964
Treatment/medication code: rhinocort 50micrograms nasal spray || id:UKB-a:144	MR Egger	6	-34.097	44.870	0.490
Treatment/medication code: rhinocort 50micrograms nasal spray || id:UKB-a:144	Weighted median	6	-29.248	27.986	0.296
Treatment/medication code: rhinocort 50micrograms nasal spray || id:UKB-a:144	Inverse variance weighted	6	-31.213	22.254	0.161
Treatment/medication code: rosiglitazone || id:UKB-a:184	MR Egger	3	2.441	75.247	0.979
Treatment/medication code: rosiglitazone || id:UKB-a:184	Weighted median	3	36.121	23.000	0.116
Treatment/medication code: rosiglitazone || id:UKB-a:184	Inverse variance weighted	3	32.194	19.556	0.100
Treatment/medication code: salbutamol || id:UKB-a:151	MR Egger	3	12.880	15.962	0.568
Treatment/medication code: salbutamol || id:UKB-a:151	Weighted median	3	-3.580	6.664	0.591
Treatment/medication code: salbutamol || id:UKB-a:151	Inverse variance weighted	3	-1.702	5.440	0.754
Treatment/medication code: seretide 50 evohaler || id:UKB-a:183	MR Egger	6	3.926	35.169	0.916
Treatment/medication code: seretide 50 evohaler || id:UKB-a:183	Weighted median	6	1.687	5.147	0.743
Treatment/medication code: seretide 50 evohaler || id:UKB-a:183	Inverse variance weighted	6	2.246	3.902	0.565
Treatment/medication code: simvastatin || id:UKB-a:121	MR Egger	29	0.777	0.895	0.393
Treatment/medication code: simvastatin || id:UKB-a:121	Weighted median	29	1.087	0.601	0.071
Treatment/medication code: simvastatin || id:UKB-a:121	Inverse variance weighted	29	1.107	0.414	0.007
Treatment/medication code: testogel 50mg gel 5g sachet || id:UKB-a:192	MR Egger	15	-2.811	29.626	0.926
Treatment/medication code: testogel 50mg gel 5g sachet || id:UKB-a:192	Weighted median	15	26.880	24.662	0.276
Treatment/medication code: testogel 50mg gel 5g sachet || id:UKB-a:192	Inverse variance weighted	15	21.694	17.808	0.223
Treatment/medication code: thyroxine product || id:UKB-a:158	MR Egger	10	4.837	9.322	0.618

Treatment/medication code: thyroxine product || id:UKB-a:158	Weighted median	10	1.822	2.952	0.537
Treatment/medication code: thyroxine product || id:UKB-a:158	Inverse variance weighted	10	-0.415	2.883	0.886
Treatment/medication code: tranexamic acid || id:UKB-a:120	Inverse variance weighted	2	27.034	22.812	0.236
Treatment/medication code: venlafaxine || id:UKB-a:167	Inverse variance weighted	2	-3.824	11.180	0.732
Treatment/medication code: ventolin 100micrograms inhaler || id:UKB-a:122	MR Egger	14	3.667	6.589	0.588
Treatment/medication code: ventolin 100micrograms inhaler || id:UKB-a:122	Weighted median	14	1.868	1.808	0.302
Treatment/medication code: ventolin 100micrograms inhaler || id:UKB-a:122	Inverse variance weighted	14	0.651	1.473	0.658
Treatment/medication code: warfarin || id:UKB-a:160	MR Egger	7	-8.112	8.403	0.379
Treatment/medication code: warfarin || id:UKB-a:160	Weighted median	7	2.637	3.717	0.478
Treatment/medication code: warfarin || id:UKB-a:160	Inverse variance weighted	7	1.119	4.468	0.802
Triglycerides || id:302	MR Egger	54	0.173	0.074	0.023
Triglycerides || id:302	Weighted median	54	0.069	0.067	0.302
Triglycerides || id:302	Inverse variance weighted	54	0.048	0.046	0.303
Triglycerides in IDL || id:872	MR Egger	17	-0.052	0.098	0.603
Triglycerides in IDL || id:872	Weighted median	17	0.040	0.053	0.446
Triglycerides in IDL || id:872	Inverse variance weighted	17	0.059	0.038	0.115
Triglycerides in large VLDL || id:892	MR Egger	9	0.106	0.157	0.520
Triglycerides in large VLDL || id:892	Weighted median	9	0.100	0.071	0.159
Triglycerides in large VLDL || id:892	Inverse variance weighted	9	0.069	0.054	0.204
Trunk fat percentage || id:UKB-a:290	MR Egger	233	-0.127	0.252	0.615
Trunk fat percentage || id:UKB-a:290	Weighted median	233	0.035	0.091	0.697
Trunk fat percentage || id:UKB-a:290	Inverse variance weighted	233	-0.023	0.071	0.751
Tryptophan || id:304	MR Egger	18	1.175	12.342	0.925
Tryptophan || id:304	Weighted median	18	1.516	0.911	0.096
Tryptophan || id:304	Inverse variance weighted	18	1.151	0.887	0.194
Type 2 diabetes || id:1090	Inverse variance weighted	2	0.072	0.080	0.369
Type 2 diabetes || id:23	MR Egger	25	-0.086	0.121	0.485
Type 2 diabetes || id:23	Weighted median	25	-0.032	0.043	0.459
Type 2 diabetes || id:23	Inverse variance weighted	25	-0.017	0.031	0.592
Type 2 diabetes || id:24	MR Egger	36	-0.051	0.057	0.379
Type 2 diabetes || id:24	Weighted median	36	-0.026	0.035	0.461
Type 2 diabetes || id:24	Inverse variance weighted	36	-0.038	0.024	0.104
Type 2 diabetes || id:25	MR Egger	25	-0.003	0.065	0.961
Type 2 diabetes || id:25	Weighted median	25	-0.025	0.036	0.496
Type 2 diabetes || id:25	Inverse variance weighted	25	-0.026	0.026	0.308
Type 2 diabetes || id:26	MR Egger	10	0.021	0.193	0.917
Type 2 diabetes || id:26	Weighted median	10	-0.039	0.047	0.402
Type 2 diabetes || id:26	Inverse variance weighted	10	-0.037	0.035	0.284
Types of physical activity in last 4 weeks: Heavy DIY (eg: weeding lawn mowing carpentry digging) || id:UKB-a:487	MR Egger	3	9.170	16.922	0.684
Types of physical activity in last 4 weeks: Heavy DIY (eg: weeding lawn mowing carpentry digging) || id:UKB-a:487	Weighted median	3	-0.533	1.255	0.671
Types of physical activity in last 4 weeks: Heavy DIY (eg: weeding lawn mowing carpentry digging) || id:UKB-a:487	Inverse variance weighted	3	-0.305	1.080	0.778
Types of physical activity in last 4 weeks: Light DIY (eg: pruning watering the lawn) || id:UKB-a:486	Inverse variance weighted	2	-0.757	1.059	0.475

Types of physical activity in last 4 weeks: None of the above || id:UKB-a:483	MR Egger	7	-3.075	9.205	0.752
Types of physical activity in last 4 weeks: None of the above || id:UKB-a:483	Weighted median	7	-0.740	2.254	0.743
Types of physical activity in last 4 weeks: None of the above || id:UKB-a:483	Inverse variance weighted	7	-0.806	1.843	0.662
Types of physical activity in last 4 weeks: Other exercises (eg: swimming cycling keep fit bowling) || id:UKB-a:484	MR Egger	7	5.388	6.035	0.413
Types of physical activity in last 4 weeks: Other exercises (eg: swimming cycling keep fit bowling) || id:UKB-a:484	Weighted median	7	0.031	0.917	0.973
Types of physical activity in last 4 weeks: Other exercises (eg: swimming cycling keep fit bowling) || id:UKB-a:484	Inverse variance weighted	7	0.026	0.699	0.971
Types of physical activity in last 4 weeks: Strenuous sports || id:UKB-a:485	MR Egger	3	-21.880	8.544	0.237
Types of physical activity in last 4 weeks: Strenuous sports || id:UKB-a:485	Weighted median	3	0.177	2.784	0.949
Types of physical activity in last 4 weeks: Strenuous sports || id:UKB-a:485	Inverse variance weighted	3	-1.267	3.840	0.741
Types of physical activity in last 4 weeks: Walking for pleasure (not as a means of transport) || id:UKB-a:482	MR Egger	6	-38.123	27.527	0.238
Types of physical activity in last 4 weeks: Walking for pleasure (not as a means of transport) || id:UKB-a:482	Weighted median	6	-1.095	1.192	0.358
Types of physical activity in last 4 weeks: Walking for pleasure (not as a means of transport) || id:UKB-a:482	Inverse variance weighted	6	-1.087	0.930	0.242
Types of transport used (excluding work): Cycle || id:UKB-a:481	MR Egger	3	47.812	94.222	0.701
Types of transport used (excluding work): Cycle || id:UKB-a:481	Weighted median	3	-0.881	2.898	0.761
Types of transport used (excluding work): Cycle || id:UKB-a:481	Inverse variance weighted	3	-0.216	2.416	0.929
Types of transport used (excluding work): Walk || id:UKB-a:479	Inverse variance weighted	2	-0.930	1.974	0.638
Tyrosine || id:325	MR Egger	3	-6.044	8.278	0.598
Tyrosine || id:325	Weighted median	3	1.327	0.932	0.154
Tyrosine || id:325	Inverse variance weighted	3	1.269	0.817	0.120
Ulcerative colitis || id:32	MR Egger	35	0.006	0.047	0.903
Ulcerative colitis || id:32	Weighted median	35	-0.010	0.023	0.651
Ulcerative colitis || id:32	Inverse variance weighted	35	0.001	0.016	0.951
Underlying (primary) cause of death: ICD10: J84.1 Other interstitial pulmonary diseases with fibrosis || id:UKB-a:358	MR Egger	7	1.523	0.960	0.173
Underlying (primary) cause of death: ICD10: J84.1 Other interstitial pulmonary diseases with fibrosis || id:UKB-a:358	Weighted median	7	0.905	0.527	0.086
Underlying (primary) cause of death: ICD10: J84.1 Other interstitial pulmonary diseases with fibrosis || id:UKB-a:358	Inverse variance weighted	7	0.241	0.478	0.614
Urate || id:1055	MR Egger	24	0.010	0.066	0.879
Urate || id:1055	Weighted median	24	-0.020	0.045	0.655
Urate || id:1055	Inverse variance weighted	24	-0.030	0.040	0.459
Uridine || id:316	MR Egger	3	2.383	3.805	0.644
Uridine || id:316	Weighted median	3	0.101	0.938	0.914
Uridine || id:316	Inverse variance weighted	3	-0.456	0.778	0.558
Usual walking pace || id:UKB-a:513	MR Egger	29	-2.541	1.494	0.101
Usual walking pace || id:UKB-a:513	Weighted median	29	-0.303	0.449	0.500
Usual walking pace || id:UKB-a:513	Inverse variance weighted	29	-0.106	0.340	0.756
Waist circumference || id:102	Inverse variance weighted	2	-0.340	0.145	0.019
Waist circumference || id:103	Inverse variance weighted	2	-0.263	0.173	0.128
Waist circumference || id:105	MR Egger	5	-0.423	1.002	0.701
Waist circumference || id:105	Weighted median	5	-0.367	0.270	0.175
Waist circumference || id:105	Inverse variance weighted	5	-0.407	0.215	0.058
Waist circumference || id:60	MR Egger	46	-0.382	0.230	0.104
Waist circumference || id:60	Weighted median	46	-0.093	0.124	0.455
Waist circumference || id:60	Inverse variance weighted	46	-0.002	0.089	0.982

Waist circumference || id:61	MR Egger	40	-0.086	0.354	0.809
Waist circumference || id:61	Weighted median	40	-0.004	0.135	0.979
Waist circumference || id:61	Inverse variance weighted	40	0.052	0.099	0.602
Waist circumference || id:62	MR Egger	18	-0.246	0.478	0.614
Waist circumference || id:62	Weighted median	18	-0.133	0.156	0.391
Waist circumference || id:62	Inverse variance weighted	18	-0.081	0.132	0.538
Waist circumference || id:63	MR Egger	17	-0.275	0.460	0.558
Waist circumference || id:63	Weighted median	17	-0.048	0.154	0.756
Waist circumference || id:63	Inverse variance weighted	17	-0.046	0.128	0.720
Waist circumference || id:64	MR Egger	15	-0.785	0.348	0.042
Waist circumference || id:64	Weighted median	15	-0.053	0.139	0.701
Waist circumference || id:64	Inverse variance weighted	15	-0.005	0.117	0.969
Waist circumference || id:65	MR Egger	14	-0.903	0.341	0.021
Waist circumference || id:65	Weighted median	14	-0.124	0.135	0.357
Waist circumference || id:65	Inverse variance weighted	14	0.003	0.125	0.983
Waist circumference || id:66	MR Egger	63	-0.180	0.337	0.594
Waist circumference || id:66	Weighted median	63	-0.125	0.113	0.272
Waist circumference || id:66	Inverse variance weighted	63	-0.098	0.080	0.221
Waist circumference || id:67	MR Egger	62	-0.317	0.349	0.368
Waist circumference || id:67	Weighted median	62	-0.081	0.115	0.479
Waist circumference || id:67	Inverse variance weighted	62	-0.106	0.081	0.190
Waist circumference || id:68	MR Egger	26	-0.075	0.349	0.831
Waist circumference || id:68	Weighted median	26	-0.157	0.131	0.231
Waist circumference || id:68	Inverse variance weighted	26	-0.120	0.088	0.171
Waist circumference || id:69	MR Egger	22	-0.192	0.407	0.643
Waist circumference || id:69	Weighted median	22	-0.129	0.134	0.338
Waist circumference || id:69	Inverse variance weighted	22	-0.096	0.095	0.311
Waist circumference || id:70	MR Egger	23	0.166	0.576	0.776
Waist circumference || id:70	Weighted median	23	-0.100	0.135	0.459
Waist circumference || id:70	Inverse variance weighted	23	-0.096	0.101	0.345
Waist circumference || id:71	MR Egger	27	0.134	0.523	0.801
Waist circumference || id:71	Weighted median	27	-0.094	0.124	0.447
Waist circumference || id:71	Inverse variance weighted	27	-0.102	0.096	0.289
Waist-to-hip ratio || id:109	MR Egger	5	2.582	2.506	0.379
Waist-to-hip ratio || id:109	Weighted median	5	-0.404	0.179	0.024
Waist-to-hip ratio || id:109	Inverse variance weighted	5	-0.278	0.156	0.075
Waist-to-hip ratio || id:111	MR Egger	9	0.186	0.563	0.751
Waist-to-hip ratio || id:111	Weighted median	9	-0.048	0.133	0.719
Waist-to-hip ratio || id:111	Inverse variance weighted	9	-0.064	0.098	0.511
Waist-to-hip ratio || id:72	MR Egger	30	-0.710	0.524	0.187
Waist-to-hip ratio || id:72	Weighted median	30	-0.043	0.164	0.794
Waist-to-hip ratio || id:72	Inverse variance weighted	30	-0.078	0.118	0.509

Waist-to-hip ratio || id:73	MR Egger	28	-0.797	0.522	0.139
Waist-to-hip ratio || id:73	Weighted median	28	-0.047	0.164	0.775
Waist-to-hip ratio || id:73	Inverse variance weighted	28	-0.092	0.114	0.419
Waist-to-hip ratio || id:80	MR Egger	33	-0.101	0.251	0.691
Waist-to-hip ratio || id:80	Weighted median	33	-0.062	0.096	0.518
Waist-to-hip ratio || id:80	Inverse variance weighted	33	-0.089	0.068	0.192
Waist-to-hip ratio || id:81	MR Egger	33	0.050	0.265	0.853
Waist-to-hip ratio || id:81	Weighted median	33	-0.010	0.094	0.916
Waist-to-hip ratio || id:81	Inverse variance weighted	33	-0.048	0.069	0.491
Waist-to-hip ratio || id:82	MR Egger	6	0.222	2.565	0.935
Waist-to-hip ratio || id:82	Weighted median	6	-0.153	0.254	0.546
Waist-to-hip ratio || id:82	Inverse variance weighted	6	0.053	0.209	0.801
Waist-to-hip ratio || id:83	MR Egger	5	-4.429	4.598	0.407
Waist-to-hip ratio || id:83	Weighted median	5	-0.053	0.258	0.837
Waist-to-hip ratio || id:83	Inverse variance weighted	5	0.140	0.205	0.495
Weight || id:106	MR Egger	8	-0.619	0.550	0.303
Weight || id:106	Weighted median	8	-0.193	0.132	0.145
Weight || id:106	Inverse variance weighted	8	-0.161	0.106	0.128
Weight || id:107	MR Egger	11	-0.689	0.359	0.087
Weight || id:107	Weighted median	11	-0.310	0.126	0.014
Weight || id:107	Inverse variance weighted	11	-0.211	0.094	0.025
Worrier / anxious feelings || id:UKB-a:51	MR Egger	33	2.135	2.103	0.318
Worrier / anxious feelings || id:UKB-a:51	Weighted median	33	0.518	0.449	0.248
Worrier / anxious feelings || id:UKB-a:51	Inverse variance weighted	33	0.228	0.316	0.472
Worry too long after embarrassment || id:UKB-a:53	MR Egger	14	-4.679	3.074	0.154
Worry too long after embarrassment || id:UKB-a:53	Weighted median	14	-0.643	0.663	0.332
Worry too long after embarrassment || id:UKB-a:53	Inverse variance weighted	14	-0.276	0.560	0.623
X-02269 || id:485	MR Egger	3	0.721	1.373	0.692
X-02269 || id:485	Weighted median	3	0.024	0.210	0.910
X-02269 || id:485	Inverse variance weighted	3	0.075	0.182	0.679
X-03056--N-[3-(2-Oxopyrrolidin-1-yl)propyl]acetamide || id:505	MR Egger	4	-2.000	1.039	0.194
X-03056--N-[3-(2-Oxopyrrolidin-1-yl)propyl]acetamide || id:505	Weighted median	4	-0.316	0.325	0.330
X-03056--N-[3-(2-Oxopyrrolidin-1-yl)propyl]acetamide || id:505	Inverse variance weighted	4	-0.502	0.258	0.052
X-03094 || id:373	MR Egger	5	0.357	2.272	0.885
X-03094 || id:373	Weighted median	5	0.582	0.581	0.316
X-03094 || id:373	Inverse variance weighted	5	0.300	0.504	0.552
X-08402 || id:426	Inverse variance weighted	2	-0.049	0.313	0.874
X-08988 || id:435	Inverse variance weighted	2	0.153	0.440	0.728
X-10510 || id:444	MR Egger	3	0.658	0.831	0.574
X-10510 || id:444	Weighted median	3	0.063	0.408	0.877
X-10510 || id:444	Inverse variance weighted	3	-0.033	0.374	0.929
X-11204 || id:484	Inverse variance weighted	2	-0.135	1.131	0.905

X-11261 || id:490	Inverse variance weighted	2	-0.114	0.227	0.617
X-11315 || id:495	MR Egger	3	-0.598	1.198	0.705
X-11315 || id:495	Weighted median	3	-0.987	0.428	0.021
X-11315 || id:495	Inverse variance weighted	3	-0.951	0.380	0.012
X-11317 || id:496	Inverse variance weighted	2	-0.253	0.799	0.751
X-11327 || id:498	Inverse variance weighted	2	-0.364	1.012	0.719
X-11423--O-sulfo-L-tyrosine || id:509	Inverse variance weighted	2	-1.634	0.696	0.019
X-11440 || id:513	MR Egger	3	0.302	0.171	0.328
X-11440 || id:513	Weighted median	3	0.075	0.107	0.483
X-11440 || id:513	Inverse variance weighted	3	0.066	0.141	0.638
X-11445--5-alpha-pregnan-3beta 20alpha-disulfate || id:517	MR Egger	3	-0.786	1.722	0.727
X-11445--5-alpha-pregnan-3beta 20alpha-disulfate || id:517	Weighted median	3	0.005	0.177	0.976
X-11445--5-alpha-pregnan-3beta 20alpha-disulfate || id:517	Inverse variance weighted	3	0.083	0.151	0.582
X-11469 || id:519	MR Egger	3	1.560	2.270	0.617
X-11469 || id:519	Weighted median	3	0.084	0.219	0.703
X-11469 || id:519	Inverse variance weighted	3	0.115	0.190	0.546
X-11491 || id:524	MR Egger	3	-0.278	0.531	0.693
X-11491 || id:524	Weighted median	3	0.021	0.189	0.913
X-11491 || id:524	Inverse variance weighted	3	0.081	0.172	0.637
X-11529 || id:528	Inverse variance weighted	2	0.013	0.061	0.832
X-11538 || id:531	MR Egger	3	-0.103	0.274	0.771
X-11538 || id:531	Weighted median	3	0.092	0.145	0.524
X-11538 || id:531	Inverse variance weighted	3	0.103	0.138	0.457
X-11593--O-methylascorbate* || id:536	MR Egger	6	0.870	0.629	0.239
X-11593--O-methylascorbate* || id:536	Weighted median	6	0.395	0.275	0.150
X-11593--O-methylascorbate* || id:536	Inverse variance weighted	6	0.205	0.416	0.622
X-11787 || id:541	Inverse variance weighted	2	-0.644	0.334	0.054
X-11792 || id:542	MR Egger	3	-0.457	0.383	0.443
X-11792 || id:542	Weighted median	3	0.062	0.112	0.579
X-11792 || id:542	Inverse variance weighted	3	0.081	0.096	0.398
X-11793--oxidized bilirubin* || id:543	Inverse variance weighted	2	0.041	0.148	0.784
X-11905 || id:560	Inverse variance weighted	2	0.110	0.278	0.693
X-12040 || id:568	Inverse variance weighted	2	0.024	0.055	0.668
X-12063 || id:570	MR Egger	6	-0.158	0.137	0.312
X-12063 || id:570	Weighted median	6	-0.071	0.098	0.469
X-12063 || id:570	Inverse variance weighted	6	-0.044	0.088	0.614
X-12092 || id:580	MR Egger	5	0.069	0.072	0.407
X-12092 || id:580	Weighted median	5	0.082	0.058	0.158
X-12092 || id:580	Inverse variance weighted	5	0.082	0.054	0.128
X-12100--hydroxytryptophan* || id:584	Inverse variance weighted	2	0.955	0.749	0.202
X-12188 || id:587	Inverse variance weighted	2	0.060	0.068	0.375
X-12189 || id:588	Inverse variance weighted	2	0.117	0.087	0.178

X-12244--N-acetylcarnosine || id:596	MR Egger	3	-4.411	4.050	0.473
X-12244--N-acetylcarnosine || id:596	Weighted median	3	0.473	0.477	0.322
X-12244--N-acetylcarnosine || id:596	Inverse variance weighted	3	0.285	0.376	0.449
X-12456 || id:613	Inverse variance weighted	2	0.089	0.206	0.668
X-12510--2-aminooctanoic acid || id:629	MR Egger	3	-0.244	0.221	0.468
X-12510--2-aminooctanoic acid || id:629	Weighted median	3	-0.218	0.137	0.110
X-12510--2-aminooctanoic acid || id:629	Inverse variance weighted	3	-0.225	0.132	0.089
X-12524 || id:630	Inverse variance weighted	2	-0.731	1.221	0.549
X-12556 || id:633	Inverse variance weighted	2	0.270	1.142	0.813
X-12644 || id:636	MR Egger	3	2.448	3.488	0.610
X-12644 || id:636	Weighted median	3	0.038	0.608	0.951
X-12644 || id:636	Inverse variance weighted	3	-0.034	0.489	0.945
X-12696 || id:640	Inverse variance weighted	2	-0.808	0.520	0.120
X-12712 || id:642	Inverse variance weighted	2	0.006	0.081	0.938
X-12728 || id:646	MR Egger	8	-0.064	0.063	0.352
X-12728 || id:646	Weighted median	8	0.006	0.017	0.705
X-12728 || id:646	Inverse variance weighted	8	-0.003	0.014	0.800
X-12798 || id:661	MR Egger	3	-0.200	0.161	0.432
X-12798 || id:661	Weighted median	3	-0.146	0.113	0.197
X-12798 || id:661	Inverse variance weighted	3	-0.146	0.115	0.204
X-12844 || id:665	MR Egger	3	4.829	2.700	0.325
X-12844 || id:665	Weighted median	3	-0.198	0.480	0.681
X-12844 || id:665	Inverse variance weighted	3	-0.162	0.532	0.761
X-12850 || id:667	MR Egger	3	-0.053	0.393	0.914
X-12850 || id:667	Weighted median	3	0.185	0.176	0.293
X-12850 || id:667	Inverse variance weighted	3	0.164	0.163	0.312
X-12855 || id:670	Inverse variance weighted	2	-0.138	0.410	0.736
X-13215 || id:675	Inverse variance weighted	2	-0.266	0.814	0.744
X-13429 || id:683	MR Egger	3	-0.244	0.307	0.573
X-13429 || id:683	Weighted median	3	0.092	0.111	0.409
X-13429 || id:683	Inverse variance weighted	3	0.103	0.111	0.351
X-13431--nonanoylcarnitine* || id:684	Inverse variance weighted	2	0.126	0.126	0.315
X-13435 || id:685	Inverse variance weighted	2	0.051	0.350	0.883
X-13496 || id:691	Inverse variance weighted	2	-1.373	1.645	0.404
X-13548 || id:693	Inverse variance weighted	2	0.004	0.981	0.997
X-14189--leucylalanine || id:719	Inverse variance weighted	2	-0.114	0.400	0.776
X-14205--alpha-glutamyltyrosine || id:720	Inverse variance weighted	2	0.295	0.247	0.232
X-14208--phenylalanylserine || id:721	Inverse variance weighted	2	0.155	0.188	0.408
X-14304--leucylalanine || id:722	Inverse variance weighted	2	-0.132	0.432	0.760
X-14626 || id:729	Inverse variance weighted	2	0.159	0.317	0.616
X-18601 || id:396	MR Egger	3	-3.494	1.596	0.273
X-18601 || id:396	Weighted median	3	-0.010	0.357	0.978

X-18601 || id:396	Inverse variance weighted	3	-0.109	0.395	0.783
Years of schooling || id:1001	MR Egger	59	-1.337	0.745	0.078
Years of schooling || id:1001	Weighted median	59	-0.413	0.173	0.017
Years of schooling || id:1001	Inverse variance weighted	59	-0.247	0.144	0.086
Years of schooling || id:1010	MR Egger	13	-0.985	1.726	0.580
Years of schooling || id:1010	Weighted median	13	-0.581	0.226	0.010
Years of schooling || id:1010	Inverse variance weighted	13	-0.298	0.221	0.177
Years of schooling || id:1011	MR Egger	19	-0.830	0.999	0.418
Years of schooling || id:1011	Weighted median	19	-0.356	0.208	0.088
Years of schooling || id:1011	Inverse variance weighted	19	-0.404	0.153	0.008
Years of schooling || id:837	MR Egger	4	-4.554	4.817	0.444
Years of schooling || id:837	Weighted median	4	0.226	0.383	0.555
Years of schooling || id:837	Inverse variance weighted	4	0.334	0.331	0.313
Zinc || id:1079	Inverse variance weighted	2	0.042	0.046	0.361

id: specific code attributed to each trait on MR Base, se: standard error, n0 of SNPs: number of SNPs

Table S3. Horizontal pleiotropy analysis performed on 635 GWASes across 570 phenotypic traits

Trait	egger intercept	se	p-value
1 5-anhydroglucitol (1 5-AG) || id:419	0.030	0.028	0.484
1-arachidonoylglycerophosphocholine* || id:558	-0.005	0.017	0.801
1-arachidonoylglycerophosphoethanolamine* || id:682	-0.025	0.033	0.583
1-arachidonoylglycerophosphoinositol* || id:634	0.031	0.032	0.434
1-eicosatrienoylglycerophosphocholine* || id:602	NA	NA	NA
1-linoleoylglycerophosphoethanolamine* || id:497	NA	NA	NA
1-palmitoylglycerophosphoethanolamine || id:707	NA	NA	NA
10-undecenoate (11:1n1) || id:482	NA	NA	NA
18,02 linoleic acid (LA) || id:893	-0.007	0.012	0.554
2-aminobutyrate || id:471	NA	NA	NA
2-hydroxyisobutyrate || id:427	0.017	0.057	0.820
22,06 docosahexaenoic acid || id:852	0.014	0.045	0.773
3-dehydrocarnitine* || id:500	0.070	0.103	0.621
3-methyl-2-oxovalerate || id:383	0.023	0.055	0.752
4-acetamidobutanoate || id:339	NA	NA	NA
4-methyl-2-oxopentanoate || id:429	NA	NA	NA
Acetoacetate || id:838	NA	NA	NA
Acetylcarnitine || id:463	NA	NA	NA
Acetylphosphate || id:378	NA	NA	NA
Adiponectin || id:1	-0.002	0.007	0.798
ADpSGEGDFXAEGGGVR* || id:601	NA	NA	NA
ADSGEGDFXAEGGGVR* || id:539	NA	NA	NA
Age at first live birth || id:UKB-a:319	0.005	0.023	0.835
Age at last live birth || id:UKB-a:320	0.074	0.093	0.512
Age at menarche || id:1095	-0.003	0.008	0.733
Age at menopause || id:1004	0.000	0.007	0.950
Age completed full time education || id:UKB-a:505	0.005	0.019	0.784
Age started oral contraceptive pill || id:UKB-a:323	NA	NA	NA
Age when periods started (menarche) || id:UKB-a:315	-0.001	0.005	0.858
Alanine || id:469	NA	NA	NA
Alanine || id:840	-0.018	0.032	0.602
Albumin || id:841	NA	NA	NA
Alcohol intake frequency. || id:UKB-a:25	-0.003	0.004	0.500
Alcohol intake versus 10 years previously || id:UKB-a:32	0.018	0.015	0.273
Alcohol usually taken with meals || id:UKB-a:31	0.010	0.040	0.812
Alpha-hydroxyisovalerate || id:616	0.009	0.027	0.796
Alzheimer's disease || id:297	0	0.018	0.997
Alzheimer's disease || id:298	0	0.014	0.988
Amyotrophic lateral sclerosis || id:1085	-0.046	0.031	0.200

Amyotrophic lateral sclerosis || id:1086	-0.018	0.053	0.765
Androsterone sulfate || id:460	0.022	0.015	0.381
Apolipoprotein A-I || id:842	-0.011	0.017	0.540
Apolipoprotein B || id:843	0.013	0.010	0.204
Arachidonic acid (20:4n6) || id:1141	0.006	0.005	0.259
Arm fat percentage (left) || id:UKB-a:286	0.003	0.004	0.385
Asparagine || id:638	NA	NA	NA
Asthma || id:44	0.022	0.034	0.541
Average number of double bonds in a fatty acid chain || id:851	-0.020	0.016	0.293
Average number of methylene groups in a fatty acid chain || id:848	-0.003	0.029	0.930
Average number of methylene groups per double bond || id:847	0.006	0.017	0.739
Average weekly beer plus cider intake || id:UKB-a:28	0.002	0.020	0.929
Average weekly champagne plus white wine intake || id:UKB-a:27	NA	NA	NA
Average weekly red wine intake || id:UKB-a:26	-0.012	0.013	0.367
Betaine || id:362	-0.015	0.027	0.673
Bilateral oophorectomy (both ovaries removed) || id:UKB-a:325	0.016	0.048	0.769
Bipolar disorder || id:800	-0.035	0.047	0.535
Bipolar disorder || id:801	-0.035	0.047	0.535
Birth length || id:29	NA	NA	NA
Birth weight || id:1083	-0.009	0.010	0.373
Birth weight || id:1084	-0.005	0.009	0.596
Birth weight || id:27	0.058	0.041	0.293
Birth weight || id:UKB-a:198	0.000	0.006	0.972
Birth weight of first child || id:UKB-a:318	-0.007	0.011	0.521
Body mass index || id:2	0.002	0.005	0.750
Body mass index || id:835	-0.003	0.006	0.675
Bradykinin des-arg(9) || id:656	0.037	0.027	0.400
Breastfed as a baby || id:UKB-a:33	NA	NA	NA
Bring up phlegm/sputum/mucus on most days || id:UKB-a:259	-0.028	0.182	0.903
Butyrylcarnitine || id:476	-0.008	0.010	0.478
C-reactive protein || id:1015	-0.079	0.039	0.290
Cancer code self-reported: basal cell carcinoma || id:UKB-a:59	-0.006	0.018	0.740
Cancer code self-reported: breast cancer || id:UKB-a:55	-0.002	0.012	0.880
Cancer code self-reported: lung cancer || id:UKB-a:54	0.076	0.044	0.227
Cancer code self-reported: malignant melanoma || id:UKB-a:58	-0.034	0.033	0.417
Cancer code self-reported: prostate cancer || id:UKB-a:57	-0.008	0.016	0.605
Cancer code self-reported: small intestine/small bowel cancer || id:UKB-a:56	-0.055	0.192	0.801
Cancer code self-reported: squamous cell carcinoma || id:UKB-a:60	0.057	0.123	0.724
Cancer diagnosed by doctor || id:UKB-a:307	-0.064	0.168	0.730
Caprylate (8:0) || id:481	NA	NA	NA
Cardioembolic stroke || id:1109	NA	NA	NA
Carnitine || id:379	-0.004	0.008	0.592

CD123 on 11c+123+DC || id:159	-0.017	0.035	0.657
CD32 on 11c+123+DC || id:162	NA	NA	NA
CD34 on Stem || id:151	NA	NA	NA
Celiac disease || id:1058	0.004	0.009	0.657
Celiac disease || id:1059	-0.006	0.030	0.839
Celiac disease || id:1060	-0.136	0.049	0.040
Celiac disease || id:276	-0.007	0.029	0.818
Childhood obesity || id:1096	0.138	0.058	0.099
Cholesterol || id:307	NA	NA	NA
Cholesterol esters in large HDL || id:875	-0.024	0.015	0.136
Cholesterol esters in large VLDL || id:881	0.014	0.007	0.042
Cholesterol esters in large VLDL || id:887	0.002	0.014	0.872
Cholesterol esters in medium HDL || id:899	-0.041	0.020	0.092
Chronic kidney disease || id:1102	-0.038	0.027	0.292
Chronotype || id:1087	-0.005	0.018	0.792
cis-trans-18:2 || id:1150	-0.001	0.018	0.948
Citrate || id:341	-0.004	0.024	0.873
Citrate || id:849	-0.047	0.068	0.540
Citrulline || id:356	-0.098	0.122	0.505
College completion || id:836	0.017	0.102	0.895
Comparative body size at age 10 || id:UKB-a:34	0.001	0.003	0.730
Concentration of IDL particles || id:870	0.015	0.008	0.088
Concentration of large HDL particles || id:878	-0.022	0.017	0.233
Concentration of large LDL particles || id:884	0.013	0.007	0.065
Concentration of large VLDL particles || id:890	-0.003	0.016	0.859
Copper || id:1073	NA	NA	NA
Coronary heart disease || id:6	-0.007	0.072	0.935
Coronary heart disease || id:7	-0.011	0.007	0.137
Coronary heart disease || id:8	-0.019	0.013	0.164
Coronary heart disease || id:9	-0.018	0.015	0.271
Creatinine (enzymatic) in urine || id:UKB-a:333	0.022	0.018	0.236
Creatinine || id:850	0.009	0.039	0.827
Crohn's disease || id:10	0.000	0.004	0.964
Crohn's disease || id:11	-0.013	0.014	0.386
Crohn's disease || id:12	-0.004	0.005	0.396
Crohn's disease || id:30	-0.003	0.006	0.563
Current tobacco smoking || id:UKB-a:16	0.033	0.018	0.081
Daytime dozing / sleeping (narcolepsy) || id:UKB-a:15	-0.015	0.022	0.498
Decanoylcarnitine || id:618	-0.022	0.046	0.716
Dehydroisoandrosterone sulfate (DHEA-S) || id:478	NA	NA	NA
Diabetes diagnosed by doctor || id:UKB-a:306	-0.003	0.006	0.602
Diagnoses - main ICD10: B37 Candidiasis || id:UKB-a:517	-0.017	0.053	0.758

Diagnoses - main ICD10: C44 Other malignant neoplasms of skin || id:UKB-a:518	0.000	0.014	0.981
Diagnoses - main ICD10: C50 Malignant neoplasm of breast || id:UKB-a:519	0.015	0.019	0.448
Diagnoses - main ICD10: C61 Malignant neoplasm of prostate || id:UKB-a:520	-0.010	0.029	0.752
Diagnoses - main ICD10: D12 Benign neoplasm of colon rectum anus and anal canal || id:UKB-a:521	0.018	0.070	0.842
Diagnoses - main ICD10: D25 Leiomyoma of uterus || id:UKB-a:522	0.110	0.049	0.155
Diagnoses - main ICD10: E03 Other hypothyroidism || id:UKB-a:523	0.038	0.100	0.716
Diagnoses - main ICD10: E04 Other non-toxic goitre || id:UKB-a:524	0.005	0.046	0.916
Diagnoses - main ICD10: G56 Mononeuropathies of upper limb || id:UKB-a:528	-0.019	0.036	0.623
Diagnoses - main ICD10: H25 Senile cataract || id:UKB-a:529	NA	NA	NA
Diagnoses - main ICD10: H26 Other cataract || id:UKB-a:530	0.107	0.080	0.406
Diagnoses - main ICD10: I10 Essential (primary) hypertension || id:UKB-a:531	NA	NA	NA
Diagnoses - main ICD10: I20 Angina pectoris || id:UKB-a:532	NA	NA	NA
Diagnoses - main ICD10: I21 Acute myocardial infarction || id:UKB-a:533	-0.012	0.046	0.833
Diagnoses - main ICD10: I25 Chronic ischaemic heart disease || id:UKB-a:534	-0.014	0.010	0.170
Diagnoses - main ICD10: I30 Acute pericarditis || id:UKB-a:535	0.005	0.055	0.929
Diagnoses - main ICD10: I48 Atrial fibrillation and flutter || id:UKB-a:536	0.004	0.010	0.676
Diagnoses - main ICD10: I80 Phlebitis and thrombophlebitis || id:UKB-a:537	0.021	0.011	0.135
Diagnoses - main ICD10: I83 Varicose veins of lower extremities || id:UKB-a:538	0.008	0.015	0.599
Diagnoses - main ICD10: I84 Haemorrhoids || id:UKB-a:539	NA	NA	NA
Diagnoses - main ICD10: J33 Nasal polyp || id:UKB-a:541	0.009	0.025	0.726
Diagnoses - main ICD10: J44 Other chronic obstructive pulmonary disease || id:UKB-a:543	NA	NA	NA
Diagnoses - main ICD10: K40 Inguinal hernia || id:UKB-a:549	-0.010	0.018	0.590
Diagnoses - main ICD10: K50 Crohn's disease [regional enteritis] || id:UKB-a:552	-0.016	0.030	0.700
Diagnoses - main ICD10: K51 Ulcerative colitis || id:UKB-a:553	NA	NA	NA
Diagnoses - main ICD10: K57 Diverticular disease of intestine || id:UKB-a:555	NA	NA	NA
Diagnoses - main ICD10: K76 Other diseases of liver || id:UKB-a:558	-0.032	0.022	0.281
Diagnoses - main ICD10: K80 Cholelithiasis || id:UKB-a:559	-0.006	0.007	0.412
Diagnoses - main ICD10: L03 Cellulitis || id:UKB-a:560	-0.016	0.025	0.636
Diagnoses - main ICD10: M10 Gout || id:UKB-a:561	0.149	0.294	0.648
Diagnoses - main ICD10: M17 Gonarthrosis [arthrosis of knee] || id:UKB-a:563	NA	NA	NA
Diagnoses - main ICD10: M21 Other acquired deformities of limbs || id:UKB-a:565	0.000	0.019	0.992
Diagnoses - main ICD10: M72 Fibroblastic disorders || id:UKB-a:572	0.003	0.008	0.724
Diagnoses - main ICD10: N19 Unspecified renal failure || id:UKB-a:573	0.005	0.077	0.950
Diagnoses - main ICD10: N20 Calculus of kidney and ureter || id:UKB-a:574	-0.012	0.183	0.958
Diagnoses - main ICD10: N40 Hyperplasia of prostate || id:UKB-a:576	-0.004	0.034	0.919
Diagnoses - main ICD10: N81 Female genital prolapse || id:UKB-a:577	-0.028	0.038	0.601
Diagnoses - main ICD10: O75 Other complications of labour and delivery not elsewhere classified || id:UKB-a:579	-0.128	0.338	0.718
Diagnoses - main ICD10: R31 Unspecified haematuria || id:UKB-a:585	0.038	0.088	0.743
Diastolic blood pressure automated reading || id:UKB-a:359	0.000	0.005	0.966
Difficulty not smoking for 1 day || id:UKB-a:344	-0.015	0.034	0.703
Dihomo-gamma-linolenic acid (20:3n6) || id:1140	0.015	0.007	0.042
Dihomo-linolenate (20:3n3 or n6) || id:712	NA	NA	NA

Docosapentaenoic acid (22:5n3) || id:1144	0.009	0.017	0.673
Doctor diagnosed asthma || id:UKB-a:255	-0.005	0.014	0.749
Doctor diagnosed hayfever or allergic rhinitis || id:UKB-a:254	-0.016	0.017	0.381
Drive faster than motorway speed limit || id:UKB-a:8	-0.023	0.032	0.499
Duration of vigorous activity || id:UKB-a:512	NA	NA	NA
Duration of walks || id:UKB-a:507	-0.076	0.128	0.610
Eicosapentaenoic acid (20:5n3) || id:1147	0.004	0.029	0.911
Epiandrosterone sulfate || id:627	NA	NA	NA
Erythronate* || id:578	0.019	0.073	0.835
Ever depressed for a whole week || id:UKB-a:373	-0.021	0.058	0.749
Ever smoked || id:UKB-a:236	0.009	0.012	0.436
Ever used hormone-replacement therapy (HRT) || id:UKB-a:324	0.013	0.017	0.514
Extreme body mass index || id:85	-0.005	0.046	0.924
Extreme height || id:86	0.005	0.010	0.616
Extreme waist-to-hip ratio || id:87	NA	NA	NA
Falls in the last year || id:UKB-a:262	-0.184	0.094	0.302
Fed-up feelings || id:UKB-a:49	-0.009	0.022	0.674
Ferritin || id:1050	0.024	0.017	0.280
Fluid intelligence score || id:UKB-a:196	0.000	0.014	0.996
Forced expiratory volume in 1-second (FEV1) Best measure || id:UKB-a:231	-0.011	0.005	0.044
Forced expiratory volume in 1-second (FEV1) predicted || id:UKB-a:234	-0.005	0.007	0.468
Forced expiratory volume in 1-second (FEV1) predicted percentage || id:UKB-a:235	0.005	0.013	0.722
Forced expiratory volume in 1-second (FEV1) || id:UKB-a:337	-0.013	0.005	0.010
Forced vital capacity (FVC) Best measure || id:UKB-a:232	-0.005	0.004	0.239
Fractured/broken bones in last 5 years || id:UKB-a:308	0.029	0.016	0.151
Free cholesterol || id:858	0.019	0.012	0.153
Free cholesterol in IDL || id:868	0.008	0.007	0.275
Free cholesterol in large HDL || id:876	-0.021	0.012	0.110
Free cholesterol in large LDL || id:882	0.014	0.007	0.063
Free cholesterol in large VLDL || id:888	0.010	0.018	0.594
Free cholesterol in medium HDL || id:900	-0.013	0.021	0.585
Free cholesterol to esterified cholesterol ratio || id:853	0.007	0.009	0.425
Frequency of depressed mood in last 2 weeks || id:UKB-a:242	0.013	0.043	0.769
Frequency of stair climbing in last 4 weeks || id:UKB-a:514	-0.232	0.125	0.160
Frequency of tenseness / restlessness in last 2 weeks || id:UKB-a:244	0.016	0.020	0.456
Frequency of tiredness / lethargy in last 2 weeks || id:UKB-a:245	0.000	0.013	0.988
Frequency of unenthusiasm / disinterest in last 2 weeks || id:UKB-a:243	-0.028	0.041	0.566
Frequency of walking for pleasure in last 4 weeks || id:UKB-a:515	NA	NA	NA
Gamma-glutamylglutamine || id:359	-0.003	0.032	0.948
Gamma-glutamylphenylalanine || id:572	NA	NA	NA
Gamma-glutamyltyrosine || id:360	0.034	0.050	0.547
Gamma-linolenic acid (18:3n6) || id:1139	0.009	0.005	0.066

Getting up in morning || id:UKB-a:10	-0.012	0.009	0.202
Glucose || id:859	0.020	0.032	0.598
Glutamine || id:860	0.017	0.020	0.448
Glutaroyl carnitine || id:699	-0.004	0.023	0.855
Glycoprotein acetyls || id:863	0.001	0.027	0.968
Glycoproteins || id:862	0.001	0.010	0.912
Gout || id:1054	NA	NA	NA
Guilty feelings || id:UKB-a:240	0.018	0.033	0.601
Had menopause || id:UKB-a:316	0.019	0.014	0.219
Haemoglobin concentration || id:270	-0.021	0.015	0.195
Hand grip strength (left) || id:UKB-a:374	0.005	0.007	0.477
HDL cholesterol || id:299	0.010	0.004	0.008
Headaches for 3+ months || id:UKB-a:354	NA	NA	NA
Hearing difficulty/problems with background noise || id:UKB-a:261	0.000	0.025	0.998
Hearing difficulty/problems: Yes || id:UKB-a:257	0.016	0.011	0.146
Heart rate || id:1056	0.000	0.016	0.985
Heel bone mineral density (BMD) T-score automated (left) || id:UKB-a:361	0.001	0.003	0.757
Heel bone mineral density (BMD) T-score automated (right) || id:UKB-a:362	0.001	0.003	0.810
Height || id:1034	-0.266	0.176	0.270
Heptanoate (7:0) || id:349	NA	NA	NA
Hexadecanedioate || id:711	0.021	0.039	0.688
Hexanoylcarnitine || id:467	0.017	0.028	0.606
High grade serous ovarian cancer || id:1121	-0.001	0.011	0.905
Hip circumference || id:100	NA	NA	NA
Hip circumference || id:101	0.043	0.076	0.615
Hip circumference || id:48	0.000	0.009	0.986
Hip circumference || id:49	0.008	0.007	0.246
Hip circumference || id:50	0.006	0.013	0.648
Hip circumference || id:51	0.008	0.016	0.610
Hip circumference || id:52	0.043	0.021	0.060
Hip circumference || id:53	0.048	0.024	0.068
Hip circumference || id:54	0.011	0.007	0.126
Hip circumference || id:55	0.004	0.007	0.550
Hip circumference || id:56	0.002	0.010	0.820
Hip circumference || id:57	0.004	0.011	0.708
Hip circumference || id:58	0.005	0.013	0.714
Hip circumference || id:59	0.008	0.013	0.518
Hippocampus volume || id:1045	NA	NA	NA
Histidine || id:866	-0.054	0.071	0.497
Home area population density - urban or rural: Scotland - Large Urban Area || id:UKB-a:228	-0.009	0.004	0.018
HWESASXX* || id:526	NA	NA	NA
Hydroxyisovaleroyl carnitine || id:698	NA	NA	NA

Illnesses of father: Chronic bronchitis/emphysema || id:UKB-a:206	NA	NA	NA
Illnesses of father: Diabetes || id:UKB-a:208	-0.009	0.011	0.403
Illnesses of father: Heart disease || id:UKB-a:201	-0.020	0.012	0.119
Illnesses of father: High blood pressure || id:UKB-a:207	-0.027	0.061	0.683
Illnesses of father: Lung cancer || id:UKB-a:205	0.100	0.074	0.406
Illnesses of father: None of the above (group 1) || id:UKB-a:202	-0.004	0.031	0.910
Illnesses of father: None of the above (group 2) || id:UKB-a:203	NA	NA	NA
Illnesses of father: Prostate cancer || id:UKB-a:204	-0.009	0.019	0.675
Illnesses of mother: Alzheimer's disease/dementia || id:UKB-a:210	-0.014	0.013	0.388
Illnesses of mother: Breast cancer || id:UKB-a:213	0.004	0.016	0.802
Illnesses of mother: Chronic bronchitis/emphysema || id:UKB-a:214	NA	NA	NA
Illnesses of mother: Diabetes || id:UKB-a:216	-0.009	0.011	0.430
Illnesses of mother: Heart disease || id:UKB-a:209	-0.016	0.023	0.551
Illnesses of mother: High blood pressure || id:UKB-a:215	-0.030	0.026	0.298
Illnesses of mother: None of the above (group 1) || id:UKB-a:211	0.011	0.041	0.814
Illnesses of siblings: Diabetes || id:UKB-a:223	-0.027	0.026	0.378
Illnesses of siblings: Heart disease || id:UKB-a:217	0.016	0.031	0.700
Illnesses of siblings: High blood pressure || id:UKB-a:222	0.006	0.037	0.865
Illnesses of siblings: None of the above (group 1) || id:UKB-a:218	0.036	0.052	0.509
Immunoglobulin G index levels || id:1017	NA	NA	NA
Indoleacetate || id:446	NA	NA	NA
Inflammatory bowel disease || id:292	-0.001	0.003	0.776
Inflammatory bowel disease || id:293	-0.012	0.018	0.524
Inflammatory bowel disease || id:294	0.002	0.004	0.647
Inflammatory bowel disease || id:31	-0.005	0.006	0.449
Invasive mucinous ovarian cancer || id:1123	NA	NA	NA
Iron || id:1049	-0.014	0.034	0.747
Irritability || id:UKB-a:47	0.002	0.028	0.952
Isobutyrylcarnitine || id:573	-0.027	0.019	0.387
Isovalerylcarnitine || id:652	-0.104	0.128	0.566
Job involves heavy manual or physical work || id:UKB-a:503	0.057	0.027	0.050
Job involves mainly walking or standing || id:UKB-a:502	-0.012	0.076	0.885
Kynurenine || id:375	-0.019	0.034	0.625
Lactate || id:894	NA	NA	NA
Laurate (12:0) || id:350	NA	NA	NA
LDL cholesterol || id:300	0.000	0.004	0.959
Leg fat percentage (left) || id:UKB-a:278	0.011	0.004	0.008
Leg fat percentage (right) || id:UKB-a:274	0.010	0.004	0.024
Length of menstrual cycle || id:UKB-a:351	0.007	0.017	0.699
Leucine || id:306	-0.004	0.026	0.881
Leucine || id:897	-0.002	0.060	0.976
Levulinate (4-oxovalerate) || id:433	NA	NA	NA

Light smokers at least 100 smokes in lifetime || id:UKB-a:313	-0.031	0.024	0.324
Linoleic acid (18:2n6) || id:1142	-0.005	0.005	0.326
Loneliness isolation || id:UKB-a:239	0.016	0.039	0.706
Long-standing illness disability or infirmity || id:UKB-a:252	-0.041	0.015	0.020
MDC:%32+ || id:266	-0.005	0.006	0.382
Mean cell haemoglobin || id:271	-0.007	0.006	0.246
Mean cell haemoglobin concentration || id:272	0.016	0.023	0.507
Mean cell volume || id:273	-0.002	0.006	0.800
Mean diameter for HDL particles || id:865	-0.007	0.013	0.581
Mean diameter for LDL particles || id:896	0.011	0.016	0.489
Mean platelet volume || id:1006	0.002	0.008	0.787
Mean time to correctly identify matches || id:UKB-a:199	0.006	0.025	0.804
Medication for cholesterol blood pressure or diabetes: Blood pressure medication || id:UKB-a:490	0.013	0.011	0.230
Medication for cholesterol blood pressure or diabetes: Cholesterol lowering medication || id:UKB-a:488	0.001	0.007	0.867
Medication for cholesterol blood pressure or diabetes: Insulin || id:UKB-a:491	-0.059	0.027	0.096
Medication for cholesterol blood pressure or diabetes: None of the above || id:UKB-a:489	0.011	0.009	0.198
Mineral and other dietary supplements: Calcium || id:UKB-a:495	NA	NA	NA
Mineral and other dietary supplements: Fish oil (including cod liver oil) || id:UKB-a:492	NA	NA	NA
Mineral and other dietary supplements: Glucosamine || id:UKB-a:494	NA	NA	NA
Mineral and other dietary supplements: None of the above || id:UKB-a:493	NA	NA	NA
Morning/evening person (chronotype) || id:UKB-a:11	0.006	0.007	0.367
Multiple sclerosis || id:1024	-0.002	0.006	0.777
Multiple sclerosis || id:1025	-0.005	0.005	0.328
Myo-inositol || id:417	NA	NA	NA
N-acetylglycine || id:449	NA	NA	NA
N2-dimethylguanosine || id:679	NA	NA	NA
Nap during day || id:UKB-a:12	-0.008	0.011	0.485
Nervous feelings || id:UKB-a:50	0.043	0.022	0.063
Neuroblastoma || id:816	0.125	0.077	0.353
Neuroticism || id:1007	0.006	0.048	0.899
Neuroticism score || id:UKB-a:230	0.020	0.011	0.071
NKeff:%314-158a+ || id:241	0.004	0.007	0.512
Non-cancer illness code self-reported: angina || id:UKB-a:62	-0.008	0.012	0.515
Non-cancer illness code self-reported: ankylosing spondylitis || id:UKB-a:88	NA	NA	NA
Non-cancer illness code self-reported: asthma || id:UKB-a:66	-0.002	0.006	0.736
Non-cancer illness code self-reported: cholelithiasis/gall stones || id:UKB-a:71	0.006	0.012	0.615
Non-cancer illness code self-reported: crohns disease || id:UKB-a:103	-0.017	0.017	0.379
Non-cancer illness code self-reported: deep venous thrombosis (dvt) || id:UKB-a:65	0.019	0.012	0.164
Non-cancer illness code self-reported: diabetes || id:UKB-a:74	0.002	0.007	0.745
Non-cancer illness code self-reported: diverticular disease/diverticulitis || id:UKB-a:102	NA	NA	NA
Non-cancer illness code self-reported: enlarged prostate || id:UKB-a:95	-0.014	0.035	0.715
Non-cancer illness code self-reported: glaucoma || id:UKB-a:79	-0.004	0.018	0.838

Non-cancer illness code self-reported: gout || id:UKB-a:107	-0.008	0.007	0.300
Non-cancer illness code self-reported: hayfever/allergic rhinitis || id:UKB-a:94	0.000	0.016	0.987
Non-cancer illness code self-reported: heart attack/myocardial infarction || id:UKB-a:63	-0.006	0.016	0.740
Non-cancer illness code self-reported: high cholesterol || id:UKB-a:108	-0.001	0.005	0.777
Non-cancer illness code self-reported: hypertension || id:UKB-a:61	0.006	0.004	0.151
Non-cancer illness code self-reported: hyperthyroidism/thyrotoxicosis || id:UKB-a:76	0.000	0.024	0.990
Non-cancer illness code self-reported: hypertrophic cardiomyopathy (hcm / hocm) || id:UKB-a:114	-0.067	0.038	0.095
Non-cancer illness code self-reported: hypopituitarism || id:UKB-a:98	-0.064	0.135	0.648
Non-cancer illness code self-reported: hypothyroidism/myxoedema || id:UKB-a:77	0.003	0.005	0.590
Non-cancer illness code self-reported: kidney stone/ureter stone/bladder stone || id:UKB-a:72	0.047	0.382	0.922
Non-cancer illness code self-reported: malabsorption/coeliac disease || id:UKB-a:101	0.009	0.011	0.428
Non-cancer illness code self-reported: migraine || id:UKB-a:78	0.020	0.023	0.413
Non-cancer illness code self-reported: nasal polyps || id:UKB-a:97	0.060	0.056	0.361
Non-cancer illness code self-reported: osteoarthritis || id:UKB-a:106	NA	NA	NA
Non-cancer illness code self-reported: osteoporosis || id:UKB-a:87	0.052	0.068	0.470
Non-cancer illness code self-reported: pernicious anaemia || id:UKB-a:90	-0.037	0.038	0.510
Non-cancer illness code self-reported: pneumothorax || id:UKB-a:111	0.055	0.119	0.673
Non-cancer illness code self-reported: polio / poliomyelitis || id:UKB-a:112	0.283	0.126	0.110
Non-cancer illness code self-reported: psoriasis || id:UKB-a:100	-0.001	0.006	0.878
Non-cancer illness code self-reported: pulmonary embolism +/- dvt || id:UKB-a:64	0.029	0.021	0.242
Non-cancer illness code self-reported: rheumatoid arthritis || id:UKB-a:105	-0.022	0.028	0.494
Non-cancer illness code self-reported: type 2 diabetes || id:UKB-a:75	-0.021	0.034	0.650
Non-cancer illness code self-reported: ulcerative colitis || id:UKB-a:104	-0.003	0.022	0.884
Non-cancer illness code self-reported: uterine fibroids || id:UKB-a:91	0.090	0.030	0.057
Non-cancer illness code self-reported: varicose veins || id:UKB-a:110	NA	NA	NA
Non-cancer illness code self-reported: vitiligo || id:UKB-a:115	-0.015	0.029	0.619
Number of children fathered || id:UKB-a:304	NA	NA	NA
Number of cigarettes currently smoked daily (current cigarette smokers) || id:UKB-a:342	0.001	0.046	0.985
Number of cigarettes previously smoked daily || id:UKB-a:328	0.003	0.026	0.936
Number of days/week of moderate physical activity 10+ minutes || id:UKB-a:508	0.021	0.043	0.652
Number of days/week of vigorous physical activity 10+ minutes || id:UKB-a:511	-0.093	0.761	0.913
Number of days/week walked 10+ minutes || id:UKB-a:506	0.059	0.031	0.089
Number of incorrect matches in round || id:UKB-a:356	-0.020	0.019	0.319
Number of live births || id:UKB-a:317	NA	NA	NA
Number of operations self-reported || id:UKB-a:23	-0.007	0.026	0.785
Number of self-reported cancers || id:UKB-a:21	-0.018	0.025	0.499
Number of self-reported non-cancer illnesses || id:UKB-a:22	-0.010	0.017	0.565
Number of treatments/medications taken || id:UKB-a:24	0.010	0.014	0.506
Obesity class 1 || id:90	0.025	0.015	0.121
Octadecanedioate || id:735	NA	NA	NA
Octanoylcarnitine || id:615	NA	NA	NA
Omega-3 fatty acids || id:855	0.009	0.028	0.776

Omega-6 fatty acids || id:856	0.004	0.014	0.773
Omega-9 and saturated fatty acids || id:857	-0.059	0.026	0.068
Other serious medical condition/disability diagnosed by doctor || id:UKB-a:309	-0.050	0.040	0.424
Ovarian cancer || id:1120	-0.008	0.014	0.590
Overall health rating || id:UKB-a:251	0.011	0.014	0.439
Pack years adult smoking as proportion of life span exposed to smoking PREVIEW ONLY || id:UKB-a:238	0.004	0.012	0.718
Pack years of smoking PREVIEW ONLY || id:UKB-a:237	-0.008	0.013	0.568
Packed cell volume || id:274	-0.015	0.027	0.588
Pain type(s) experienced in last month: Back pain || id:UKB-a:473	-0.042	0.025	0.139
Pain type(s) experienced in last month: Headache || id:UKB-a:469	0.002	0.010	0.814
Pain type(s) experienced in last month: Hip pain || id:UKB-a:475	-0.008	0.028	0.815
Pain type(s) experienced in last month: Knee pain || id:UKB-a:476	-0.044	0.035	0.258
Pain type(s) experienced in last month: Neck or shoulder pain || id:UKB-a:472	NA	NA	NA
Pain type(s) experienced in last month: None of the above || id:UKB-a:470	0.040	0.026	0.142
palmitoleic acid (16:1n7) || id:1156	0.016	0.043	0.732
Parkinson's disease || id:812	0.025	0.073	0.769
Past tobacco smoking || id:UKB-a:17	-0.003	0.015	0.843
Peak expiratory flow (PEF) || id:UKB-a:338	-0.001	0.008	0.909
Percent emphysema || id:1005	NA	NA	NA
PGC cross-disorder traits || id:803	-0.126	0.048	0.122
Phospholipids in IDL || id:871	0.011	0.007	0.136
Phospholipids in large HDL || id:879	-0.018	0.018	0.322
Phospholipids in large LDL || id:885	0.017	0.007	0.031
Phospholipids in large VLDL || id:891	-0.014	0.019	0.484
Platelet count || id:1008	-0.001	0.009	0.921
Potassium in urine || id:UKB-a:334	-0.015	0.034	0.675
Primary sclerosing cholangitis || id:1112	-0.008	0.010	0.417
Proline || id:355	0.010	0.017	0.671
Propionylcarnitine || id:479	-0.025	0.019	0.327
Prospective memory result || id:UKB-a:197	NA	NA	NA
Pulse rate || id:UKB-a:363	0.000	0.006	0.964
Pulse wave peak to peak time || id:UKB-a:365	NA	NA	NA
Pulse wave reflection index || id:UKB-a:364	NA	NA	NA
Putamen volume || id:1047	0.031	0.032	0.431
Pyroglutamine* || id:501	-0.026	0.027	0.437
Ratio of bisallylic groups to double bonds || id:844	-0.016	0.014	0.319
Ratio of bisallylic groups to total fatty acids || id:845	-0.015	0.015	0.422
Reason for reducing amount of alcohol drunk: Health precaution || id:UKB-a:314	NA	NA	NA
Red blood cell count || id:275	-0.001	0.010	0.890
Rheumatoid arthritis || id:831	-0.009	0.012	0.477
Rheumatoid arthritis || id:832	-0.002	0.007	0.722
Rheumatoid arthritis || id:833	-0.005	0.006	0.397

Rheumatoid arthritis || id:834	0.009	0.013	0.530
Risk taking || id:UKB-a:241	0.037	0.022	0.140
Schizophrenia || id:22	0.007	0.009	0.444
Seen a psychiatrist for nerves anxiety tension or depression || id:UKB-a:247	0.024	0.042	0.674
Seen doctor (GP) for nerves anxiety tension or depression || id:UKB-a:246	-0.001	0.024	0.956
Sensitivity / hurt feelings || id:UKB-a:48	0.015	0.013	0.273
Serine || id:464	NA	NA	NA
Serum creatinine (eGFRcrea) || id:1104	0.013	0.009	0.172
Serum creatinine (eGFRcrea) || id:1105	0.006	0.009	0.536
Serum cystatin C (eGFRcys) || id:1106	0.014	0.021	0.553
Sitting height ratio || id:1070	0.049	0.078	0.592
Sleep duration || id:1088	0.001	0.076	0.992
Sleep duration || id:UKB-a:9	0.024	0.012	0.045
Sleeplessness / insomnia || id:UKB-a:13	-0.010	0.010	0.338
Smoking status: Current || id:UKB-a:225	0.023	0.019	0.236
Smoking status: Previous || id:UKB-a:224	-0.016	0.038	0.672
Snoring || id:UKB-a:14	0.016	0.018	0.361
Sodium in urine || id:UKB-a:335	0.002	0.012	0.901
Started insulin within one year diagnosis of diabetes || id:UKB-a:331	-0.002	0.023	0.943
Stearate (18:0) || id:331	NA	NA	NA
Stearic acid (18:0) plasma levels || id:1158	-0.139	0.224	0.646
Suffer from 'nerves' || id:UKB-a:200	-0.011	0.040	0.786
Systemic lupus erythematosus || id:815	0.019	0.139	0.912
Systemic lupus erythematosus || id:815	0.019	0.139	0.912
Systolic blood pressure automated reading || id:UKB-a:360	0.002	0.005	0.638
Taking other prescription medications || id:UKB-a:310	-0.026	0.027	0.366
Tense / 'highly strung' || id:UKB-a:52	0.041	0.033	0.230
Tetradecanedioate || id:709	0.043	0.033	0.416
Time from waking to first cigarette || id:UKB-a:343	-0.037	0.109	0.792
Time spent driving || id:UKB-a:7	NA	NA	NA
Time spent using computer || id:UKB-a:6	0.010	0.019	0.601
Time spent watching television (TV) || id:UKB-a:5	0.017	0.011	0.119
Tobacco smoking: Ex-smoker || id:UKB-a:260	NA	NA	NA
Total cholesterol || id:301	0.002	0.004	0.645
Total cholesterol in HDL || id:864	-0.007	0.014	0.628
Total cholesterol in IDL || id:867	0.010	0.007	0.162
Total cholesterol in large HDL || id:874	-0.024	0.013	0.081
Total cholesterol in large LDL || id:880	0.016	0.007	0.026
Total cholesterol in large VLDL || id:886	0.000	0.017	0.995
Total cholesterol in LDL || id:895	0.016	0.007	0.028
Total cholesterol in medium HDL || id:898	-0.019	0.018	0.353
Total lipids in IDL || id:869	0.012	0.007	0.100

Total lipids in large HDL || id:877	-0.022	0.017	0.240
Total lipids in large LDL || id:883	0.013	0.006	0.050
Total lipids in large VLDL || id:889	-0.013	0.019	0.533
Total lipids in medium HDL || id:901	-0.038	0.040	0.407
trans-cis-18:2 || id:1148	-0.081	0.233	0.786
Transferrin || id:1052	-0.009	0.008	0.307
Transferrin Saturation || id:1051	0.031	0.016	0.307
Treatment/medication code: allopurinol || id:UKB-a:142	-0.005	0.008	0.549
Treatment/medication code: amlodipine || id:UKB-a:150	0.015	0.014	0.310
Treatment/medication code: arcoxia 90mg tablet || id:UKB-a:185	-0.120	0.051	0.038
Treatment/medication code: aspirin || id:UKB-a:132	0.004	0.022	0.871
Treatment/medication code: atenolol || id:UKB-a:131	-0.029	0.035	0.448
Treatment/medication code: atorvastatin || id:UKB-a:176	0.002	0.010	0.819
Treatment/medication code: atrovent 20micrograms inhaler || id:UKB-a:123	-0.005	0.064	0.951
Treatment/medication code: becotide 50 inhaler || id:UKB-a:124	-0.038	0.051	0.595
Treatment/medication code: bendroflumethiazide || id:UKB-a:193	0.014	0.013	0.282
Treatment/medication code: bisoprolol || id:UKB-a:148	NA	NA	NA
Treatment/medication code: carbimazole || id:UKB-a:140	-0.013	0.035	0.724
Treatment/medication code: clopidogrel || id:UKB-a:180	NA	NA	NA
Treatment/medication code: doxazosin || id:UKB-a:149	NA	NA	NA
Treatment/medication code: eumovate cream || id:UKB-a:152	0.007	0.029	0.860
Treatment/medication code: ezetimibe || id:UKB-a:191	0.027	0.029	0.399
Treatment/medication code: flecainide || id:UKB-a:163	0.047	0.025	0.313
Treatment/medication code: gliclazide || id:UKB-a:139	-0.005	0.016	0.756
Treatment/medication code: hydroxocobalamin product || id:UKB-a:134	-0.033	0.129	0.807
Treatment/medication code: ibuprofen || id:UKB-a:136	NA	NA	NA
Treatment/medication code: indivina 1mg/2.5mg tablet || id:UKB-a:181	0.090	0.035	0.030
Treatment/medication code: insulin product || id:UKB-a:153	-0.043	0.021	0.097
Treatment/medication code: isosorbide dinitrate || id:UKB-a:119	0.047	0.036	0.232
Treatment/medication code: kapake tablet || id:UKB-a:125	0.030	0.035	0.421
Treatment/medication code: letrozole || id:UKB-a:174	0.000	0.026	0.994
Treatment/medication code: levothyroxine sodium || id:UKB-a:190	0.002	0.007	0.731
Treatment/medication code: liothyronine || id:UKB-a:157	0.000	0.034	0.996
Treatment/medication code: lisinopril || id:UKB-a:116	NA	NA	NA
Treatment/medication code: lisinopril+hydrochlorothiazide 10mg/12.5mg tablet || id:UKB-a:127	-0.078	0.056	0.223
Treatment/medication code: metformin || id:UKB-a:159	-0.003	0.009	0.695
Treatment/medication code: morphine || id:UKB-a:137	0.017	0.049	0.759
Treatment/medication code: nicorandil || id:UKB-a:165	NA	NA	NA
Treatment/medication code: paracetamol || id:UKB-a:194	0.071	0.036	0.091
Treatment/medication code: prednisolone || id:UKB-a:141	NA	NA	NA
Treatment/medication code: quinine || id:UKB-a:138	NA	NA	NA
Treatment/medication code: ramipril || id:UKB-a:117	0.001	0.033	0.976

Treatment/medication code: rhinocort 50micrograms nasal spray || id:UKB-a:144	0.003	0.036	0.945
Treatment/medication code: rosiglitazone || id:UKB-a:184	0.060	0.148	0.753
Treatment/medication code: salbutamol || id:UKB-a:151	-0.029	0.030	0.509
Treatment/medication code: seretide 50 evohaler || id:UKB-a:183	-0.003	0.060	0.964
Treatment/medication code: simvastatin || id:UKB-a:121	0.003	0.007	0.680
Treatment/medication code: testogel 50mg gel 5g sachet || id:UKB-a:192	0.020	0.019	0.320
Treatment/medication code: thyroxine product || id:UKB-a:158	-0.013	0.022	0.568
Treatment/medication code: tranexamic acid || id:UKB-a:120	NA	NA	NA
Treatment/medication code: venlafaxine || id:UKB-a:167	NA	NA	NA
Treatment/medication code: ventolin 100micrograms inhaler || id:UKB-a:122	-0.011	0.023	0.647
Treatment/medication code: warfarin || id:UKB-a:160	0.028	0.022	0.259
Triglycerides || id:302	-0.008	0.004	0.037
Triglycerides in IDL || id:872	0.014	0.011	0.238
Triglycerides in large VLDL || id:892	-0.005	0.019	0.807
Trunk fat percentage || id:UKB-a:290	0.002	0.005	0.667
Tryptophan || id:304	0.000	0.065	0.999
Type 2 diabetes || id:1090	NA	NA	NA
Type 2 diabetes || id:23	0.007	0.012	0.560
Type 2 diabetes || id:24	0.002	0.006	0.813
Type 2 diabetes || id:25	-0.003	0.008	0.699
Type 2 diabetes || id:26	-0.008	0.026	0.769
Types of physical activity in last 4 weeks: Heavy DIY (eg: weeding lawn mowing carpentry digging) || id:UKB-a:487	-0.084	0.149	0.675
Types of physical activity in last 4 weeks: Light DIY (eg: pruning watering the lawn) || id:UKB-a:486	NA	NA	NA
Types of physical activity in last 4 weeks: None of the above || id:UKB-a:483	0.009	0.035	0.811
Types of physical activity in last 4 weeks: Other exercises (eg: swimming cycling keep fit bowling) || id:UKB-a:484	-0.046	0.051	0.412
Types of physical activity in last 4 weeks: Strenuous sports || id:UKB-a:485	0.106	0.043	0.243
Types of physical activity in last 4 weeks: Walking for pleasure (not as a means of transport) || id:UKB-a:482	0.243	0.180	0.249
Types of transport used (excluding work): Cycle || id:UKB-a:481	-0.202	0.395	0.700
Types of transport used (excluding work): Walk || id:UKB-a:479	NA	NA	NA
Tyrosine || id:325	0.079	0.089	0.538
Ulcerative colitis || id:32	-0.001	0.009	0.914
Underlying (primary) cause of death: ICD10: J84.1 Other interstitial pulmonary diseases with fibrosis || id:UKB-a:358	-0.096	0.064	0.194
Urate || id:1055	-0.005	0.006	0.446
Uridine || id:316	-0.032	0.042	0.585
Usual walking pace || id:UKB-a:513	0.026	0.015	0.106
Waist circumference || id:102	NA	NA	NA
Waist circumference || id:103	NA	NA	NA
Waist circumference || id:105	0.001	0.031	0.988
Waist circumference || id:60	0.012	0.007	0.081
Waist circumference || id:61	0.004	0.010	0.686
Waist circumference || id:62	0.006	0.016	0.724
Waist circumference || id:63	0.008	0.016	0.610

Waist circumference || id:64	0.033	0.014	0.036
Waist circumference || id:65	0.040	0.014	0.017
Waist circumference || id:66	0.002	0.009	0.802
Waist circumference || id:67	0.006	0.009	0.537
Waist circumference || id:68	-0.002	0.012	0.896
Waist circumference || id:69	0.003	0.014	0.811
Waist circumference || id:70	-0.009	0.020	0.649
Waist circumference || id:71	-0.008	0.018	0.651
Waist-to-hip ratio || id:109	-0.137	0.120	0.336
Waist-to-hip ratio || id:111	-0.013	0.029	0.666
Waist-to-hip ratio || id:72	0.016	0.013	0.227
Waist-to-hip ratio || id:73	0.018	0.013	0.178
Waist-to-hip ratio || id:80	0.000	0.010	0.962
Waist-to-hip ratio || id:81	-0.004	0.010	0.707
Waist-to-hip ratio || id:82	-0.005	0.078	0.950
Waist-to-hip ratio || id:83	0.153	0.154	0.393
Weight || id:106	0.025	0.030	0.428
Weight || id:107	0.024	0.018	0.202
Worrier / anxious feelings || id:UKB-a:51	-0.016	0.018	0.366
Worry too long after embarrassment || id:UKB-a:53	0.039	0.027	0.171
X-02269 || id:485	-0.032	0.067	0.718
X-03056--N-[3-(2-Oxopyrrolidin-1-yl)propyl]acetamide || id:505	0.057	0.038	0.275
X-03094 || id:373	-0.001	0.039	0.981
X-08402 || id:426	NA	NA	NA
X-08988 || id:435	NA	NA	NA
X-10510 || id:444	-0.021	0.023	0.523
X-11204 || id:484	NA	NA	NA
X-11261 || id:490	NA	NA	NA
X-11315 || id:495	-0.013	0.042	0.808
X-11317 || id:496	NA	NA	NA
X-11327 || id:498	NA	NA	NA
X-11423--O-sulfo-L-tyrosine || id:509	NA	NA	NA
X-11440 || id:513	-0.029	0.016	0.325
X-11445--5-alpha-pregnan-3beta 20alpha-disulfate || id:517	0.062	0.123	0.701
X-11469 || id:519	-0.067	0.106	0.638
X-11491 || id:524	0.024	0.033	0.605
X-11529 || id:528	NA	NA	NA
X-11538 || id:531	0.017	0.020	0.545
X-11593--O-methylascorbate* || id:536	-0.021	0.016	0.251
X-11787 || id:541	NA	NA	NA
X-11792 || id:542	0.050	0.034	0.384
X-11793--oxidized bilirubin* || id:543	NA	NA	NA

X-11905 || id:560	NA	NA	NA
X-12040 || id:568	NA	NA	NA
X-12063 || id:570	0.012	0.011	0.338
X-12092 || id:580	0.002	0.009	0.800
X-12100--hydroxytryptophan* || id:584	NA	NA	NA
X-12188 || id:587	NA	NA	NA
X-12189 || id:588	NA	NA	NA
X-12244--N-acetylcarnosine || id:596	0.102	0.088	0.452
X-12456 || id:613	NA	NA	NA
X-12510--2-aminooctanoic acid || id:629	0.002	0.017	0.932
X-12524 || id:630	NA	NA	NA
X-12556 || id:633	NA	NA	NA
X-12644 || id:636	-0.047	0.066	0.603
X-12696 || id:640	NA	NA	NA
X-12712 || id:642	NA	NA	NA
X-12728 || id:646	0.060	0.062	0.366
X-12798 || id:661	0.006	0.013	0.716
X-12844 || id:665	-0.124	0.066	0.313
X-12850 || id:667	0.018	0.030	0.652
X-12855 || id:670	NA	NA	NA
X-13215 || id:675	NA	NA	NA
X-13429 || id:683	0.036	0.030	0.440
X-13431--nonanoylcarnitine* || id:684	NA	NA	NA
X-13435 || id:685	NA	NA	NA
X-13496 || id:691	NA	NA	NA
X-13548 || id:693	NA	NA	NA
X-14189--leucylalanine || id:719	NA	NA	NA
X-14205--alpha-glutamyltyrosine || id:720	NA	NA	NA
X-14208--phenylalanylserine || id:721	NA	NA	NA
X-14304--leucylalanine || id:722	NA	NA	NA
X-14626 || id:729	NA	NA	NA
X-18601 || id:396	0.154	0.071	0.277
Years of schooling || id:1001	0.019	0.013	0.142
Years of schooling || id:1010	0.017	0.043	0.696
Years of schooling || id:1011	0.010	0.023	0.672
Years of schooling || id:837	0.116	0.114	0.416
Zinc || id:1079	NA	NA	NA

id: specific code attributed to each trait on MR Base, se: standard error

Table S4. Heterogeneity analysis performed on 635 GWASes across 570 phenotypic traits

Trait	Method	Q	Q df	Q p-value
1 5-anhydroglucitol (1 5-AG) || id:419	Maximum likelihood	1.240	2	0.537
1 5-anhydroglucitol (1 5-AG) || id:419	MR Egger	0.147	1	0.701
1 5-anhydroglucitol (1 5-AG) || id:419	Inverse variance weighted	0.623	2	0.732
1-arachidonoylglycerophosphocholine* || id:558	Maximum likelihood	0.204	2	0.903
1-arachidonoylglycerophosphocholine* || id:558	MR Egger	0.100	1	0.752
1-arachidonoylglycerophosphocholine* || id:558	Inverse variance weighted	0.102	2	0.950
1-arachidonoylglycerophosphoethanolamine* || id:682	Maximum likelihood	0.608	2	0.738
1-arachidonoylglycerophosphoethanolamine* || id:682	MR Egger	0.019	1	0.890
1-arachidonoylglycerophosphoethanolamine* || id:682	Inverse variance weighted	0.304	2	0.859
1-arachidonoylglycerophosphoinositol* || id:634	Maximum likelihood	1.458	3	0.692
1-arachidonoylglycerophosphoinositol* || id:634	MR Egger	0.520	2	0.771
1-arachidonoylglycerophosphoinositol* || id:634	Inverse variance weighted	0.974	3	0.808
1-eicosatrienoylglycerophosphocholine* || id:602	Maximum likelihood	0	1	0.988
1-eicosatrienoylglycerophosphocholine* || id:602	Inverse variance weighted	0	1	1
1-linoleoylglycerophosphoethanolamine* || id:497	Maximum likelihood	0.221	1	0.638
1-linoleoylglycerophosphoethanolamine* || id:497	Inverse variance weighted	0	1	1
1-palmitoylglycerophosphoethanolamine || id:707	Maximum likelihood	0.693	1	0.405
1-palmitoylglycerophosphoethanolamine || id:707	Inverse variance weighted	0	1	1
10-undecenoate (11:1n1) || id:482	Maximum likelihood	0.893	1	0.345
10-undecenoate (11:1n1) || id:482	Inverse variance weighted	0	1	1
18,02 linoleic acid (LA) || id:893	Maximum likelihood	9.860	12	0.628
18,02 linoleic acid (LA) || id:893	MR Egger	9.569	11	0.569
18,02 linoleic acid (LA) || id:893	Inverse variance weighted	9.112	12	0.693
2-aminobutyrate || id:471	Maximum likelihood	0.416	1	0.519
2-aminobutyrate || id:471	Inverse variance weighted	0	1	1
2-hydroxyisobutyrate || id:427	Maximum likelihood	6.796	2	0.033
2-hydroxyisobutyrate || id:427	MR Egger	6.302	1	0.012
2-hydroxyisobutyrate || id:427	Inverse variance weighted	3.418	2	0.181
22,06 docosahexaenoic acid || id:852	Maximum likelihood	2.702	4	0.608
22,06 docosahexaenoic acid || id:852	MR Egger	2.634	3	0.451
22,06 docosahexaenoic acid || id:852	Inverse variance weighted	2.049	4	0.726
3-dehydrocarnitine* || id:500	Maximum likelihood	3.993	2	0.136
3-dehydrocarnitine* || id:500	MR Egger	2.737	1	0.098
3-dehydrocarnitine* || id:500	Inverse variance weighted	1.997	2	0.368
3-methyl-2-oxovalerate || id:383	Maximum likelihood	0.195	2	0.907
3-methyl-2-oxovalerate || id:383	MR Egger	0.028	1	0.868
3-methyl-2-oxovalerate || id:383	Inverse variance weighted	0.098	2	0.952
4-acetamidobutanoate || id:339	Maximum likelihood	4.809	1	0.028
4-acetamidobutanoate || id:339	Inverse variance weighted	0	1	1

4-methyl-2-oxopentanoate || id:429	Maximum likelihood	4.957	1	0.026
4-methyl-2-oxopentanoate || id:429	Inverse variance weighted	0	1	1
Acetoacetate || id:838	Maximum likelihood	4.338	1	0.037
Acetoacetate || id:838	Inverse variance weighted	0	1	1
Acetylcarnitine || id:463	Maximum likelihood	0.011	1	0.918
Acetylcarnitine || id:463	Inverse variance weighted	0	1	1
Acetylphosphate || id:378	Maximum likelihood	0.134	1	0.715
Acetylphosphate || id:378	Inverse variance weighted	0	1	1
Adiponectin || id:1	Maximum likelihood	11.960	13	0.531
Adiponectin || id:1	MR Egger	11.900	12	0.454
Adiponectin || id:1	Inverse variance weighted	11.048	13	0.607
ADpSGEGDFXAEGGGVR* || id:601	Maximum likelihood	1.538	1	0.215
ADpSGEGDFXAEGGGVR* || id:601	Inverse variance weighted	0	1	1
ADSGEGDFXAEGGGVR* || id:539	Maximum likelihood	0.027	1	0.869
ADSGEGDFXAEGGGVR* || id:539	Inverse variance weighted	0	1	1
Age at first live birth || id:UKB-a:319	Maximum likelihood	18.432	16	0.299
Age at first live birth || id:UKB-a:319	MR Egger	18.390	15	0.243
Age at first live birth || id:UKB-a:319	Inverse variance weighted	17.292	16	0.367
Age at last live birth || id:UKB-a:320	Maximum likelihood	6.628	3	0.085
Age at last live birth || id:UKB-a:320	MR Egger	5.050	2	0.080
Age at last live birth || id:UKB-a:320	Inverse variance weighted	4.420	3	0.220
Age at menarche || id:1095	Maximum likelihood	70.028	59	0.154
Age at menarche || id:1095	MR Egger	69.907	58	0.136
Age at menarche || id:1095	Inverse variance weighted	68.862	59	0.178
Age at menopause || id:1004	Maximum likelihood	53.982	37	0.035
Age at menopause || id:1004	MR Egger	53.994	36	0.027
Age at menopause || id:1004	Inverse variance weighted	52.541	37	0.047
Age completed full time education || id:UKB-a:505	Maximum likelihood	28.230	19	0.079
Age completed full time education || id:UKB-a:505	MR Egger	28.171	18	0.059
Age completed full time education || id:UKB-a:505	Inverse variance weighted	26.803	19	0.109
Age started oral contraceptive pill || id:UKB-a:323	Maximum likelihood	0.290	1	0.590
Age started oral contraceptive pill || id:UKB-a:323	Inverse variance weighted	0	1	1
Age when periods started (menarche) || id:UKB-a:315	Maximum likelihood	156.281	117	0.009
Age when periods started (menarche) || id:UKB-a:315	MR Egger	156.333	116	0.007
Age when periods started (menarche) || id:UKB-a:315	Inverse variance weighted	155.040	117	0.011
Alanine || id:469	Maximum likelihood	1.347	1	0.246
Alanine || id:469	Inverse variance weighted	0	1	1
Alanine || id:840	Maximum likelihood	2.001	5	0.849
Alanine || id:840	MR Egger	1.686	4	0.793
Alanine || id:840	Inverse variance weighted	1.605	5	0.901
Albumin || id:841	Maximum likelihood	0.002	1	0.964
Albumin || id:841	Inverse variance weighted	0	1	1

Alcohol intake frequency. || id:UKB-a:25	Maximum likelihood	41.177	41	0.463
Alcohol intake frequency. || id:UKB-a:25	MR Egger	40.707	40	0.439
Alcohol intake frequency. || id:UKB-a:25	Inverse variance weighted	40.174	41	0.507
Alcohol intake versus 10 years previously || id:UKB-a:32	Maximum likelihood	13.327	8	0.101
Alcohol intake versus 10 years previously || id:UKB-a:32	MR Egger	11.226	7	0.129
Alcohol intake versus 10 years previously || id:UKB-a:32	Inverse variance weighted	11.809	8	0.160
Alcohol usually taken with meals || id:UKB-a:31	Maximum likelihood	9.744	13	0.715
Alcohol usually taken with meals || id:UKB-a:31	MR Egger	9.686	12	0.643
Alcohol usually taken with meals || id:UKB-a:31	Inverse variance weighted	8.996	13	0.773
Alpha-hydroxyisovalerate || id:616	Maximum likelihood	0.159	2	0.923
Alpha-hydroxyisovalerate || id:616	MR Egger	0.050	1	0.824
Alpha-hydroxyisovalerate || id:616	Inverse variance weighted	0.080	2	0.961
Alzheimer's disease || id:297	Maximum likelihood	8.790	11	0.641
Alzheimer's disease || id:297	MR Egger	8.809	10	0.550
Alzheimer's disease || id:297	Inverse variance weighted	8.008	11	0.713
Alzheimer's disease || id:298	Maximum likelihood	24.962	18	0.126
Alzheimer's disease || id:298	MR Egger	24.971	17	0.095
Alzheimer's disease || id:298	Inverse variance weighted	23.584	18	0.169
Amyotrophic lateral sclerosis || id:1085	Maximum likelihood	8.719	6	0.190
Amyotrophic lateral sclerosis || id:1085	MR Egger	12.107	5	0.033
Amyotrophic lateral sclerosis || id:1085	Inverse variance weighted	14.480	6	0.025
Amyotrophic lateral sclerosis || id:1086	Maximum likelihood	3.298	3	0.348
Amyotrophic lateral sclerosis || id:1086	MR Egger	7.849	2	0.020
Amyotrophic lateral sclerosis || id:1086	Inverse variance weighted	5.538	3	0.136
Androsterone sulfate || id:460	Maximum likelihood	4.103	2	0.129
Androsterone sulfate || id:460	MR Egger	1.301	1	0.254
Androsterone sulfate || id:460	Inverse variance weighted	2.052	2	0.358
Apolipoprotein A-I || id:842	Maximum likelihood	4.379	9	0.885
Apolipoprotein A-I || id:842	MR Egger	3.977	8	0.859
Apolipoprotein A-I || id:842	Inverse variance weighted	3.900	9	0.918
Apolipoprotein B || id:843	Maximum likelihood	25.653	17	0.081
Apolipoprotein B || id:843	MR Egger	23.233	16	0.108
Apolipoprotein B || id:843	Inverse variance weighted	24.261	17	0.113
Arachidonic acid (20:4n6) || id:1141	Maximum likelihood	80.349	85	0.623
Arachidonic acid (20:4n6) || id:1141	MR Egger	79.060	84	0.632
Arachidonic acid (20:4n6) || id:1141	Inverse variance weighted	79.404	85	0.651
Arm fat percentage (left) || id:UKB-a:286	Maximum likelihood	359.587	247	0
Arm fat percentage (left) || id:UKB-a:286	MR Egger	358.482	246	0
Arm fat percentage (left) || id:UKB-a:286	Inverse variance weighted	358.131	247	0
Asparagine || id:638	Maximum likelihood	3.114	1	0.078
Asparagine || id:638	Inverse variance weighted	0	1	1
Asthma || id:44	Maximum likelihood	6.355	7	0.499

Asthma || id:44	MR Egger	5.967	6	0.427
Asthma || id:44	Inverse variance weighted	5.475	7	0.602
Average number of double bonds in a fatty acid chain || id:851	Maximum likelihood	3.468	4	0.483
Average number of double bonds in a fatty acid chain || id:851	MR Egger	1.849	3	0.604
Average number of double bonds in a fatty acid chain || id:851	Inverse variance weighted	2.601	4	0.627
Average number of methylene groups in a fatty acid chain || id:848	Maximum likelihood	0.850	2	0.654
Average number of methylene groups in a fatty acid chain || id:848	MR Egger	0.838	1	0.360
Average number of methylene groups in a fatty acid chain || id:848	Inverse variance weighted	0.425	2	0.808
Average number of methylene groups per double bond || id:847	Maximum likelihood	3.655	4	0.455
Average number of methylene groups per double bond || id:847	MR Egger	3.499	3	0.321
Average number of methylene groups per double bond || id:847	Inverse variance weighted	2.741	4	0.602
Average weekly beer plus cider intake || id:UKB-a:28	Maximum likelihood	20.702	10	0.023
Average weekly beer plus cider intake || id:UKB-a:28	MR Egger	20.761	9	0.014
Average weekly beer plus cider intake || id:UKB-a:28	Inverse variance weighted	18.702	10	0.044
Average weekly champagne plus white wine intake || id:UKB-a:27	Maximum likelihood	0.149	1	0.699
Average weekly champagne plus white wine intake || id:UKB-a:27	Inverse variance weighted	0	1	1
Average weekly red wine intake || id:UKB-a:26	Maximum likelihood	13.754	11	0.247
Average weekly red wine intake || id:UKB-a:26	MR Egger	12.631	10	0.245
Average weekly red wine intake || id:UKB-a:26	Inverse variance weighted	12.510	11	0.327
Betaine || id:362	Maximum likelihood	0.412	2	0.814
Betaine || id:362	MR Egger	0.094	1	0.760
Betaine || id:362	Inverse variance weighted	0.206	2	0.902
Bilateral oophorectomy (both ovaries removed) || id:UKB-a:325	Maximum likelihood	1.660	3	0.646
Bilateral oophorectomy (both ovaries removed) || id:UKB-a:325	MR Egger	1.547	2	0.461
Bilateral oophorectomy (both ovaries removed) || id:UKB-a:325	Inverse variance weighted	1.107	3	0.775
Bipolar disorder || id:800	Maximum likelihood	5.892	3	0.117
Bipolar disorder || id:800	MR Egger	4.623	2	0.099
Bipolar disorder || id:800	Inverse variance weighted	3.932	3	0.269
Bipolar disorder || id:801	Maximum likelihood	5.892	3	0.117
Bipolar disorder || id:801	MR Egger	4.623	2	0.099
Bipolar disorder || id:801	Inverse variance weighted	3.932	3	0.269
Birth length || id:29	Maximum likelihood	0.678	1	0.410
Birth length || id:29	Inverse variance weighted	0	1	1
Birth weight || id:1083	Maximum likelihood	55.116	42	0.084
Birth weight || id:1083	MR Egger	54.046	41	0.083
Birth weight || id:1083	Inverse variance weighted	53.804	42	0.105
Birth weight || id:1084	Maximum likelihood	54.713	45	0.152
Birth weight || id:1084	MR Egger	54.361	44	0.136
Birth weight || id:1084	Inverse variance weighted	53.497	45	0.180
Birth weight || id:27	Maximum likelihood	2.524	3	0.471
Birth weight || id:27	MR Egger	0.529	2	0.767
Birth weight || id:27	Inverse variance weighted	1.683	3	0.641

Birth weight || id:UKB-a:198	Maximum likelihood	93.713	80	0.140
Birth weight || id:UKB-a:198	MR Egger	93.731	79	0.123
Birth weight || id:UKB-a:198	Inverse variance weighted	92.561	80	0.159
Birth weight of first child || id:UKB-a:318	Maximum likelihood	50.154	41	0.155
Birth weight of first child || id:UKB-a:318	MR Egger	49.639	40	0.141
Birth weight of first child || id:UKB-a:318	Inverse variance weighted	48.937	41	0.185
Body mass index || id:2	Maximum likelihood	100.863	78	0.042
Body mass index || id:2	MR Egger	100.735	77	0.036
Body mass index || id:2	Inverse variance weighted	99.576	78	0.050
Body mass index || id:835	Maximum likelihood	84.476	67	0.073
Body mass index || id:835	MR Egger	84.251	66	0.064
Body mass index || id:835	Inverse variance weighted	83.217	67	0.087
Bradykinin des-arg(9) || id:656	Maximum likelihood	2.075	2	0.354
Bradykinin des-arg(9) || id:656	MR Egger	0.187	1	0.665
Bradykinin des-arg(9) || id:656	Inverse variance weighted	1.039	2	0.594
Breastfed as a baby || id:UKB-a:33	Maximum likelihood	3.442	1	0.064
Breastfed as a baby || id:UKB-a:33	Inverse variance weighted	0	1	1
Bring up phlegm/sputum/mucus on most days || id:UKB-a:259	Maximum likelihood	0.067	2	0.967
Bring up phlegm/sputum/mucus on most days || id:UKB-a:259	MR Egger	0.044	1	0.835
Bring up phlegm/sputum/mucus on most days || id:UKB-a:259	Inverse variance weighted	0.034	2	0.983
Butyrylcarnitine || id:476	Maximum likelihood	2.942	5	0.709
Butyrylcarnitine || id:476	MR Egger	2.330	4	0.675
Butyrylcarnitine || id:476	Inverse variance weighted	2.354	5	0.798
C-reactive protein || id:1015	Maximum likelihood	4.166	2	0.125
C-reactive protein || id:1015	MR Egger	0.000	1	1.000
C-reactive protein || id:1015	Inverse variance weighted	2.084	2	0.353
Cancer code self-reported: basal cell carcinoma || id:UKB-a:59	Maximum likelihood	7.707	13	0.862
Cancer code self-reported: basal cell carcinoma || id:UKB-a:59	MR Egger	7.595	12	0.816
Cancer code self-reported: basal cell carcinoma || id:UKB-a:59	Inverse variance weighted	7.117	13	0.896
Cancer code self-reported: breast cancer || id:UKB-a:55	Maximum likelihood	17.285	16	0.367
Cancer code self-reported: breast cancer || id:UKB-a:55	MR Egger	17.266	15	0.303
Cancer code self-reported: breast cancer || id:UKB-a:55	Inverse variance weighted	16.212	16	0.438
Cancer code self-reported: lung cancer || id:UKB-a:54	Maximum likelihood	3.455	3	0.327
Cancer code self-reported: lung cancer || id:UKB-a:54	MR Egger	0.505	2	0.777
Cancer code self-reported: lung cancer || id:UKB-a:54	Inverse variance weighted	2.310	3	0.511
Cancer code self-reported: malignant melanoma || id:UKB-a:58	Maximum likelihood	3.921	3	0.270
Cancer code self-reported: malignant melanoma || id:UKB-a:58	MR Egger	2.591	2	0.274
Cancer code self-reported: malignant melanoma || id:UKB-a:58	Inverse variance weighted	2.614	3	0.455
Cancer code self-reported: prostate cancer || id:UKB-a:57	Maximum likelihood	8.934	12	0.709
Cancer code self-reported: prostate cancer || id:UKB-a:57	MR Egger	8.653	11	0.654
Cancer code self-reported: prostate cancer || id:UKB-a:57	Inverse variance weighted	8.193	12	0.770
Cancer code self-reported: small intestine/small bowel cancer || id:UKB-a:56	Maximum likelihood	3.600	3	0.308

Cancer code self-reported: small intestine/small bowel cancer || id:UKB-a:56	MR Egger	3.493	2	0.174
Cancer code self-reported: small intestine/small bowel cancer || id:UKB-a:56	Inverse variance weighted	2.424	3	0.489
Cancer code self-reported: squamous cell carcinoma || id:UKB-a:60	Maximum likelihood	3.219	2	0.200
Cancer code self-reported: squamous cell carcinoma || id:UKB-a:60	MR Egger	2.696	1	0.101
Cancer code self-reported: squamous cell carcinoma || id:UKB-a:60	Inverse variance weighted	1.638	2	0.441
Cancer diagnosed by doctor || id:UKB-a:307	Maximum likelihood	2.407	4	0.661
Cancer diagnosed by doctor || id:UKB-a:307	MR Egger	2.275	3	0.517
Cancer diagnosed by doctor || id:UKB-a:307	Inverse variance weighted	1.814	4	0.770
Caprylate (8:0) || id:481	Maximum likelihood	0.058	1	0.809
Caprylate (8:0) || id:481	Inverse variance weighted	0	1	1
Cardioembolic stroke || id:1109	Maximum likelihood	0.431	1	0.512
Cardioembolic stroke || id:1109	Inverse variance weighted	0	1	1
Carnitine || id:379	Maximum likelihood	18.661	15	0.230
Carnitine || id:379	MR Egger	18.276	14	0.195
Carnitine || id:379	Inverse variance weighted	17.424	15	0.294
CD123 on 11c+123+DC || id:159	Maximum likelihood	3.302	5	0.654
CD123 on 11c+123+DC || id:159	MR Egger	3.074	4	0.546
CD123 on 11c+123+DC || id:159	Inverse variance weighted	2.643	5	0.755
CD32 on 11c+123+DC || id:162	Maximum likelihood	0.515	1	0.473
CD32 on 11c+123+DC || id:162	Inverse variance weighted	0	1	1
CD34 on Stem || id:151	Maximum likelihood	2.043	1	0.153
CD34 on Stem || id:151	Inverse variance weighted	0	1	1
Celiac disease || id:1058	Maximum likelihood	18.665	12	0.097
Celiac disease || id:1058	MR Egger	18.317	11	0.075
Celiac disease || id:1058	Inverse variance weighted	17.109	12	0.146
Celiac disease || id:1059	Maximum likelihood	27.145	10	0.002
Celiac disease || id:1059	MR Egger	27.018	9	0.001
Celiac disease || id:1059	Inverse variance weighted	24.434	10	0.007
Celiac disease || id:1060	Maximum likelihood	12.090	6	0.060
Celiac disease || id:1060	MR Egger	4.493	5	0.481
Celiac disease || id:1060	Inverse variance weighted	10.101	6	0.120
Celiac disease || id:276	Maximum likelihood	27.506	11	0.004
Celiac disease || id:276	MR Egger	27.364	10	0.002
Celiac disease || id:276	Inverse variance weighted	25.015	11	0.009
Childhood obesity || id:1096	Maximum likelihood	11.708	4	0.020
Childhood obesity || id:1096	MR Egger	4.093	3	0.252
Childhood obesity || id:1096	Inverse variance weighted	8.812	4	0.066
Cholesterol || id:307	Maximum likelihood	0.756	1	0.385
Cholesterol || id:307	Inverse variance weighted	0	1	1
Cholesterol esters in large HDL || id:875	Maximum likelihood	18.179	13	0.151
Cholesterol esters in large HDL || id:875	MR Egger	15.018	12	0.240
Cholesterol esters in large HDL || id:875	Inverse variance weighted	16.813	13	0.208

Cholesterol esters in large VLDL || id:881	Maximum likelihood	22.797	20	0.299
Cholesterol esters in large VLDL || id:881	MR Egger	18.107	19	0.515
Cholesterol esters in large VLDL || id:881	Inverse variance weighted	21.705	20	0.357
Cholesterol esters in large VLDL || id:887	Maximum likelihood	7.045	9	0.632
Cholesterol esters in large VLDL || id:887	MR Egger	7.061	8	0.530
Cholesterol esters in large VLDL || id:887	Inverse variance weighted	6.301	9	0.709
Cholesterol esters in medium HDL || id:899	Maximum likelihood	8.069	6	0.233
Cholesterol esters in medium HDL || id:899	MR Egger	3.856	5	0.570
Cholesterol esters in medium HDL || id:899	Inverse variance weighted	6.824	6	0.337
Chronic kidney disease || id:1102	Maximum likelihood	2.303	3	0.512
Chronic kidney disease || id:1102	MR Egger	0.298	2	0.862
Chronic kidney disease || id:1102	Inverse variance weighted	1.541	3	0.673
Chronotype || id:1087	Maximum likelihood	3.423	8	0.905
Chronotype || id:1087	MR Egger	3.371	7	0.849
Chronotype || id:1087	Inverse variance weighted	3.015	8	0.933
cis-trans-18:2 || id:1150	Maximum likelihood	3.430	4	0.489
cis-trans-18:2 || id:1150	MR Egger	3.476	3	0.324
cis-trans-18:2 || id:1150	Inverse variance weighted	2.611	4	0.625
Citrate || id:341	Maximum likelihood	5.835	5	0.323
Citrate || id:341	MR Egger	5.795	4	0.215
Citrate || id:341	Inverse variance weighted	4.669	5	0.458
Citrate || id:849	Maximum likelihood	5.795	4	0.215
Citrate || id:849	MR Egger	5.001	3	0.172
Citrate || id:849	Inverse variance weighted	4.347	4	0.361
Citrulline || id:356	Maximum likelihood	2.444	3	0.485
Citrulline || id:356	MR Egger	1.801	2	0.406
Citrulline || id:356	Inverse variance weighted	1.633	3	0.652
College completion || id:836	Maximum likelihood	0.343	2	0.842
College completion || id:836	MR Egger	0.330	1	0.565
College completion || id:836	Inverse variance weighted	0.179	2	0.914
Comparative body size at age 10 || id:UKB-a:34	Maximum likelihood	215.896	154	0.001
Comparative body size at age 10 || id:UKB-a:34	MR Egger	215.758	153	0.001
Comparative body size at age 10 || id:UKB-a:34	Inverse variance weighted	214.525	154	0.001
Concentration of IDL particles || id:870	Maximum likelihood	18.826	17	0.339
Concentration of IDL particles || id:870	MR Egger	15.558	16	0.484
Concentration of IDL particles || id:870	Inverse variance weighted	17.754	17	0.404
Concentration of large HDL particles || id:878	Maximum likelihood	15.215	11	0.173
Concentration of large HDL particles || id:878	MR Egger	13.117	10	0.217
Concentration of large HDL particles || id:878	Inverse variance weighted	13.846	11	0.242
Concentration of large LDL particles || id:884	Maximum likelihood	22.253	20	0.327
Concentration of large LDL particles || id:884	MR Egger	18.463	19	0.492
Concentration of large LDL particles || id:884	Inverse variance weighted	21.184	20	0.386

Concentration of large VLDL particles || id:890	Maximum likelihood	7.180	7	0.410
Concentration of large VLDL particles || id:890	MR Egger	7.180	6	0.305
Concentration of large VLDL particles || id:890	Inverse variance weighted	6.189	7	0.518
Copper || id:1073	Maximum likelihood	0.004	1	0.947
Copper || id:1073	Inverse variance weighted	0	1	1
Coronary heart disease || id:6	Maximum likelihood	1.248	2	0.536
Coronary heart disease || id:6	MR Egger	1.245	1	0.265
Coronary heart disease || id:6	Inverse variance weighted	0.629	2	0.730
Coronary heart disease || id:7	Maximum likelihood	39.665	36	0.310
Coronary heart disease || id:7	MR Egger	37.262	35	0.365
Coronary heart disease || id:7	Inverse variance weighted	38.622	36	0.352
Coronary heart disease || id:8	Maximum likelihood	11.063	14	0.681
Coronary heart disease || id:8	MR Egger	8.908	13	0.780
Coronary heart disease || id:8	Inverse variance weighted	10.288	14	0.741
Coronary heart disease || id:9	Maximum likelihood	11.755	9	0.227
Coronary heart disease || id:9	MR Egger	10.026	8	0.263
Coronary heart disease || id:9	Inverse variance weighted	10.470	9	0.314
Creatinine (enzymatic) in urine || id:UKB-a:333	Maximum likelihood	30.350	20	0.064
Creatinine (enzymatic) in urine || id:UKB-a:333	MR Egger	28.169	19	0.080
Creatinine (enzymatic) in urine || id:UKB-a:333	Inverse variance weighted	28.868	20	0.090
Creatinine || id:850	Maximum likelihood	1.286	5	0.936
Creatinine || id:850	MR Egger	1.231	4	0.873
Creatinine || id:850	Inverse variance weighted	1.029	5	0.960
Crohn's disease || id:10	Maximum likelihood	129.125	102	0.036
Crohn's disease || id:10	MR Egger	129.160	101	0.031
Crohn's disease || id:10	Inverse variance weighted	127.896	102	0.042
Crohn's disease || id:11	Maximum likelihood	20.422	12	0.060
Crohn's disease || id:11	MR Egger	19.030	11	0.061
Crohn's disease || id:11	Inverse variance weighted	18.740	12	0.095
Crohn's disease || id:12	Maximum likelihood	165.660	116	0.002
Crohn's disease || id:12	MR Egger	164.629	115	0.002
Crohn's disease || id:12	Inverse variance weighted	164.238	116	0.002
Crohn's disease || id:30	Maximum likelihood	57.017	46	0.128
Crohn's disease || id:30	MR Egger	56.590	45	0.115
Crohn's disease || id:30	Inverse variance weighted	55.778	46	0.153
Current tobacco smoking || id:UKB-a:16	Maximum likelihood	22.851	15	0.087
Current tobacco smoking || id:UKB-a:16	MR Egger	18.278	14	0.194
Current tobacco smoking || id:UKB-a:16	Inverse variance weighted	21.380	15	0.125
Daytime dozing / sleeping (narcolepsy) || id:UKB-a:15	Maximum likelihood	27.551	18	0.069
Daytime dozing / sleeping (narcolepsy) || id:UKB-a:15	MR Egger	26.809	17	0.061
Daytime dozing / sleeping (narcolepsy) || id:UKB-a:15	Inverse variance weighted	26.035	18	0.099
Decanoylcarnitine || id:618	Maximum likelihood	4.132	2	0.127

Decanoylcarnitine || id:618	MR Egger	3.424	1	0.064
Decanoylcarnitine || id:618	Inverse variance weighted	2.103	2	0.349
Dehydroisoandrosterone sulfate (DHEA-S) || id:478	Maximum likelihood	3.654	1	0.056
Dehydroisoandrosterone sulfate (DHEA-S) || id:478	Inverse variance weighted	0	1	1
Diabetes diagnosed by doctor || id:UKB-a:306	Maximum likelihood	40.824	48	0.759
Diabetes diagnosed by doctor || id:UKB-a:306	MR Egger	40.620	47	0.733
Diabetes diagnosed by doctor || id:UKB-a:306	Inverse variance weighted	40.044	48	0.786
Diagnoses - main ICD10: B37 Candidiasis || id:UKB-a:517	Maximum likelihood	2.548	6	0.863
Diagnoses - main ICD10: B37 Candidiasis || id:UKB-a:517	MR Egger	2.507	5	0.775
Diagnoses - main ICD10: B37 Candidiasis || id:UKB-a:517	Inverse variance weighted	2.178	6	0.903
Diagnoses - main ICD10: C44 Other malignant neoplasms of skin || id:UKB-a:518	Maximum likelihood	13.086	19	0.834
Diagnoses - main ICD10: C44 Other malignant neoplasms of skin || id:UKB-a:518	MR Egger	13.086	18	0.786
Diagnoses - main ICD10: C44 Other malignant neoplasms of skin || id:UKB-a:518	Inverse variance weighted	12.398	19	0.868
Diagnoses - main ICD10: C50 Malignant neoplasm of breast || id:UKB-a:519	Maximum likelihood	4.033	6	0.672
Diagnoses - main ICD10: C50 Malignant neoplasm of breast || id:UKB-a:519	MR Egger	3.361	5	0.645
Diagnoses - main ICD10: C50 Malignant neoplasm of breast || id:UKB-a:519	Inverse variance weighted	3.364	6	0.762
Diagnoses - main ICD10: C61 Malignant neoplasm of prostate || id:UKB-a:520	Maximum likelihood	3.723	9	0.929
Diagnoses - main ICD10: C61 Malignant neoplasm of prostate || id:UKB-a:520	MR Egger	3.623	8	0.889
Diagnoses - main ICD10: C61 Malignant neoplasm of prostate || id:UKB-a:520	Inverse variance weighted	3.315	9	0.950
Diagnoses - main ICD10: D12 Benign neoplasm of colon rectum anus and anal canal || id:UKB-a:521	Maximum likelihood	0.878	2	0.645
Diagnoses - main ICD10: D12 Benign neoplasm of colon rectum anus and anal canal || id:UKB-a:521	MR Egger	0.814	1	0.367
Diagnoses - main ICD10: D12 Benign neoplasm of colon rectum anus and anal canal || id:UKB-a:521	Inverse variance weighted	0.439	2	0.803
Diagnoses - main ICD10: D25 Leiomyoma of uterus || id:UKB-a:522	Maximum likelihood	6.597	3	0.086
Diagnoses - main ICD10: D25 Leiomyoma of uterus || id:UKB-a:522	MR Egger	1.636	2	0.441
Diagnoses - main ICD10: D25 Leiomyoma of uterus || id:UKB-a:522	Inverse variance weighted	4.407	3	0.221
Diagnoses - main ICD10: E03 Other hypothyroidism || id:UKB-a:523	Maximum likelihood	1.202	6	0.977
Diagnoses - main ICD10: E03 Other hypothyroidism || id:UKB-a:523	MR Egger	1.057	5	0.958
Diagnoses - main ICD10: E03 Other hypothyroidism || id:UKB-a:523	Inverse variance weighted	1.004	6	0.985
Diagnoses - main ICD10: E04 Other non-toxic goitre || id:UKB-a:524	Maximum likelihood	2.016	4	0.733
Diagnoses - main ICD10: E04 Other non-toxic goitre || id:UKB-a:524	MR Egger	2.003	3	0.572
Diagnoses - main ICD10: E04 Other non-toxic goitre || id:UKB-a:524	Inverse variance weighted	1.512	4	0.824
Diagnoses - main ICD10: G56 Mononeuropathies of upper limb || id:UKB-a:528	Maximum likelihood	5.521	4	0.238
Diagnoses - main ICD10: G56 Mononeuropathies of upper limb || id:UKB-a:528	MR Egger	5.033	3	0.169
Diagnoses - main ICD10: G56 Mononeuropathies of upper limb || id:UKB-a:528	Inverse variance weighted	4.151	4	0.386
Diagnoses - main ICD10: H25 Senile cataract || id:UKB-a:529	Maximum likelihood	0.261	1	0.610
Diagnoses - main ICD10: H25 Senile cataract || id:UKB-a:529	Inverse variance weighted	0	1	1
Diagnoses - main ICD10: H26 Other cataract || id:UKB-a:530	Maximum likelihood	2.655	2	0.265
Diagnoses - main ICD10: H26 Other cataract || id:UKB-a:530	MR Egger	0.842	1	0.359
Diagnoses - main ICD10: H26 Other cataract || id:UKB-a:530	Inverse variance weighted	1.329	2	0.515
Diagnoses - main ICD10: I10 Essential (primary) hypertension || id:UKB-a:531	Maximum likelihood	0.695	1	0.405
Diagnoses - main ICD10: I10 Essential (primary) hypertension || id:UKB-a:531	Inverse variance weighted	0	1	1
Diagnoses - main ICD10: I20 Angina pectoris || id:UKB-a:532	Maximum likelihood	0.564	1	0.453

Diagnoses - main ICD10: I20 Angina pectoris || id:UKB-a:532	Inverse variance weighted	0	1	1
Diagnoses - main ICD10: I21 Acute myocardial infarction || id:UKB-a:533	Maximum likelihood	0.113	2	0.945
Diagnoses - main ICD10: I21 Acute myocardial infarction || id:UKB-a:533	MR Egger	0.043	1	0.836
Diagnoses - main ICD10: I21 Acute myocardial infarction || id:UKB-a:533	Inverse variance weighted	0.058	2	0.972
Diagnoses - main ICD10: I25 Chronic ischaemic heart disease || id:UKB-a:534	Maximum likelihood	11.305	14	0.662
Diagnoses - main ICD10: I25 Chronic ischaemic heart disease || id:UKB-a:534	MR Egger	9.254	13	0.754
Diagnoses - main ICD10: I25 Chronic ischaemic heart disease || id:UKB-a:534	Inverse variance weighted	10.549	14	0.721
Diagnoses - main ICD10: I30 Acute pericarditis || id:UKB-a:535	Maximum likelihood	1.518	6	0.958
Diagnoses - main ICD10: I30 Acute pericarditis || id:UKB-a:535	MR Egger	1.517	5	0.911
Diagnoses - main ICD10: I30 Acute pericarditis || id:UKB-a:535	Inverse variance weighted	1.272	6	0.973
Diagnoses - main ICD10: I48 Atrial fibrillation and flutter || id:UKB-a:536	Maximum likelihood	7.239	12	0.841
Diagnoses - main ICD10: I48 Atrial fibrillation and flutter || id:UKB-a:536	MR Egger	7.058	11	0.794
Diagnoses - main ICD10: I48 Atrial fibrillation and flutter || id:UKB-a:536	Inverse variance weighted	6.639	12	0.881
Diagnoses - main ICD10: I80 Phlebitis and thrombophlebitis || id:UKB-a:537	Maximum likelihood	5.759	5	0.330
Diagnoses - main ICD10: I80 Phlebitis and thrombophlebitis || id:UKB-a:537	MR Egger	2.269	4	0.686
Diagnoses - main ICD10: I80 Phlebitis and thrombophlebitis || id:UKB-a:537	Inverse variance weighted	4.615	5	0.465
Diagnoses - main ICD10: I83 Varicose veins of lower extremities || id:UKB-a:538	Maximum likelihood	16.638	14	0.276
Diagnoses - main ICD10: I83 Varicose veins of lower extremities || id:UKB-a:538	MR Egger	16.283	13	0.234
Diagnoses - main ICD10: I83 Varicose veins of lower extremities || id:UKB-a:538	Inverse variance weighted	15.457	14	0.348
Diagnoses - main ICD10: I84 Haemorrhoids || id:UKB-a:539	Maximum likelihood	0.226	1	0.635
Diagnoses - main ICD10: I84 Haemorrhoids || id:UKB-a:539	Inverse variance weighted	0	1	1
Diagnoses - main ICD10: J33 Nasal polyp || id:UKB-a:541	Maximum likelihood	6.497	6	0.370
Diagnoses - main ICD10: J33 Nasal polyp || id:UKB-a:541	MR Egger	6.331	5	0.275
Diagnoses - main ICD10: J33 Nasal polyp || id:UKB-a:541	Inverse variance weighted	5.421	6	0.491
Diagnoses - main ICD10: J44 Other chronic obstructive pulmonary disease || id:UKB-a:543	Maximum likelihood	2.805	1	0.094
Diagnoses - main ICD10: J44 Other chronic obstructive pulmonary disease || id:UKB-a:543	Inverse variance weighted	0	1	1
Diagnoses - main ICD10: K40 Inguinal hernia || id:UKB-a:549	Maximum likelihood	7.879	9	0.546
Diagnoses - main ICD10: K40 Inguinal hernia || id:UKB-a:549	MR Egger	7.575	8	0.476
Diagnoses - main ICD10: K40 Inguinal hernia || id:UKB-a:549	Inverse variance weighted	7.014	9	0.636
Diagnoses - main ICD10: K50 Crohn's disease [regional enteritis] || id:UKB-a:552	Maximum likelihood	0.694	2	0.707
Diagnoses - main ICD10: K50 Crohn's disease [regional enteritis] || id:UKB-a:552	MR Egger	0.455	1	0.500
Diagnoses - main ICD10: K50 Crohn's disease [regional enteritis] || id:UKB-a:552	Inverse variance weighted	0.357	2	0.836
Diagnoses - main ICD10: K51 Ulcerative colitis || id:UKB-a:553	Maximum likelihood	0.283	1	0.595
Diagnoses - main ICD10: K51 Ulcerative colitis || id:UKB-a:553	Inverse variance weighted	0	1	1
Diagnoses - main ICD10: K57 Diverticular disease of intestine || id:UKB-a:555	Maximum likelihood	1.485	1	0.223
Diagnoses - main ICD10: K57 Diverticular disease of intestine || id:UKB-a:555	Inverse variance weighted	0	1	1
Diagnoses - main ICD10: K76 Other diseases of liver || id:UKB-a:558	Maximum likelihood	2.656	3	0.448
Diagnoses - main ICD10: K76 Other diseases of liver || id:UKB-a:558	MR Egger	0.559	2	0.756
Diagnoses - main ICD10: K76 Other diseases of liver || id:UKB-a:558	Inverse variance weighted	1.796	3	0.616
Diagnoses - main ICD10: K80 Cholelithiasis || id:UKB-a:559	Maximum likelihood	9.987	10	0.442
Diagnoses - main ICD10: K80 Cholelithiasis || id:UKB-a:559	MR Egger	9.230	9	0.416
Diagnoses - main ICD10: K80 Cholelithiasis || id:UKB-a:559	Inverse variance weighted	8.991	10	0.533

Diagnoses - main ICD10: L03 Cellulitis || id:UKB-a:560	Maximum likelihood	0.600	2	0.741
Diagnoses - main ICD10: L03 Cellulitis || id:UKB-a:560	MR Egger	0.185	1	0.667
Diagnoses - main ICD10: L03 Cellulitis || id:UKB-a:560	Inverse variance weighted	0.300	2	0.861
Diagnoses - main ICD10: M10 Gout || id:UKB-a:561	Maximum likelihood	10.474	4	0.033
Diagnoses - main ICD10: M10 Gout || id:UKB-a:561	MR Egger	9.650	3	0.022
Diagnoses - main ICD10: M10 Gout || id:UKB-a:561	Inverse variance weighted	7.856	4	0.097
Diagnoses - main ICD10: M17 Gonarthrosis [arthrosis of knee] || id:UKB-a:563	Maximum likelihood	0.644	1	0.422
Diagnoses - main ICD10: M17 Gonarthrosis [arthrosis of knee] || id:UKB-a:563	Inverse variance weighted	0	1	1
Diagnoses - main ICD10: M21 Other acquired deformities of limbs || id:UKB-a:565	Maximum likelihood	2.641	5	0.755
Diagnoses - main ICD10: M21 Other acquired deformities of limbs || id:UKB-a:565	MR Egger	2.643	4	0.619
Diagnoses - main ICD10: M21 Other acquired deformities of limbs || id:UKB-a:565	Inverse variance weighted	2.114	5	0.833
Diagnoses - main ICD10: M72 Fibroblastic disorders || id:UKB-a:572	Maximum likelihood	14.558	18	0.692
Diagnoses - main ICD10: M72 Fibroblastic disorders || id:UKB-a:572	MR Egger	14.452	17	0.635
Diagnoses - main ICD10: M72 Fibroblastic disorders || id:UKB-a:572	Inverse variance weighted	13.770	18	0.744
Diagnoses - main ICD10: N19 Unspecified renal failure || id:UKB-a:573	Maximum likelihood	5.180	6	0.521
Diagnoses - main ICD10: N19 Unspecified renal failure || id:UKB-a:573	MR Egger	5.206	5	0.391
Diagnoses - main ICD10: N19 Unspecified renal failure || id:UKB-a:573	Inverse variance weighted	4.342	6	0.630
Diagnoses - main ICD10: N20 Calculus of kidney and ureter || id:UKB-a:574	Maximum likelihood	1.377	2	0.502
Diagnoses - main ICD10: N20 Calculus of kidney and ureter || id:UKB-a:574	MR Egger	1.395	1	0.238
Diagnoses - main ICD10: N20 Calculus of kidney and ureter || id:UKB-a:574	Inverse variance weighted	0.700	2	0.705
Diagnoses - main ICD10: N40 Hyperplasia of prostate || id:UKB-a:576	Maximum likelihood	9.332	6	0.156
Diagnoses - main ICD10: N40 Hyperplasia of prostate || id:UKB-a:576	MR Egger	9.334	5	0.096
Diagnoses - main ICD10: N40 Hyperplasia of prostate || id:UKB-a:576	Inverse variance weighted	7.796	6	0.253
Diagnoses - main ICD10: N81 Female genital prolapse || id:UKB-a:577	Maximum likelihood	0.546	2	0.761
Diagnoses - main ICD10: N81 Female genital prolapse || id:UKB-a:577	MR Egger	0.022	1	0.883
Diagnoses - main ICD10: N81 Female genital prolapse || id:UKB-a:577	Inverse variance weighted	0.273	2	0.872
Diagnoses - main ICD10: O75 Other complications of labour and delivery not elsewhere classified || id:UKB-a:579	Maximum likelihood	8.931	7	0.258
Diagnoses - main ICD10: O75 Other complications of labour and delivery not elsewhere classified || id:UKB-a:579	MR Egger	8.744	6	0.189
Diagnoses - main ICD10: O75 Other complications of labour and delivery not elsewhere classified || id:UKB-a:579	Inverse variance weighted	7.673	7	0.362
Diagnoses - main ICD10: R31 Unspecified haematuria || id:UKB-a:585	Maximum likelihood	3.410	2	0.182
Diagnoses - main ICD10: R31 Unspecified haematuria || id:UKB-a:585	MR Egger	2.908	1	0.088
Diagnoses - main ICD10: R31 Unspecified haematuria || id:UKB-a:585	Inverse variance weighted	1.720	2	0.423
Diastolic blood pressure automated reading || id:UKB-a:359	Maximum likelihood	185.820	175	0.273
Diastolic blood pressure automated reading || id:UKB-a:359	MR Egger	185.826	174	0.256
Diastolic blood pressure automated reading || id:UKB-a:359	Inverse variance weighted	184.766	175	0.292
Difficulty not smoking for 1 day || id:UKB-a:344	Maximum likelihood	1.530	3	0.675
Difficulty not smoking for 1 day || id:UKB-a:344	MR Egger	1.346	2	0.510
Difficulty not smoking for 1 day || id:UKB-a:344	Inverse variance weighted	1.026	3	0.795
Dihomo-gamma-linolenic acid (20:3n6) || id:1140	Maximum likelihood	121.963	149	0.949
Dihomo-gamma-linolenic acid (20:3n6) || id:1140	MR Egger	117.828	148	0.968
Dihomo-gamma-linolenic acid (20:3n6) || id:1140	Inverse variance weighted	121.236	149	0.954
Dihomo-linolenate (20:3n3 or n6) || id:712	Maximum likelihood	0.189	1	0.664

Dihomo-linolenate (20:3n3 or n6) || id:712	Inverse variance weighted	0	1	1
Docosapentaenoic acid (22:5n3) || id:1144	Maximum likelihood	0.868	2	0.648
Docosapentaenoic acid (22:5n3) || id:1144	MR Egger	0.550	1	0.458
Docosapentaenoic acid (22:5n3) || id:1144	Inverse variance weighted	0.434	2	0.805
Doctor diagnosed asthma || id:UKB-a:255	Maximum likelihood	13.154	19	0.831
Doctor diagnosed asthma || id:UKB-a:255	MR Egger	13.052	18	0.788
Doctor diagnosed asthma || id:UKB-a:255	Inverse variance weighted	12.465	19	0.865
Doctor diagnosed hayfever or allergic rhinitis || id:UKB-a:254	Maximum likelihood	33.103	17	0.011
Doctor diagnosed hayfever or allergic rhinitis || id:UKB-a:254	MR Egger	31.508	16	0.012
Doctor diagnosed hayfever or allergic rhinitis || id:UKB-a:254	Inverse variance weighted	31.159	17	0.019
Drive faster than motorway speed limit || id:UKB-a:8	Maximum likelihood	14.217	11	0.221
Drive faster than motorway speed limit || id:UKB-a:8	MR Egger	13.700	10	0.187
Drive faster than motorway speed limit || id:UKB-a:8	Inverse variance weighted	13.066	11	0.289
Duration of vigorous activity || id:UKB-a:512	Maximum likelihood	0.026	1	0.873
Duration of vigorous activity || id:UKB-a:512	Inverse variance weighted	0	1	1
Duration of walks || id:UKB-a:507	Maximum likelihood	8.396	3	0.039
Duration of walks || id:UKB-a:507	MR Egger	7.306	2	0.026
Duration of walks || id:UKB-a:507	Inverse variance weighted	5.744	3	0.125
Eicosapentaenoic acid (20:5n3) || id:1147	Maximum likelihood	4.328	3	0.228
Eicosapentaenoic acid (20:5n3) || id:1147	MR Egger	4.397	2	0.111
Eicosapentaenoic acid (20:5n3) || id:1147	Inverse variance weighted	2.955	3	0.399
Epiandrosterone sulfate || id:627	Maximum likelihood	8.478	1	0.004
Epiandrosterone sulfate || id:627	Inverse variance weighted	0	1	1
Erythronate* || id:578	Maximum likelihood	0.796	2	0.672
Erythronate* || id:578	MR Egger	0.726	1	0.394
Erythronate* || id:578	Inverse variance weighted	0.398	2	0.819
Ever depressed for a whole week || id:UKB-a:373	Maximum likelihood	3.764	3	0.288
Ever depressed for a whole week || id:UKB-a:373	MR Egger	3.528	2	0.171
Ever depressed for a whole week || id:UKB-a:373	Inverse variance weighted	2.510	3	0.473
Ever smoked || id:UKB-a:236	Maximum likelihood	33.749	36	0.576
Ever smoked || id:UKB-a:236	MR Egger	33.146	35	0.558
Ever smoked || id:UKB-a:236	Inverse variance weighted	32.828	36	0.620
Ever used hormone-replacement therapy (HRT) || id:UKB-a:324	Maximum likelihood	1.435	4	0.838
Ever used hormone-replacement therapy (HRT) || id:UKB-a:324	MR Egger	0.894	3	0.827
Ever used hormone-replacement therapy (HRT) || id:UKB-a:324	Inverse variance weighted	1.079	4	0.898
Extreme body mass index || id:85	Maximum likelihood	12.419	6	0.053
Extreme body mass index || id:85	MR Egger	12.641	5	0.027
Extreme body mass index || id:85	Inverse variance weighted	10.555	6	0.103
Extreme height || id:86	Maximum likelihood	42.317	44	0.544
Extreme height || id:86	MR Egger	42.063	43	0.512
Extreme height || id:86	Inverse variance weighted	41.356	44	0.586
Extreme waist-to-hip ratio || id:87	Maximum likelihood	0	1	0.985

Extreme waist-to-hip ratio || id:87	Inverse variance weighted	0	1	1
Falls in the last year || id:UKB-a:262	Maximum likelihood	5.557	2	0.062
Falls in the last year || id:UKB-a:262	MR Egger	1.189	1	0.276
Falls in the last year || id:UKB-a:262	Inverse variance weighted	2.848	2	0.241
Fed-up feelings || id:UKB-a:49	Maximum likelihood	47.423	30	0.023
Fed-up feelings || id:UKB-a:49	MR Egger	47.152	29	0.018
Fed-up feelings || id:UKB-a:49	Inverse variance weighted	45.865	30	0.032
Ferritin || id:1050	Maximum likelihood	2.448	3	0.485
Ferritin || id:1050	MR Egger	0.301	2	0.860
Ferritin || id:1050	Inverse variance weighted	1.633	3	0.652
Fluid intelligence score || id:UKB-a:196	Maximum likelihood	71.177	41	0.002
Fluid intelligence score || id:UKB-a:196	MR Egger	71.234	40	0.002
Fluid intelligence score || id:UKB-a:196	Inverse variance weighted	69.497	41	0.004
Forced expiratory volume in 1-second (FEV1) Best measure || id:UKB-a:231	Maximum likelihood	188.333	140	0.004
Forced expiratory volume in 1-second (FEV1) Best measure || id:UKB-a:231	MR Egger	182.945	139	0.007
Forced expiratory volume in 1-second (FEV1) Best measure || id:UKB-a:231	Inverse variance weighted	187.019	140	0.005
Forced expiratory volume in 1-second (FEV1) predicted || id:UKB-a:234	Maximum likelihood	97.135	79	0.081
Forced expiratory volume in 1-second (FEV1) predicted || id:UKB-a:234	MR Egger	96.487	78	0.076
Forced expiratory volume in 1-second (FEV1) predicted || id:UKB-a:234	Inverse variance weighted	95.917	79	0.095
Forced expiratory volume in 1-second (FEV1) predicted percentage || id:UKB-a:235	Maximum likelihood	83.369	51	0.003
Forced expiratory volume in 1-second (FEV1) predicted percentage || id:UKB-a:235	MR Egger	83.161	50	0.002
Forced expiratory volume in 1-second (FEV1) predicted percentage || id:UKB-a:235	Inverse variance weighted	81.739	51	0.004
Forced expiratory volume in 1-second (FEV1) || id:UKB-a:337	Maximum likelihood	215.280	154	0.001
Forced expiratory volume in 1-second (FEV1) || id:UKB-a:337	MR Egger	205.996	153	0.003
Forced expiratory volume in 1-second (FEV1) || id:UKB-a:337	Inverse variance weighted	213.882	154	0.001
Forced vital capacity (FVC) Best measure || id:UKB-a:232	Maximum likelihood	248.613	189	0.002
Forced vital capacity (FVC) Best measure || id:UKB-a:232	MR Egger	246.869	188	0.003
Forced vital capacity (FVC) Best measure || id:UKB-a:232	Inverse variance weighted	247.385	189	0.003
Fractured/broken bones in last 5 years || id:UKB-a:308	Maximum likelihood	4.782	5	0.443
Fractured/broken bones in last 5 years || id:UKB-a:308	MR Egger	1.658	4	0.798
Fractured/broken bones in last 5 years || id:UKB-a:308	Inverse variance weighted	3.838	5	0.573
Free cholesterol || id:858	Maximum likelihood	6.719	11	0.821
Free cholesterol || id:858	MR Egger	4.333	10	0.931
Free cholesterol || id:858	Inverse variance weighted	6.117	11	0.865
Free cholesterol in IDL || id:868	Maximum likelihood	18.219	17	0.375
Free cholesterol in IDL || id:868	MR Egger	16.888	16	0.393
Free cholesterol in IDL || id:868	Inverse variance weighted	17.165	17	0.443
Free cholesterol in large HDL || id:876	Maximum likelihood	20.069	15	0.169
Free cholesterol in large HDL || id:876	MR Egger	16.646	14	0.276
Free cholesterol in large HDL || id:876	Inverse variance weighted	18.777	15	0.224
Free cholesterol in large LDL || id:882	Maximum likelihood	20.507	17	0.249
Free cholesterol in large LDL || id:882	MR Egger	16.455	16	0.422

Free cholesterol in large LDL || id:882	Inverse variance weighted	19.337	17	0.310
Free cholesterol in large VLDL || id:888	Maximum likelihood	6.694	9	0.669
Free cholesterol in large VLDL || id:888	MR Egger	6.421	8	0.600
Free cholesterol in large VLDL || id:888	Inverse variance weighted	5.981	9	0.742
Free cholesterol in medium HDL || id:900	Maximum likelihood	1.482	4	0.830
Free cholesterol in medium HDL || id:900	MR Egger	1.117	3	0.773
Free cholesterol in medium HDL || id:900	Inverse variance weighted	1.118	4	0.891
Free cholesterol to esterified cholesterol ratio || id:853	Maximum likelihood	6.123	12	0.910
Free cholesterol to esterified cholesterol ratio || id:853	MR Egger	5.443	11	0.908
Free cholesterol to esterified cholesterol ratio || id:853	Inverse variance weighted	5.620	12	0.934
Frequency of depressed mood in last 2 weeks || id:UKB-a:242	Maximum likelihood	10.307	6	0.112
Frequency of depressed mood in last 2 weeks || id:UKB-a:242	MR Egger	10.113	5	0.072
Frequency of depressed mood in last 2 weeks || id:UKB-a:242	Inverse variance weighted	8.590	6	0.198
Frequency of stair climbing in last 4 weeks || id:UKB-a:514	Maximum likelihood	11.730	4	0.019
Frequency of stair climbing in last 4 weeks || id:UKB-a:514	MR Egger	5.611	3	0.132
Frequency of stair climbing in last 4 weeks || id:UKB-a:514	Inverse variance weighted	9.067	4	0.059
Frequency of tenseness / restlessness in last 2 weeks || id:UKB-a:244	Maximum likelihood	5.867	6	0.438
Frequency of tenseness / restlessness in last 2 weeks || id:UKB-a:244	MR Egger	5.208	5	0.391
Frequency of tenseness / restlessness in last 2 weeks || id:UKB-a:244	Inverse variance weighted	4.905	6	0.556
Frequency of tiredness / lethargy in last 2 weeks || id:UKB-a:245	Maximum likelihood	21.012	19	0.336
Frequency of tiredness / lethargy in last 2 weeks || id:UKB-a:245	MR Egger	21.023	18	0.278
Frequency of tiredness / lethargy in last 2 weeks || id:UKB-a:245	Inverse variance weighted	19.917	19	0.400
Frequency of unenthusiasm / disinterest in last 2 weeks || id:UKB-a:243	Maximum likelihood	2.159	3	0.540
Frequency of unenthusiasm / disinterest in last 2 weeks || id:UKB-a:243	MR Egger	1.715	2	0.424
Frequency of unenthusiasm / disinterest in last 2 weeks || id:UKB-a:243	Inverse variance weighted	1.453	3	0.693
Frequency of walking for pleasure in last 4 weeks || id:UKB-a:515	Maximum likelihood	0.735	1	0.391
Frequency of walking for pleasure in last 4 weeks || id:UKB-a:515	Inverse variance weighted	0	1	1
Gamma-glutamylglutamine || id:359	Maximum likelihood	0.007	2	0.996
Gamma-glutamylglutamine || id:359	MR Egger	0.001	1	0.980
Gamma-glutamylglutamine || id:359	Inverse variance weighted	0.004	2	0.998
Gamma-glutamylphenylalanine || id:572	Maximum likelihood	0.185	1	0.667
Gamma-glutamylphenylalanine || id:572	Inverse variance weighted	0	1	1
Gamma-glutamyltyrosine || id:360	Maximum likelihood	1.854	4	0.763
Gamma-glutamyltyrosine || id:360	MR Egger	1.429	3	0.699
Gamma-glutamyltyrosine || id:360	Inverse variance weighted	1.415	4	0.842
Gamma-linolenic acid (18:3n6) || id:1139	Maximum likelihood	366.123	368	0.518
Gamma-linolenic acid (18:3n6) || id:1139	MR Egger	362.747	367	0.553
Gamma-linolenic acid (18:3n6) || id:1139	Inverse variance weighted	365.146	368	0.532
Getting up in morning || id:UKB-a:10	Maximum likelihood	34.936	36	0.519
Getting up in morning || id:UKB-a:10	MR Egger	33.278	35	0.551
Getting up in morning || id:UKB-a:10	Inverse variance weighted	33.998	36	0.564
Glucose || id:859	Maximum likelihood	3.634	3	0.304

Glucose || id:859	MR Egger	3.056	2	0.217
Glucose || id:859	Inverse variance weighted	2.431	3	0.488
Glutamine || id:860	Maximum likelihood	7.158	4	0.128
Glutamine || id:860	MR Egger	5.712	3	0.127
Glutamine || id:860	Inverse variance weighted	5.369	4	0.252
Glutaroyl carnitine || id:699	Maximum likelihood	2.987	7	0.886
Glutaroyl carnitine || id:699	MR Egger	2.953	6	0.815
Glutaroyl carnitine || id:699	Inverse variance weighted	2.562	7	0.922
Glycoprotein acetyls || id:863	Maximum likelihood	2.779	6	0.836
Glycoprotein acetyls || id:863	MR Egger	2.800	5	0.731
Glycoprotein acetyls || id:863	Inverse variance weighted	2.335	6	0.886
Glycoproteins || id:862	Maximum likelihood	6.336	9	0.706
Glycoproteins || id:862	MR Egger	6.323	8	0.611
Glycoproteins || id:862	Inverse variance weighted	5.632	9	0.776
Gout || id:1054	Maximum likelihood	8.381	1	0.004
Gout || id:1054	Inverse variance weighted	0	1	1
Guilty feelings || id:UKB-a:240	Maximum likelihood	29.531	15	0.014
Guilty feelings || id:UKB-a:240	MR Egger	28.990	14	0.010
Guilty feelings || id:UKB-a:240	Inverse variance weighted	27.611	15	0.024
Had menopause || id:UKB-a:316	Maximum likelihood	8.672	8	0.371
Had menopause || id:UKB-a:316	MR Egger	6.847	7	0.445
Had menopause || id:UKB-a:316	Inverse variance weighted	7.588	8	0.475
Haemoglobin concentration || id:270	Maximum likelihood	28.512	14	0.012
Haemoglobin concentration || id:270	MR Egger	25.008	13	0.023
Haemoglobin concentration || id:270	Inverse variance weighted	26.556	14	0.022
Hand grip strength (left) || id:UKB-a:374	Maximum likelihood	84.521	82	0.403
Hand grip strength (left) || id:UKB-a:374	MR Egger	84.091	81	0.385
Hand grip strength (left) || id:UKB-a:374	Inverse variance weighted	83.589	82	0.430
HDL cholesterol || id:299	Maximum likelihood	118.206	85	0.010
HDL cholesterol || id:299	MR Egger	108.772	84	0.036
HDL cholesterol || id:299	Inverse variance weighted	116.815	85	0.013
Headaches for 3+ months || id:UKB-a:354	Maximum likelihood	10.507	1	0.001
Headaches for 3+ months || id:UKB-a:354	Inverse variance weighted	0	1	1
Hearing difficulty/problems with background noise || id:UKB-a:261	Maximum likelihood	16.830	14	0.265
Hearing difficulty/problems with background noise || id:UKB-a:261	MR Egger	16.842	13	0.207
Hearing difficulty/problems with background noise || id:UKB-a:261	Inverse variance weighted	15.639	14	0.336
Hearing difficulty/problems: Yes || id:UKB-a:257	Maximum likelihood	18.173	22	0.696
Hearing difficulty/problems: Yes || id:UKB-a:257	MR Egger	15.897	21	0.775
Hearing difficulty/problems: Yes || id:UKB-a:257	Inverse variance weighted	17.355	22	0.743
Heart rate || id:1056	Maximum likelihood	6.853	14	0.940
Heart rate || id:1056	MR Egger	6.856	13	0.909
Heart rate || id:1056	Inverse variance weighted	6.366	14	0.956

Heel bone mineral density (BMD) T-score automated (left) || id:UKB-a:361	Maximum likelihood	155.417	135	0.110
Heel bone mineral density (BMD) T-score automated (left) || id:UKB-a:361	MR Egger	155.339	134	0.100
Heel bone mineral density (BMD) T-score automated (left) || id:UKB-a:361	Inverse variance weighted	154.300	135	0.122
Heel bone mineral density (BMD) T-score automated (right) || id:UKB-a:362	Maximum likelihood	155.026	136	0.126
Heel bone mineral density (BMD) T-score automated (right) || id:UKB-a:362	MR Egger	154.989	135	0.115
Heel bone mineral density (BMD) T-score automated (right) || id:UKB-a:362	Inverse variance weighted	153.915	136	0.140
Height || id:1034	Maximum likelihood	3.962	3	0.266
Height || id:1034	MR Egger	1.678	2	0.432
Height || id:1034	Inverse variance weighted	2.643	3	0.450
Heptanoate (7:0) || id:349	Maximum likelihood	0.057	1	0.811
Heptanoate (7:0) || id:349	Inverse variance weighted	0	1	1
Hexadecanedioate || id:711	Maximum likelihood	0.660	2	0.719
Hexadecanedioate || id:711	MR Egger	0.377	1	0.539
Hexadecanedioate || id:711	Inverse variance weighted	0.331	2	0.848
Hexanoylcarnitine || id:467	Maximum likelihood	8.986	3	0.029
Hexanoylcarnitine || id:467	MR Egger	7.628	2	0.022
Hexanoylcarnitine || id:467	Inverse variance weighted	6.020	3	0.111
High grade serous ovarian cancer || id:1121	Maximum likelihood	6.246	12	0.903
High grade serous ovarian cancer || id:1121	MR Egger	6.231	11	0.857
High grade serous ovarian cancer || id:1121	Inverse variance weighted	5.726	12	0.929
Hip circumference || id:100	Maximum likelihood	0.021	1	0.885
Hip circumference || id:100	Inverse variance weighted	0	1	1
Hip circumference || id:101	Maximum likelihood	4.127	4	0.389
Hip circumference || id:101	MR Egger	3.783	3	0.286
Hip circumference || id:101	Inverse variance weighted	3.134	4	0.536
Hip circumference || id:48	Maximum likelihood	49.968	47	0.356
Hip circumference || id:48	MR Egger	49.969	46	0.319
Hip circumference || id:48	Inverse variance weighted	48.907	47	0.396
Hip circumference || id:49	Maximum likelihood	53.441	51	0.381
Hip circumference || id:49	MR Egger	52.012	50	0.395
Hip circumference || id:49	Inverse variance weighted	52.399	51	0.419
Hip circumference || id:50	Maximum likelihood	27.024	23	0.255
Hip circumference || id:50	MR Egger	26.767	22	0.220
Hip circumference || id:50	Inverse variance weighted	25.852	23	0.308
Hip circumference || id:51	Maximum likelihood	21.685	16	0.154
Hip circumference || id:51	MR Egger	21.305	15	0.127
Hip circumference || id:51	Inverse variance weighted	20.334	16	0.206
Hip circumference || id:52	Maximum likelihood	20.951	18	0.282
Hip circumference || id:52	MR Egger	16.900	17	0.461
Hip circumference || id:52	Inverse variance weighted	19.792	18	0.345
Hip circumference || id:53	Maximum likelihood	14.922	14	0.384
Hip circumference || id:53	MR Egger	10.978	13	0.613

Hip circumference || id:53	Inverse variance weighted	13.859	14	0.460
Hip circumference || id:54	Maximum likelihood	79.801	73	0.274
Hip circumference || id:54	MR Egger	77.233	72	0.315
Hip circumference || id:54	Inverse variance weighted	78.708	73	0.303
Hip circumference || id:55	Maximum likelihood	72.094	72	0.475
Hip circumference || id:55	MR Egger	71.738	71	0.453
Hip circumference || id:55	Inverse variance weighted	71.101	72	0.508
Hip circumference || id:56	Maximum likelihood	35.290	39	0.640
Hip circumference || id:56	MR Egger	35.255	38	0.597
Hip circumference || id:56	Inverse variance weighted	34.402	39	0.679
Hip circumference || id:57	Maximum likelihood	35.436	37	0.542
Hip circumference || id:57	MR Egger	35.323	36	0.501
Hip circumference || id:57	Inverse variance weighted	34.507	37	0.586
Hip circumference || id:58	Maximum likelihood	25.050	29	0.676
Hip circumference || id:58	MR Egger	24.918	28	0.632
Hip circumference || id:58	Inverse variance weighted	24.192	29	0.719
Hip circumference || id:59	Maximum likelihood	24.267	29	0.716
Hip circumference || id:59	MR Egger	23.845	28	0.690
Hip circumference || id:59	Inverse variance weighted	23.437	29	0.756
Hippocampus volume || id:1045	Maximum likelihood	0.578	1	0.447
Hippocampus volume || id:1045	Inverse variance weighted	0	1	1
Histidine || id:866	Maximum likelihood	9.451	4	0.051
Histidine || id:866	MR Egger	7.888	3	0.048
Histidine || id:866	Inverse variance weighted	7.088	4	0.131
Home area population density - urban or rural: Scotland - Large Urban Area || id:UKB-a:228	Maximum likelihood	254.292	249	0.395
Home area population density - urban or rural: Scotland - Large Urban Area || id:UKB-a:228	MR Egger	248.648	248	0.476
Home area population density - urban or rural: Scotland - Large Urban Area || id:UKB-a:228	Inverse variance weighted	253.271	249	0.413
HWESASXX* || id:526	Maximum likelihood	1.021	1	0.312
HWESASXX* || id:526	Inverse variance weighted	0	1	1
Hydroxyisovaleroyl carnitine || id:698	Maximum likelihood	0.327	1	0.568
Hydroxyisovaleroyl carnitine || id:698	Inverse variance weighted	0	1	1
Illnesses of father: Chronic bronchitis/emphysema || id:UKB-a:206	Maximum likelihood	0.000	1	0.997
Illnesses of father: Chronic bronchitis/emphysema || id:UKB-a:206	Inverse variance weighted	0	1	1
Illnesses of father: Diabetes || id:UKB-a:208	Maximum likelihood	10.017	13	0.693
Illnesses of father: Diabetes || id:UKB-a:208	MR Egger	9.286	12	0.678
Illnesses of father: Diabetes || id:UKB-a:208	Inverse variance weighted	9.267	13	0.753
Illnesses of father: Heart disease || id:UKB-a:201	Maximum likelihood	27.154	17	0.056
Illnesses of father: Heart disease || id:UKB-a:201	MR Egger	23.234	16	0.108
Illnesses of father: Heart disease || id:UKB-a:201	Inverse variance weighted	25.577	17	0.083
Illnesses of father: High blood pressure || id:UKB-a:207	Maximum likelihood	2.421	5	0.788
Illnesses of father: High blood pressure || id:UKB-a:207	MR Egger	2.238	4	0.692
Illnesses of father: High blood pressure || id:UKB-a:207	Inverse variance weighted	1.945	5	0.857

Illnesses of father: Lung cancer || id:UKB-a:205	Maximum likelihood	1.975	2	0.372
Illnesses of father: Lung cancer || id:UKB-a:205	MR Egger	0.164	1	0.686
Illnesses of father: Lung cancer || id:UKB-a:205	Inverse variance weighted	0.989	2	0.610
Illnesses of father: None of the above (group 1) || id:UKB-a:202	Maximum likelihood	5.686	5	0.338
Illnesses of father: None of the above (group 1) || id:UKB-a:202	MR Egger	5.738	4	0.220
Illnesses of father: None of the above (group 1) || id:UKB-a:202	Inverse variance weighted	4.607	5	0.466
Illnesses of father: None of the above (group 2) || id:UKB-a:203	Maximum likelihood	2.121	1	0.145
Illnesses of father: None of the above (group 2) || id:UKB-a:203	Inverse variance weighted	0	1	1
Illnesses of father: Prostate cancer || id:UKB-a:204	Maximum likelihood	3.476	6	0.747
Illnesses of father: Prostate cancer || id:UKB-a:204	MR Egger	3.281	5	0.657
Illnesses of father: Prostate cancer || id:UKB-a:204	Inverse variance weighted	2.899	6	0.821
Illnesses of mother: Alzheimer's disease/dementia || id:UKB-a:210	Maximum likelihood	1.474	3	0.688
Illnesses of mother: Alzheimer's disease/dementia || id:UKB-a:210	MR Egger	0.279	2	0.870
Illnesses of mother: Alzheimer's disease/dementia || id:UKB-a:210	Inverse variance weighted	0.986	3	0.805
Illnesses of mother: Breast cancer || id:UKB-a:213	Maximum likelihood	11.751	10	0.302
Illnesses of mother: Breast cancer || id:UKB-a:213	MR Egger	11.665	9	0.233
Illnesses of mother: Breast cancer || id:UKB-a:213	Inverse variance weighted	10.576	10	0.391
Illnesses of mother: Chronic bronchitis/emphysema || id:UKB-a:214	Maximum likelihood	2.484	1	0.115
Illnesses of mother: Chronic bronchitis/emphysema || id:UKB-a:214	Inverse variance weighted	0	1	1
Illnesses of mother: Diabetes || id:UKB-a:216	Maximum likelihood	11.255	12	0.507
Illnesses of mother: Diabetes || id:UKB-a:216	MR Egger	10.611	11	0.476
Illnesses of mother: Diabetes || id:UKB-a:216	Inverse variance weighted	10.342	12	0.586
Illnesses of mother: Heart disease || id:UKB-a:209	Maximum likelihood	1.369	3	0.713
Illnesses of mother: Heart disease || id:UKB-a:209	MR Egger	0.868	2	0.648
Illnesses of mother: Heart disease || id:UKB-a:209	Inverse variance weighted	0.915	3	0.822
Illnesses of mother: High blood pressure || id:UKB-a:215	Maximum likelihood	3.199	8	0.921
Illnesses of mother: High blood pressure || id:UKB-a:215	MR Egger	1.952	7	0.962
Illnesses of mother: High blood pressure || id:UKB-a:215	Inverse variance weighted	2.815	8	0.945
Illnesses of mother: None of the above (group 1) || id:UKB-a:211	Maximum likelihood	8.110	3	0.044
Illnesses of mother: None of the above (group 1) || id:UKB-a:211	MR Egger	7.862	2	0.020
Illnesses of mother: None of the above (group 1) || id:UKB-a:211	Inverse variance weighted	5.428	3	0.143
Illnesses of siblings: Diabetes || id:UKB-a:223	Maximum likelihood	2.770	4	0.597
Illnesses of siblings: Diabetes || id:UKB-a:223	MR Egger	1.707	3	0.635
Illnesses of siblings: Diabetes || id:UKB-a:223	Inverse variance weighted	2.079	4	0.721
Illnesses of siblings: Heart disease || id:UKB-a:217	Maximum likelihood	0.266	2	0.875
Illnesses of siblings: Heart disease || id:UKB-a:217	MR Egger	0.010	1	0.922
Illnesses of siblings: Heart disease || id:UKB-a:217	Inverse variance weighted	0.135	2	0.935
Illnesses of siblings: High blood pressure || id:UKB-a:222	Maximum likelihood	22.699	11	0.019
Illnesses of siblings: High blood pressure || id:UKB-a:222	MR Egger	22.634	10	0.012
Illnesses of siblings: High blood pressure || id:UKB-a:222	Inverse variance weighted	20.639	11	0.037
Illnesses of siblings: None of the above (group 1) || id:UKB-a:218	Maximum likelihood	11.991	7	0.101
Illnesses of siblings: None of the above (group 1) || id:UKB-a:218	MR Egger	11.351	6	0.078

Illnesses of siblings: None of the above (group 1) || id:UKB-a:218	Inverse variance weighted	10.528	7	0.161
Immunoglobulin G index levels || id:1017	Maximum likelihood	0.409	1	0.523
Immunoglobulin G index levels || id:1017	Inverse variance weighted	0	1	1
Indoleacetate || id:446	Maximum likelihood	1.526	1	0.217
Indoleacetate || id:446	Inverse variance weighted	0	1	1
Inflammatory bowel disease || id:292	Maximum likelihood	104.026	111	0.668
Inflammatory bowel disease || id:292	MR Egger	103.966	110	0.644
Inflammatory bowel disease || id:292	Inverse variance weighted	103.110	111	0.691
Inflammatory bowel disease || id:293	Maximum likelihood	9.671	7	0.208
Inflammatory bowel disease || id:293	MR Egger	8.987	6	0.174
Inflammatory bowel disease || id:293	Inverse variance weighted	8.292	7	0.308
Inflammatory bowel disease || id:294	Maximum likelihood	168.406	128	0.010
Inflammatory bowel disease || id:294	MR Egger	168.131	127	0.009
Inflammatory bowel disease || id:294	Inverse variance weighted	167.094	128	0.012
Inflammatory bowel disease || id:31	Maximum likelihood	65.993	57	0.194
Inflammatory bowel disease || id:31	MR Egger	65.328	56	0.184
Inflammatory bowel disease || id:31	Inverse variance weighted	64.850	57	0.222
Invasive mucinous ovarian cancer || id:1123	Maximum likelihood	0.682	1	0.409
Invasive mucinous ovarian cancer || id:1123	Inverse variance weighted	0	1	1
Iron || id:1049	Maximum likelihood	3.678	2	0.159
Iron || id:1049	MR Egger	3.127	1	0.077
Iron || id:1049	Inverse variance weighted	1.839	2	0.399
Irritability || id:UKB-a:47	Maximum likelihood	49.727	25	0.002
Irritability || id:UKB-a:47	MR Egger	49.817	24	0.001
Irritability || id:UKB-a:47	Inverse variance weighted	47.832	25	0.004
Isobutyrylcarnitine || id:573	Maximum likelihood	2.094	2	0.351
Isobutyrylcarnitine || id:573	MR Egger	0.044	1	0.835
Isobutyrylcarnitine || id:573	Inverse variance weighted	1.055	2	0.590
Isovalerylcarnitine || id:652	Maximum likelihood	0.883	2	0.643
Isovalerylcarnitine || id:652	MR Egger	0.223	1	0.637
Isovalerylcarnitine || id:652	Inverse variance weighted	0.442	2	0.802
Job involves heavy manual or physical work || id:UKB-a:503	Maximum likelihood	27.021	15	0.029
Job involves heavy manual or physical work || id:UKB-a:503	MR Egger	20.520	14	0.115
Job involves heavy manual or physical work || id:UKB-a:503	Inverse variance weighted	25.424	15	0.045
Job involves mainly walking or standing || id:UKB-a:502	Maximum likelihood	8.340	6	0.214
Job involves mainly walking or standing || id:UKB-a:502	MR Egger	8.366	5	0.137
Job involves mainly walking or standing || id:UKB-a:502	Inverse variance weighted	7.004	6	0.320
Kynurenine || id:375	Maximum likelihood	13.871	3	0.003
Kynurenine || id:375	MR Egger	11.923	2	0.003
Kynurenine || id:375	Inverse variance weighted	9.248	3	0.026
Lactate || id:894	Maximum likelihood	2.109	1	0.146
Lactate || id:894	Inverse variance weighted	0	1	1

Laurate (12:0) || id:350	Maximum likelihood	0.029	1	0.865
Laurate (12:0) || id:350	Inverse variance weighted	0	1	1
LDL cholesterol || id:300	Maximum likelihood	112.714	77	0.005
LDL cholesterol || id:300	MR Egger	112.823	76	0.004
LDL cholesterol || id:300	Inverse variance weighted	111.361	77	0.006
Leg fat percentage (left) || id:UKB-a:278	Maximum likelihood	309.770	246	0.004
Leg fat percentage (left) || id:UKB-a:278	MR Egger	301.087	245	0.008
Leg fat percentage (left) || id:UKB-a:278	Inverse variance weighted	308.511	246	0.004
Leg fat percentage (right) || id:UKB-a:274	Maximum likelihood	311.275	244	0.002
Leg fat percentage (right) || id:UKB-a:274	MR Egger	304.776	243	0.004
Leg fat percentage (right) || id:UKB-a:274	Inverse variance weighted	309.999	244	0.003
Length of menstrual cycle || id:UKB-a:351	Maximum likelihood	5.870	5	0.319
Length of menstrual cycle || id:UKB-a:351	MR Egger	5.650	4	0.227
Length of menstrual cycle || id:UKB-a:351	Inverse variance weighted	4.714	5	0.452
Leucine || id:306	Maximum likelihood	9.129	11	0.610
Leucine || id:306	MR Egger	9.110	10	0.522
Leucine || id:306	Inverse variance weighted	8.304	11	0.686
Leucine || id:897	Maximum likelihood	0.906	2	0.636
Leucine || id:897	MR Egger	0.905	1	0.341
Leucine || id:897	Inverse variance weighted	0.453	2	0.797
Levulinate (4-oxovalerate) || id:433	Maximum likelihood	0.152	1	0.697
Levulinate (4-oxovalerate) || id:433	Inverse variance weighted	0	1	1
Light smokers at least 100 smokes in lifetime || id:UKB-a:313	Maximum likelihood	3.333	3	0.343
Light smokers at least 100 smokes in lifetime || id:UKB-a:313	MR Egger	1.654	2	0.437
Light smokers at least 100 smokes in lifetime || id:UKB-a:313	Inverse variance weighted	2.222	3	0.528
Linoleic acid (18:2n6) || id:1142	Maximum likelihood	117.762	99	0.096
Linoleic acid (18:2n6) || id:1142	MR Egger	116.627	98	0.097
Linoleic acid (18:2n6) || id:1142	Inverse variance weighted	116.597	99	0.109
Loneliness isolation || id:UKB-a:239	Maximum likelihood	2.327	4	0.676
Loneliness isolation || id:UKB-a:239	MR Egger	2.177	3	0.537
Loneliness isolation || id:UKB-a:239	Inverse variance weighted	1.762	4	0.779
Long-standing illness disability or infirmity || id:UKB-a:252	Maximum likelihood	18.436	13	0.142
Long-standing illness disability or infirmity || id:UKB-a:252	MR Egger	11.302	12	0.503
Long-standing illness disability or infirmity || id:UKB-a:252	Inverse variance weighted	17.022	13	0.198
MDC:%32+ || id:266	Maximum likelihood	213.891	205	0.321
MDC:%32+ || id:266	MR Egger	213.106	204	0.317
MDC:%32+ || id:266	Inverse variance weighted	212.863	205	0.339
Mean cell haemoglobin || id:271	Maximum likelihood	26.073	29	0.622
Mean cell haemoglobin || id:271	MR Egger	24.670	28	0.646
Mean cell haemoglobin || id:271	Inverse variance weighted	25.177	29	0.669
Mean cell haemoglobin concentration || id:272	Maximum likelihood	9.000	12	0.703
Mean cell haemoglobin concentration || id:272	MR Egger	8.539	11	0.664

Mean cell haemoglobin concentration || id:272	Inverse variance weighted	8.258	12	0.765
Mean cell volume || id:273	Maximum likelihood	39.084	37	0.376
Mean cell volume || id:273	MR Egger	39.014	36	0.336
Mean cell volume || id:273	Inverse variance weighted	38.028	37	0.422
Mean diameter for HDL particles || id:865	Maximum likelihood	7.967	12	0.788
Mean diameter for HDL particles || id:865	MR Egger	7.645	11	0.745
Mean diameter for HDL particles || id:865	Inverse variance weighted	7.305	12	0.837
Mean diameter for LDL particles || id:896	Maximum likelihood	4.180	7	0.759
Mean diameter for LDL particles || id:896	MR Egger	3.639	6	0.725
Mean diameter for LDL particles || id:896	Inverse variance weighted	3.586	7	0.826
Mean platelet volume || id:1006	Maximum likelihood	13.804	22	0.908
Mean platelet volume || id:1006	MR Egger	13.730	21	0.881
Mean platelet volume || id:1006	Inverse variance weighted	13.177	22	0.928
Mean time to correctly identify matches || id:UKB-a:199	Maximum likelihood	53.003	28	0.003
Mean time to correctly identify matches || id:UKB-a:199	MR Egger	52.890	27	0.002
Mean time to correctly identify matches || id:UKB-a:199	Inverse variance weighted	51.119	28	0.005
Medication for cholesterol blood pressure or diabetes: Blood pressure medication || id:UKB-a:490	Maximum likelihood	68.140	47	0.024
Medication for cholesterol blood pressure or diabetes: Blood pressure medication || id:UKB-a:490	MR Egger	66.019	46	0.028
Medication for cholesterol blood pressure or diabetes: Blood pressure medication || id:UKB-a:490	Inverse variance weighted	66.692	47	0.031
Medication for cholesterol blood pressure or diabetes: Cholesterol lowering medication || id:UKB-a:488	Maximum likelihood	40.317	33	0.178
Medication for cholesterol blood pressure or diabetes: Cholesterol lowering medication || id:UKB-a:488	MR Egger	40.467	32	0.145
Medication for cholesterol blood pressure or diabetes: Cholesterol lowering medication || id:UKB-a:488	Inverse variance weighted	39.275	33	0.209
Medication for cholesterol blood pressure or diabetes: Insulin || id:UKB-a:491	Maximum likelihood	8.418	5	0.135
Medication for cholesterol blood pressure or diabetes: Insulin || id:UKB-a:491	MR Egger	3.731	4	0.444
Medication for cholesterol blood pressure or diabetes: Insulin || id:UKB-a:491	Inverse variance weighted	6.739	5	0.241
Medication for cholesterol blood pressure or diabetes: None of the above || id:UKB-a:489	Maximum likelihood	46.642	41	0.252
Medication for cholesterol blood pressure or diabetes: None of the above || id:UKB-a:489	MR Egger	44.725	40	0.280
Medication for cholesterol blood pressure or diabetes: None of the above || id:UKB-a:489	Inverse variance weighted	45.506	41	0.290
Mineral and other dietary supplements: Calcium || id:UKB-a:495	Maximum likelihood	0.001	1	0.978
Mineral and other dietary supplements: Calcium || id:UKB-a:495	Inverse variance weighted	0	1	1
Mineral and other dietary supplements: Fish oil (including cod liver oil) || id:UKB-a:492	Maximum likelihood	0.840	1	0.359
Mineral and other dietary supplements: Fish oil (including cod liver oil) || id:UKB-a:492	Inverse variance weighted	0	1	1
Mineral and other dietary supplements: Glucosamine || id:UKB-a:494	Maximum likelihood	0.366	1	0.545
Mineral and other dietary supplements: Glucosamine || id:UKB-a:494	Inverse variance weighted	0	1	1
Mineral and other dietary supplements: None of the above || id:UKB-a:493	Maximum likelihood	0.236	1	0.627
Mineral and other dietary supplements: None of the above || id:UKB-a:493	Inverse variance weighted	0	1	1
Morning/evening person (chronotype) || id:UKB-a:11	Maximum likelihood	105.925	81	0.033
Morning/evening person (chronotype) || id:UKB-a:11	MR Egger	104.866	80	0.033
Morning/evening person (chronotype) || id:UKB-a:11	Inverse variance weighted	104.638	81	0.040
Multiple sclerosis || id:1024	Maximum likelihood	28.229	22	0.168
Multiple sclerosis || id:1024	MR Egger	28.119	21	0.137
Multiple sclerosis || id:1024	Inverse variance weighted	26.947	22	0.213

Multiple sclerosis || id:1025	Maximum likelihood	66.233	45	0.021
Multiple sclerosis || id:1025	MR Egger	64.842	44	0.022
Multiple sclerosis || id:1025	Inverse variance weighted	64.812	45	0.028
Myo-inositol || id:417	Maximum likelihood	0.084	1	0.772
Myo-inositol || id:417	Inverse variance weighted	0	1	1
N-acetylglycine || id:449	Maximum likelihood	0.099	1	0.753
N-acetylglycine || id:449	Inverse variance weighted	0	1	1
N2-dimethylguanosine || id:679	Maximum likelihood	1.471	1	0.225
N2-dimethylguanosine || id:679	Inverse variance weighted	0	1	1
Nap during day || id:UKB-a:12	Maximum likelihood	65.834	47	0.036
Nap during day || id:UKB-a:12	MR Egger	65.135	46	0.033
Nap during day || id:UKB-a:12	Inverse variance weighted	64.434	47	0.046
Nervous feelings || id:UKB-a:50	Maximum likelihood	36.525	25	0.064
Nervous feelings || id:UKB-a:50	MR Egger	31.521	24	0.139
Nervous feelings || id:UKB-a:50	Inverse variance weighted	35.065	25	0.087
Neuroblastoma || id:816	Maximum likelihood	2.905	2	0.234
Neuroblastoma || id:816	MR Egger	0.345	1	0.557
Neuroblastoma || id:816	Inverse variance weighted	1.477	2	0.478
Neuroticism || id:1007	Maximum likelihood	10.150	8	0.255
Neuroticism || id:1007	MR Egger	10.260	7	0.174
Neuroticism || id:1007	Inverse variance weighted	9	8	0.342
Neuroticism score || id:UKB-a:230	Maximum likelihood	75.800	63	0.129
Neuroticism score || id:UKB-a:230	MR Egger	71.878	62	0.183
Neuroticism score || id:UKB-a:230	Inverse variance weighted	74.598	63	0.150
NKeff:%314-158a+ || id:241	Maximum likelihood	283.249	226	0.006
NKeff:%314-158a+ || id:241	MR Egger	282.712	225	0.005
NKeff:%314-158a+ || id:241	Inverse variance weighted	282.000	226	0.007
Non-cancer illness code self-reported: angina || id:UKB-a:62	Maximum likelihood	2.711	12	0.997
Non-cancer illness code self-reported: angina || id:UKB-a:62	MR Egger	2.282	11	0.997
Non-cancer illness code self-reported: angina || id:UKB-a:62	Inverse variance weighted	2.506	12	0.998
Non-cancer illness code self-reported: ankylosing spondylitis || id:UKB-a:88	Maximum likelihood	0.116	1	0.734
Non-cancer illness code self-reported: ankylosing spondylitis || id:UKB-a:88	Inverse variance weighted	0	1	1
Non-cancer illness code self-reported: asthma || id:UKB-a:66	Maximum likelihood	96.196	71	0.025
Non-cancer illness code self-reported: asthma || id:UKB-a:66	MR Egger	96.059	70	0.021
Non-cancer illness code self-reported: asthma || id:UKB-a:66	Inverse variance weighted	94.861	71	0.031
Non-cancer illness code self-reported: cholelithiasis/gall stones || id:UKB-a:71	Maximum likelihood	19.102	11	0.059
Non-cancer illness code self-reported: cholelithiasis/gall stones || id:UKB-a:71	MR Egger	18.603	10	0.046
Non-cancer illness code self-reported: cholelithiasis/gall stones || id:UKB-a:71	Inverse variance weighted	17.366	11	0.098
Non-cancer illness code self-reported: crohns disease || id:UKB-a:103	Maximum likelihood	4.287	5	0.509
Non-cancer illness code self-reported: crohns disease || id:UKB-a:103	MR Egger	3.362	4	0.499
Non-cancer illness code self-reported: crohns disease || id:UKB-a:103	Inverse variance weighted	3.472	5	0.628
Non-cancer illness code self-reported: deep venous thrombosis (dvt) || id:UKB-a:65	Maximum likelihood	13.398	8	0.099

Non-cancer illness code self-reported: deep venous thrombosis (dvt) || id:UKB-a:65	MR Egger	9.961	7	0.191
Non-cancer illness code self-reported: deep venous thrombosis (dvt) || id:UKB-a:65	Inverse variance weighted	11.729	8	0.164
Non-cancer illness code self-reported: diabetes || id:UKB-a:74	Maximum likelihood	35.692	35	0.436
Non-cancer illness code self-reported: diabetes || id:UKB-a:74	MR Egger	35.620	34	0.392
Non-cancer illness code self-reported: diabetes || id:UKB-a:74	Inverse variance weighted	34.712	35	0.482
Non-cancer illness code self-reported: diverticular disease/diverticulitis || id:UKB-a:102	Maximum likelihood	1.700	1	0.192
Non-cancer illness code self-reported: diverticular disease/diverticulitis || id:UKB-a:102	Inverse variance weighted	0	1	1
Non-cancer illness code self-reported: enlarged prostate || id:UKB-a:95	Maximum likelihood	2.493	6	0.869
Non-cancer illness code self-reported: enlarged prostate || id:UKB-a:95	MR Egger	2.347	5	0.799
Non-cancer illness code self-reported: enlarged prostate || id:UKB-a:95	Inverse variance weighted	2.080	6	0.912
Non-cancer illness code self-reported: glaucoma || id:UKB-a:79	Maximum likelihood	6.075	7	0.531
Non-cancer illness code self-reported: glaucoma || id:UKB-a:79	MR Egger	6.029	6	0.420
Non-cancer illness code self-reported: glaucoma || id:UKB-a:79	Inverse variance weighted	5.207	7	0.635
Non-cancer illness code self-reported: gout || id:UKB-a:107	Maximum likelihood	29.474	21	0.103
Non-cancer illness code self-reported: gout || id:UKB-a:107	MR Egger	27.896	20	0.112
Non-cancer illness code self-reported: gout || id:UKB-a:107	Inverse variance weighted	28.071	21	0.138
Non-cancer illness code self-reported: hayfever/allergic rhinitis || id:UKB-a:94	Maximum likelihood	24.296	19	0.185
Non-cancer illness code self-reported: hayfever/allergic rhinitis || id:UKB-a:94	MR Egger	24.297	18	0.146
Non-cancer illness code self-reported: hayfever/allergic rhinitis || id:UKB-a:94	Inverse variance weighted	23.018	19	0.237
Non-cancer illness code self-reported: heart attack/myocardial infarction || id:UKB-a:63	Maximum likelihood	2.535	9	0.980
Non-cancer illness code self-reported: heart attack/myocardial infarction || id:UKB-a:63	MR Egger	2.446	8	0.964
Non-cancer illness code self-reported: heart attack/myocardial infarction || id:UKB-a:63	Inverse variance weighted	2.279	9	0.986
Non-cancer illness code self-reported: high cholesterol || id:UKB-a:108	Maximum likelihood	41.973	48	0.717
Non-cancer illness code self-reported: high cholesterol || id:UKB-a:108	MR Egger	41.992	47	0.680
Non-cancer illness code self-reported: high cholesterol || id:UKB-a:108	Inverse variance weighted	41.196	48	0.746
Non-cancer illness code self-reported: hypertension || id:UKB-a:61	Maximum likelihood	149.660	153	0.561
Non-cancer illness code self-reported: hypertension || id:UKB-a:61	MR Egger	147.584	152	0.586
Non-cancer illness code self-reported: hypertension || id:UKB-a:61	Inverse variance weighted	148.686	153	0.583
Non-cancer illness code self-reported: hyperthyroidism/thyrotoxicosis || id:UKB-a:76	Maximum likelihood	9.559	5	0.089
Non-cancer illness code self-reported: hyperthyroidism/thyrotoxicosis || id:UKB-a:76	MR Egger	9.562	4	0.048
Non-cancer illness code self-reported: hyperthyroidism/thyrotoxicosis || id:UKB-a:76	Inverse variance weighted	7.650	5	0.177
Non-cancer illness code self-reported: hypertrophic cardiomyopathy (hcm / hocm) || id:UKB-a:114	Maximum likelihood	18.516	22	0.675
Non-cancer illness code self-reported: hypertrophic cardiomyopathy (hcm / hocm) || id:UKB-a:114	MR Egger	15.454	21	0.799
Non-cancer illness code self-reported: hypertrophic cardiomyopathy (hcm / hocm) || id:UKB-a:114	Inverse variance weighted	17.675	22	0.725
Non-cancer illness code self-reported: hypopituitarism || id:UKB-a:98	Maximum likelihood	13.048	8	0.110
Non-cancer illness code self-reported: hypopituitarism || id:UKB-a:98	MR Egger	12.689	7	0.080
Non-cancer illness code self-reported: hypopituitarism || id:UKB-a:98	Inverse variance weighted	11.463	8	0.177
Non-cancer illness code self-reported: hypothyroidism/myxoedema || id:UKB-a:77	Maximum likelihood	105.268	79	0.026
Non-cancer illness code self-reported: hypothyroidism/myxoedema || id:UKB-a:77	MR Egger	104.927	78	0.023
Non-cancer illness code self-reported: hypothyroidism/myxoedema || id:UKB-a:77	Inverse variance weighted	103.988	79	0.031
Non-cancer illness code self-reported: kidney stone/ureter stone/bladder stone || id:UKB-a:72	Maximum likelihood	0.890	2	0.641
Non-cancer illness code self-reported: kidney stone/ureter stone/bladder stone || id:UKB-a:72	MR Egger	0.879	1	0.348

Non-cancer illness code self-reported: kidney stone/ureter stone/bladder stone || id:UKB-a:72	Inverse variance weighted	0.447	2	0.800
Non-cancer illness code self-reported: malabsorption/coeliac disease || id:UKB-a:101	Maximum likelihood	16.445	12	0.172
Non-cancer illness code self-reported: malabsorption/coeliac disease || id:UKB-a:101	MR Egger	15.491	11	0.161
Non-cancer illness code self-reported: malabsorption/coeliac disease || id:UKB-a:101	Inverse variance weighted	15.076	12	0.237
Non-cancer illness code self-reported: migraine || id:UKB-a:78	Maximum likelihood	10.768	12	0.549
Non-cancer illness code self-reported: migraine || id:UKB-a:78	MR Egger	10.049	11	0.526
Non-cancer illness code self-reported: migraine || id:UKB-a:78	Inverse variance weighted	9.874	12	0.627
Non-cancer illness code self-reported: nasal polyps || id:UKB-a:97	Maximum likelihood	2.935	4	0.569
Non-cancer illness code self-reported: nasal polyps || id:UKB-a:97	MR Egger	1.784	3	0.619
Non-cancer illness code self-reported: nasal polyps || id:UKB-a:97	Inverse variance weighted	2.203	4	0.698
Non-cancer illness code self-reported: osteoarthritis || id:UKB-a:106	Maximum likelihood	4.546	1	0.033
Non-cancer illness code self-reported: osteoarthritis || id:UKB-a:106	Inverse variance weighted	0	1	1
Non-cancer illness code self-reported: osteoporosis || id:UKB-a:87	Maximum likelihood	10.661	7	0.154
Non-cancer illness code self-reported: osteoporosis || id:UKB-a:87	MR Egger	9.701	6	0.138
Non-cancer illness code self-reported: osteoporosis || id:UKB-a:87	Inverse variance weighted	9.138	7	0.243
Non-cancer illness code self-reported: pernicious anaemia || id:UKB-a:90	Maximum likelihood	1.682	2	0.431
Non-cancer illness code self-reported: pernicious anaemia || id:UKB-a:90	MR Egger	0.746	1	0.388
Non-cancer illness code self-reported: pernicious anaemia || id:UKB-a:90	Inverse variance weighted	0.842	2	0.656
Non-cancer illness code self-reported: pneumothorax || id:UKB-a:111	Maximum likelihood	1.983	4	0.739
Non-cancer illness code self-reported: pneumothorax || id:UKB-a:111	MR Egger	1.766	3	0.622
Non-cancer illness code self-reported: pneumothorax || id:UKB-a:111	Inverse variance weighted	1.487	4	0.829
Non-cancer illness code self-reported: polio / poliomyelitis || id:UKB-a:112	Maximum likelihood	11.969	4	0.018
Non-cancer illness code self-reported: polio / poliomyelitis || id:UKB-a:112	MR Egger	4.505	3	0.212
Non-cancer illness code self-reported: polio / poliomyelitis || id:UKB-a:112	Inverse variance weighted	9.053	4	0.060
Non-cancer illness code self-reported: psoriasis || id:UKB-a:100	Maximum likelihood	9.134	14	0.822
Non-cancer illness code self-reported: psoriasis || id:UKB-a:100	MR Egger	9.110	13	0.765
Non-cancer illness code self-reported: psoriasis || id:UKB-a:100	Inverse variance weighted	8.482	14	0.863
Non-cancer illness code self-reported: pulmonary embolism +/- dvt || id:UKB-a:64	Maximum likelihood	8.951	5	0.111
Non-cancer illness code self-reported: pulmonary embolism +/- dvt || id:UKB-a:64	MR Egger	6.084	4	0.193
Non-cancer illness code self-reported: pulmonary embolism +/- dvt || id:UKB-a:64	Inverse variance weighted	7.163	5	0.209
Non-cancer illness code self-reported: rheumatoid arthritis || id:UKB-a:105	Maximum likelihood	9.511	4	0.050
Non-cancer illness code self-reported: rheumatoid arthritis || id:UKB-a:105	MR Egger	7.922	3	0.048
Non-cancer illness code self-reported: rheumatoid arthritis || id:UKB-a:105	Inverse variance weighted	7.138	4	0.129
Non-cancer illness code self-reported: type 2 diabetes || id:UKB-a:75	Maximum likelihood	0.428	2	0.808
Non-cancer illness code self-reported: type 2 diabetes || id:UKB-a:75	MR Egger	0.053	1	0.817
Non-cancer illness code self-reported: type 2 diabetes || id:UKB-a:75	Inverse variance weighted	0.214	2	0.899
Non-cancer illness code self-reported: ulcerative colitis || id:UKB-a:104	Maximum likelihood	6.732	6	0.346
Non-cancer illness code self-reported: ulcerative colitis || id:UKB-a:104	MR Egger	6.714	5	0.243
Non-cancer illness code self-reported: ulcerative colitis || id:UKB-a:104	Inverse variance weighted	5.621	6	0.467
Non-cancer illness code self-reported: uterine fibroids || id:UKB-a:91	Maximum likelihood	11.203	4	0.024
Non-cancer illness code self-reported: uterine fibroids || id:UKB-a:91	MR Egger	2.065	3	0.559
Non-cancer illness code self-reported: uterine fibroids || id:UKB-a:91	Inverse variance weighted	8.403	4	0.078

Non-cancer illness code self-reported: varicose veins || id:UKB-a:110	Maximum likelihood	2.541	1	0.111
Non-cancer illness code self-reported: varicose veins || id:UKB-a:110	Inverse variance weighted	0	1	1
Non-cancer illness code self-reported: vitiligo || id:UKB-a:115	Maximum likelihood	8.852	9	0.451
Non-cancer illness code self-reported: vitiligo || id:UKB-a:115	MR Egger	8.567	8	0.380
Non-cancer illness code self-reported: vitiligo || id:UKB-a:115	Inverse variance weighted	7.869	9	0.547
Number of children fathered || id:UKB-a:304	Maximum likelihood	0.407	1	0.524
Number of children fathered || id:UKB-a:304	Inverse variance weighted	0.000	1	1
Number of cigarettes currently smoked daily (current cigarette smokers) || id:UKB-a:342	Maximum likelihood	0.813	2	0.666
Number of cigarettes currently smoked daily (current cigarette smokers) || id:UKB-a:342	MR Egger	0.813	1	0.367
Number of cigarettes currently smoked daily (current cigarette smokers) || id:UKB-a:342	Inverse variance weighted	0.407	2	0.816
Number of cigarettes previously smoked daily || id:UKB-a:328	Maximum likelihood	1.052	2	0.591
Number of cigarettes previously smoked daily || id:UKB-a:328	MR Egger	1.042	1	0.307
Number of cigarettes previously smoked daily || id:UKB-a:328	Inverse variance weighted	0.527	2	0.769
Number of days/week of moderate physical activity 10+ minutes || id:UKB-a:508	Maximum likelihood	5.545	6	0.476
Number of days/week of moderate physical activity 10+ minutes || id:UKB-a:508	MR Egger	5.457	5	0.363
Number of days/week of moderate physical activity 10+ minutes || id:UKB-a:508	Inverse variance weighted	4.757	6	0.575
Number of days/week of vigorous physical activity 10+ minutes || id:UKB-a:511	Maximum likelihood	18.554	3	0.0003
Number of days/week of vigorous physical activity 10+ minutes || id:UKB-a:511	MR Egger	18.416	2	0.0001
Number of days/week of vigorous physical activity 10+ minutes || id:UKB-a:511	Inverse variance weighted	12.370	3	0.0062
Number of days/week walked 10+ minutes || id:UKB-a:506	Maximum likelihood	15.670	10	0.109
Number of days/week walked 10+ minutes || id:UKB-a:506	MR Egger	11.163	9	0.265
Number of days/week walked 10+ minutes || id:UKB-a:506	Inverse variance weighted	14.107	10	0.168
Number of incorrect matches in round || id:UKB-a:356	Maximum likelihood	14.199	16	0.584
Number of incorrect matches in round || id:UKB-a:356	MR Egger	13.157	15	0.590
Number of incorrect matches in round || id:UKB-a:356	Inverse variance weighted	13.333	16	0.648
Number of live births || id:UKB-a:317	Maximum likelihood	0.057	1	0.812
Number of live births || id:UKB-a:317	Inverse variance weighted	0	1	1
Number of operations self-reported || id:UKB-a:23	Maximum likelihood	8.114	7	0.323
Number of operations self-reported || id:UKB-a:23	MR Egger	8.047	6	0.235
Number of operations self-reported || id:UKB-a:23	Inverse variance weighted	6.991	7	0.430
Number of self-reported cancers || id:UKB-a:21	Maximum likelihood	8.666	7	0.278
Number of self-reported cancers || id:UKB-a:21	MR Egger	7.982	6	0.239
Number of self-reported cancers || id:UKB-a:21	Inverse variance weighted	7.431	7	0.385
Number of self-reported non-cancer illnesses || id:UKB-a:22	Maximum likelihood	18.374	19	0.498
Number of self-reported non-cancer illnesses || id:UKB-a:22	MR Egger	18.084	18	0.450
Number of self-reported non-cancer illnesses || id:UKB-a:22	Inverse variance weighted	17.459	19	0.559
Number of treatments/medications taken || id:UKB-a:24	Maximum likelihood	21.752	21	0.414
Number of treatments/medications taken || id:UKB-a:24	MR Egger	21.264	20	0.382
Number of treatments/medications taken || id:UKB-a:24	Inverse variance weighted	20.717	21	0.476
Obesity class 1 || id:90	Maximum likelihood	37.206	16	0.002
Obesity class 1 || id:90	MR Egger	31.523	15	0.007
Obesity class 1 || id:90	Inverse variance weighted	34.881	16	0.004

Octadecanedioate || id:735	Maximum likelihood	0.603	1	0.437
Octadecanedioate || id:735	Inverse variance weighted	0	1	1
Octanoylcarnitine || id:615	Maximum likelihood	5.132	1	0.023
Octanoylcarnitine || id:615	Inverse variance weighted	0	1	1
Omega-3 fatty acids || id:855	Maximum likelihood	2.569	4	0.632
Omega-3 fatty acids || id:855	MR Egger	2.493	3	0.477
Omega-3 fatty acids || id:855	Inverse variance weighted	1.942	4	0.746
Omega-6 fatty acids || id:856	Maximum likelihood	4.959	10	0.894
Omega-6 fatty acids || id:856	MR Egger	4.879	9	0.845
Omega-6 fatty acids || id:856	Inverse variance weighted	4.470	10	0.924
Omega-9 and saturated fatty acids || id:857	Maximum likelihood	8.073	6	0.233
Omega-9 and saturated fatty acids || id:857	MR Egger	2.727	5	0.742
Omega-9 and saturated fatty acids || id:857	Inverse variance weighted	6.742	6	0.345
Other serious medical condition/disability diagnosed by doctor || id:UKB-a:309	Maximum likelihood	2.858	2	0.240
Other serious medical condition/disability diagnosed by doctor || id:UKB-a:309	MR Egger	1.094	1	0.295
Other serious medical condition/disability diagnosed by doctor || id:UKB-a:309	Inverse variance weighted	1.434	2	0.488
Ovarian cancer || id:1120	Maximum likelihood	6.294	9	0.710
Ovarian cancer || id:1120	MR Egger	5.983	8	0.649
Ovarian cancer || id:1120	Inverse variance weighted	5.598	9	0.779
Overall health rating || id:UKB-a:251	Maximum likelihood	73.900	53	0.030
Overall health rating || id:UKB-a:251	MR Egger	73.128	52	0.028
Overall health rating || id:UKB-a:251	Inverse variance weighted	72.589	53	0.038
Pack years adult smoking as proportion of life span exposed to smoking PREVIEW ONLY || id:UKB-a:238	Maximum likelihood	5.975	10	0.817
Pack years adult smoking as proportion of life span exposed to smoking PREVIEW ONLY || id:UKB-a:238	MR Egger	5.850	9	0.755
Pack years adult smoking as proportion of life span exposed to smoking PREVIEW ONLY || id:UKB-a:238	Inverse variance weighted	5.390	10	0.864
Pack years of smoking PREVIEW ONLY || id:UKB-a:237	Maximum likelihood	2.886	9	0.969
Pack years of smoking PREVIEW ONLY || id:UKB-a:237	MR Egger	2.539	8	0.960
Pack years of smoking PREVIEW ONLY || id:UKB-a:237	Inverse variance weighted	2.573	9	0.979
Packed cell volume || id:274	Maximum likelihood	21.977	12	0.038
Packed cell volume || id:274	MR Egger	21.521	11	0.028
Packed cell volume || id:274	Inverse variance weighted	20.287	12	0.062
Pain type(s) experienced in last month: Back pain || id:UKB-a:473	Maximum likelihood	5.678	7	0.578
Pain type(s) experienced in last month: Back pain || id:UKB-a:473	MR Egger	2.767	6	0.837
Pain type(s) experienced in last month: Back pain || id:UKB-a:473	Inverse variance weighted	4.870	7	0.676
Pain type(s) experienced in last month: Headache || id:UKB-a:469	Maximum likelihood	30.035	30	0.464
Pain type(s) experienced in last month: Headache || id:UKB-a:469	MR Egger	29.983	29	0.415
Pain type(s) experienced in last month: Headache || id:UKB-a:469	Inverse variance weighted	29.040	30	0.516
Pain type(s) experienced in last month: Hip pain || id:UKB-a:475	Maximum likelihood	0.090	2	0.956
Pain type(s) experienced in last month: Hip pain || id:UKB-a:475	MR Egger	0.000	1	0.985
Pain type(s) experienced in last month: Hip pain || id:UKB-a:475	Inverse variance weighted	0.045	2	0.978
Pain type(s) experienced in last month: Knee pain || id:UKB-a:476	Maximum likelihood	4.399	6	0.623
Pain type(s) experienced in last month: Knee pain || id:UKB-a:476	MR Egger	2.775	5	0.735

Pain type(s) experienced in last month: Knee pain || id:UKB-a:476	Inverse variance weighted	3.670	6	0.721
Pain type(s) experienced in last month: Neck or shoulder pain || id:UKB-a:472	Maximum likelihood	1.240	1	0.266
Pain type(s) experienced in last month: Neck or shoulder pain || id:UKB-a:472	Inverse variance weighted	0	1	1
Pain type(s) experienced in last month: None of the above || id:UKB-a:470	Maximum likelihood	12.290	14	0.583
Pain type(s) experienced in last month: None of the above || id:UKB-a:470	MR Egger	9.919	13	0.701
Pain type(s) experienced in last month: None of the above || id:UKB-a:470	Inverse variance weighted	11.484	14	0.648
palmitoleic acid (16:1n7) || id:1156	Maximum likelihood	5.309	4	0.257
palmitoleic acid (16:1n7) || id:1156	MR Egger	5.080	3	0.166
palmitoleic acid (16:1n7) || id:1156	Inverse variance weighted	3.989	4	0.407
Parkinson's disease || id:812	Maximum likelihood	3.774	3	0.287
Parkinson's disease || id:812	MR Egger	3.603	2	0.165
Parkinson's disease || id:812	Inverse variance weighted	2.537	3	0.469
Past tobacco smoking || id:UKB-a:17	Maximum likelihood	59.509	40	0.024
Past tobacco smoking || id:UKB-a:17	MR Egger	59.463	39	0.019
Past tobacco smoking || id:UKB-a:17	Inverse variance weighted	58.036	40	0.032
Peak expiratory flow (PEF) || id:UKB-a:338	Maximum likelihood	115.118	77	0.003
Peak expiratory flow (PEF) || id:UKB-a:338	MR Egger	115.099	76	0.003
Peak expiratory flow (PEF) || id:UKB-a:338	Inverse variance weighted	113.624	77	0.004
Percent emphysema || id:1005	Maximum likelihood	0.034	1	0.853
Percent emphysema || id:1005	Inverse variance weighted	0	1	1
PGC cross-disorder traits || id:803	Maximum likelihood	7.539	3	0.057
PGC cross-disorder traits || id:803	MR Egger	0.869	2	0.648
PGC cross-disorder traits || id:803	Inverse variance weighted	5.079	3	0.166
Phospholipids in IDL || id:871	Maximum likelihood	19.346	19	0.435
Phospholipids in IDL || id:871	MR Egger	16.935	18	0.528
Phospholipids in IDL || id:871	Inverse variance weighted	18.357	19	0.499
Phospholipids in large HDL || id:879	Maximum likelihood	14.975	11	0.184
Phospholipids in large HDL || id:879	MR Egger	13.523	10	0.196
Phospholipids in large HDL || id:879	Inverse variance weighted	13.631	11	0.254
Phospholipids in large LDL || id:885	Maximum likelihood	22.693	19	0.251
Phospholipids in large LDL || id:885	MR Egger	17.257	18	0.506
Phospholipids in large LDL || id:885	Inverse variance weighted	21.542	19	0.308
Phospholipids in large VLDL || id:891	Maximum likelihood	3.202	7	0.866
Phospholipids in large VLDL || id:891	MR Egger	2.664	6	0.850
Phospholipids in large VLDL || id:891	Inverse variance weighted	2.761	7	0.906
Platelet count || id:1008	Maximum likelihood	41.596	31	0.097
Platelet count || id:1008	MR Egger	41.593	30	0.078
Platelet count || id:1008	Inverse variance weighted	40.265	31	0.123
Potassium in urine || id:UKB-a:334	Maximum likelihood	5.381	8	0.716
Potassium in urine || id:UKB-a:334	MR Egger	5.195	7	0.636
Potassium in urine || id:UKB-a:334	Inverse variance weighted	4.713	8	0.788
Primary sclerosing cholangitis || id:1112	Maximum likelihood	22.047	16	0.142

Primary sclerosing cholangitis || id:1112	MR Egger	21.076	15	0.134
Primary sclerosing cholangitis || id:1112	Inverse variance weighted	20.674	16	0.191
Proline || id:355	Maximum likelihood	0.416	2	0.812
Proline || id:355	MR Egger	0.093	1	0.761
Proline || id:355	Inverse variance weighted	0.208	2	0.901
Propionylcarnitine || id:479	Maximum likelihood	2.108	3	0.550
Propionylcarnitine || id:479	MR Egger	0.452	2	0.798
Propionylcarnitine || id:479	Inverse variance weighted	1.406	3	0.704
Prospective memory result || id:UKB-a:197	Maximum likelihood	0.762	1	0.383
Prospective memory result || id:UKB-a:197	Inverse variance weighted	0	1	1
Pulse rate || id:UKB-a:363	Maximum likelihood	47.595	49	0.530
Pulse rate || id:UKB-a:363	MR Egger	47.603	48	0.489
Pulse rate || id:UKB-a:363	Inverse variance weighted	46.634	49	0.570
Pulse wave peak to peak time || id:UKB-a:365	Maximum likelihood	0.830	1	0.362
Pulse wave peak to peak time || id:UKB-a:365	Inverse variance weighted	0	1	1
Pulse wave reflection index || id:UKB-a:364	Maximum likelihood	0.041	1	0.839
Pulse wave reflection index || id:UKB-a:364	Inverse variance weighted	0	1	1
Putamen volume || id:1047	Maximum likelihood	1.363	3	0.714
Putamen volume || id:1047	MR Egger	0.409	2	0.815
Putamen volume || id:1047	Inverse variance weighted	0.911	3	0.823
Pyroglutamine* || id:501	Maximum likelihood	1.232	3	0.745
Pyroglutamine* || id:501	MR Egger	0.309	2	0.857
Pyroglutamine* || id:501	Inverse variance weighted	0.824	3	0.844
Ratio of bisallylic groups to double bonds || id:844	Maximum likelihood	3.426	4	0.489
Ratio of bisallylic groups to double bonds || id:844	MR Egger	2.005	3	0.571
Ratio of bisallylic groups to double bonds || id:844	Inverse variance weighted	2.570	4	0.632
Ratio of bisallylic groups to total fatty acids || id:845	Maximum likelihood	2.924	3	0.404
Ratio of bisallylic groups to total fatty acids || id:845	MR Egger	1.921	2	0.383
Ratio of bisallylic groups to total fatty acids || id:845	Inverse variance weighted	1.949	3	0.583
Reason for reducing amount of alcohol drunk: Health precaution || id:UKB-a:314	Maximum likelihood	0	1	0.999
Reason for reducing amount of alcohol drunk: Health precaution || id:UKB-a:314	Inverse variance weighted	0	1	1
Red blood cell count || id:275	Maximum likelihood	38.276	25	0.043
Red blood cell count || id:275	MR Egger	38.393	24	0.032
Red blood cell count || id:275	Inverse variance weighted	36.887	25	0.059
Rheumatoid arthritis || id:831	Maximum likelihood	15.449	13	0.280
Rheumatoid arthritis || id:831	MR Egger	14.817	12	0.252
Rheumatoid arthritis || id:831	Inverse variance weighted	14.291	13	0.354
Rheumatoid arthritis || id:832	Maximum likelihood	50.037	33	0.029
Rheumatoid arthritis || id:832	MR Egger	49.850	32	0.023
Rheumatoid arthritis || id:832	Inverse variance weighted	48.534	33	0.040
Rheumatoid arthritis || id:833	Maximum likelihood	73.067	44	0.004
Rheumatoid arthritis || id:833	MR Egger	71.846	43	0.004

Rheumatoid arthritis || id:833	Inverse variance weighted	71.409	44	0.006
Rheumatoid arthritis || id:834	Maximum likelihood	12.015	7	0.100
Rheumatoid arthritis || id:834	MR Egger	11.187	6	0.083
Rheumatoid arthritis || id:834	Inverse variance weighted	10.299	7	0.172
Risk taking || id:UKB-a:241	Maximum likelihood	9.617	9	0.382
Risk taking || id:UKB-a:241	MR Egger	6.968	8	0.540
Risk taking || id:UKB-a:241	Inverse variance weighted	8.576	9	0.477
Schizophrenia || id:22	Maximum likelihood	95.869	68	0.015
Schizophrenia || id:22	MR Egger	95.035	67	0.014
Schizophrenia || id:22	Inverse variance weighted	94.465	68	0.019
Seen a psychiatrist for nerves anxiety tension or depression || id:UKB-a:247	Maximum likelihood	1.289	2	0.525
Seen a psychiatrist for nerves anxiety tension or depression || id:UKB-a:247	MR Egger	0.978	1	0.323
Seen a psychiatrist for nerves anxiety tension or depression || id:UKB-a:247	Inverse variance weighted	0.647	2	0.724
Seen doctor (GP) for nerves anxiety tension or depression || id:UKB-a:246	Maximum likelihood	26.474	18	0.089
Seen doctor (GP) for nerves anxiety tension or depression || id:UKB-a:246	MR Egger	26.469	17	0.066
Seen doctor (GP) for nerves anxiety tension or depression || id:UKB-a:246	Inverse variance weighted	25.004	18	0.125
Sensitivity / hurt feelings || id:UKB-a:48	Maximum likelihood	18.308	22	0.688
Sensitivity / hurt feelings || id:UKB-a:48	MR Egger	17.043	21	0.709
Sensitivity / hurt feelings || id:UKB-a:48	Inverse variance weighted	17.476	22	0.737
Serine || id:464	Maximum likelihood	0.010	1	0.922
Serine || id:464	Inverse variance weighted	0	1	1
Serum creatinine (eGFRcrea) || id:1104	Maximum likelihood	45.996	41	0.273
Serum creatinine (eGFRcrea) || id:1104	MR Egger	43.902	40	0.310
Serum creatinine (eGFRcrea) || id:1104	Inverse variance weighted	44.901	41	0.312
Serum creatinine (eGFRcrea) || id:1105	Maximum likelihood	46.188	41	0.267
Serum creatinine (eGFRcrea) || id:1105	MR Egger	45.745	40	0.246
Serum creatinine (eGFRcrea) || id:1105	Inverse variance weighted	45.065	41	0.306
Serum cystatin C (eGFRcys) || id:1106	Maximum likelihood	14.653	4	0.005
Serum cystatin C (eGFRcys) || id:1106	MR Egger	12.765	3	0.005
Serum cystatin C (eGFRcys) || id:1106	Inverse variance weighted	10.989	4	0.027
Sitting height ratio || id:1070	Maximum likelihood	2.533	3	0.469
Sitting height ratio || id:1070	MR Egger	2.113	2	0.348
Sitting height ratio || id:1070	Inverse variance weighted	1.691	3	0.639
Sleep duration || id:1088	Maximum likelihood	2.243	2	0.326
Sleep duration || id:1088	MR Egger	2.249	1	0.134
Sleep duration || id:1088	Inverse variance weighted	1.125	2	0.570
Sleep duration || id:UKB-a:9	Maximum likelihood	59.412	40	0.025
Sleep duration || id:UKB-a:9	MR Egger	53.605	39	0.060
Sleep duration || id:UKB-a:9	Inverse variance weighted	58.000	40	0.033
Sleeplessness / insomnia || id:UKB-a:13	Maximum likelihood	30.221	27	0.304
Sleeplessness / insomnia || id:UKB-a:13	MR Egger	29.179	26	0.303
Sleeplessness / insomnia || id:UKB-a:13	Inverse variance weighted	29.126	27	0.355

Smoking status: Current || id:UKB-a:225	Maximum likelihood	16.421	14	0.288
Smoking status: Current || id:UKB-a:225	MR Egger	14.692	13	0.327
Smoking status: Current || id:UKB-a:225	Inverse variance weighted	15.262	14	0.360
Smoking status: Previous || id:UKB-a:224	Maximum likelihood	28.907	17	0.035
Smoking status: Previous || id:UKB-a:224	MR Egger	28.576	16	0.027
Smoking status: Previous || id:UKB-a:224	Inverse variance weighted	27.208	17	0.055
Snoring || id:UKB-a:14	Maximum likelihood	13.176	21	0.902
Snoring || id:UKB-a:14	MR Egger	12.309	20	0.905
Snoring || id:UKB-a:14	Inverse variance weighted	12.555	21	0.923
Sodium in urine || id:UKB-a:335	Maximum likelihood	30.624	31	0.485
Sodium in urine || id:UKB-a:335	MR Egger	30.627	30	0.434
Sodium in urine || id:UKB-a:335	Inverse variance weighted	29.655	31	0.535
Started insulin within one year diagnosis of diabetes || id:UKB-a:331	Maximum likelihood	8.903	7	0.260
Started insulin within one year diagnosis of diabetes || id:UKB-a:331	MR Egger	8.895	6	0.180
Started insulin within one year diagnosis of diabetes || id:UKB-a:331	Inverse variance weighted	7.631	7	0.366
Stearate (18:0) || id:331	Maximum likelihood	1.181	1	0.277
Stearate (18:0) || id:331	Inverse variance weighted	0	1	1
Stearic acid (18:0) plasma levels || id:1158	Maximum likelihood	1.270	2	0.530
Stearic acid (18:0) plasma levels || id:1158	MR Egger	0.883	1	0.347
Stearic acid (18:0) plasma levels || id:1158	Inverse variance weighted	0.635	2	0.728
Suffer from 'nerves' || id:UKB-a:200	Maximum likelihood	10.589	9	0.305
Suffer from 'nerves' || id:UKB-a:200	MR Egger	10.524	8	0.230
Suffer from 'nerves' || id:UKB-a:200	Inverse variance weighted	9.447	9	0.397
Systemic lupus erythematosus || id:815	Maximum likelihood	4.144	2	0.126
Systemic lupus erythematosus || id:815	MR Egger	4.145	1	0.042
Systemic lupus erythematosus || id:815	Inverse variance weighted	2.113	2	0.348
Systolic blood pressure automated reading || id:UKB-a:360	Maximum likelihood	200.586	161	0.019
Systolic blood pressure automated reading || id:UKB-a:360	MR Egger	200.341	160	0.017
Systolic blood pressure automated reading || id:UKB-a:360	Inverse variance weighted	199.373	161	0.021
Taking other prescription medications || id:UKB-a:310	Maximum likelihood	15.214	9	0.085
Taking other prescription medications || id:UKB-a:310	MR Egger	13.661	8	0.091
Taking other prescription medications || id:UKB-a:310	Inverse variance weighted	13.536	9	0.140
Tense / 'highly strung' || id:UKB-a:52	Maximum likelihood	11.251	15	0.735
Tense / 'highly strung' || id:UKB-a:52	MR Egger	9.698	14	0.784
Tense / 'highly strung' || id:UKB-a:52	Inverse variance weighted	10.521	15	0.786
Tetradecanedioate || id:709	Maximum likelihood	2.164	2	0.339
Tetradecanedioate || id:709	MR Egger	0.461	1	0.497
Tetradecanedioate || id:709	Inverse variance weighted	1.086	2	0.581
Time from waking to first cigarette || id:UKB-a:343	Maximum likelihood	0.302	2	0.860
Time from waking to first cigarette || id:UKB-a:343	MR Egger	0.188	1	0.664
Time from waking to first cigarette || id:UKB-a:343	Inverse variance weighted	0.151	2	0.927
Time spent driving || id:UKB-a:7	Maximum likelihood	0.546	1	0.460

Time spent driving || id:UKB-a:7	Inverse variance weighted	0	1	1
Time spent using computer || id:UKB-a:6	Maximum likelihood	59.668	35	0.006
Time spent using computer || id:UKB-a:6	MR Egger	59.287	34	0.005
Time spent using computer || id:UKB-a:6	Inverse variance weighted	58.065	35	0.008
Time spent watching television (TV) || id:UKB-a:5	Maximum likelihood	67.919	56	0.132
Time spent watching television (TV) || id:UKB-a:5	MR Egger	65.063	55	0.166
Time spent watching television (TV) || id:UKB-a:5	Inverse variance weighted	66.811	56	0.153
Tobacco smoking: Ex-smoker || id:UKB-a:260	Maximum likelihood	3.079	1	0.079
Tobacco smoking: Ex-smoker || id:UKB-a:260	Inverse variance weighted	0	1	1
Total cholesterol || id:301	Maximum likelihood	122.418	84	0.004
Total cholesterol || id:301	MR Egger	122.183	83	0.003
Total cholesterol || id:301	Inverse variance weighted	121.040	84	0.005
Total cholesterol in HDL || id:864	Maximum likelihood	8.100	12	0.777
Total cholesterol in HDL || id:864	MR Egger	7.875	11	0.724
Total cholesterol in HDL || id:864	Inverse variance weighted	7.446	12	0.827
Total cholesterol in IDL || id:867	Maximum likelihood	17.733	18	0.473
Total cholesterol in IDL || id:867	MR Egger	15.630	17	0.550
Total cholesterol in IDL || id:867	Inverse variance weighted	16.775	18	0.539
Total cholesterol in large HDL || id:874	Maximum likelihood	19.232	14	0.156
Total cholesterol in large HDL || id:874	MR Egger	15.120	13	0.300
Total cholesterol in large HDL || id:874	Inverse variance weighted	17.911	14	0.211
Total cholesterol in large LDL || id:880	Maximum likelihood	23.940	20	0.245
Total cholesterol in large LDL || id:880	MR Egger	18.161	19	0.512
Total cholesterol in large LDL || id:880	Inverse variance weighted	22.793	20	0.299
Total cholesterol in large VLDL || id:886	Maximum likelihood	4.179	8	0.841
Total cholesterol in large VLDL || id:886	MR Egger	4.204	7	0.756
Total cholesterol in large VLDL || id:886	Inverse variance weighted	3.679	8	0.885
Total cholesterol in LDL || id:895	Maximum likelihood	25.236	20	0.193
Total cholesterol in LDL || id:895	MR Egger	19.463	19	0.428
Total cholesterol in LDL || id:895	Inverse variance weighted	24.015	20	0.242
Total cholesterol in medium HDL || id:898	Maximum likelihood	3.072	5	0.689
Total cholesterol in medium HDL || id:898	MR Egger	1.993	4	0.737
Total cholesterol in medium HDL || id:898	Inverse variance weighted	2.477	5	0.780
Total lipids in IDL || id:869	Maximum likelihood	19.065	19	0.453
Total lipids in IDL || id:869	MR Egger	16.101	18	0.585
Total lipids in IDL || id:869	Inverse variance weighted	18.100	19	0.516
Total lipids in large HDL || id:877	Maximum likelihood	15.182	11	0.174
Total lipids in large HDL || id:877	MR Egger	13.148	10	0.216
Total lipids in large HDL || id:877	Inverse variance weighted	13.815	11	0.243
Total lipids in large LDL || id:883	Maximum likelihood	22.855	21	0.352
Total lipids in large LDL || id:883	MR Egger	18.553	20	0.551
Total lipids in large LDL || id:883	Inverse variance weighted	21.813	21	0.410

Total lipids in large VLDL || id:889	Maximum likelihood	5.960	8	0.652
Total lipids in large VLDL || id:889	MR Egger	5.555	7	0.593
Total lipids in large VLDL || id:889	Inverse variance weighted	5.235	8	0.732
Total lipids in medium HDL || id:901	Maximum likelihood	3.322	4	0.506
Total lipids in medium HDL || id:901	MR Egger	2.415	3	0.491
Total lipids in medium HDL || id:901	Inverse variance weighted	2.505	4	0.644
trans-cis-18:2 || id:1148	Maximum likelihood	0.122	2	0.941
trans-cis-18:2 || id:1148	MR Egger	0.001	1	0.973
trans-cis-18:2 || id:1148	Inverse variance weighted	0.061	2	0.970
Transferrin || id:1052	Maximum likelihood	4.272	7	0.748
Transferrin || id:1052	MR Egger	3.029	6	0.805
Transferrin || id:1052	Inverse variance weighted	3.663	7	0.818
Transferrin Saturation || id:1051	Maximum likelihood	3.642	2	0.162
Transferrin Saturation || id:1051	MR Egger	0.001	1	0.970
Transferrin Saturation || id:1051	Inverse variance weighted	1.821	2	0.402
Treatment/medication code: allopurinol || id:UKB-a:142	Maximum likelihood	17.114	14	0.250
Treatment/medication code: allopurinol || id:UKB-a:142	MR Egger	16.631	13	0.217
Treatment/medication code: allopurinol || id:UKB-a:142	Inverse variance weighted	15.892	14	0.320
Treatment/medication code: amlodipine || id:UKB-a:150	Maximum likelihood	9.016	18	0.959
Treatment/medication code: amlodipine || id:UKB-a:150	MR Egger	7.922	17	0.968
Treatment/medication code: amlodipine || id:UKB-a:150	Inverse variance weighted	8.517	18	0.970
Treatment/medication code: arcoxia 90mg tablet || id:UKB-a:185	Maximum likelihood	21.255	13	0.068
Treatment/medication code: arcoxia 90mg tablet || id:UKB-a:185	MR Egger	14.656	12	0.261
Treatment/medication code: arcoxia 90mg tablet || id:UKB-a:185	Inverse variance weighted	19.628	13	0.105
Treatment/medication code: aspirin || id:UKB-a:132	Maximum likelihood	0.821	6	0.992
Treatment/medication code: aspirin || id:UKB-a:132	MR Egger	0.815	5	0.976
Treatment/medication code: aspirin || id:UKB-a:132	Inverse variance weighted	0.703	6	0.994
Treatment/medication code: atenolol || id:UKB-a:131	Maximum likelihood	4.591	5	0.468
Treatment/medication code: atenolol || id:UKB-a:131	MR Egger	3.888	4	0.421
Treatment/medication code: atenolol || id:UKB-a:131	Inverse variance weighted	3.674	5	0.597
Treatment/medication code: atorvastatin || id:UKB-a:176	Maximum likelihood	15.912	17	0.530
Treatment/medication code: atorvastatin || id:UKB-a:176	MR Egger	15.965	16	0.455
Treatment/medication code: atorvastatin || id:UKB-a:176	Inverse variance weighted	15.076	17	0.590
Treatment/medication code: atrovent 20micrograms inhaler || id:UKB-a:123	Maximum likelihood	0.011	2	0.995
Treatment/medication code: atrovent 20micrograms inhaler || id:UKB-a:123	MR Egger	0.005	1	0.944
Treatment/medication code: atrovent 20micrograms inhaler || id:UKB-a:123	Inverse variance weighted	0.006	2	0.997
Treatment/medication code: becotide 50 inhaler || id:UKB-a:124	Maximum likelihood	4.239	2	0.120
Treatment/medication code: becotide 50 inhaler || id:UKB-a:124	MR Egger	2.754	1	0.097
Treatment/medication code: becotide 50 inhaler || id:UKB-a:124	Inverse variance weighted	2.126	2	0.345
Treatment/medication code: bendroflumethiazide || id:UKB-a:193	Maximum likelihood	18.810	26	0.844
Treatment/medication code: bendroflumethiazide || id:UKB-a:193	MR Egger	17.608	25	0.859
Treatment/medication code: bendroflumethiazide || id:UKB-a:193	Inverse variance weighted	18.092	26	0.872

Treatment/medication code: bisoprolol || id:UKB-a:148	Maximum likelihood	0.054	1	0.816
Treatment/medication code: bisoprolol || id:UKB-a:148	Inverse variance weighted	0	1	1
Treatment/medication code: carbimazole || id:UKB-a:140	Maximum likelihood	4.506	4	0.342
Treatment/medication code: carbimazole || id:UKB-a:140	MR Egger	4.460	3	0.216
Treatment/medication code: carbimazole || id:UKB-a:140	Inverse variance weighted	3.512	4	0.476
Treatment/medication code: clopidogrel || id:UKB-a:180	Maximum likelihood	0.036	1	0.849
Treatment/medication code: clopidogrel || id:UKB-a:180	Inverse variance weighted	0	1	1
Treatment/medication code: doxazosin || id:UKB-a:149	Maximum likelihood	0	1	1.000
Treatment/medication code: doxazosin || id:UKB-a:149	Inverse variance weighted	0	1	1
Treatment/medication code: eumovate cream || id:UKB-a:152	Maximum likelihood	0.319	2	0.853
Treatment/medication code: eumovate cream || id:UKB-a:152	MR Egger	0.281	1	0.596
Treatment/medication code: eumovate cream || id:UKB-a:152	Inverse variance weighted	0.165	2	0.921
Treatment/medication code: ezetimibe || id:UKB-a:191	Maximum likelihood	1.979	5	0.852
Treatment/medication code: ezetimibe || id:UKB-a:191	MR Egger	1.165	4	0.884
Treatment/medication code: ezetimibe || id:UKB-a:191	Inverse variance weighted	1.643	5	0.896
Treatment/medication code: flecainide || id:UKB-a:163	Maximum likelihood	3.457	2	0.178
Treatment/medication code: flecainide || id:UKB-a:163	MR Egger	0.007	1	0.931
Treatment/medication code: flecainide || id:UKB-a:163	Inverse variance weighted	1.746	2	0.418
Treatment/medication code: gliclazide || id:UKB-a:139	Maximum likelihood	2.477	4	0.649
Treatment/medication code: gliclazide || id:UKB-a:139	MR Egger	2.368	3	0.500
Treatment/medication code: gliclazide || id:UKB-a:139	Inverse variance weighted	1.863	4	0.761
Treatment/medication code: hydroxocobalamin product || id:UKB-a:134	Maximum likelihood	5.524	6	0.479
Treatment/medication code: hydroxocobalamin product || id:UKB-a:134	MR Egger	5.454	5	0.363
Treatment/medication code: hydroxocobalamin product || id:UKB-a:134	Inverse variance weighted	4.606	6	0.595
Treatment/medication code: ibuprofen || id:UKB-a:136	Maximum likelihood	0.393	1	0.531
Treatment/medication code: ibuprofen || id:UKB-a:136	Inverse variance weighted	0	1	1
Treatment/medication code: indivina 1mg/2.5mg tablet || id:UKB-a:181	Maximum likelihood	10.900	10	0.365
Treatment/medication code: indivina 1mg/2.5mg tablet || id:UKB-a:181	MR Egger	4.460	9	0.879
Treatment/medication code: indivina 1mg/2.5mg tablet || id:UKB-a:181	Inverse variance weighted	10.021	10	0.439
Treatment/medication code: insulin product || id:UKB-a:153	Maximum likelihood	8.703	6	0.191
Treatment/medication code: insulin product || id:UKB-a:153	MR Egger	4.546	5	0.474
Treatment/medication code: insulin product || id:UKB-a:153	Inverse variance weighted	7.255	6	0.298
Treatment/medication code: isosorbide dinitrate || id:UKB-a:119	Maximum likelihood	13.159	10	0.215
Treatment/medication code: isosorbide dinitrate || id:UKB-a:119	MR Egger	11.136	9	0.266
Treatment/medication code: isosorbide dinitrate || id:UKB-a:119	Inverse variance weighted	11.853	10	0.295
Treatment/medication code: kapake tablet || id:UKB-a:125	Maximum likelihood	8.579	12	0.738
Treatment/medication code: kapake tablet || id:UKB-a:125	MR Egger	7.888	11	0.723
Treatment/medication code: kapake tablet || id:UKB-a:125	Inverse variance weighted	7.872	12	0.795
Treatment/medication code: letrozole || id:UKB-a:174	Maximum likelihood	2.566	4	0.633
Treatment/medication code: letrozole || id:UKB-a:174	MR Egger	2.574	3	0.462
Treatment/medication code: letrozole || id:UKB-a:174	Inverse variance weighted	1.930	4	0.749
Treatment/medication code: levothyroxine sodium || id:UKB-a:190	Maximum likelihood	76.284	53	0.020

Treatment/medication code: levothyroxine sodium || id:UKB-a:190	MR Egger	76.145	52	0.016
Treatment/medication code: levothyroxine sodium || id:UKB-a:190	Inverse variance weighted	74.880	53	0.026
Treatment/medication code: liothyronine || id:UKB-a:157	Maximum likelihood	7.761	12	0.804
Treatment/medication code: liothyronine || id:UKB-a:157	MR Egger	7.761	11	0.735
Treatment/medication code: liothyronine || id:UKB-a:157	Inverse variance weighted	7.114	12	0.850
Treatment/medication code: lisinopril || id:UKB-a:116	Maximum likelihood	2.164	1	0.141
Treatment/medication code: lisinopril || id:UKB-a:116	Inverse variance weighted	0	1	1
Treatment/medication code: lisinopril+hydrochlorothiazide 10mg/12.5mg tablet || id:UKB-a:127	Maximum likelihood	5.690	6	0.459
Treatment/medication code: lisinopril+hydrochlorothiazide 10mg/12.5mg tablet || id:UKB-a:127	MR Egger	3.756	5	0.585
Treatment/medication code: lisinopril+hydrochlorothiazide 10mg/12.5mg tablet || id:UKB-a:127	Inverse variance weighted	4.743	6	0.577
Treatment/medication code: metformin || id:UKB-a:159	Maximum likelihood	28.672	24	0.233
Treatment/medication code: metformin || id:UKB-a:159	MR Egger	28.511	23	0.197
Treatment/medication code: metformin || id:UKB-a:159	Inverse variance weighted	27.510	24	0.281
Treatment/medication code: morphine || id:UKB-a:137	Maximum likelihood	3.529	3	0.317
Treatment/medication code: morphine || id:UKB-a:137	MR Egger	3.342	2	0.188
Treatment/medication code: morphine || id:UKB-a:137	Inverse variance weighted	2.365	3	0.500
Treatment/medication code: nicorandil || id:UKB-a:165	Maximum likelihood	0.039	1	0.844
Treatment/medication code: nicorandil || id:UKB-a:165	Inverse variance weighted	0	1	1
Treatment/medication code: paracetamol || id:UKB-a:194	Maximum likelihood	13.671	8	0.091
Treatment/medication code: paracetamol || id:UKB-a:194	MR Egger	8.840	7	0.264
Treatment/medication code: paracetamol || id:UKB-a:194	Inverse variance weighted	11.968	8	0.153
Treatment/medication code: prednisolone || id:UKB-a:141	Maximum likelihood	0.404	1	0.525
Treatment/medication code: prednisolone || id:UKB-a:141	Inverse variance weighted	0	1	1
Treatment/medication code: quinine || id:UKB-a:138	Maximum likelihood	0.012	1	0.914
Treatment/medication code: quinine || id:UKB-a:138	Inverse variance weighted	0	1	1
Treatment/medication code: ramipril || id:UKB-a:117	Maximum likelihood	2.382	4	0.666
Treatment/medication code: ramipril || id:UKB-a:117	MR Egger	2.381	3	0.497
Treatment/medication code: ramipril || id:UKB-a:117	Inverse variance weighted	1.786	4	0.775
Treatment/medication code: rhinocort 50micrograms nasal spray || id:UKB-a:144	Maximum likelihood	3.447	5	0.631
Treatment/medication code: rhinocort 50micrograms nasal spray || id:UKB-a:144	MR Egger	3.463	4	0.484
Treatment/medication code: rhinocort 50micrograms nasal spray || id:UKB-a:144	Inverse variance weighted	2.775	5	0.735
Treatment/medication code: rosiglitazone || id:UKB-a:184	Maximum likelihood	0.228	2	0.892
Treatment/medication code: rosiglitazone || id:UKB-a:184	MR Egger	0.066	1	0.798
Treatment/medication code: rosiglitazone || id:UKB-a:184	Inverse variance weighted	0.117	2	0.943
Treatment/medication code: salbutamol || id:UKB-a:151	Maximum likelihood	1.330	2	0.514
Treatment/medication code: salbutamol || id:UKB-a:151	MR Egger	0.387	1	0.534
Treatment/medication code: salbutamol || id:UKB-a:151	Inverse variance weighted	0.666	2	0.717
Treatment/medication code: seretide 50 evohaler || id:UKB-a:183	Maximum likelihood	3.869	5	0.568
Treatment/medication code: seretide 50 evohaler || id:UKB-a:183	MR Egger	3.874	4	0.423
Treatment/medication code: seretide 50 evohaler || id:UKB-a:183	Inverse variance weighted	3.101	5	0.684
Treatment/medication code: simvastatin || id:UKB-a:121	Maximum likelihood	27.563	28	0.488
Treatment/medication code: simvastatin || id:UKB-a:121	MR Egger	27.483	27	0.438

Treatment/medication code: simvastatin || id:UKB-a:121	Inverse variance weighted	26.672	28	0.536
Treatment/medication code: testogel 50mg gel 5g sachet || id:UKB-a:192	Maximum likelihood	6.837	14	0.941
Treatment/medication code: testogel 50mg gel 5g sachet || id:UKB-a:192	MR Egger	5.791	13	0.953
Treatment/medication code: testogel 50mg gel 5g sachet || id:UKB-a:192	Inverse variance weighted	6.372	14	0.956
Treatment/medication code: thyroxine product || id:UKB-a:158	Maximum likelihood	16.996	9	0.049
Treatment/medication code: thyroxine product || id:UKB-a:158	MR Egger	16.277	8	0.039
Treatment/medication code: thyroxine product || id:UKB-a:158	Inverse variance weighted	15.109	9	0.088
Treatment/medication code: tranexamic acid || id:UKB-a:120	Maximum likelihood	0.286	1	0.593
Treatment/medication code: tranexamic acid || id:UKB-a:120	Inverse variance weighted	0	1	1
Treatment/medication code: venlafaxine || id:UKB-a:167	Maximum likelihood	0.689	1	0.406
Treatment/medication code: venlafaxine || id:UKB-a:167	Inverse variance weighted	0	1	1
Treatment/medication code: ventolin 100micrograms inhaler || id:UKB-a:122	Maximum likelihood	16.351	13	0.231
Treatment/medication code: ventolin 100micrograms inhaler || id:UKB-a:122	MR Egger	16.060	12	0.189
Treatment/medication code: ventolin 100micrograms inhaler || id:UKB-a:122	Inverse variance weighted	15.097	13	0.301
Treatment/medication code: warfarin || id:UKB-a:160	Maximum likelihood	17.468	6	0.008
Treatment/medication code: warfarin || id:UKB-a:160	MR Egger	13.194	5	0.022
Treatment/medication code: warfarin || id:UKB-a:160	Inverse variance weighted	14.562	6	0.024
Triglycerides || id:302	Maximum likelihood	59.671	53	0.246
Triglycerides || id:302	MR Egger	54.845	52	0.367
Triglycerides || id:302	Inverse variance weighted	58.551	53	0.279
Triglycerides in IDL || id:872	Maximum likelihood	9.454	16	0.894
Triglycerides in IDL || id:872	MR Egger	7.958	15	0.925
Triglycerides in IDL || id:872	Inverse variance weighted	8.879	16	0.918
Triglycerides in large VLDL || id:892	Maximum likelihood	5.647	8	0.687
Triglycerides in large VLDL || id:892	MR Egger	5.600	7	0.587
Triglycerides in large VLDL || id:892	Inverse variance weighted	4.956	8	0.762
Trunk fat percentage || id:UKB-a:290	Maximum likelihood	373.663	232	0.000
Trunk fat percentage || id:UKB-a:290	MR Egger	373.366	231	0.000
Trunk fat percentage || id:UKB-a:290	Inverse variance weighted	372.056	232	0.000
Tryptophan || id:304	Maximum likelihood	34.740	17	0.007
Tryptophan || id:304	MR Egger	34.814	16	0.004
Tryptophan || id:304	Inverse variance weighted	32.766	17	0.012
Type 2 diabetes || id:1090	Maximum likelihood	5.621	1	0.018
Type 2 diabetes || id:1090	Inverse variance weighted	0	1	1
Type 2 diabetes || id:23	Maximum likelihood	19.450	24	0.728
Type 2 diabetes || id:23	MR Egger	19.105	23	0.695
Type 2 diabetes || id:23	Inverse variance weighted	18.645	24	0.771
Type 2 diabetes || id:24	Maximum likelihood	36.368	35	0.405
Type 2 diabetes || id:24	MR Egger	36.342	34	0.360
Type 2 diabetes || id:24	Inverse variance weighted	35.363	35	0.451
Type 2 diabetes || id:25	Maximum likelihood	8.831	24	0.998
Type 2 diabetes || id:25	MR Egger	8.683	23	0.997

Type 2 diabetes || id:25	Inverse variance weighted	8.468	24	0.999
Type 2 diabetes || id:26	Maximum likelihood	7.364	9	0.599
Type 2 diabetes || id:26	MR Egger	7.290	8	0.506
Type 2 diabetes || id:26	Inverse variance weighted	6.562	9	0.683
Types of physical activity in last 4 weeks: Heavy DIY (eg: weeding lawn mowing carpentry digging) || id:UKB-a:487	Maximum likelihood	0.316	2	0.854
Types of physical activity in last 4 weeks: Heavy DIY (eg: weeding lawn mowing carpentry digging) || id:UKB-a:487	MR Egger	0.001	1	0.973
Types of physical activity in last 4 weeks: Heavy DIY (eg: weeding lawn mowing carpentry digging) || id:UKB-a:487	Inverse variance weighted	0.158	2	0.924
Types of physical activity in last 4 weeks: Light DIY (eg: pruning watering the lawn) || id:UKB-a:486	Maximum likelihood	0.848	1	0.357
Types of physical activity in last 4 weeks: Light DIY (eg: pruning watering the lawn) || id:UKB-a:486	Inverse variance weighted	0	1	1
Types of physical activity in last 4 weeks: None of the above || id:UKB-a:483	Maximum likelihood	7.380	6	0.287
Types of physical activity in last 4 weeks: None of the above || id:UKB-a:483	MR Egger	7.295	5	0.200
Types of physical activity in last 4 weeks: None of the above || id:UKB-a:483	Inverse variance weighted	6.157	6	0.406
Types of physical activity in last 4 weeks: Other exercises (eg: swimming cycling keep fit bowling) || id:UKB-a:484	Maximum likelihood	6.082	6	0.414
Types of physical activity in last 4 weeks: Other exercises (eg: swimming cycling keep fit bowling) || id:UKB-a:484	MR Egger	5.243	5	0.387
Types of physical activity in last 4 weeks: Other exercises (eg: swimming cycling keep fit bowling) || id:UKB-a:484	Inverse variance weighted	5.069	6	0.535
Types of physical activity in last 4 weeks: Strenuous sports || id:UKB-a:485	Maximum likelihood	6.214	2	0.045
Types of physical activity in last 4 weeks: Strenuous sports || id:UKB-a:485	MR Egger	0.009	1	0.926
Types of physical activity in last 4 weeks: Strenuous sports || id:UKB-a:485	Inverse variance weighted	3.116	2	0.211
Types of physical activity in last 4 weeks: Walking for pleasure (not as a means of transport) || id:UKB-a:482	Maximum likelihood	2.619	5	0.758
Types of physical activity in last 4 weeks: Walking for pleasure (not as a means of transport) || id:UKB-a:482	MR Egger	0.827	4	0.935
Types of physical activity in last 4 weeks: Walking for pleasure (not as a means of transport) || id:UKB-a:482	Inverse variance weighted	2.111	5	0.834
Types of transport used (excluding work): Cycle || id:UKB-a:481	Maximum likelihood	2.236	2	0.327
Types of transport used (excluding work): Cycle || id:UKB-a:481	MR Egger	1.775	1	0.183
Types of transport used (excluding work): Cycle || id:UKB-a:481	Inverse variance weighted	1.118	2	0.572
Types of transport used (excluding work): Walk || id:UKB-a:479	Maximum likelihood	2.129	1	0.145
Types of transport used (excluding work): Walk || id:UKB-a:479	Inverse variance weighted	0	1	1
Tyrosine || id:325	Maximum likelihood	0.774	2	0.679
Tyrosine || id:325	MR Egger	0.000001	1	0.999
Tyrosine || id:325	Inverse variance weighted	0.394	2	0.821
Ulcerative colitis || id:32	Maximum likelihood	40.207	34	0.214
Ulcerative colitis || id:32	MR Egger	40.193	33	0.182
Ulcerative colitis || id:32	Inverse variance weighted	39.024	34	0.254
Underlying (primary) cause of death: ICD10: J84.1 Other interstitial pulmonary diseases with fibrosis || id:UKB-a:358	Maximum likelihood	11.099	6	0.085
Underlying (primary) cause of death: ICD10: J84.1 Other interstitial pulmonary diseases with fibrosis || id:UKB-a:358	MR Egger	7.676	5	0.175
Underlying (primary) cause of death: ICD10: J84.1 Other interstitial pulmonary diseases with fibrosis || id:UKB-a:358	Inverse variance weighted	9.272	6	0.159
Urate || id:1055	Maximum likelihood	36.093	23	0.040
Urate || id:1055	MR Egger	35.136	22	0.037
Urate || id:1055	Inverse variance weighted	34.526	23	0.058
Uridine || id:316	Maximum likelihood	1.526	2	0.466
Uridine || id:316	MR Egger	0.950	1	0.330
Uridine || id:316	Inverse variance weighted	0.765	2	0.682
Usual walking pace || id:UKB-a:513	Maximum likelihood	41.838	28	0.045

Usual walking pace || id:UKB-a:513	MR Egger	37.923	27	0.079
Usual walking pace || id:UKB-a:513	Inverse variance weighted	40.349	28	0.062
Waist circumference || id:102	Maximum likelihood	0.202	1	0.654
Waist circumference || id:102	Inverse variance weighted	0	1	1
Waist circumference || id:103	Maximum likelihood	1.067	1	0.302
Waist circumference || id:103	Inverse variance weighted	0	1	1
Waist circumference || id:105	Maximum likelihood	1.345	4	0.854
Waist circumference || id:105	MR Egger	1.370	3	0.712
Waist circumference || id:105	Inverse variance weighted	1.028	4	0.906
Waist circumference || id:60	Maximum likelihood	55.790	45	0.130
Waist circumference || id:60	MR Egger	52.018	44	0.190
Waist circumference || id:60	Inverse variance weighted	54.551	45	0.156
Waist circumference || id:61	Maximum likelihood	47.372	39	0.168
Waist circumference || id:61	MR Egger	47.170	38	0.146
Waist circumference || id:61	Inverse variance weighted	46.161	39	0.200
Waist circumference || id:62	Maximum likelihood	25.142	17	0.092
Waist circumference || id:62	MR Egger	24.949	16	0.071
Waist circumference || id:62	Inverse variance weighted	23.671	17	0.129
Waist circumference || id:63	Maximum likelihood	21.685	16	0.154
Waist circumference || id:63	MR Egger	21.305	15	0.127
Waist circumference || id:63	Inverse variance weighted	20.334	16	0.206
Waist circumference || id:64	Maximum likelihood	20.179	14	0.125
Waist circumference || id:64	MR Egger	14.189	13	0.361
Waist circumference || id:64	Inverse variance weighted	18.737	14	0.175
Waist circumference || id:65	Maximum likelihood	21.067	13	0.072
Waist circumference || id:65	MR Egger	12.807	12	0.383
Waist circumference || id:65	Inverse variance weighted	19.447	13	0.110
Waist circumference || id:66	Maximum likelihood	69.124	62	0.249
Waist circumference || id:66	MR Egger	69.084	61	0.223
Waist circumference || id:66	Inverse variance weighted	68.040	62	0.279
Waist circumference || id:67	Maximum likelihood	71.263	61	0.173
Waist circumference || id:67	MR Egger	70.850	60	0.160
Waist circumference || id:67	Inverse variance weighted	70.135	61	0.198
Waist circumference || id:68	Maximum likelihood	24.506	25	0.490
Waist circumference || id:68	MR Egger	24.506	24	0.433
Waist circumference || id:68	Inverse variance weighted	23.543	25	0.546
Waist circumference || id:69	Maximum likelihood	22.554	21	0.368
Waist circumference || id:69	MR Egger	22.501	20	0.314
Waist circumference || id:69	Inverse variance weighted	21.492	21	0.429
Waist circumference || id:70	Maximum likelihood	28.320	22	0.165
Waist circumference || id:70	MR Egger	28.061	21	0.138
Waist circumference || id:70	Inverse variance weighted	27.058	22	0.209

Waist circumference || id:71	Maximum likelihood	33.792	26	0.140
Waist circumference || id:71	MR Egger	33.525	25	0.118
Waist circumference || id:71	Inverse variance weighted	32.507	26	0.177
Waist-to-hip ratio || id:109	Maximum likelihood	4.851	4	0.303
Waist-to-hip ratio || id:109	MR Egger	3.449	3	0.327
Waist-to-hip ratio || id:109	Inverse variance weighted	3.714	4	0.446
Waist-to-hip ratio || id:111	Maximum likelihood	5.452	8	0.708
Waist-to-hip ratio || id:111	MR Egger	5.254	7	0.629
Waist-to-hip ratio || id:111	Inverse variance weighted	4.775	8	0.781
Waist-to-hip ratio || id:72	Maximum likelihood	32.129	29	0.314
Waist-to-hip ratio || id:72	MR Egger	30.477	28	0.341
Waist-to-hip ratio || id:72	Inverse variance weighted	31.031	29	0.364
Waist-to-hip ratio || id:73	Maximum likelihood	25.300	27	0.558
Waist-to-hip ratio || id:73	MR Egger	23.394	26	0.611
Waist-to-hip ratio || id:73	Inverse variance weighted	24.373	27	0.610
Waist-to-hip ratio || id:80	Maximum likelihood	20.740	32	0.937
Waist-to-hip ratio || id:80	MR Egger	20.755	31	0.918
Waist-to-hip ratio || id:80	Inverse variance weighted	20.109	32	0.949
Waist-to-hip ratio || id:81	Maximum likelihood	21.603	32	0.918
Waist-to-hip ratio || id:81	MR Egger	21.464	31	0.899
Waist-to-hip ratio || id:81	Inverse variance weighted	20.933	32	0.933
Waist-to-hip ratio || id:82	Maximum likelihood	5.819	5	0.324
Waist-to-hip ratio || id:82	MR Egger	5.815	4	0.213
Waist-to-hip ratio || id:82	Inverse variance weighted	4.657	5	0.459
Waist-to-hip ratio || id:83	Maximum likelihood	4.270	4	0.371
Waist-to-hip ratio || id:83	MR Egger	3.219	3	0.359
Waist-to-hip ratio || id:83	Inverse variance weighted	3.211	4	0.523
Weight || id:106	Maximum likelihood	7.841	7	0.347
Weight || id:106	MR Egger	7.039	6	0.317
Weight || id:106	Inverse variance weighted	6.760	7	0.454
Weight || id:107	Maximum likelihood	10.218	10	0.422
Weight || id:107	MR Egger	8.418	9	0.493
Weight || id:107	Inverse variance weighted	9.282	10	0.506
Worrier / anxious feelings || id:UKB-a:51	Maximum likelihood	31.903	32	0.472
Worrier / anxious feelings || id:UKB-a:51	MR Egger	31.066	31	0.463
Worrier / anxious feelings || id:UKB-a:51	Inverse variance weighted	30.913	32	0.521
Worry too long after embarrassment || id:UKB-a:53	Maximum likelihood	19.940	13	0.097
Worry too long after embarrassment || id:UKB-a:53	MR Egger	16.958	12	0.151
Worry too long after embarrassment || id:UKB-a:53	Inverse variance weighted	18.415	13	0.142
X-02269 || id:485	Maximum likelihood	0.643	2	0.725
X-02269 || id:485	MR Egger	0.419	1	0.518
X-02269 || id:485	Inverse variance weighted	0.322	2	0.851

X-03056--N-[3-(2-Oxopyrrolidin-1-yl)propyl]acetamide || id:505	Maximum likelihood	2.371	3	0.499
X-03056--N-[3-(2-Oxopyrrolidin-1-yl)propyl]acetamide || id:505	MR Egger	0.196	2	0.906
X-03056--N-[3-(2-Oxopyrrolidin-1-yl)propyl]acetamide || id:505	Inverse variance weighted	1.609	3	0.657
X-03094 || id:373	Maximum likelihood	5.839	4	0.211
X-03094 || id:373	MR Egger	5.850	3	0.119
X-03094 || id:373	Inverse variance weighted	4.388	4	0.356
X-08402 || id:426	Maximum likelihood	1.417	1	0.234
X-08402 || id:426	Inverse variance weighted	0	1	1
X-08988 || id:435	Maximum likelihood	0.835	1	0.361
X-08988 || id:435	Inverse variance weighted	0	1	1
X-10510 || id:444	Maximum likelihood	1.605	2	0.448
X-10510 || id:444	MR Egger	0.737	1	0.391
X-10510 || id:444	Inverse variance weighted	0.802	2	0.670
X-11204 || id:484	Maximum likelihood	1.134	1	0.287
X-11204 || id:484	Inverse variance weighted	0	1	1
X-11261 || id:490	Maximum likelihood	0.130	1	0.719
X-11261 || id:490	Inverse variance weighted	0	1	1
X-11315 || id:495	Maximum likelihood	0.095	2	0.954
X-11315 || id:495	MR Egger	0.003	1	0.953
X-11315 || id:495	Inverse variance weighted	0.050	2	0.975
X-11317 || id:496	Maximum likelihood	0.188	1	0.665
X-11317 || id:496	Inverse variance weighted	0	1	1
X-11327 || id:498	Maximum likelihood	0.970	1	0.325
X-11327 || id:498	Inverse variance weighted	0	1	1
X-11423--O-sulfo-L-tyrosine || id:509	Maximum likelihood	0.395	1	0.530
X-11423--O-sulfo-L-tyrosine || id:509	Inverse variance weighted	0	1	1
X-11440 || id:513	Maximum likelihood	3.380	2	0.185
X-11440 || id:513	MR Egger	0.191	1	0.662
X-11440 || id:513	Inverse variance weighted	1.691	2	0.429
X-11445--5-alpha-pregnan-3beta 20alpha-disulfate || id:517	Maximum likelihood	1.070	2	0.585
X-11445--5-alpha-pregnan-3beta 20alpha-disulfate || id:517	MR Egger	0.816	1	0.366
X-11445--5-alpha-pregnan-3beta 20alpha-disulfate || id:517	Inverse variance weighted	0.536	2	0.764
X-11469 || id:519	Maximum likelihood	0.430	2	0.807
X-11469 || id:519	MR Egger	0.023	1	0.879
X-11469 || id:519	Inverse variance weighted	0.215	2	0.898
X-11491 || id:524	Maximum likelihood	0.872	2	0.647
X-11491 || id:524	MR Egger	0.363	1	0.547
X-11491 || id:524	Inverse variance weighted	0.436	2	0.804
X-11529 || id:528	Maximum likelihood	0.552	1	0.457
X-11529 || id:528	Inverse variance weighted	0	1	1
X-11538 || id:531	Maximum likelihood	0.989	2	0.610
X-11538 || id:531	MR Egger	0.235	1	0.628

X-11538 || id:531	Inverse variance weighted	0.495	2	0.781
X-11593--O-methylascorbate* || id:536	Maximum likelihood	13.541	5	0.019
X-11593--O-methylascorbate* || id:536	MR Egger	9.352	4	0.053
X-11593--O-methylascorbate* || id:536	Inverse variance weighted	10.839	5	0.055
X-11787 || id:541	Maximum likelihood	0.005	1	0.943
X-11787 || id:541	Inverse variance weighted	0	1	1
X-11792 || id:542	Maximum likelihood	2.096	2	0.351
X-11792 || id:542	MR Egger	0	1	0.989
X-11792 || id:542	Inverse variance weighted	1.055	2	0.590
X-11793--oxidized bilirubin* || id:543	Maximum likelihood	0.238	1	0.625
X-11793--oxidized bilirubin* || id:543	Inverse variance weighted	0	1	1
X-11905 || id:560	Maximum likelihood	1.754	1	0.185
X-11905 || id:560	Inverse variance weighted	0	1	1
X-12040 || id:568	Maximum likelihood	0.153	1	0.696
X-12040 || id:568	Inverse variance weighted	0	1	1
X-12063 || id:570	Maximum likelihood	3.472	5	0.628
X-12063 || id:570	MR Egger	2.291	4	0.682
X-12063 || id:570	Inverse variance weighted	2.778	5	0.734
X-12092 || id:580	Maximum likelihood	2.641	4	0.620
X-12092 || id:580	MR Egger	2.566	3	0.463
X-12092 || id:580	Inverse variance weighted	1.982	4	0.739
X-12100--hydroxytryptophan* || id:584	Maximum likelihood	0.024	1	0.877
X-12100--hydroxytryptophan* || id:584	Inverse variance weighted	0	1	1
X-12188 || id:587	Maximum likelihood	0.024	1	0.878
X-12188 || id:587	Inverse variance weighted	0	1	1
X-12189 || id:588	Maximum likelihood	0.010	1	0.921
X-12189 || id:588	Inverse variance weighted	0	1	1
X-12244--N-acetylcarnosine || id:596	Maximum likelihood	1.986	2	0.370
X-12244--N-acetylcarnosine || id:596	MR Egger	0.634	1	0.426
X-12244--N-acetylcarnosine || id:596	Inverse variance weighted	0.995	2	0.608
X-12456 || id:613	Maximum likelihood	0.730	1	0.393
X-12456 || id:613	Inverse variance weighted	0	1	1
X-12510--2-aminooctanoic acid || id:629	Maximum likelihood	0.272	2	0.873
X-12510--2-aminooctanoic acid || id:629	MR Egger	0.261	1	0.609
X-12510--2-aminooctanoic acid || id:629	Inverse variance weighted	0.136	2	0.934
X-12524 || id:630	Maximum likelihood	0.167	1	0.683
X-12524 || id:630	Inverse variance weighted	0	1	1
X-12556 || id:633	Maximum likelihood	5.019	1	0.025
X-12556 || id:633	Inverse variance weighted	0	1	1
X-12644 || id:636	Maximum likelihood	0.660	2	0.719
X-12644 || id:636	MR Egger	0.143	1	0.705
X-12644 || id:636	Inverse variance weighted	0.330	2	0.848

X-12696 || id:640	Maximum likelihood	0.505	1	0.477
X-12696 || id:640	Inverse variance weighted	0.000	1	1
X-12712 || id:642	Maximum likelihood	0.467	1	0.494
X-12712 || id:642	Inverse variance weighted	0.000	1	1
X-12728 || id:646	Maximum likelihood	3.418	7	0.844
X-12728 || id:646	MR Egger	2.464	6	0.872
X-12728 || id:646	Inverse variance weighted	2.931	7	0.891
X-12798 || id:661	Maximum likelihood	0.283	2	0.868
X-12798 || id:661	MR Egger	0.055	1	0.815
X-12798 || id:661	Inverse variance weighted	0.142	2	0.932
X-12844 || id:665	Maximum likelihood	3.519	2	0.172
X-12844 || id:665	MR Egger	0.028	1	0.867
X-12844 || id:665	Inverse variance weighted	1.761	2	0.415
X-12850 || id:667	Maximum likelihood	0.404	2	0.817
X-12850 || id:667	MR Egger	0.037	1	0.848
X-12850 || id:667	Inverse variance weighted	0.204	2	0.903
X-12855 || id:670	Maximum likelihood	0.084	1	0.772
X-12855 || id:670	Inverse variance weighted	0	1	1
X-13215 || id:675	Maximum likelihood	0.018	1	0.893
X-13215 || id:675	Inverse variance weighted	0	1	1
X-13429 || id:683	Maximum likelihood	1.496	2	0.473
X-13429 || id:683	MR Egger	0.035	1	0.852
X-13429 || id:683	Inverse variance weighted	0.750	2	0.687
X-13431--nonanoylcarnitine* || id:684	Maximum likelihood	0.494	1	0.482
X-13431--nonanoylcarnitine* || id:684	Inverse variance weighted	0	1	1
X-13435 || id:685	Maximum likelihood	0.084	1	0.772
X-13435 || id:685	Inverse variance weighted	0	1	1
X-13496 || id:691	Maximum likelihood	2.125	1	0.145
X-13496 || id:691	Inverse variance weighted	0	1	1
X-13548 || id:693	Maximum likelihood	0.090	1	0.765
X-13548 || id:693	Inverse variance weighted	0	1	1
X-14189--leucylalanine || id:719	Maximum likelihood	4.066	1	0.044
X-14189--leucylalanine || id:719	Inverse variance weighted	0	1	1
X-14205--alpha-glutamyltyrosine || id:720	Maximum likelihood	0.226	1	0.635
X-14205--alpha-glutamyltyrosine || id:720	Inverse variance weighted	0	1	1
X-14208--phenylalanylserine || id:721	Maximum likelihood	0.004	1	0.949
X-14208--phenylalanylserine || id:721	Inverse variance weighted	0	1	1
X-14304--leucylalanine || id:722	Maximum likelihood	4.036	1	0.045
X-14304--leucylalanine || id:722	Inverse variance weighted	0	1	1
X-14626 || id:729	Maximum likelihood	0.769	1	0.381
X-14626 || id:729	Inverse variance weighted	0	1	1
X-18601 || id:396	Maximum likelihood	4.740	2	0.093

X-18601 || id:396	MR Egger	0.130	1	0.718
X-18601 || id:396	Inverse variance weighted	2.374	2	0.305
Years of schooling || id:1001	Maximum likelihood	92.298	58	0.003
Years of schooling || id:1001	MR Egger	89.021	57	0.004
Years of schooling || id:1001	Inverse variance weighted	90.892	58	0.004
Years of schooling || id:1010	Maximum likelihood	20.726	12	0.055
Years of schooling || id:1010	MR Egger	20.560	11	0.038
Years of schooling || id:1010	Inverse variance weighted	19.123	12	0.086
Years of schooling || id:1011	Maximum likelihood	16.466	18	0.560
Years of schooling || id:1011	MR Egger	16.367	17	0.498
Years of schooling || id:1011	Inverse variance weighted	15.633	18	0.618
Years of schooling || id:837	Maximum likelihood	3.217	3	0.359
Years of schooling || id:837	MR Egger	2.136	2	0.344
Years of schooling || id:837	Inverse variance weighted	2.161	3	0.540
Zinc || id:1079	Maximum likelihood	0.026	1	0.873
Zinc || id:1079	Inverse variance weighted	0	1	1

id: specific code attributed to each trait on MR Base, Q: Cochran's Q test estimates, Q: Cochran's Q test degree freedom

Table S5. SNPs used to construct the three instruments causally linked to ALS

A. LDL cholesterol || id:300

SNP	effect allele	other allele	eaf	beta	se	p-value
rs7254892	G	A	0·968	0·485	0·012	1·00E-200
rs646776	T	C	0·788	0·160	0·004	1·00E-200
rs6511720	G	T	0·902	0·221	0·006	1·00E-200
rs1367117	A	G	0·288	0·119	0·004	9·48E-183
rs11591147	G	T	0·983	0·497	0·018	8·58E-143
rs12721109	G	A	0·983	0·446	0·018	2·99E-122
rs6544713	T	C	0·294	0·081	0·004	4·84E-83
rs12916	C	T	0·431	0·073	0·004	7·79E-78
rs2965157	T	C	0·979	0·189	0·011	7·29E-62
rs2954029	A	T	0·532	0·056	0·004	2·10E-50
rs579459	C	T	0·215	0·067	0·005	2·42E-44
rs2228603	C	T	0·929	0·104	0·007	4·43E-44
rs2000999	A	G	0·185	0·065	0·005	4·22E-41
rs174583	C	T	0·625	0·052	0·004	7·00E-41
rs247616	C	T	0·707	0·055	0·004	2·57E-37
rs7551981	T	G	0·595	0·047	0·004	1·36E-33
rs6882076	C	T	0·666	0·046	0·004	3·31E-31
rs6065311	C	T	0·460	0·042	0·004	1·66E-30
rs964184	G	C	NA	0·086	0·008	2·01E-26
rs2587534	A	G	0·528	0·039	0·004	8·06E-25
rs75687619	T	G	0·024	0·174	0·016	8·05E-24
rs9987289	G	A	0·925	0·071	0·007	8·53E-24
rs2073547	G	A	0·194	0·049	0·005	1·92E-21
rs1564348	C	T	0·145	0·048	0·005	2·76E-21
rs10893499	A	G	0·144	0·052	0·005	3·86E-21
rs1169288	C	A	0·334	0·038	0·004	6·45E-21
rs2738459	A	C	0·555	0·053	0·006	2·26E-19
rs6016373	A	G	0·627	0·035	0·004	7·95E-19
rs10947332	A	G	0·132	0·050	0·006	6·97E-18
rs3757354	C	T	0·790	0·038	0·004	2·09E-17
rs7832643	T	G	0·405	0·034	0·004	2·67E-17
rs10903129	G	A	0·537	0·033	0·004	3·03E-17
rs13277801	C	T	0·347	0·034	0·004	3·99E-17
rs2642438	G	A	0·745	0·035	0·004	7·32E-16
rs8017377	A	G	0·459	0·030	0·004	2·52E-15
rs4722551	C	T	0·170	0·039	0·005	3·95E-14
rs10832962	T	C	0·719	0·032	0·004	6·62E-14
rs2737252	G	A	0·744	0·031	0·004	7·04E-14
rs2419604	A	G	0·318	0·030	0·004	7·49E-14
rs4970712	C	A	0·806	0·034	0·004	2·46E-13
rs6504872	T	C	0·472	0·027	0·004	3·48E-13
rs10490626	G	A	0·921	0·051	0·007	1·70E-12
rs12748152	T	C	0·071	0·050	0·007	3·21E-12
rs4530754	A	G	0·582	0·028	0·004	3·58E-12
rs3184504	C	T	0·534	0·027	0·004	4·20E-12
rs2315065	A	C	0·087	0·110	0·016	5·23E-12
rs16831243	T	C	0·181	0·038	0·006	9·06E-12
rs72902576	T	G	0·963	0·093	0·013	9·58E-12
rs1801689	C	A	0·037	0·103	0·014	9·81E-12
rs7534572	G	C	0·690	0·041	0·006	1·29E-11
rs676388	C	T	0·463	0·027	0·004	1·31E-11
rs9875338	G	A	0·612	0·027	0·004	2·21E-11
rs4942486	T	C	0·462	0·024	0·004	2·26E-11
rs2886232	T	C	0·120	0·045	0·006	3·88E-11
rs1883025	C	T	0·757	0·030	0·004	6·14E-11
rs6909746	C	T	0·608	0·026	0·004	7·86E-11
rs314253	T	C	0·665	0·024	0·004	3·44E-10
rs364585	G	A	0·633	0·025	0·004	4·28E-10
rs6709904	A	G	0·887	0·055	0·009	4·58E-10
rs1800961	C	T	0·966	0·069	0·011	6·03E-10
rs112201728	T	C	0·058	0·068	0·010	8·51E-10

rs3780181	A	G	0·947	0·045	0·007	1·76E-09
rs17404153	G	T	0·856	0·034	0·005	1·83E-09
rs1408272	T	G	0·947	0·052	0·008	3·68E-09
rs267733	A	G	0·863	0·033	0·005	5·29E-09
rs2328223	C	A	0·249	0·030	0·005	5·63E-09
rs2710642	A	G	0·619	0·024	0·004	6·09E-09
rs16891156	C	A	0·018	0·097	0·017	8·23E-09
rs2030746	T	C	0·398	0·021	0·004	8·61E-09
rs7640978	C	T	0·895	0·039	0·007	9·84E-09
rs12066643	C	T	0·881	0·039	0·006	1·06E-08
rs5763662	T	C	0·025	0·077	0·012	1·19E-08
rs6818397	T	G	0·413	0·022	0·004	1·68E-08
rs2390536	A	G	0·368	0·022	0·004	2·04E-08
rs1250229	C	T	0·789	0·024	0·004	3·13E-08
rs4253776	G	A	0·124	0·031	0·006	3·35E-08
rs2495495	T	C	0·135	0·034	0·006	3·52E-08
rs10195252	T	C	0·582	0·024	0·004	3·81E-08
rs11563251	T	C	0·125	0·035	0·006	4·50E-08
rs13206249	G	A	0·784	0·038	0·006	4·53E-08

SNP, single nucleotide polymorphism; se, standard error; eaf, effect allele frequency.

B. Coronary heart disease || id:7

SNP	effect allele	other allele	eaf	beta	se	p-value
rs2891168	A	G	0·511	-0·193	0·009	2·29E-98
rs9349379	A	G	0·568	-0·132	0·010	1·81E-42
rs55730499	C	T	0·944	-0·317	0·024	5·39E-39
rs7528419	A	G	0·786	0·115	0·011	1·97E-23
rs115654617	C	A	0·893	-0·138	0·016	3·12E-18
rs4468572	C	T	0·586	0·077	0·010	4·44E-16
rs10080815	T	G	0·972	-0·247	0·031	1·33E-15
rs28451064	G	A	0·879	-0·128	0·016	1·33E-15
rs56289821	G	A	0·900	0·134	0·017	4·44E-15
rs1870634	G	T	0·637	0·076	0·010	5·55E-15
rs9970807	C	T	0·915	0·126	0·017	5·00E-14
rs67180937	G	T	0·663	0·079	0·011	1·01E-12
rs1412444	C	T	0·631	-0·067	0·010	5·15E-12
rs2519093	C	T	0·809	-0·080	0·012	1·19E-11
rs12202017	A	G	0·700	0·067	0·010	1·98E-11
rs2487928	G	A	0·582	-0·063	0·010	4·41E-11
rs11556924	C	T	0·687	0·073	0·011	5·34E-11
rs2681472	A	G	0·799	-0·074	0·011	6·17E-11
rs2128739	C	A	0·676	-0·066	0·010	7·05E-11
rs4420638	A	G	0·834	-0·092	0·014	7·07E-11
rs2107595	G	A	0·800	-0·073	0·011	8·05E-11
rs180803	T	G	0·029	-0·181	0·028	1·64E-10
rs11838776	G	A	0·737	-0·069	0·011	1·83E-10
rs11065979	C	T	0·635	-0·069	0·011	1·93E-10
rs7568458	T	A	0·551	-0·060	0·010	3·62E-10
rs4593108	C	G	0·795	0·071	0·012	8·82E-10
rs3918226	C	T	0·935	-0·133	0·022	1·69E-09
rs6689306	G	A	0·552	-0·056	0·009	2·60E-09
rs17678683	T	G	0·912	-0·099	0·017	3·00E-09
rs1199338	A	C	0·838	-0·074	0·012	3·90E-09
rs56062135	C	T	0·794	0·070	0·012	4·52E-09
rs11191416	T	G	0·873	0·079	0·014	4·65E-09
rs10840293	A	G	0·550	0·055	0·010	1·28E-08
rs10139550	C	G	0·577	-0·055	0·010	1·38E-08
rs16986953	G	A	0·895	-0·085	0·015	1·45E-08
rs56336142	T	C	0·807	0·067	0·012	1·85E-08
rs7212798	T	C	0·853	-0·080	0·014	1·88E-08
rs515135	C	T	0·792	0·067	0·012	3·09E-08
rs663129	G	A	0·743	-0·058	0·011	3·20E-08
rs8042271	A	G	0·098	-0·097	0·018	3·68E-08
rs17087335	G	T	0·785	-0·061	0·011	4·59E-08

SNP, single nucleotide polymorphism; se, standard error; eaf, effect allele frequency.

C. Self-reported high cholesterol || id: UKB-a:108

SNP	effect allele	other allele	eaf	beta	se	p-value
rs7412	T	C	0·081	-0·037	0·001	4·73E-145
rs6511720	T	G	0·119	-0·026	0·001	2·77E-100
rs12740374	T	G	0·222	-0·018	0·001	1·38E-81
rs1367117	A	G	0·339	0·016	0·001	1·53E-76
rs964184	C	G	0·868	-0·022	0·001	2·6E-75
rs28601761	G	C	0·422	-0·014	0·001	1·04E-66
rs11591147	T	G	0·017	-0·048	0·003	1·87E-55
rs4299376	T	G	0·677	-0·011	0·001	3·82E-39
rs74617384	T	A	0·081	0·019	0·001	6·37E-39
rs12916	C	T	0·401	0·01	0·001	4·01E-38
rs1260326	C	T	0·607	-0·01	0·001	3·17E-36
rs117733303	G	A	0·019	0·031	0·003	2·99E-26
rs112019714	C	T	0·028	0·025	0·002	2·06E-25
rs1883711	C	G	0·031	0·024	0·002	4·52E-25
rs2738447	C	A	0·592	0·008	0·001	7·37E-24
rs7534572	G	C	0·646	0·008	0·001	1·72E-22
rs58542926	T	C	0·076	-0·015	0·002	2·4E-22
rs635634	T	C	0·186	0·01	0·001	6·94E-22
rs77542162	G	A	0·023	0·025	0·003	1·51E-21
rs34042070	G	C	0·186	0·009	0·001	3·53E-20
rs4704727	G	T	0·66	0·007	0·001	5·23E-19
rs10410835	C	T	0·524	0·007	0·001	1·25E-18
rs2244608	G	A	0·315	0·007	0·001	2·50E-16
rs472495	T	G	0·651	0·006	0·001	1·61E-14
rs1883025	T	C	0·253	-0·007	0·001	2·42E-14
rs622871	G	A	0·686	0·007	0·001	3·39E-14
rs10260606	C	G	0·182	0·008	0·001	7·53E-14
rs10096633	T	C	0·123	-0·009	0·001	1·09E-13
rs7707394	A	G	0·354	0·006	0·001	1·31E-13
rs1601935	T	G	0·655	-0·006	0·001	3·28E-13
rs57180587	T	A	0·146	0·008	0·001	3·89E-13
rs2237107	G	C	0·204	-0·007	0·001	1·19E-12
rs3127580	T	C	0·158	0·008	0·001	1·92E-12
rs6090040	C	A	0·52	-0·006	0·001	3·21E-12
rs4470903	G	C	0·218	0·007	0·001	3·85E-12
rs59950280	A	G	0·331	0·006	0·001	1·01E-11
rs58691354	T	C	0·156	0·007	0·001	1·48E-11
rs10504255	A	G	0·662	-0·006	0·001	1·57E-11
rs12471811	T	A	0·107	0·008	0·001	5·96E-11
rs2125345	C	T	0·294	-0·006	0·001	1·19E-10
rs6458349	A	G	0·723	0·006	0·001	2·10E-10
rs11580878	G	A	0·497	-0·005	0·001	2·94E-10
rs10804330	C	T	0·43	-0·005	0·001	5·99E-10
rs34707604	C	T	0·257	0·006	0·001	9·24E-10
rs7012637	A	G	0·475	0·005	0·001	2·30E-09
rs7213086	G	C	0·553	0·005	0·001	2·43E-09
rs9376091	T	C	0·262	-0·005	0·001	5·72E-09
rs56299331	T	C	0·202	0·006	0·001	7·55E-09
rs28807203	C	A	0·049	-0·011	0·002	7·97E-09
rs456598	A	G	0·142	0·007	0·001	8·66E-09
rs2618567	T	G	0·659	-0·005	0·001	1·57E-08
rs73534263	G	A	0·107	0·007	0·001	2·94E-08
rs3918226	T	C	0·082	0·008	0·001	2·99E-08
rs799157	C	T	0·957	-0·011	0·002	3·16E-08

SNP, single nucleotide polymorphism; se, standard error; eaf, effect allele frequency.

Table S6. Leave-one-out analysis of the three exposures causally linked to ALS

A. LDL cholesterol || id:300

SNP	beta	se	p-value
rs10195252	0·112	0·038	0·003
rs10490626	0·110	0·038	0·004
rs10832962	0·111	0·038	0·003
rs10893499	0·117	0·037	0·002
rs10903129	0·109	0·038	0·004
rs10947332	0·109	0·038	0·004
rs112201728	0·110	0·038	0·004
rs11563251	0·113	0·038	0·003
rs11591147	0·132	0·038	0·001
rs1169288	0·111	0·038	0·004
rs12066643	0·110	0·038	0·004
rs1250229	0·113	0·037	0·002
rs12721109	0·113	0·039	0·004
rs12748152	0·112	0·038	0·003
rs12916	0·116	0·038	0·002
rs13206249	0·109	0·038	0·004
rs13277801	0·111	0·038	0·004
rs1367117	0·098	0·039	0·011
rs1408272	0·109	0·038	0·004
rs1564348	0·110	0·038	0·004
rs16831243	0·110	0·038	0·004
rs16891156	0·109	0·038	0·004
rs17404153	0·112	0·038	0·003
rs174583	0·113	0·038	0·003
rs1800961	0·113	0·038	0·003
rs1801689	0·113	0·038	0·003
rs1883025	0·113	0·038	0·003
rs2000999	0·109	0·038	0·004
rs2030746	0·112	0·038	0·003
rs2073547	0·111	0·038	0·004
rs2228603	0·110	0·038	0·004
rs2315065	0·106	0·038	0·005
rs2328223	0·112	0·038	0·003
rs2390536	0·111	0·038	0·004
rs2419604	0·107	0·037	0·004
rs247616	0·109	0·038	0·004
rs2495495	0·111	0·038	0·003
rs2587534	0·110	0·038	0·004
rs2642438	0·111	0·038	0·003
rs267733	0·117	0·036	0·001
rs2710642	0·110	0·038	0·004
rs2737252	0·108	0·038	0·004
rs2738459	0·104	0·037	0·005
rs2886232	0·109	0·038	0·004
rs2965157	0·112	0·038	0·004
rs314253	0·113	0·038	0·003
rs3184504	0·105	0·036	0·004
rs364585	0·109	0·038	0·004
rs3757354	0·113	0·038	0·003
rs3780181	0·107	0·037	0·004
rs4253776	0·112	0·038	0·003
rs4530754	0·111	0·038	0·004
rs4722551	0·112	0·038	0·003
rs4942486	0·114	0·038	0·002
rs4970712	0·112	0·038	0·003
rs5763662	0·109	0·038	0·004
rs579459	0·105	0·038	0·006
rs6016373	0·112	0·038	0·003
rs6065311	0·106	0·038	0·005
rs646776	0·112	0·040	0·005
rs6504872	0·117	0·037	0·001
rs6511720	0·118	0·040	0·003
rs6544713	0·111	0·038	0·004

rs6709904	0·114	0·038	0·002
rs676388	0·112	0·038	0·003
rs6818397	0·111	0·038	0·004
rs6882076	0·110	0·038	0·004
rs6909746	0·111	0·038	0·003
rs7254892	0·112	0·041	0·007
rs72902576	0·109	0·038	0·004
rs7534572	0·110	0·038	0·004
rs7551981	0·106	0·038	0·005
rs75687619	0·102	0·037	0·006
rs7640978	0·110	0·038	0·004
rs7832643	0·110	0·038	0·004
rs8017377	0·113	0·038	0·003
rs9875338	0·109	0·038	0·004
rs9987289	0·114	0·038	0·003
All	0·111	0·038	0·003

id: specific code attributed to each trait on MR Base, SNP: single nucleotide polymorphism, se: standard error

2. Coronary heart disease || id:7

SNP	beta	se	pval
rs10080815	0.059	0.032	0.064
rs10840293	0.067	0.031	0.028
rs11065979	0.076	0.030	0.011
rs11191416	0.056	0.030	0.064
rs11556924	0.070	0.031	0.024
rs115654617	0.060	0.032	0.063
rs11838776	0.063	0.032	0.049
rs1199338	0.063	0.032	0.049
rs12202017	0.061	0.032	0.057
rs1412444	0.060	0.032	0.059
rs16986953	0.064	0.031	0.041
rs17087335	0.061	0.032	0.056
rs17678683	0.059	0.032	0.062
rs1870634	0.067	0.031	0.033
rs2107595	0.064	0.032	0.045
rs2128739	0.063	0.032	0.050
rs2487928	0.070	0.030	0.022
rs2519093	0.056	0.031	0.071
rs2681472	0.064	0.032	0.042
rs28451064	0.058	0.032	0.071
rs2891168	0.051	0.035	0.142
rs3918226	0.065	0.032	0.039
rs4420638	0.057	0.031	0.068
rs4468572	0.064	0.032	0.044
rs4593108	0.062	0.032	0.051
rs515135	0.056	0.030	0.068
rs55730499	0.058	0.033	0.082
rs56062135	0.058	0.032	0.064
rs56289821	0.060	0.032	0.062
rs56336142	0.063	0.032	0.046
rs663129	0.063	0.032	0.049
rs6689306	0.059	0.032	0.062
rs67180937	0.059	0.032	0.065
rs7528419	0.059	0.032	0.068
rs8042271	0.060	0.032	0.056
rs9349379	0.068	0.033	0.039
rs9970807	0.065	0.032	0.040
All	0.062	0.031	0.047

3. Self-reported high cholesterol || id: UKB-a:108

SNP	beta	se	pval
rs10096633	0.900	0.244	0.0002
rs10260606	0.872	0.244	0.0004
rs10410835	0.861	0.244	0.0004
rs10504255	0.892	0.244	0.0003
rs10804330	0.883	0.244	0.0003
rs112019714	0.794	0.245	0.0012
rs11580878	0.859	0.244	0.0004
rs11591147	0.976	0.248	0.0001
rs117733303	0.856	0.245	0.0005
rs12471811	0.874	0.244	0.0003
rs1260326	0.872	0.247	0.0004
rs12740374	0.875	0.252	0.0005
rs12916	0.921	0.247	0.0002
rs1367117	0.773	0.251	0.0021
rs1601935	0.860	0.244	0.0004
rs1883025	0.904	0.244	0.0002
rs1883711	0.890	0.245	0.0003
rs2125345	0.836	0.244	0.0006
rs2237107	0.873	0.244	0.0003
rs2244608	0.868	0.244	0.0004
rs2618567	0.893	0.243	0.0002
rs2738447	0.869	0.245	0.0004
rs28807203	0.880	0.243	0.0003
rs34042070	0.853	0.245	0.0005
rs3918226	0.884	0.243	0.0003
rs4299376	0.878	0.247	0.0004
rs4470903	0.891	0.244	0.0003
rs456598	0.877	0.243	0.0003
rs4704727	0.864	0.245	0.0004
rs472495	0.808	0.244	0.0009
rs56299331	0.897	0.244	0.0002
rs57180587	0.878	0.244	0.0003
rs58542926	0.848	0.245	0.0005
rs58691354	0.898	0.244	0.0002
rs59950280	0.874	0.244	0.0003
rs6090040	0.883	0.244	0.0003
rs622871	0.871	0.244	0.0004

rs635634	0.825	0.245	0.0008
rs6458349	0.891	0.244	0.0003
rs6511720	0.918	0.255	0.0003
rs7012637	0.845	0.244	0.0005
rs73534263	0.847	0.243	0.0005
rs7412	0.895	0.258	0.0005
rs74617384	0.856	0.246	0.0005
rs7534572	0.869	0.245	0.0004
rs77542162	0.860	0.243	0.0004
rs799157	0.885	0.243	0.0003
rs9376091	0.889	0.244	0.0003
rs964184	0.808	0.252	0.0013
All	0.871	0.243	0.0003

Table S7. Horizontal pleiotropy, heterogeneity, and directionality analyses of the three exposures causally linked to ALS

		Horizontal pleiotropy		
**Exposure**	**Directionality**	**egger intercept**	**se**	**p-value**
LDL cholesterol || id:300	+	0·0002	0·0035	0·9590
Coronary heart disease || id:7	+	0·011	0·007	0·137
Self-reported: high cholesterol || id:UKB-a:108	+	0·001	0·005	0·777

	Heterogeneity					
**Exposure**	**Maximum likelihood Q**	**Maximum likelihood Q p-value**	**IVW Q**	**IVW Q p-value**	**MR Egger Q**	**MR Egger Q p-value**
LDL cholesterol || id:300	112·71	0·005	111·361	0·006	112·823	0·004*
Coronary heart disease || id:7	39·665	0·310	38·622	0·352	37·262	0·365
Self-reported: high cholesterol || id:UKB-a:108	41·973	0·717	41·196	0·746	41·992	0·680

id, specific code attributed to each trait on *MR Base;* se, standard error; Q, Cochran's Q test; LDL, low density lipoprotein; IVW, Inverse variance weighted

* Cochran's Q test identified the presence of slight heterogeneity for LDL cholesterol driven by the following SNPs: rs10893499, rs11591147, rs267733, rs2738459, rs3184504, rs3780181, rs6504872, rs75687619.

Radial analysis showed that the association was still present after removing these variants (inverse-variance weighted beta = 0.11, standard error (SE) = 0.035, p-value = 0.0019).

Table S8. Reverse causality analysis

Exposure	Outcome	MR test	No. of SNPs	beta	se	p-value
ALS	Coronary heart disease || id:7	MR Egger	5	0·173	0·118	0·24
ALS	Coronary heart disease || id:7	Weighted median	5	0·051	0·055	0·351
ALS	Coronary heart disease || id:7	Inverse variance weighted	5	0·015	0·043	0·729
ALS	Self-reported: high cholesterol || id:UKB-a:108	MR Egger	5	-0·001	0·008	0·896
ALS	Self-reported: high cholesterol || id:UKB-a:108	Weighted median	5	-0·004	0·004	0·234
ALS	Self-reported: high cholesterol || id:UKB-a:108	Inverse variance weighted	5	-0·004	0·003	0·167

id, specific code attributed to each trait by *MR Base;* se, standard error; No. of SNPs, number of SNPs; ALS, amyotrophic lateral sclerosis; MR, Mendelian randomization

Table S9. Genetic risk profiling of LDL cholesterol in ALS subtypes

	C9orf72 carriers versus non-carriers		
	First	Second	Third
Odds ratio	1	0,904	0,952
CI (95%)		(0,727-1,123)	(0,793-1,148)
p-value	0.89		

	Familial versus sporadic		
	First	Second	Third
Odds ratio	1	1,004	1,039
CI (95%)		(0,827-1,218)	(0,880-1,23)
p-value	0.76		

	Gender		
	t	df	p-value
Female cases versus male cases	0,07	7306,8	0,943
Female controls versus male controls	1,362	21282	0,173

CI, confidence interval; df, degrees of freedom; t-student test

Table S10. Bayesian colocalization analysis of the 78 independent regions associated to LDL cholesterol and ALS

Region	NSNPs	PP.H0	PP.H1	PP.H2	PP.H3	PP.H4
chr5:73656720-75651786	2066	2.82E-32	1.18E-05	2.39E-29	2.48E-05	1.00
chr5:155390511-157388284	1961	1.85E-13	1.96E-04	4.68E-11	0.04	0.96
chr6:115374815-117352060	1673	2.06E-21	1.98E-16	6.68E-06	0.64	0.36
chr20:38156052-40153172	2016	2.84E-09	6.97E-05	3.52E-05	0.86	0.14
chr20:38727714-40723640	1932	4.69E-09	3.06E-04	1.39E-05	0.90	0.10
chr2:164514239-166513062	1733	2.22E-24	5.54E-15	3.68E-10	0.92	0.08
chr17:44787313-46438249	1071	1.45E-06	1.37E-02	9.54E-05	0.91	0.08
chr2:20275591-22274537	2169	4.09E-23	1.10E-01	3.13E-22	0.84	0.05
chr2:20265682-22263589	2171	4.69E-23	1.09E-01	3.61E-22	0.84	0.05
chr2:233679644-235679245	2465	5.00E-19	4.06E-14	1.20E-05	0.97	0.03
chr2:215308820-217304152	1903	4.77E-09	1.39E-06	3.36E-03	0.98	0.02
chr1:233850211-235832555	1848	1.71E-17	1.40E-15	1.20E-02	0.98	8.03E-03
chr7:43582693-45582209	1620	1.47E-08	5.97E-07	2.40E-02	0.97	3.84E-03
chr8:144075281-146019378	656	5.67E-08	2.68E-05	2.11E-03	1.00	2.08E-03
chr8:115664240-117661566	1626	1.85E-20	3.03E-04	6.09E-17	1.00	1.43E-03
chr12:110888857-112879955	1132	1.00E-17	8.01E-05	1.25E-13	1.00	1.20E-03
chr1:91995681-93986217	1394	7.22E-29	2.47E-01	2.20E-28	0.75	9.67E-04
chr11:60609972-62609576	1328	6.50E-12	1.04E-09	6.19E-03	0.99	9.09E-04
chr9:135154341-137153882	2150	7.93E-14	2.15E-07	3.68E-07	1.00	8.79E-04
chr7:24993067-26991230	2429	3.82E-27	5.30E-08	7.20E-20	1.00	6.34E-04
chr17:63211340-65201873	2145	4.37E-41	4.08E-38	1.07E-03	1.00	3.29E-04
chr1:54721930-56718115	1864	7.95E-159	2.88E-154	2.76E-05	1.00	2.98E-04
chr1:26138451-28137264	885	1.01E-07	2.04E-03	4.92E-05	1.00	9.68E-05
chr2:117836737-119834827	2007	5.91E-08	2.23E-04	2.65E-04	1.00	7.20E-05
chr14:23884137-25883885	2038	4.17E-15	1.57E-08	2.65E-07	1.00	7.11E-05
chr7:20487120-22484651	2647	4.45E-13	4.59E-09	9.70E-05	1.00	4.58E-05
chr11:115648917-117648917	2228	1.61E-12	1.61E-04	1.00E-08	1.00	2.42E-05
chr2:62149557-64149557	1103	1.50E-09	3.20E-04	4.68E-06	1.00	2.36E-05

chr2:134762344-136762344	1349	7.30E-21	4.94E-03	1.47E-18	1.00	1.90E-05
chr13:31953388-33953388	2087	2.93E-27	4.06E-14	7.22E-14	1.00	1.16E-05
chr16:71108093-73108093	1294	1.09E-16	1.04E-05	1.05E-11	1.00	1.14E-05
chr1:61999675-63999675	1883	1.75E-10	3.61E-03	4.84E-08	1.00	9.27E-06
chr17:6091650-8091650	1359	1.09E-20	3.51E-12	3.10E-09	1.00	4.13E-06
chr4:2434885-4434885	1120	1.21E-16	1.28E-11	9.42E-06	1.00	1.32E-06
chr1:55240278-57240278	2108	4.23E-178	3.57E-170	1.19E-08	1.00	8.72E-07
chr19:18329924-20329924	1242	3.79E-30	1.88E-06	2.02E-24	1.00	7.59E-07
chr1:149958836-151958836	902	8.98E-25	3.93E-15	2.29E-10	1.00	7.49E-07
chr1:219970028-221970028	1690	4.75E-19	2.52E-13	1.88E-06	1.00	6.25E-07
chr10:112944271-114944271	2068	5.30E-28	1.00E-16	5.29E-12	1.00	4.00E-07
chr22:29378703-31378703	1518	1.98E-14	7.39E-05	2.67E-10	1.00	1.65E-07
chr19:10202306-12202306	921	9.54E-16	1.20E-09	7.93E-07	1.00	1.35E-07
chr16:55989590-57989590	1801	7.70E-16	6.38E-08	1.21E-08	1.00	1.20E-07
chr19:10238473-12238473	926	6.75E-16	9.72E-10	6.94E-07	1.00	1.18E-07
chr8:58353534-60353534	2118	3.83E-27	5.85E-21	6.55E-07	1.00	8.21E-08
chr19:48211969-50211969	1156	1.14E-16	2.74E-06	4.16E-11	1.00	5.62E-08
chr2:43080324-45080324	2448	1.46E-36	1.58E-27	9.24E-10	1.00	4.51E-08
chr2:43073881-45073881	2458	1.08E-36	1.26E-27	8.62E-10	1.00	4.27E-08
chr2:120309488-122309488	1374	3.18E-19	5.25E-12	6.05E-08	1.00	3.46E-08
chr1:24768937-26768937	1312	3.28E-39	4.14E-06	7.91E-34	1.00	2.84E-08
chr6:15175022-17175022	2088	2.99E-21	6.32E-07	4.72E-15	1.00	8.42E-09
chr6:15127407-17127407	2118	2.16E-21	6.21E-07	3.48E-15	1.00	8.29E-09
chr1:108818530-110818530	1400	2.52E-16	4.53E-07	5.57E-10	1.00	1.99E-09
chr9:1640759-3640759	2954	4.79E-28	5.14E-20	9.32E-09	1.00	1.87E-09
chr22:45629479-47629479	1824	1.01E-45	5.31E-13	1.90E-33	1.00	1.70E-09
chr1:54496556-56496556	1869	2.08E-159	1.97E-149	1.05E-10	1.00	7.57E-10
chr1:54505647-56505647	1873	1.19E-159	1.23E-149	9.71E-11	1.00	7.05E-10
chr12:120416650-122416650	1364	1.14E-21	1.52E-09	7.52E-13	1.00	6.03E-10
chr3:131163200-133163200	2072	3.96E-35	9.31E-25	4.26E-11	1.00	5.56E-10
chr8:8183358-10183358	3198	2.69E-19	2.74E-10	9.83E-10	1.00	3.22E-10

chr6:31677440-33677440	3183	3.86E-23	3.07E-14	1.25E-09	1.00	2.12E-10
chr17:66150176-68150176	1790	6.81E-33	1.09E-10	6.26E-23	1.00	1.11E-10
chr5:121855416-123855416	2110	2.78E-21	6.91E-09	4.02E-13	1.00	5.91E-11
chr11:125241979-127241979	2374	4.41E-26	7.81E-16	5.64E-11	1.00	1.33E-11
chr20:11962718-13962718	2314	2.20E-27	4.68E-10	4.70E-18	1.00	2.33E-12
chr9:106664301-108664301	2400	2.13E-30	2.52E-14	8.45E-17	1.00	1.80E-12
chr19:44447221-46447221	1333	5.46E-56	6.69E-45	8.17E-12	1.00	1.71E-12
chr19:44399344-46399344	1339	1.90E-56	4.61E-45	4.11E-12	1.00	8.61E-13
chr19:44389596-46389596	1333	2.69E-56	7.39E-45	3.64E-12	1.00	7.62E-13
chr19:44176340-46176340	1311	1.01E-56	4.48E-45	2.25E-12	1.00	4.71E-13
chr3:31533010-33533010	1362	7.35E-43	3.45E-27	2.13E-16	1.00	1.69E-15
chr11:17656271-19656271	1831	5.15E-36	1.48E-15	3.48E-21	1.00	5.93E-16
chr6:160108144-162108144	2407	4.24E-35	5.19E-18	8.18E-18	1.00	1.08E-17
chr6:159578860-161578860	2506	3.48E-36	3.18E-18	1.09E-18	1.00	3.65E-18
chr6:159608804-161608804	2517	3.18E-36	3.27E-18	9.72E-19	1.00	3.59E-18
chr6:159551486-161551486	2516	1.71E-36	2.41E-18	7.08E-19	1.00	2.71E-18
chr20:42042364-44042364	1573	2.48E-41	5.97E-20	4.16E-22	1.00	1.23E-21
chr6:24842951-26842951	3005	8.22E-49	9.06E-21	9.07E-29	1.00	1.62E-23
chr20:16845921-18845921	2208	1.81E-48	8.29E-22	2.18E-27	1.00	1.52E-24

NSNPS, Number of SNPs; PP.H0, Posterior probability of no shared causal variant in the region; PP.H1, Posterior probability of a causal ALS variant but no LDL cholesterol variant; PP.H2, Posterior probability of a causal LDL cholesterol variant but no ALS variant; PP.H3: Posterior probability of both traits to have a different causal variant within the analysed region; PP.H4, Posterior probability of a shared causal variant within the analysed region

Table S11. Summary of the cohorts included in the exposures of interest and the outcome

EXPOSURE		OUTCOME
**LDL Cholesterol Pubmed ID: 24097068**	**Coronary Heart Disease Pubmed ID: 26343387**	**Amyotrophic lateral slerosis Pubmed ID: 29566793**
ADVANCE	ADVANCE	NEI Age-Related Eye Disease Study (AREDS)
AIDHS/SDS	AGES	Framingham Cohort
AMC-PAS	ARIC	High Density SNP Association Analysis of Melanoma
AMISH	BAS	CIDR: The NeuroGenetics Research Consortium Parkinson's Disease Study
BC58	CARDIOGENICS	GENEVA Genetics of Early Onset Stroke (GEOS) Study
CLHNS	CAS	Genes and Blood Clotting Study (GABC)
D2D 2007 (T2D)	CCGB	Woman's Health Initiative (WHI GARNET)
D2D 2007 (controls)	COROGENE	Polycystic Ovary Syndrome Genetics (POLYGEN)
deCODE	DUKE	Alzheimer's Disease Genetics Consortium Genome Wide Association Study
DIAGEN (T2D)	EGCUT	Autopsy-Confirmed Parkinson Disease GWAS Consortium (APDGC)
DIAGEN (controls)	FamHS	NIA Long Life Family Study (LLFS)
DILGOM	FGENTCARD	The Genetic Architecture of Smoking and Smoking Cessation
DPS (T2D)	FHS	A Genome-Wide Association Study of Fuchs' Endothelial Corneal Dystrophy
DPS (controls)	GenRIC	Health and Retirement Study (HRS)
DRAGON (TAICHI)	GerMIFS I-IV	NINDS Stroke Genetics Network (SiGN)
DR'S EXTRA (T2D)	GoDARTS	GWAS on Selected WHI Hormone Trial European Americans
DR'S EXTRA (controls)	HPS	NCI Non-Hodgkin Lymphoma GWAS
EAS	HSDS	Barrett's and Esophageal Adenocarcinoma Genetic Susceptibility Study (BEAGESS)
EGCUT	BioMe Biobank Program (IPM-HispAmer, EurAmer, AfrAmer)	LNG
Ely	ITH	
EPIC-CAD cases	LIFE-Heart	
(EPIC-Norfolk CAD set)	LOLIPOP	
EPIC-T2D cases	LURIC	
(EPIC-Norfolk T2D set)	MAYOVDB	
EPIC-T2D controls	MedStar	
FBPP	MIGen	
Fenland	OHGS	
FINCAVAS	PennCATH	
FUSION2 (T2D)	PIVUS	
FUSION2 (controls)	PREDICTCVD	
GLACIER	PROCARDIS	
Go-DARTs	PROMIS	
GXE	PROSPER	
HALST (TAICHI)	RS	
HUNT (T2D)	SDS/AIDHS	
HUNT (controls)	THISEAS	
IMPROVE	TWINGENE	
KORA F3	ULSAM	
KORA F4	WGHS	

MRC/UVRIGPC	WTCCC
LURIC (cases)	
LURIC (controls)	
MDC	
METSIM (T2D)	
METSIM (controls)	
NFBC86	
NSHD	
PIVUS	
PROMIS	
SAPPHIRe (TAICHI)	
SardiNIA	
SCARFSHEEP	
SEY	
SPT	
STR	
TACT (TAICHI)	
TCAD (TAICHI)	
TCAGEN (TAICHI)	
THISEAS	
TROMSO (T2D)	
TROMSO (controls)	
TUDR (TAICHI)	
ULSAM	
WHII	

Description of the significant phenotypes genetically correlated with ALS and included in the UK Biobank dataset

“Fluid intelligence score”
https://biobank.ctsu.ox.ac.uk/crystal/label.cgi?id=100027
This category contains data on questions designed to assess 'Fluid intelligence' (i.e. the capacity to solve problems that require logic and reasoning ability, independent of acquired knowledge). The participant has 2 minutes to complete as many questions as possible from the test. This test was incorporated into the touchscreen towards the end of recruitment.”

“Qualifications”
https://biobank.ctsu.ox.ac.uk/crystal/refer.cgi?id=100471
“This category contains data from the touchscreen questionnaire designed to assess the question: Which of the following qualifications do you have? You can select more than one: College or University Degree, A levels, AS levels or equivalent, O levels/GCSEs or equivalent, CSEs or equivalent, NVQ or HND or HNC or equivalent, Other professional qualifications, eg: nursing, teaching, none of the above.

“Types of transport used (excluding work): Walk/ Public transport:
http://biobank.ctsu.ox.ac.uk/crystal/field.cgi?id=6162
This category contains data on questions designed to assess the question:” In the last 4 weeks, which forms of transport have you used most often to get about? (Not including any journeys to and from work; you can select more than one answer)", Choose between: “car/motor vehicle, walk, public transport, none of the above, prefer not to answer.”

“Types of physical activity in last 4 weeks: Light DIY”
https://biobank.ctsu.ox.ac.uk/crystal/field.cgi?id=6164
Current Field was collected from all participants who indicated that they spent time doing light DIY (e.g. pruning, watering the lawn) in the previous 4 weeks, as defined by their answers to

“Types of physical activity in last 4 weeks: Walking for pleasure”
https://biobank.ctsu.ox.ac.uk/crystal/field.cgi?id=6164
Current Field was collected from all participants who indicated that they spent time walking for pleasure in the previous 4 weeks, as defined by their answers to the questionnaire.

“Duration of moderate activity”
https://biobank.ctsu.ox.ac.uk/crystal/field.cgi?id=894
Current Field was collected from participants who indicated that they did moderate physical activities for 10 minutes on at least 1 day per week in the previous 4 weeks, as defined by their answers to the questionnaire.

“Job involves mainly walking or standing”
https://biobank.ctsu.ox.ac.uk/crystal/field.cgi?id=806
Current Field was collected from the participants who indicated they were in paid employment or self-employed as defined by their answers to the questionnaire.

“Exposure to tobacco smoke at home”:
https://biobank.ctsu.ox.ac.uk/crystal/field.cgi?id=1269
“This category contains data from the touchscreen questionnaire designed to assess the question: "At home, about how many hours per week are you exposed to other people's tobacco smoke?". Please give the number of hours in a typical week over the
last year that you have been exposed to other people's tobacco smoke in the home environment

“Light smokers (at least 100 smokes in lifetime)”
https://biobank.ctsu.ox.ac.uk/crystal/field.cgi?id=2644
Current Field was collected from participants who indicated they smoked occasionally or Just tried once or twice, as defined by their answers to the questionnaire.

“Frequency of tenseness / restlessness in last 2 weeks”
https://biobank.ctsu.ox.ac.uk/crystal/field.cgi?id=2070
“This category contains data from the touchscreen questionnaire designed to assess the question: "Over the past two weeks, how often have you felt tense, fidgety or restless?"

International ALS Genomics Consortium

Yevgeniya Abramzon[1,2], Sampath Arepalli[3], Robert H. Baloh[4], Robert Bowser[5], Christopher B. Brady[6], Alexis Brice[7,8], James Broach[9], Roy H. Campbell[10], William Camu[11,12], Ruth Chia[1], Adriano Chiò[13,14], John Cooper-Knock[15], Daniele Cusi[16], Jinhui Ding[17], Carsten Drepper[18], Vivian E. Drory[19], Travis L. Dunckley[20], John D. Eicher[21], Faraz Faghri[10,22], Eva Feldman[23], Mary Kay Floeter[24], Pietro Fratta[2], Joshua T. Geiger[25], Glenn Gerhard[20], J. Raphael Gibbs[17], Summer B. Gibson[26], Jonathan D. Glass[27], John Hardy[28], Matthew B. Harms[29], Terry D. Heiman-Patterson[30,31], Dena G. Hernandez[3], Lilja Jansson[32], Freya Kamel[33], Janine Kirby[15], Neil W. Kowall[34], Hannu Laaksovirta[32], Francesco Landi[35], Isabelle Le Ber[7,8], Serge Lumbroso[36], Daniel J.L. MacGowan[37], Nicholas J. Maragakis[38], Kevin Mouzat[36], Natalie A. Murphy[1], Liisa Myllykangas[39], Mike A. Nalls[22,40], Aude Nicolas[1], Richard W. Orrell[41], Lyle W. Ostrow[38], Roger Pamphlett[42], Stuart Pickering-Brown[43], Erik Pioro[44], Hannah A. Pliner[1], Stefan M. Pulst[26], John M. Ravits[45], Alan E. Renton[1,46], Alberto Rivera[1], Wim Robbrecht[47], Ekaterina Rogaeva[48], Sara Rollinson[43], Jeffrey D. Rothstein[38], Erika Salvi[49], Sonja W. Scholz[38,50], Michael Sendtner[18], Pamela J. Shaw[15], Katie C. Sidle[28], Zachary Simmons[51], Andrew B. Singleton[22], David C. Stone[21], Raimo Sulkava[52], Pentti J. Tienari[32], Bryan J. Traynor[1,38], John Q. Trojanowski[53], Juan C. Troncoso[54], Philip Van Damme[47,55], Vivianna M. Van Deerlin[53], Ludo Van Den Bosch[47], Lorne Zinman[56].

Affiliations

1. Neuromuscular Diseases Research Section, Laboratory of Neurogenetics, National Institutes on Aging, National Institutes of Health, Porter Neuroscience Research Center, Bethesda, MD 20892, USA
2. Sobell Department of Motor Neuroscience and Movement Disorders, University College London, Institute of Neurology, London, UK
3. Genomics Technology Group, Laboratory of Neurogenetics, National Institutes on Aging, National Institutes of Health, Porter Neuroscience Research Center, Bethesda, MD 20892, USA
4. Department of Neurology, Cedars-Sinai Medical Center, Los Angeles, California, USA
5. Division of Neurology, Barrow Neurological Institute, Phoenix, Arizona, USA
6. Research and Development Service, Veterans Affairs Boston Healthcare System, Boston, MA, USA
7. Université Pierre et Marie Curie, Centre de Recherche de l'Institut du Cerveau et de la Moelle épinière, Paris, France
8. INSERM, U975, Paris, France
9. Department of Biochemistry, Penn State College of Medicine, Hershey, PA, USA
10. Department of Computer Science, University of Illinois at Urbana-Champaign, Urbana, IL, USA
11. The Institute for Neurosciences of Montpellier, Inserm UMR1051, Saint Eloi Hospital, Montpellier, France
12. Department of Neurology, Gui-de-Chauliac Hospital, Montpellier, France
13. 'Rita Levi Montalcini' Department of Neuroscience, University of Turin, Turin, Italy
14. Neuroscience Institute of Torino, Turin 10124, Italy
15. Department of Neuroscience, University of Sheffield, Sheffield, UK
16. Department of Health Sciences, University of Milan at San Paolo Hospital, Milan, Italy
17. Computational Biology Core, Laboratory of Neurogenetics, National Institutes on Aging, National Institutes of Health, Porter Neuroscience Research Center, Bethesda, MD 20892, USA

18. Institute for Clinical Neurobiology, University of Wurzburg, D-97078 Wurzburg, Germany
19. Department of Neurology, Tel-Aviv Sourasky Medical Center, Tel-Aviv, Israel
20. Department of Pathology, Penn State College of Medicine, Hershey, PA, USA
21. Genetics, Genetics and Pharmacogenomics, Merck Research Laboratories, Merck & Co., Inc., West Point, PA 19486, USA
22. Molecular Genetics Section, Laboratory of Neurogenetics, National Institutes on Aging, National Institutes of Health, Porter Neuroscience Research Center, Bethesda, MD 20892, USA
23. Department of Neurology, University of Michigan, Ann Arbor, Michigan, USA
24. Motor Neuron Disorders Unit, National Institute of Neurological Disorders and Stroke, National Institutes of Health, Bethesda, MD 20892, USA
25. Neurodegenerative Diseases Research Unit, National Institute of Neurological Disorders and Stroke, National Institutes of Health, Bethesda, MD, USA
26. Departments of Neurology, University of Utah School of Medicine, Salt Lake City, Utah, USA
27. Department of Neurology, Emory University School of Medicine, Atlanta, GA
28. Department of Molecular Neuroscience and Reta Lila Weston Laboratories, Institute of Neurology, University College London, Queen Square House, London WC1N 3BG, UK
29. Department of Neurology, Columbia University, New York, NY, USA
30. Department of Neurology, Drexel University College of Medicine, Philadelphia, PA, USA
31. Department of Neurology, Lewis Katz School of Medicine, Temple University, Philadelphia, PA, USA
32. Department of Neurology, Helsinki University Central Hospital and Molecular Neurology Programme, Biomedicum, University of Helsinki, Helsinki, FIN-02900, Finland
33. Epidemiology Branch, National Institute of Environmental Health Sciences, NC 27709, USA
34. VA Boston Healthcare System, US Department of Veteran Affairs, Boston, MA, USA
35. Center for Geriatric Medicine, Department of Geriatrics, Neurosciences and Orthopedics, Catholic University of Sacred Heart, Rome 00168, Italy
36. Service de Biochimie, CHU de Nîmes, Nîmes, France
37. Neuromuscular Division and ALS Center, Beth Israel Medical Center, Albert Einstein College of Medicine, NY, USA
38. Department of Neurology, Johns Hopkins University, Baltimore, MD 21287, USA
39. Department of Pathology, Haartman Institute/HUSLAB, University of Helsinki and Folkha¨ lsan Research Center (LM), Helsinki, FIN-02900, Finland
40. Data Tecnica International, Glen Echo, MD, USA
41. Department of Clinical Neuroscience, Institute of Neurology, University College London, London NW3 2PG, UK
42. Discipline of Pathology, Brain and Mind Centre, The University of Sydney, 94 Mallett St, Camperdown, NSW 2050 Australia
43. Faculty of Human and Medical Sciences, University of Manchester, Manchester M13 9PT, UK
44. Department of Neurology, Neuromuscular Center, Neurological Institute, Cleveland Clinic, Cleveland, Ohio, USA
45. Department of Neuroscience, University of California San Diego, La Jolla, CA, USA
46. Ronald M. Loeb Center for Alzheimer's Disease, Icahn School of Medicine at Mount Sinai, Department of Neuroscience, New York, NY 10029, USA
47. Department of Neurosciences, Experimental Neurology and Leuven Research Institute for Neuroscience and Disease, University of Leuven, Leuven, Belgium

48. Tanz Centre for Research of Neurodegenerative Diseases and Toronto Western Hospital, Division of Neurology, Department of Medicine, University of Toronto, Toronto, ON, M5S 3H2, Canada
49. Department of Health Sciences, University of Milan, Milan, Italy
50. Neurodegenerative Diseases Research Unit, National Institute of Neurological Disorders and Stroke, National Institutes of Health, Bethesda, MD 20892, USA
51. Department of Neurology, Penn State Hershey Medical Center, Hershey, PA, USA
52. Section of Geriatrics, Institute of Public Health and Clinical Nutrition, University of Eastern Finland, Kuopio, FIN-70211, Finland
53. Department of Pathology and Laboratory Medicine, University of Pennsylvania, Philadelphia, PA, USA
54. Clinical and Neuropathology Core, Johns Hopkins University School of Medicine, Baltimore, MD, USA
55. VIB, Center for Brain & Disease Research, Laboratory of Neurobiology, Leuven, Belgium
56. Division of Neurology, Department of Internal Medicine, Sunnybrook Health Sciences Centre, University of Toronto, Toronto, ON, M4N 3M5, Canada

The ITALSGEN Consortium

Alessandro Arosio[1], Marco Barberis[2], Ilaria Bartolomei[3], Stefania Battistini[4], Michele Benigni[4], Giuseppe Borghero[5], Maura Brunetti[6], Andrea Calvo[2], Stefania Cammarosano[2], Antonino Cannas[5], Antonio Canosa[2], Margherita Capasso[7], Claudia Caponnetto[8], Carla Caredda[5], Paola Carrera[9], Federico Casale[2], Sebastiano Cavallaro[10], Adriano Chiò[2,11], Tiziana Colletti[12], Francesca L. Conforti[10], Amelia Conte[13] Lucia Corrado[14], Emanuela Costantino[5], Sandra D'Alfonso[14], Antonio Fasano[15], Cinzia Femiano[16], Carlo Ferrarese[1], Nicola Fini[15], Gianluca Floris[5], Giuseppe Fuda[2], Fabio Giannini[4], Maurizio Grassano[2,17], Antonio Ilardi[2], Vincenzo La Bella[12], Serena Lattante[13], Giancarlo Logroscino[18,19], Francesco O. Logullo[20], Daniela Loi[21], Christian Lunetta[22], Gianluigi Mancardi[8], Paola Mandich[8], Jessica Mandrioli[15], Umberto Manera[2], Giuseppe Marangi[13], Kalliopi Marinou[23], Giuseppe Marrali[2], Maria Giovanna Marrosu[5], Letizia Mazzini[24], Maurizio Melis[5], Sonia Messina[25], Cristina Moglia[2], Maria Rosaria Monsurro[16], Gabriele Mora[23], Lorena Mosca[27], Patrizia Occhineri[26], Paola Origone[8], Carla Pani[5], Silvana Penco (deceased)[27], Antonio Petrucci[28], Giovanni Piccirillo[16], Angelo Pirisi[26], Fabrizio Pisano[29], Maura Pugliatti[26], Gabriella Restagno[6], Claudia Ricci[4], Maria Rita Murru[5], Nilo Riva[9], Mario Sabatelli[13], Fabrizio Salvi[3], Marialuisa Santarelli[30], Riccardo Sideri[23], Isabella Simone[18], Rossella Spataro[12], Raffaella Tanel[31], Gioacchino Tedeschi[16], Stefania Tranquilli[5], Lucio Tremolizzo[1], Francesca Trojsi[16], Paolo Volanti[32], and Marcella Zollino[13].

Affiliations

1. Neurology Unit, School of Medicine and Surgery and NeuroMI, University of Milano-Bicocca, Monza, Italy
2. 'Rita Levi Montalcini' Department of Neuroscience, University of Turin, Turin, Italy
3. Center for Diagnosis and Cure of Rare Diseases, Department of Neurology, IRCCS Institute of Neurological Sciences, Bologna, Italy
4. Department of Medical, Surgical and Neurological Sciences, University of Siena, Siena, Italy
5. Department of Neurology, Azienda Universitario Ospedaliera di Cagliari and University of Cagliari, Cagliari, Italy
6. Molecular Genetics Unit, Department of Clinical Pathology, A.S.O. O.I.R.M.-S. Anna, 10126 Turin, Italy
7. Department of Neurology, University of Chieti, Chieti, Italy
8. Department of Neurosciences, Ophthalmology, Genetics, Rehabilitation, Maternal and Child Health, IRCCS Azienda Ospedaliero-Universitaria San Martino IST, Genoa, Italy
9. Department of Neurology and Institute of Experimental Neurology (INSPE), IRCCS San Raffaele Scientific Institute, Milan, Italy
10. Institute of Neurological Sciences, National Research Council, Mangone, Cosenza, Italy
11. Neuroscience Institute of Torino, Turin 10124, Italy
12. ALS Clinical Research Center, Bio. Ne. C., University of Palermo, Palermo, Italy.
13. Centro Clinico NEMO-Roma. Neurological Institute, Catholic University and I.C.O.M.M. Association for ALS Research, Rome, Italy
14. Department of Health Sciences, University of Eastern Piedmont, Novara, Italy
15. Department of Neuroscience, S. Agostino-Estense Hospital, University of Modena and Reggio Emilia, Modena, Italy
16. Department of Medical, Surgical Neurological Metabolic and Aging Sciences, Second University of Naples, Naples, Italy
17. Azienda Ospedaliero Universitaria Citta della Salute e della Scienza, Turin, Italy
18. Department of Basic Medical Sciences, Neurosciences and Sense Organs, University of Bari, Bari, Italy

19. Department of Clinical and Research Neurology, “Pia Fondazione Cardinal G. Panico” Hospital, Tricase (LE), Bari, Italy
20. Neurological Clinic, Marche Polytechnic University, Ancona, Italy
21. Azienda Sanitaria Locale n. 2, Olbia-Tempio, Olbia, Italy
22. NeuroMuscular Omnicenter, Serena Onlus Foundation, Milan, Italy
23. Department of Neurological Rehabilitation, Fondazione Salvatore Maugeri, IRCCS, Istituto Scientifico di Milano, Milano, Italy
24. Maggiore della Carita' University Hospital, Novara, Itay
25. Department of Clinical and Experimental Medicine, University of Messina and Nemo Sud Clinical Center for Neuromuscular Diseases, Aurora Foundation, Messina, Italy
26. Department of Biomedical and Surgical Sciences, Section of Neurological, Psychiatric and Psychological Sciences, University of Ferrara, Ferrara, Italy
27. Department of Laboratory Medicine, Medical Genetics, Niguarda Ca' Granda Hospital, Milan, Italy
28. Neurology Department, San Camillo Hospital, Rome, Italy
29. Department of Neurological Rehabilitation, "Salvatore Maugeri" Clinical-Scientific Institute, Istituto di Ricovero e Cura a Carattere Scientifico, Veruno, Italy
30. Department of Medicine, Azienda Complesso Ospedaliero, San Filippo Neri, Rome, Italy
31. Department of Neurology, Santa Chiara Hospital, Trento, Italy
32. Neurorehabilitation Unit/ALS Center, Scientific Clinical Institutes (ICS) Maugeri, IRCCS, Mistretta, Messina, Italy
